# A multicomponent approach for the preparation of homoallylic alcohols†
†Raw spectra can be found at DOI: 10.17863/CAM.591

‡
‡Electronic supplementary information (ESI) available. CCDC 1483699. For ESI and crystallographic data in CIF or other electronic format see DOI: 10.1039/c6sc02581a



**DOI:** 10.1039/c6sc02581a

**Published:** 2016-07-08

**Authors:** Jian-Siang Poh, Shing-Hing Lau, Iain G. Dykes, Duc N. Tran, Claudio Battilocchio, Steven V. Ley

**Affiliations:** a Innovative Technology Centre , Department of Chemistry , University of Cambridge , Lensfield Road , Cambridge CB2 1EW , UK . Email: svl1000@cam.ac.uk ; http://www.leygroup.ch.cam.ac.uk

## Abstract

Here we report the *in situ* generation of transient allylic boronic species, by reacting TMSCHN_2_ and *E*-vinyl boronic acids, followed by their subsequent trapping with aldehydes as electrophiles to yield homoallylic alcohols. This metal-free reaction was initially discovered by the use of a flow chemistry approach to generate a variety of homoallylic alcohols in a straightforward fashion and then transferred to a batch protocol.

## Abstract 2

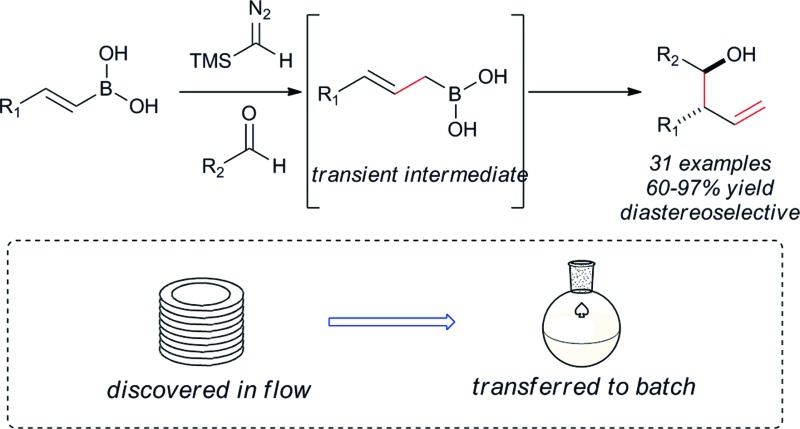
The generation of transient boronic acid species followed by their subsequent trapping with aldehydes as electrophiles to yield homoallylic alcohols, in a multicomponent and metal-free fashion.

## Introduction

**Fig. 1:**
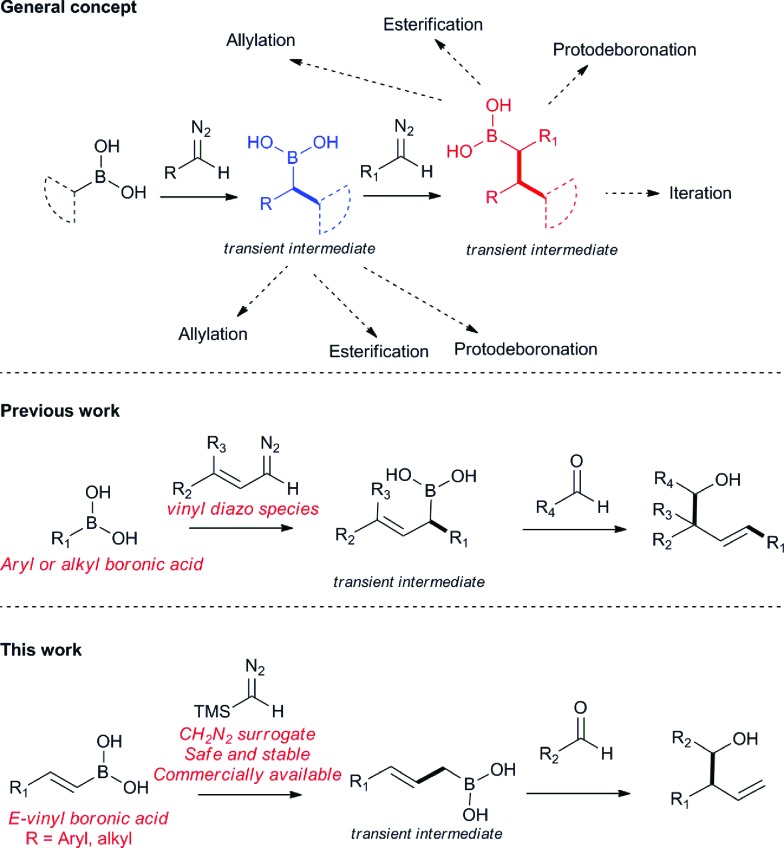
General concept for the reaction of diazo compounds and boronic acid species for the iterative C–C bond formation strategy, previous work on allylation of transient boronic acids, and current work using TMSCHN_2_ as commercially available diazo compounds.

Homoallylic alcohols are valuable synthetic building blocks, in particular, for the construction of polyketide natural products.^1–4^ Whilst they can be accessed in a metal-free fashion by crotylation/allylation methods of Roush^5–10^ and Brown,^11–19^ the instability or availability of the appropriate allyl boronic coupling partner is an important limitation.^20–22^ Furthermore, installation of side chain groups other than methyl is not always straightforward using these methods. Other procedures are known but tend to lack generality.^23–38^ In a continuation of our work in the area (Fig. 1),^39–44^ we report here a new multi-component coupling to prepare homoallylic alcohols which was first discovered using flow chemistry^45^ methods and later transferred to a batch protocol, involving reactions of vinyl boronic acids with trimethylsilyl diazomethane (TMSCHN_2_)^46–49^ to generate the allylic boronic species *in situ* which are then intercepted with aldehydes to form new C–C bonds.

## Results and discussion

**Scheme 1:**
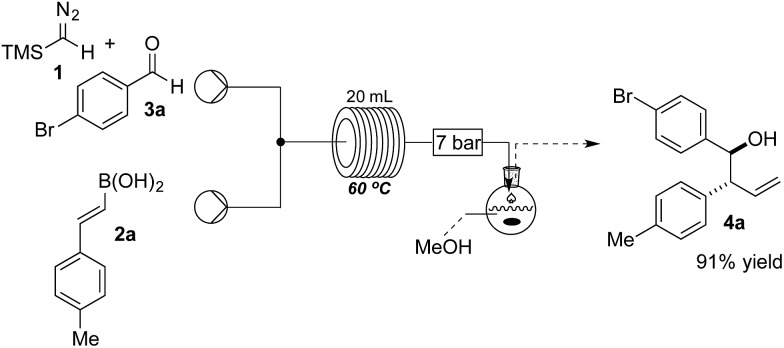
Flow set up for the continuous synthesis of homoallylic alcohols **4**.

The reaction was initially optimised with available flow equipment using TMS-diazomethane **1** (TMSCHN_2_), *E*-4-methylphenyl vinyl boronic acid **2a** and 4-bromobenzaldehyde **3a** as substrates (Scheme 1). Interestingly, early investigations showed the product to be a homoallylic alcohol (**4a**), which was obtained in good yield. We therefore initiated a rapid evaluation of the reaction parameters in order to obtain a robust system. The first attempt to define these reaction parameters employed a flow strategy whereby a solution containing the vinyl boronic acid **2a** (0.04 M, in CH_2_Cl_2_ : THF, 4 : 1 v/v) and a solution of TMSCHN_2_ **1** and aldehyde **3a** (0.066 M and 0.033 M, respectively, in CH_2_Cl_2_) were combined at a T-piece and reacted in a 20 mL perfluoroalkoxy alkanes (PFA) reactor coil (*τ* = 25 min). The exiting stream of borylated intermediate was then quenched with MeOH (2 mL), in a batch-fed integrated fashion, to yield the final product **4a**. Following this approach, we were able to rapidly identify suitable conditions, with compound **4a** being generated (0.29 mmol scale, 91% yield) at 60 °C. The choice of solvent was crucial as solvent systems that are able to strongly coordinate with the boronic acid partner (*e.g.* neat THF) can favour the formation of by-products. We were soon able to identify that a mixture of CH_2_Cl_2_ and THF (4 : 1 v/v) was the optimal solvent system to carry out the reaction continuously without causing any undesirable rate or selectivity issues on the reaction.

With this protocol in place, we were able to rapidly access a range of homoallylic alcohols by using various vinyl boronic acids **2** and aldehydes **3** (Table 1).

**Table 1:** Multicomponent homoallylic alcohol synthesis in flow^*a*^

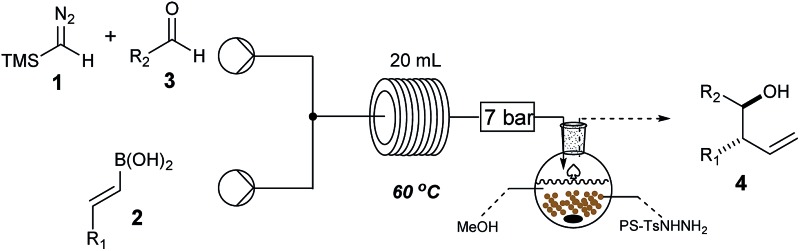
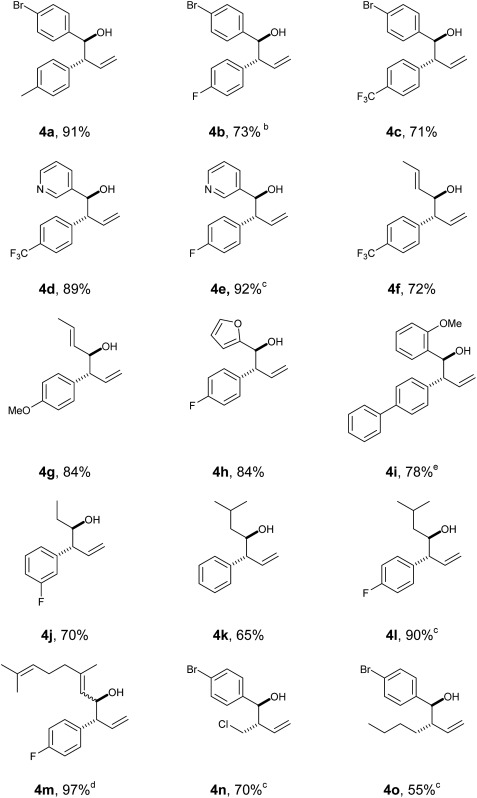

^*a*^Standard reaction conditions: solution of TMSCHN_2_ **1** and aldehyde **3** in CH_2_Cl_2_ (0.066 M and 0.033 M, respectively), solution of vinyl boronic acid **2** in CH_2_Cl_2_ : THF (4 : 1 v/v, 0.04 M), 60 °C, residence time 23 min (isolated yield); for more detail see ESI.

^*b*^Reaction performed on 10 mmol scale (analytic sample was collected at steady state for 340 min).

^*c*^Yield was measured using a ^1^H-NMR standard (1,3,5-trimethoxybenzene).

^*d*^ *E*/*Z* ratio 2 : 1.

^*e*^Solvent mixture used for the vinyl boronic acid was CH_2_Cl_2_ : THF (2 : 1 v/v, 0.04 M).

Both electron withdrawing and electron-donating substituents on the vinyl boronic partner were associated with particularly good yields (including aryl and alkyl groups). Screening of both aromatic and aliphatic aldehyde traps successfully provided the products with good-to-excellent yields in all cases. In all the examples, high degree of diastereoselectivity was observed (confirmed by X-ray crystallography data, see ESI‡).

It is worth mentioning that reacting *Z*-vinyl boronic acids under the standard conditions led to a complex mixture, suggesting that the molecular geometry of the boronic species is mechanistically relevant.

The robustness of the protocol was quickly assessed by performing a larger-scale reaction (10 mmol) which gave product **4b** in a consistent yield of 73%.

In order to facilitate downstream processing and improve the practicality of the method, any residual aldehyde was scavenged using a polymer-supported tosylhydrazine resin.^50,51^

**Scheme 2:**
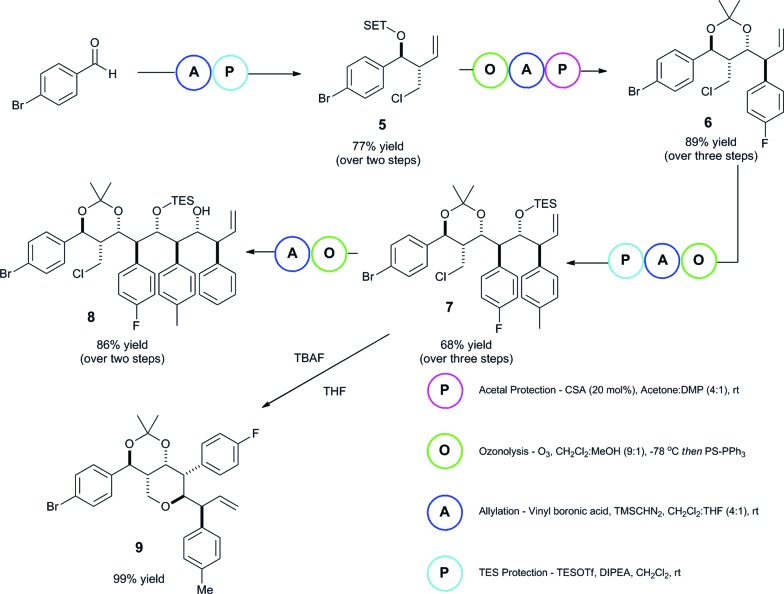
Iterative process to polyol **8** and tetrahydropyran derivative **9**.

Wishing to broaden the practical aspect of this multicomponent metal-free reaction, we decided to transfer the method directly into a batch process (Scheme 2), having taken care to safely handle the TMSCHN_2_ reagent. The optimised batch protocol was developed at room temperature with reaction times being within the range from 2 h to 16 h for similar reaction scale (0.3 mmol). The scope of the reaction under batch conditions was expanded and demonstrated the generality of the method (Table 2).

**Table 2:** Multicomponent homoallylic alcohol synthesis in batch^*a*^

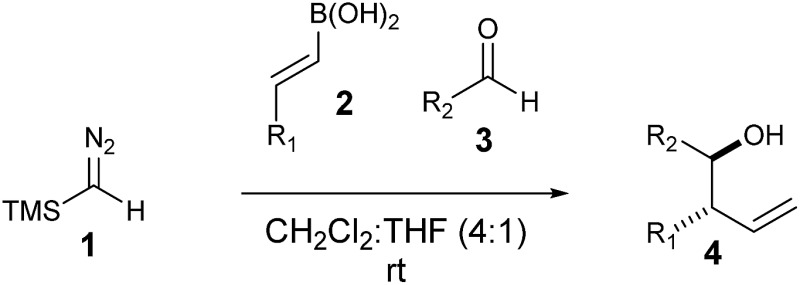
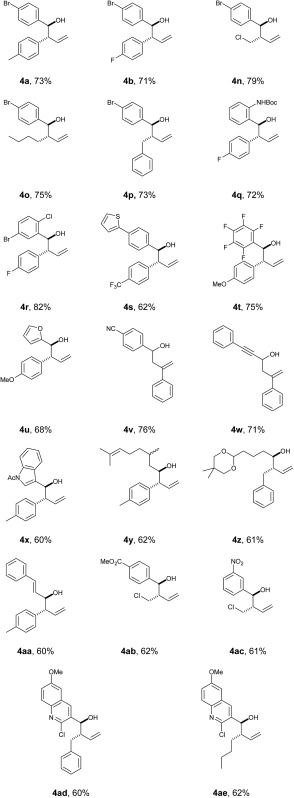

^*a*^Standard reaction conditions: 0.6 mmol of TMSCHN_2_ **1**, 0.36 mmol of vinyl boronic acid **2**, 0.3 mmol of aldehyde **3**, in CH_2_Cl_2_ : THF (4 : 1 v/v, 4 mL) at rt, 2 h to 16 h; for more detail see ESI.

The iterative importance of this methodology was then showcased in the generation of polyol backbone materials (Scheme 2). The iteration process consisted of a sequence of allylation processes. In between each allylation, the homoallylic alcohol intermediate underwent an alcohol protection stage, followed by ozonolysis of the olefin to regenerate the aldehyde component for further reaction. The sequence was repeated four times to rapidly provide a complex derivative **8** (see ESI‡ for a detailed reaction description). Analysis of the ^1^H/^13^C shifts of the acetonide for the 2^nd^ iteration product **6** confirms a twist-boat structure, indicating Felkin–Anh selectivity.^52–55^

In addition to this, we were able to selectively elaborate intermediate **7** into the corresponding tetrahydropyran derivative **9**, showing the versatility of such a method for generation of rather underrepresented molecular entities.

**Scheme 3:**
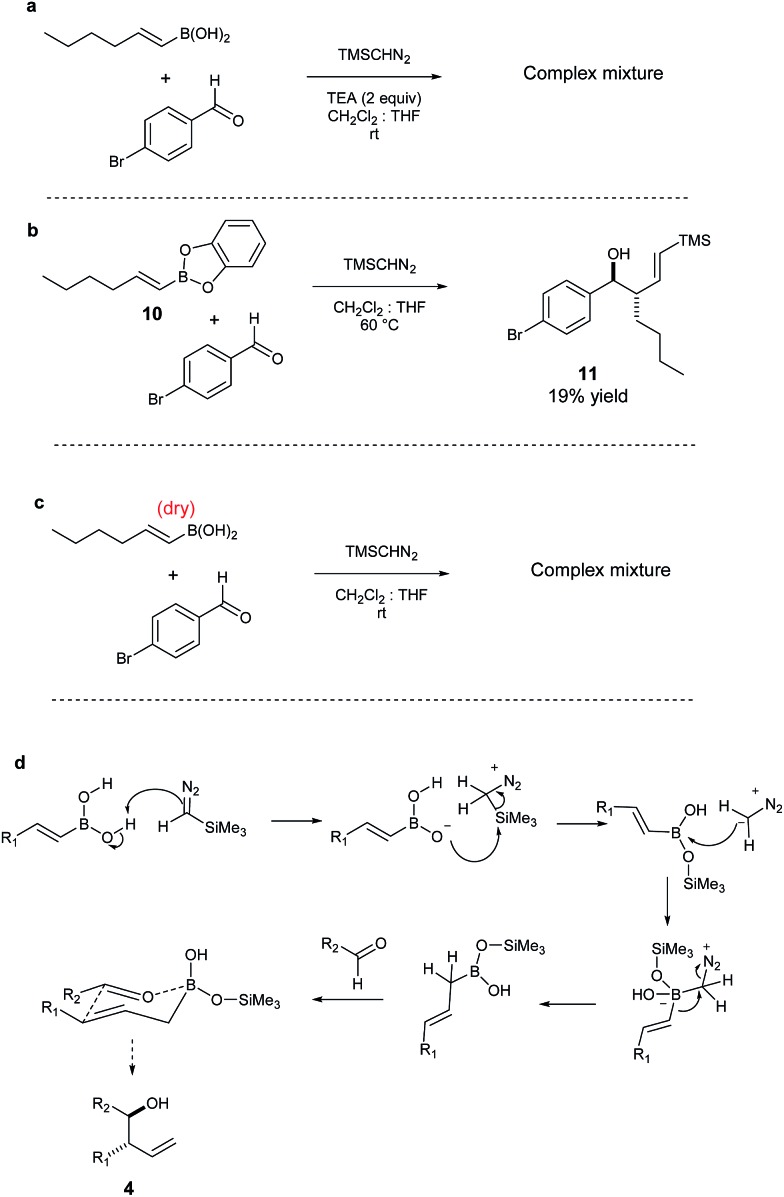
(a) Reaction run in the presence of a base; (b) reaction run with vinyl boronic catechol ester; (c) reaction run with “dry” boronic acid; (d) proposed reaction mechanism.

From a mechanistic viewpoint, we speculate that the absence of the trimethylsilyl group in the product may be due to the instability of TMSCHN_2_, in the presence of *E*-vinyl boronic acids.^56–58^ Efforts to elucidate the mechanism confirmed that the addition of a base (*e.g.* TEA) shuts down the reaction. Similarly, the treatment of the vinyl boronic acid solution with a drying reagent (*e.g.* molecular sieves) followed by addition of aldehyde and TMSCHN_2_, afforded a very complex mixture and only traces of product. This suggests that the acidity of the vinyl boronic acid is important in respect to the desired reaction outcome.^59^ Another piece of information showed that when catechol borane **10** was employed as the vinyl boronic ester partner under the same reaction conditions, vinyl derivative **11** was obtained (19% yield) along with recovered starting material. Additionally, ^1^H-NMR studies indicate that after addition of TMSCHN_2_ to the boronic acid partner, the main intermediate in the reaction mixture is the corresponding allylic boronic acid, in which no silylated intermediate is observed.^60^ We therefore propose a mechanism where TMSCHN_2_ is first protonated by the boronic acid; the boronate intermediate then reacts with the trimethylsilane group in order to generate a highly reactive diazomethane partner; this diazomethane reacts with the vinyl boronic acid to provide the corresponding allylic boronic acid (Scheme 3). This intermediate then reacts with the aldehyde electrophile to yield the final anti product after deborylation.

## Conclusions

In conclusion, we have developed a robust and scalable metal-free multicomponent approach towards the synthesis of homoallylic alcohols, using TMSCHN_2_, *E*-vinyl boronic acids and aldehydes. The method was first developed using a flow-based route and subsequently transferred to a batch procedure in order to demonstrate the generality of the chemistry. The power of this method was further exemplified in an iterative fashion to build novel and potentially useful oligomeric materials. Current investigations are directed towards the development of an asymmetric version of this method.
