# Supplementary material for: A multicomponent approach for the preparation of homoallylic alcohols
Source: Chem Sci. 2016 Jul 8;7(11):6803–7. doi: 10.1039/c6sc02581a (PMC5134730; doi:10.1039/c6sc02581a)
Supplement: Supplementary file 1 [file SC-007-C6SC02581A-s001.pdf]

Electronic Supplementary Material (ESI) for Chemical Science.
This journal is © The Royal Society of Chemistry 2016

Supporting Information

A multicomponent approach for the preparation of homoallylic alcohols

Jian-Siang Poh,‡ [a] Shing-Hing Lau,‡ [a] Iain G. Dykes,[a] Duc. N. Tran,[a] Claudio Battilocchio[a] and Steven V. Ley*[a]

[a] Innovative Technology Centre, Department of Chemistry, University of Cambridge, Lensfield Road, CB2 1EW, Cambridge, UK

Contents

1. General experimental details 3
2. Synthetic procedures and characterisation for homoallylic alcohols 4
   2.1. Homoallylic alcohol synthesis in flow 4
   2.2. Homoallylic alcohol synthesis in batch 12
   2.3. Iterative polyol sequence 23
3. COSY ^{1}H-NMR studies 30
4. ^{1}H and ^{13}C NMR spectra of compound **11** 31
5. ^{1}H and ^{13}C-NMR spectra 32
References 93

1. General experimental details

All batch reactions were performed using oven-dried glassware (200 °C) under an atmosphere of argon unless otherwise stated. All flow reactions were performed using a Uniqsis FlowSyn platform[1] or a Vapourtec R2+R4 system.[2] Solvents were freshly distilled over sodium benzophenone ketyl (THF) or calcium hydride (CH_2Cl_2, hexane and EtOAc). All reagents were obtained from commercial sources and used without further purification.

Flash column chromatography was performed using high-purity grade silica gel (Merck grade 9385) with a pore size 60 Å and 230–400 mesh particle size under air pressure. Analytical thin layer chromatography (TLC) was performed using silica gel 60 F_{254} pre-coated glass backed plates and visualized by ultraviolet radiation (254 nm) and/or potassium permanganate solution as appropriate.

^{1}H NMR spectra were recorded on a 600 MHz Avance 600 BBI Spectrometer as indicated. Chemical shifts are reported in ppm with the resonance resulting from incomplete deuteration of the solvent as the internal standard ($CDCl_3$: 7.26 ppm). ^{13}C NMR spectra were recorded the same spectrometer with complete proton decoupling. Chemical shifts are reported in ppm with the solvent resonance as the internal standard ($^{13}CDCl_3$: 77.16 ppm, t). ^{19}F NMR spectra were recorded on a 376 MHz Avance III HD Spectrometer. Chemical shifts are reported in ppm with $CFCl_3$ as the external standard ($CFCl_3$: 0.00 ppm). Data are reported as follows: chemical shift δ/ppm, integration (^{1}H only), multiplicity (s = singlet, d = doublet, t = triplet, q = quartet, qn = quintet, br = broad, m = multiplet or combinations thereof; ^{13}C signals are singlets unless otherwise stated), coupling constants *J* in Hz, assignment. Spectra are assigned as fully as possible, using ^{1}H-COSY, DEPT-135, HMQC and HMBC where appropriate to facilitate structural determination. Signals that cannot be unambiguously assigned are reported with all possible assignments separated by a slash (e.g. H1/H2) or descriptions of their environments (e.g. ArH, OH). Multiple signals arising from diastereotopic or (pseudo)axial/equatorial positions are suffixed alphabetically (e.g. $H1_a$, $H1_b$). Overlapping signals that cannot be resolved are reported with their assignments denoted in list format (e.g. H1, H2 and H3). ^{1}H NMR signals are reported to 2 decimal places and ^{13}C signals to 1 decimal place unless rounding would produce a value identical to another signal. In this case, an additional decimal place is reported for both signals concerned.

Infrared spectra were recorded neat as thin films on a Perkin-Elmer Spectrum One FTIR spectrometer and selected peaks are reported (s = strong, m = medium, w = weak, br = broad).

High resolution mass spectrometry (HRMS) was performed using positive electrospray ionisation (ESI+), on either a Waters Micromass LCT Premier spectrometer or performed by the Mass Spectrometry Service for the Chemistry Department at the University of Cambridge. All *m/z* values are reported to 4 decimal places and are within ± 5 ppm of theoretical values.

2. Synthetic procedures and characterisation for homoallylic alcohols

2.1. Homoallylic alcohol synthesis in flow

General flow procedure for homoallylic alcohol synthesis: A solution of the vinyl boronic acid **2** (0.04 M, in CH_2Cl_2 : THF, 4:1 v/v) and a solution of $TMSCHN_2$ (**1**) and aldehyde **3** (0.066 M and 0.033 M, respectively, in CH_2Cl_2) were pumped (flow rate 0.4 mL min^{-1} each channel) and combined at a T-piece before reacting in a 20 mL perfluoroalkoxy alkanes (PFA) reactor coil (o.d. 1/16″, τ = 25 min), at 60 °C. The exiting stream of borylated intermediate was then collected in a flask containing PS-tosyl hydrazine and MeOH (2 mL), and stirred overnight. The solvent was removed under reduced and the residue passed through a plug of silica gel to provide the desired homoallylic alcohol **4**.

1-(4-bromophenyl)-2-(*p*-tolyl)but-3-en-1-ol (4a):

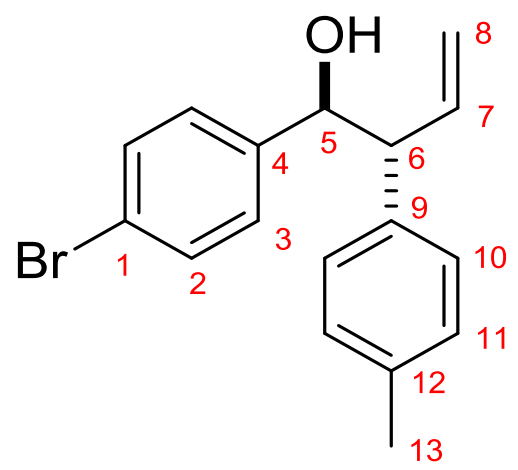

Isolated as a colourless oil (82.6 mg, 0.260 mmol, 91%) after silica gel column chromatography (eluent: 10% → 15% EtOAc/hexane), following the general batch procedure for homoallylic alcohol synthesis.

^{1}H NMR (600 MHz, $CDCl_3$): δ 7.33 (d, *J* = 8.4 Hz, 2 H, H2), 7.04 (d, *J* = 7.9 Hz, 2 H, H11), 7.02 (d, *J* = 8.4 Hz, 2 H, H3), 6.93 (d, *J* = 7.9 Hz, 2 H, H10), 6.20 (ddd, *J* = 17.3, 10.2, 8.4 Hz, 1 H, H7), 5.26 (d, *J* = 10.2 Hz, 1 H, $H8_{cis}$), 5.22 (d, *J* = 17.1 Hz, 1 H, $H8_{trans}$), 4.78 (dd, *J* = 7.8, 2.0 Hz, 1 H, H5), 3.45 (t, *J* = 8.4 Hz, 1 H, H6), 2.37 (d, *J* = 2.0 Hz, 1 H, OH), 2.29 (s, 3 H, H13).
^{13}C NMR (150 MHz, $CDCl_3$): δ 141.0 (C1), 137.8 (C7), 137.2 (C9), 136.5 (C12), 131.1 (C2), 129.3 (C11), 128.6 (C3), 128.2 (C10), 121.3 (C4), 118.7 (C8), 76.7 (C5), 59.0 (C6), 21.1 (C13).
FTIR (ν_{max}, cm^{-1}): 3440 (br w, OH), 3023 (w), 2919 (w), 1637 (w, C=C), 1593 (w), 1513 (m), 1487 (m), 1407 (w), 1307 (w), 1188 (w), 1104 (w), 1070 (m), 1041 (m), 1010 (s), 920 (m), 819 (s), 777 (w).
HRMS (ESI): calculated for $C_{17}H_{18}BrO$ $[M+H]^+$ 317.0536, found 317.0538.
R_f = 0.30 (15% EtOAc/hexane).

1-(4-bromophenyl)-2-(4-fluorophenyl)but-3-en-1-ol (4b):

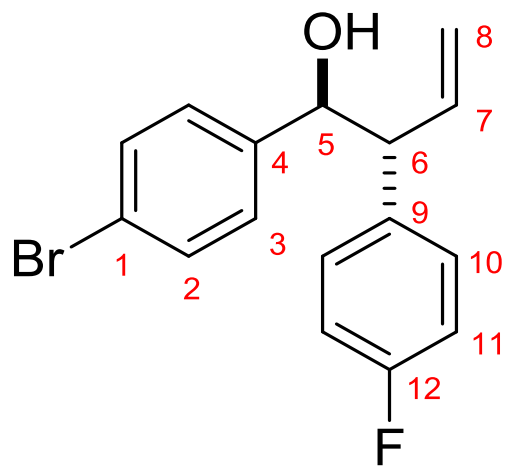

Isolated as a colourless oil (67.1 mg, 0.209 mmol, 73%) after silica gel column chromatography (eluent: 10% → 15% EtOAc/hexane), following the general flow procedure for homoallylic alcohol synthesis. Collection at steady state over 340 min for scale up provided 1.14 g of material (73%).

^{1}H NMR (600 MHz, $CDCl_3$): δ 7.33 (d, *J* = 8.4 Hz, 2 H, H2), 6.98 (m, 4 H, H3 and H10), 6.91 (t, *J* = 8.7 Hz, 2 H, H11), 6.18 (ddd, *J* = 17.1, 10.2, 8.1, 1 H, H7), 5.29 (dd, *J* = 10.2, 0.5 Hz, 1 H, $H8_{cis}$), 5.23 (d, *J* = 17.1 Hz, 1 H, $H8_{trans}$), 4.74 (dd, *J* = 8.1, 2.0 Hz, 1 H, H5), 3.47 (t, *J* = 8.1 Hz, 1 H, H6), 2.38 (d, *J* = 2.0 Hz, 1 H, OH).
^{13}C NMR (150 MHz, $CDCl_3$): δ 161.7 (d, *J* = 245.3 Hz, C12), 140.8 (C1), 137.5 (C7), 136.0 (d, *J* = 3.2 Hz, C9), 131.2 (C2), 129.9 (d, *J* = 7.9 Hz, C10), 128.5 (C3), 121.5 (C4), 119.0 (C8), 115.4 (d, *J* = 21.2 Hz, C11), 76.8 (d, *J* = 1.1 Hz, C5), 58.5 (C6).
^{19}F NMR (376 MHz, $CDCl_3$): δ -115.8 (s, 1 F, F12).
FTIR (ν_{max}, cm^{-1}): 3420 (w, OH), 3076 (w), 2890 (w), 1637 (w, C=C), 1603 (w), 1508 (s), 1488 (m), 1407 (w), 1306 (w), 1222 (s), 1160 (m), 1095 (w), 1070 (m), 1042 (w), 1010 (s), 923 (w), 860 (w), 824 (s).
HRMS (ESI): calculated for $C_{16}H_{13}BrF$ $[M+H-H_2O]^+$ 303.0179, found 303.0193.
R_f = 0.17 (10% EtOAc/hexane).

1-(4-bromophenyl)-2-(4-(trifluoromethyl)phenyl)but-3-en-1-ol (4c):

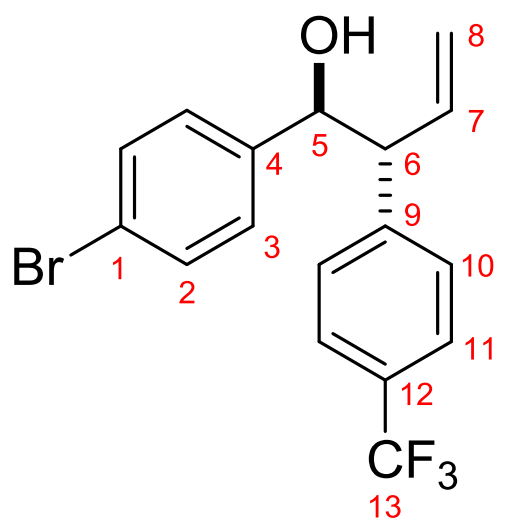

Isolated as a colourless oil (75.6 mg, 0.204 mmol, 71%) after silica gel column chromatography (eluent: 15% EtOAc/hexane), following the general flow procedure for homoallylic alcohol synthesis.

^{1}H NMR (600 MHz, $CDCl_3$): δ 7.48 (d, *J* = 8.1 Hz, 2 H, H11), 7.35 (d, *J* = 8.4 Hz, 2 H, H2), 7.17 (d, *J* = 8.1 Hz, 2 H, H10), 7.01 (d, *J* = 8.4 Hz, 2 H, H3), 6.20 (ddd, *J* = 17.1, 10.2, 9.0 Hz, 1 H, H7), 5.31 (d, *J* = 10.2 Hz, 1 H, $H8_{cis}$), 5.23 (d, *J* = 17.1 Hz, 1 H, $H8_{trans}$), 4.82 (d, *J* = 7.5 Hz, 1 H, H5), 3.59 – 3.54 (m, 1 H, H6), 2.32 (br d, *J* = 1.6 Hz, 1 H, OH).
^{13}C NMR (150 MHz, $CDCl_3$): δ 144.5 (q, *J* = 0.9 Hz, C9), 140.6 (C1), 136.7 (C7), 131.4 (C2), 129.2 (q, *J* = 32.4 Hz, C12), 128.8 (C10), 128.4 (C3), 125.5 (q, *J* = 3.8 Hz, C11), 124.2 (q, *J* = 272.0 Hz, C13), 121.7 (C4), 119.7 (C8), 76.6 (C5), 59.0 (C6).
^{19}F NMR (376 MHz, $CDCl_3$): δ -62.5 (s, 3 F, F13).
FTIR (ν_{max}, cm^{-1}): 3420 (br w, OH), 2922 (w), 1618 (w, C=C), 1592 (w), 1487 (w), 1413 (w), 1384 (w), 1324 (s), 1163 (m), 1122 (s), 1068 (s), 1010 (m), 925 (w), 821 (w), 767 (w).

HRMS (ESI): calculated for $C_{17}H_{13}BrF_3$ $[M+H-H_2O]^+$ 353.0147, found 353.0150.
$\boldsymbol{R_f}$ = 0.22 (15% EtOAc/hexane).

1-(pyridin-3-yl)-2-(4-(trifluoromethyl)phenyl)but-3-en-1-ol (4d):

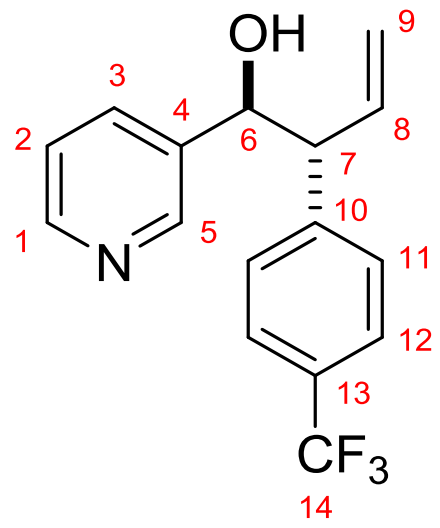

Isolated as a brown oil (74.5 mg, 0.254 mmol, 89%) after silica gel column chromatography (eluent: 80% EtOAc/hexane), following the general flow procedure for homoallylic alcohol synthesis.

^{1}H NMR (600 MHz, $CDCl_3$): δ 8.31 (dd, J = 4.8, 1.3 Hz, 1 H, H1), 8.25 (d, J = 1.7 Hz, 1 H, H5), 7.50 – 7.45 (m, 3 H, H3 and H12), 7.18 (d, J = 8.1 Hz, 2 H, H11), 7.13 (dd, J = 7.8, 4.8 Hz, 1 H, H2), 6.21 (ddd, J = 17.1, 10.3, 8.8 Hz, 1 H, H8), 5.29 (d, J = 10.3 Hz, 1 H, $H9_{cis}$), 5.18 (d, J = 17.1 Hz, 1 H, $H9_{trans}$), 4.88 (d, J = 7.3 Hz, 1 H, H6), 3.65 (br s, 1 H, OH), 3.61 – 3.56 (m, 1 H, H7).
^{13}C NMR (150 MHz, $CDCl_3$): δ 148.7 (C1), 148.1 (C5), 144.4 (q, J = 1.0 Hz, C10), 137.5 (C4), 136.3 (C8), 134.4 (C3), 131.0, 129.1 (q, J = 32.4 Hz, C13), 128.7 (C11), 125.4 (q, J = 3.7 Hz, C12), 124.0 (d, J = 272.0 Hz, C14), 123.1 (C2), 119.5 (C9), 74.7 (C6), 58.6 (C7).
^{19}F NMR (376 MHz, $CDCl_3$): δ -62.5 (s, 3 F, F14).
FTIR (ν_{max}, cm^{-1}): 3164 (br w, OH), 1617 (w, C=C), 1581 (w), 1509 (w), 1426 (w), 1325 (s), 1164 (w), 1122 (m), 1068 (m), 1018 (w), 924 (w), 831 (w).
HRMS (ESI): calculated for $C_{16}H_{15}F_3NO$ $[M+H]^+$ 294.1100, found 294.1104.
$\boldsymbol{R_f}$ = 0.30 (80% EtOAc/hexane).

2-(4-fluorophenyl)-1-(pyridin-3-yl)but-3-en-1-ol (4e):

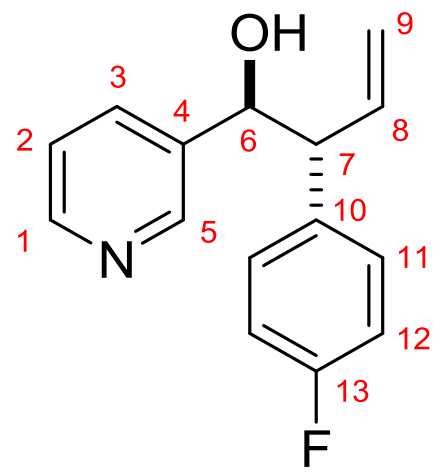

Isolated as a brown solid (39.0 mg, 0.160 mmol, 56%) after silica gel column chromatography (eluent: 80% EtOAc/hexane → EtOAc), following the general flow procedure for homoallylic alcohol synthesis.

^{1}H NMR (600 MHz, $CDCl_3$): δ 8.34 (dd, J = 4.8, 1.8 Hz, 1 H, H1), 8.24 (d, J = 1.8 Hz, 1 H, H5), 7.47 (dt, J = 7.8, 1.8 Hz, 1 H, H3), 7.13 (dd, J = 7.8, 4.8 Hz, 1 H, H2), 6.99 (dd, J = 8.6, 5.4 Hz, 2 H, H11), 6.90 (t, J = 8.6 Hz, 2 H, H12), 6.20 (ddd, J = 17.1, 10.2, 8.3 Hz, 1 H, H8), 5.29 (dd, J = 10.2, 0.7 Hz, 1 H, $H9_{cis}$), 5.21 (d, J = 17.1 Hz, 1 H, $H9_{trans}$), 4.81 (d, J = 8.3 Hz, 1 H, H6), 3.50 (t, J = 8.3 Hz, 1 H, H7), 3.31 (br s, 1 H, OH).
^{13}C NMR (150 MHz, $CDCl_3$): δ 161.8 (d, J = 245.6 Hz, C13), 148.7 (C1), 148.4 (C5), 137.6 (C4), 137.2 (C8), 135.9 (d, J = 3.3 Hz, C10), 134.5 (C3), 129.9 (d, J = 7.9 Hz, C11), 123.1 (C2), 119.1 (C9), 115.5 (d, J = 21.2 Hz, C12), 75.2 (C6), 58.4 (C7).
^{19}F NMR (376 MHz, $CDCl_3$): δ -115.6 (s, 1 F, F13).

FTIR (ν_{max}, cm^{-1}): 3176 (br w, OH), 1638 (w, C=C), 1601 (w), 1507 (s), 1427 (w), 1222 (m), 1160 (w), 1043 (w), 1029 (w), 922 (w), 829 (w).
HRMS (ESI): calculated for $C_{15}H_{15}FNO$ $[M+H]^+$ 244.1132, found 244.1129.
***R**f* = 0.17 (80% EtOAc/hexane).

(*E*)-3-(4-(trifluoromethyl)phenyl)hepta-1,5-dien-4-ol (4f):

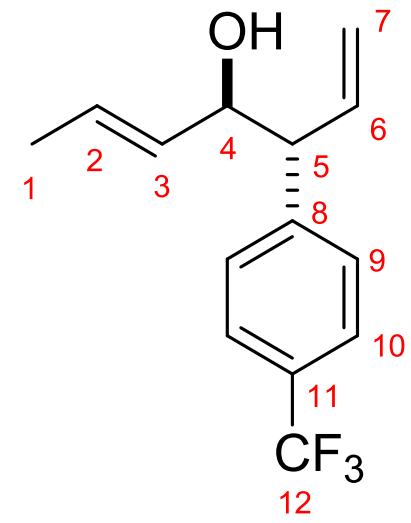

Isolated as a yellow oil (52.8 mg, 0.206 mmol, 72%) after silica gel column chromatography (eluent: 15% EtOAc/hexane), following the general flow procedure for homoallylic alcohol synthesis.

^{1}H NMR (600 MHz, $CDCl_3$): δ 7.56 (d, *J* = 8.2 Hz, 2 H, H10), 7.33 (d, *J* = 8.2 Hz, 2 H, H9), 6.13 (ddd, *J* = 17.1, 10.2, 8.7 Hz, 1 H, H6), 5.60 (dqd, *J* = 15.3, 6.5, 0.9 Hz, 1 H, H2), 5.37 (ddq, *J* = 15.3, 7.1, 1.5 Hz, 1 H, H3), 5.26 (d, *J* = 10.3 Hz, 1 H, $H7_{cis}$), 5.19 (d, *J* = 17.1 Hz, 1 H, $H7_{trans}$), 4.31 – 4.27 (m, 1 H, H4), 3.44 – 3.40 (m, 1 H, H5), 1.86 (br s, 1 H, OH), 1.61 (ddd, *J* = 6.5, 1.5, 0.5 Hz, 3 H, H1).
^{13}C NMR (150 MHz, $CDCl_3$): δ 145.4 (q, *J* = 1.1 Hz, C8), 137.3 (C6), 131.1 (C3), 129.0 (q, *J* = 32.4 Hz, C11), 129.0 (C9), 128.9 (C2), 125.4 (q, *J* = 3.8 Hz, C10), 124.4 (q, *J* = 271.8 Hz, C12), 118.6 (C7), 75.3 (C4), 57.0 (C5), 17.8 (C1).
^{19}F NMR (376 MHz, $CDCl_3$): δ -62.4 (s, 3 F, F12).
FTIR (ν_{max}, cm^{-1}): 3420 (br w, OH), 2919 (w), 1618 (w, C=C), 1326 (s), 1164 (m), 1124 (m), 1069 (m), 1019 (w), 966 (w), 923 (w), 834 (w).
HRMS (ESI): calculated for $C_{14}H_{14}F_3O$ $[M+H-H_2]^+$ 255.0991, found 255.1004.
***R**f* = 0.22 (15% EtOAc/hexane).

(*E*)-3-(4-methoxyphenyl)hepta-1,5-dien-4-ol (4g):

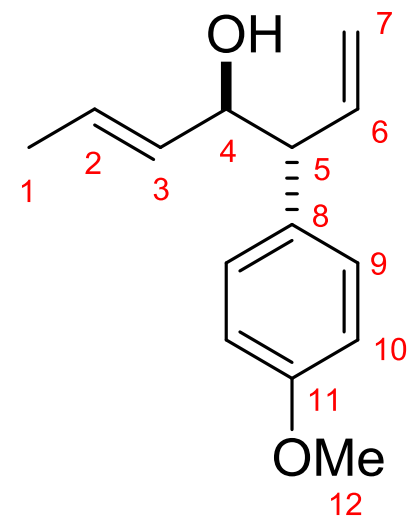

Isolated as a yellow oil (52.5 mg, 0.240 mmol, 84%) after silica gel column chromatography (eluent: 15% EtOAc/hexane), following the general flow procedure for homoallylic alcohol synthesis.

^{1}H NMR (600 MHz, $CDCl_3$): δ 7.12 (d, *J* = 8.7 Hz, 2 H, H9), 6.85 (d, *J* = 8.7 Hz, 2 H, H10), 6.11 (ddd, *J* = 17.1, 10.2, 8.8 Hz, 1 H, H6), 5.59 (dqd, *J* = 15.3, 6.5, 0.9 Hz, 1 H, H2), 5.39 (ddq, *J* = 15.3, 6.8, 1.6 Hz, 1 H, H3), 5.21 (dd, *J* = 10.2, 1.1 Hz, 1 H, $H7_{cis}$), 5.17 (d, *J* = 17.1 Hz, 1 H, $H7_{trans}$), 4.25 – 4.21 (m, 1 H, H4), 3.79 (s, 3 H, H12), 3.32 – 3.28 (m, 1 H, H5), 1.86 (br s, 1 H, OH), 1.61 (ddd, *J* = 6.5, 1.6, 0.9 Hz, 3 H, H1).

^{13}C NMR (150 MHz, $CDCl_3$): δ 158.4 (C11), 138.5 (C6), 133.1 (C8), 131.5 (C3), 129.5 (C9), 128.1 (C2), 117.6 (C7), 114.0 (C10), 75.4 (C4), 56.5 (C5), 55.3 (C12), 17.9 (C1).
FTIR (ν_{max}, cm^{-1}): 3425 (br w, OH), 2937 (w), 1611 (w, C=C), 1512 (s), 1465 (w), 1303 (w), 1247 (s), 1179 (m), 1036 (m), 966 (w), 919 (w), 828 (w).
HRMS (ESI): calculated for $C_{14}H_{17}O$ $[M+H-H_2O]^+$ 201.1274, found 201.1280.
R R_f = 0.32 (15% EtOAc/hexane).

2-(4-fluorophenyl)-1-(furan-2-yl)but-3-en-1-ol (4h):

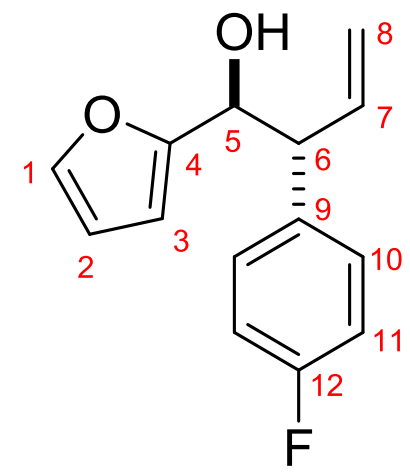

Isolated as a yellow oil (55.9 mg, 0.241 mmol, 84%) after silica gel column chromatography (eluent: 15% EtOAc/hexane), following the general flow procedure for homoallylic alcohol synthesis.

^{1}H NMR (600 MHz, $CDCl_3$): δ 7.31 (dd, *J* = 1.8, 0.7 Hz, 1 H, H1), 7.09 (dd, *J* = 8.7, 5.4 Hz, 2 H, H10), 6.92 (t, *J* = 8.7 Hz, 2 H, H11), 6.21 (dd, *J* = 3.2, 1.8 Hz, 1 H, H2), 6.18 (ddd, *J* = 17.1, 10.3, 8.2 Hz, 1 H, H7), 6.05 (d, *J* = 3.2 Hz, 1 H, H3), 5.28 (d, *J* = 10.3 Hz, 1 H, $H8_{cis}$), 5.23 (d, *J* = 17.1 Hz, 1 H, $H8_{trans}$), 4.84 (d, *J* = 8.2 Hz, 1 H, H5), 3.83 (t, *J* = 8.2 Hz, 1 H, H6), 2.27 (br s, 1 H, OH).
^{13}C NMR (150 MHz, $CDCl_3$): δ 161.8 (d, *J* = 245.0 Hz, C12), 154.1 (C4), 142.0 (C1), 137.6 (C7), 136.2 (d, *J* = 3.3 Hz, C9), 129.7 (d, *J* = 7.9 Hz, C10), 118.7 (C8), 115.4 (d, *J* = 21.2 Hz, C11), 110.2 (C2), 107.8 (C3), 71.1 (C5), 55.1 (C6).
^{19}F NMR (376 MHz, $CDCl_3$): δ -116.1 (s, 1 F, F12).
FTIR (ν_{max}, cm^{-1}): 3435 (br w, OH), 1603 (w, C=C), 1509 (s), 1412 (w), 1223 (m), 1160 (w), 1149 (w), 1011 (w), 920 (w), 884 (w), 859 (w), 832 (w).
HRMS (ESI): calculated for $C_{14}H_{12}FO_2$ $[M+H-H_2]^+$ 231.0816, found 231.0822.
R_f = 0.24 (15% EtOAc/hexane).

2-([1,1'-biphenyl]-4-yl)-1-(2-methoxyphenyl)but-3-en-1-ol (4i):

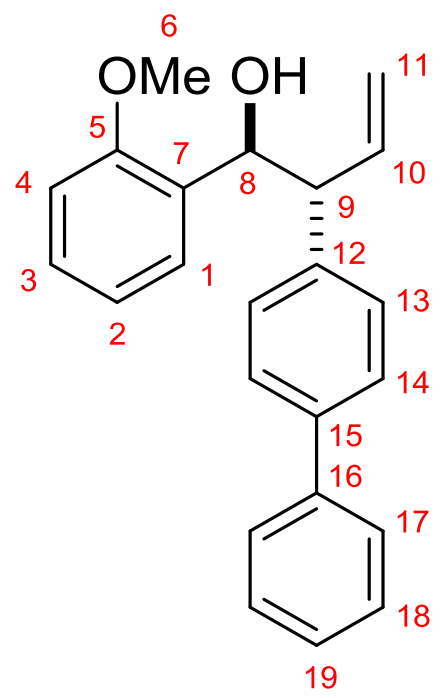

Isolated as a colourless oil (73.4 mg, 0.222 mmol, 78%) after silica gel column chromatography (eluent: 15% EtOAc/hexane), following a modified general flow procedure for homoallylic alcohol synthesis; the boronic acid was dissolved in CH_2Cl_2/THF (2:1) instead.

^{1}H NMR (600 MHz, $CDCl_3$): δ 7.55 (dd, *J* = 8.2, 1.0 Hz, 2 H, H17), 7.46 (d, *J* = 8.2 Hz, 2 H, H13), 7.42 (t, *J* = 7.7 Hz, 2 H, H18), 7.33 (t, *J* = 7.4 Hz, 1 H, H19), 7.25 – 7.21 (m, 3 H, H1

and H14), 7.19 (td, J = 8.1, 1.7 Hz, 1 H, H3), 6.88 (td, J = 8.1, 0.7 Hz, 1 H, H2), 6.79 (d, J = 8.1 Hz, 1 H, H4), 6.34 (ddd, J = 17.2, 10.2, 8.6 Hz, 1 H, H10), 5.23 (dd, J = 10.2, 1.1 Hz, 1 H, $H11_{cis}$), 5.20 (t, J = 6.0 Hz, 1 H, H8), 5.13 (d, J = 17.2 Hz, 1 H, $H11_{trans}$), 3.83 – 3.78 (m, 1 H, H9), 3.74 (s, 3 H, H6), 2.68 (d, J = 6.0 Hz, 1 H, OH).
^{13}C NMR (150 MHz, $CDCl_3$): δ 156.5 (C5), 141.1 (C12/C16), 141.0 (C12/C16), 139.3 (C15), 137.9 (C10), 130.3 (C7), 128.9 (C14/C18), 128.8 (C14/C18), 128.4 (C3), 128.1 (C1), 127.2 (C19), 127.1 (C17), 126.9 (C13), 120.6 (C2), 117.8 (C11), 110.4 (C4), 73.8 (C8), 56.5 (C9), 55.3 (C6).
FTIR (ν_{max}, cm^{-1}): 3526 (br w, OH), 2960 (w), 2837 (w), 1602 (m, C=C), 1488 (w), 1460 (w), 1438 (w), 1239 (w), 1205 (m), 1151 (s), 1067 (m), 1008 (w), 918 (w), 821 (w), 755 (w).
HRMS (ESI): calculated for $C_{23}H_{21}O_2$ $[M+H-H_2]^+$ 329.1536, found 329.1542.
R_f = 0.24 (15% EtOAc/hexane).

4-(3-fluorophenyl)hex-5-en-3-ol (4j):

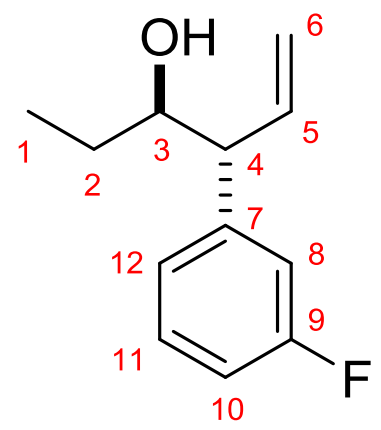

Isolated as a colourless oil (39.0 mg, 0.201 mmol, 70%) after silica gel column chromatography (eluent: 15% EtOAc/hexane), following the general flow procedure for homoallylic alcohol synthesis.

^{1}H NMR (600 MHz, $CDCl_3$): δ 7.30 – 7.26 (m, 1 H, H11), 7.00 (d, J = 7.7 Hz, 1 H, H8), 6.96 – 6.89 (m, 2 H, H10 and H12), 6.09 (ddd, J = 17.1, 10.2, 9.1 Hz, 1 H, H5), 5.25 (dd, J = 10.2, 1.2 Hz, 1 H, $H6_{cis}$), 5.21 (d, J = 17.1 Hz, 1 H, $H6_{trans}$), 3.74 – 3.69 (m, 1 H, H3), 3.29 – 3.25 (m, 1 H, H4), 1.75 (br d, J = 3.2 Hz, 1 H, OH), 1.48 – 1.41 (m, 1 H, $H2_a$), 1.37 – 1.29 (m, 1 H, $H2_b$), 0.94 (t, J = 7.4 Hz, 3 H, H1).
^{13}C NMR (150 MHz, $CDCl_3$): δ 163.1 (d, J = 246.0 Hz, C9), 144.5 (d, J = 6.9 Hz, C7), 137.8 (C5), 130.2 (d, J = 8.3 Hz, C11), 123.8 (d, J = 2.8 Hz, C12), 118.5 (C6), 115.1 (d, J = 21.4 Hz, C8), 113.7 (d, J = 21.0 Hz, C10), 75.3 (C3), 56.7 (d, J = 1.5 Hz, C4), 27.5 (C2), 10.1 (C1).
^{19}F NMR (376 MHz, $CDCl_3$): δ -112.9 (s, 1 F, F9).
FTIR (ν_{max}, cm^{-1}): 3447 (br w, OH), 2937 (m), 1614 (m, C=C), 1588 (s), 1489 (s), 1448 (m), 1261 (m), 1151 (w), 965 (w), 921 (w), 782 (m).
HRMS (ESI): calculated for $C_{12}H_{16}FO$ $[M+H]^+$ 195.1180, found 195.1182.
R_f = 0.27 (15% EtOAc/hexane).

6-methyl-3-phenylhept-1-en-4-ol (4k):

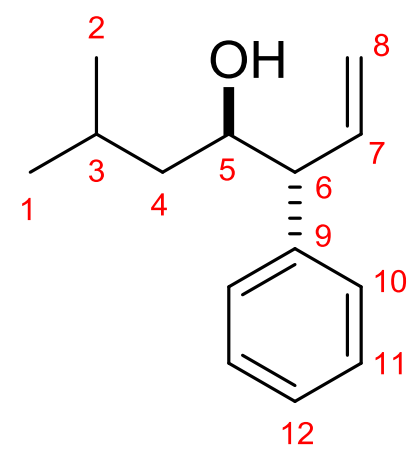

Isolated as a colourless oil (37.8 mg, 0.185 mmol, 65%) after silica gel column chromatography (eluent: 15% EtOAc/hexane), following the general flow procedure for homoallylic alcohol synthesis.

^{1}**H NMR (600 MHz, CDCl$_3$):** δ 7.32 (t, *J* = 7.5 Hz, 2 H, H11), 7.23 (t, *J* = 7.5 Hz, 1 H, H12), 7.20 (d, *J* = 7.5 Hz, 2 H, H10), 6.12 (ddd, *J* = 17.1, 10.1, 9.0 Hz, 1 H, H7), 5.25 – 5.17 (m, 2 H, H8$_{cis}$ and H8$_{trans}$), 3.90 – 3.85 (m, 1 H, H5), 3.21 (dd, *J* = 9.0, 7.3 Hz, 1 H, H6), 1.86 – 1.76 (m, 1 H, H3), 1.74 (dd, *J* = 3.4, 0.8 Hz, 1 H, OH), 1.33 (ddd, *J* = 14.1, 9.8, 4.5 Hz, 1 H, H4$_a$), 1.12 – 1.06 (m, 1 H, H4$_b$), 0.87 (d, *J* = 6.7 Hz, 3 H, H1/H2), 0.84 (d, *J* = 6.6 Hz, 3 H, H1/H2).
^{13}**C NMR (150 MHz, CDCl$_3$):** δ 141.9 (C9), 138.5 (C7), 128.8 (C11), 128.2 (C10), 126.8 (C12), 118.0 (C8), 72.1 (C5), 58.1 (C6), 43.8 (C4), 24.7 (C3), 23.8 (C1/C2), 21.7 (C1/C2).
FTIR (ν_{max}, cm^{-1}): 3436 (br w, OH), 2956 (m), 2929 (w), 2869 (w), 1602 (w, C=C), 1493 (w), 1468 (w), 1453 (w), 1367 (w), 1151 (w), 1063 (w), 918 (w), 843 (w), 759 (w).
HRMS (ESI): calculated for $C_{14}H_{19}$ $[M+H-H_2O]^+$ 187.1481, found 187.1490.
R_f = 0.39 (15% EtOAc/hexane).

(4-fluorophenyl)-6-methylhept-1-en-4-ol (4l):

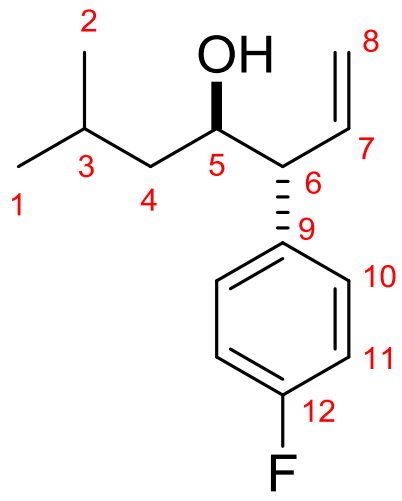

Isolated as a colourless oil (28.1 mg, 0.126 mmol, 44%) after silica gel column chromatography (eluent: 10% EtOAc/hexane), following the general flow procedure for homoallylic alcohol synthesis.

^{1}**H NMR (600 MHz, CDCl$_3$):** δ 7.17 (dd, *J* = 8.7, 5.4 Hz, 2 H, H10), 7.01 (t, *J* = 8.7 Hz, 2 H, H11), 6.08 (ddd, *J* = 17.1, 10.2, 9.1 Hz, 1 H, H7), 5.23 (dd, *J* = 10.2, 1.4 Hz, 1 H, H8$_{cis}$), 5.18 (d, *J* = 17.1 Hz, 1 H, H8$_{trans}$), 3.86 – 3.80 (td, *J* = 7.0, 3.5 Hz, 1 H, H5), 3.20 (dd, *J* = 8.5, 7.4 Hz, 1 H, H6), 1.85 – 1.75 (m, 1 H, H3), 1.72 (br d, *J* = 2.5 Hz, 1 H, OH), 1.31 (ddd, *J* = 14.0, 9.8, 4.5 Hz, 1 H, H4$_a$), 1.07 (ddd, *J* = 14.0, 9.6, 2.9 Hz, 1 H, H4$_b$), 0.87 (d, *J* = 6.7 Hz, 3 H, H1/H2), 0.84 (d, *J* = 6.6 Hz, 3 H, H1/H2).
^{13}**C NMR (150 MHz, CDCl$_3$):** δ 161.7 (d, *J* = 244.8 Hz, C12), 138.3 (C7), 137.6 (d, *J* = 3.2 Hz, C9), 129.6 (d, *J* = 7.8 Hz, C10), 118.2 (C8), 115.6 (d, *J* = 21.1 Hz, C11), 72.1 (C5), 57.1 (C6), 43.9 (C4), 24.7 (C3), 23.8 (C1/C2), 21.7 (C1/C2).
^{19}**F NMR (376 MHz, CDCl$_3$):** δ -116.4 (s, 1 F, F12).
FTIR (ν_{max}, cm^{-1}): 3426 (br w, OH), 2957 (m), 1603 (w, C=C), 1509 (s), 1468 (w), 1368 (w), 1224 (m), 1160 (w), 1063 (w), 920 (w), 831 (w).
HRMS (ESI): calculated for $C_{14}H_{20}FO$ $[M+H]^+$ 223.1493, found 223.1499.
R_f = 0.26 (10% EtOAc/hexane).

(*E*)-3-(4-fluorophenyl)-6,10-dimethylundeca-1,5,9-trien-4-ol (4m-*E*, geranial-derived) and **(*Z*)-3-(4-fluorophenyl)-6,10-dimethylundeca-1,5,9-trien-4-ol (4m-*Z*, neral-derived):**

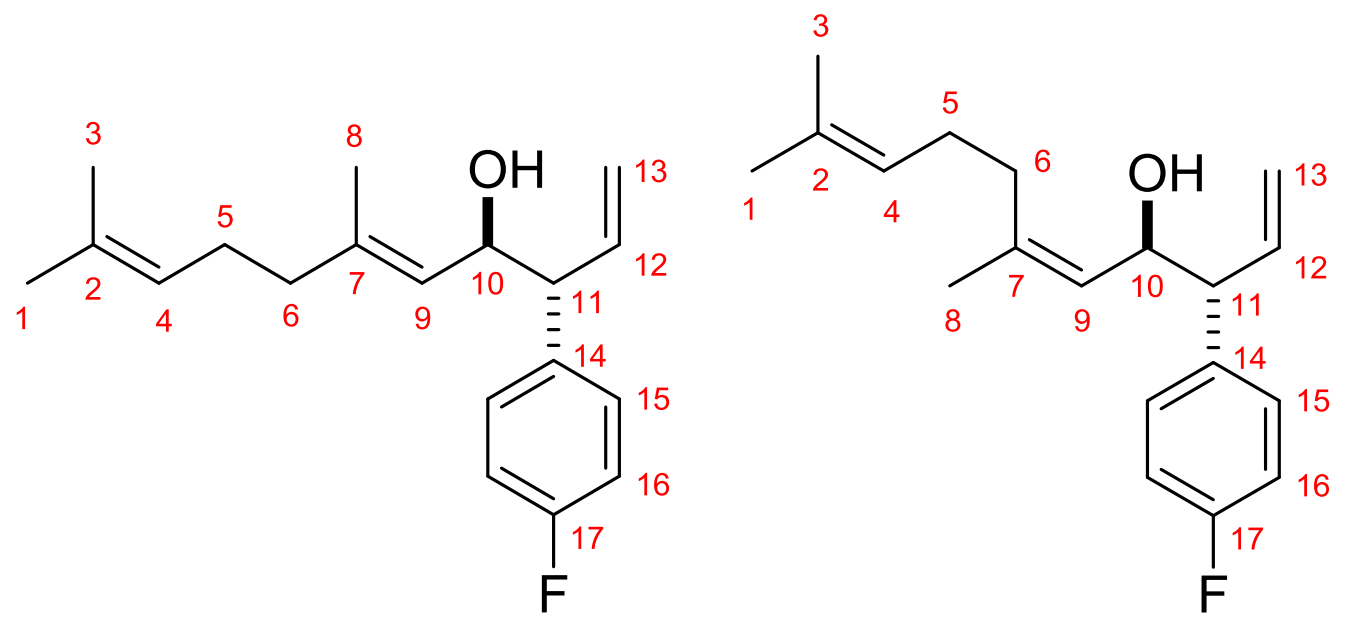

(N.B. Using citral as the aldehyde component, 2:1 mixture of geranial and neral). Isolated as separable *E*/*Z* isomers as colourless oils (*E*-isomer: 53.7 mg, 0.186 mmol; *Z*-isomer: 26.2 mg, 0.091 mmol; combined yield 97%, *E*/*Z* ratio 2:1) after silica gel column chromatography (eluent: 10% EtOAc/hexane), following the general flow procedure for homoallylic alcohol synthesis.

E-isomer:
^{1}H NMR (600 MHz, $CDCl_3$): δ 7.16 (dd, *J* = 8.7, 5.5 Hz, 2 H, H15), 6.97 (t, *J* = 8.7 Hz, 2 H, H16), 6.14 (ddd, *J* = 17.1, 10.2, 8.8 Hz, 1 H, H12), 5.24 (dd, *J* = 10.2, 1.0 Hz, 1 H, $H13_{cis}$), 5.21 (d, *J* = 17.1 Hz, 1 H, $H13_{trans}$), 5.09 (dd, *J* = 8.9, 1.0 Hz, 1 H, H9), 4.99 – 4.94 (m, 1 H, H4), 4.53 – 4.48 (m, 1 H, H10), 3.35 – 3.30 (m, 1 H, H11), 2.05 – 1.88 (m, 4 H, H5 and H6), 1.74 (br s, 1 H, OH), 1.66 (s, 3 H, H1/H3), 1.57 (s, 3 H, H1/H3), 1.47 (d, *J* = 1.0 Hz, 3 H, H8).
^{13}C NMR (150 MHz, $CDCl_3$): δ 161.7 (d, *J* = 244.6 Hz, C17), 140.1 (C7), 138.2 (C12), 136.8 (d, *J* = 3.2 Hz, C14), 131.7 (C2), 130.0 (d, *J* = 7.8 Hz, C15), 125.1 (C9), 124.0 (C4), 118.0 (C13), 115.2 (d, *J* = 21.1 Hz, C16), 71.1 (C10), 56.8 (C11), 39.7 (C6), 26.4 (C5), 25.8 (C1/C3), 17.8 (C1/C3), 16.8 (C8).
^{19}F NMR (376 MHz, $CDCl_3$): δ -116.5 (s, 1 F, F17).
FTIR (ν_{max}, cm^{-1}): 3447 (br w, OH), 2918 (m), 1638 (w), 1603 (w, C=C), 1509 (s), 1448 (w), 1378 (w), 1223 (m), 1159 (w), 1096 (w), 1001 (w), 918 (w), 861 (w), 829 (m).
HRMS (ESI): calculated for $C_{19}H_{24}FO$ $[M+H-H_2]^+$ 287.1806, found 287.1808.
R_f = 0.20 (10% EtOAc/hexane).

Z-isomer:
^{1}H NMR (600 MHz, $CDCl_3$): δ 7.17 (dd, *J* = 8.7, 5.5 Hz, 2 H, H15), 6.97 (t, *J* = 8.7 Hz, 2 H, H16), 6.15 (ddd, *J* = 17.1, 10.3, 8.8 Hz, 1 H, H12), 5.23 (dd, *J* = 10.3, 1.2 Hz, 1 H, $H13_{cis}$), 5.18 (d, *J* = 17.1 Hz, 1 H, $H13_{trans}$), 5.14 (d, *J* = 9.1 Hz, 1 H, H9), 5.05 (t, *J* = 6.8 Hz, 1 H, H4), 4.53 – 4.47 (m, 1 H, H10), 3.35 – 3.29 (m, 1 H, H11), 2.05 – 1.96 (m, 2 H, $H5_a$ and $H6_a$), 1.96 – 1.90 (m, 1 H, $H6_b$), 1.90 – 1.80 (m, 1 H, $H5_b$), 1.69 (s, 3 H, H1/H3), 1.66 (br d, *J* = 1.7 Hz, 1 H, OH), 1.64 (d, *J* = 1.3 Hz, 3 H, H8), 1.58 (s, 3 H, H1/H3).
^{13}C NMR (150 MHz, $CDCl_3$): δ 161.7 (d, *J* = 244.6 Hz, C17), 140.4 (C7), 138.1 (C12), 137.1 (d, *J* = 3.2 Hz, C14), 132.4 (C2), 129.9 (d, *J* = 7.8 Hz, C15), 126.1 (C9), 124.0 (C4), 117.9 (C13), 115.3 (d, *J* = 21.1 Hz, C16), 70.6 (C10), 56.4 (C11), 32.4 (C6), 26.5 (C5), 25.8 (C1/C3), 23.4 (C8), 17.8 (C1/C3).
^{19}F NMR (376 MHz, $CDCl_3$): δ -116.6 (s, 1 F, F17).
FTIR (ν_{max}, cm^{-1}): 3447 (br w, OH), 2967 (w), 2914 (w), 1637 (w), 1603 (w, C=C), 1508 (s), 1448 (w), 1377 (w), 1222 (m), 1159 (w), 1094 (w), 996 (w), 919 (w), 859 (w), 829 (m).
HRMS (ESI): calculated for $C_{19}H_{24}FO$ $[M+H-H_2]^+$ 287.1806, found 287.1809.
R_f = 0.27 (10% EtOAc/hexane).

2.2. Homoallylic alcohol synthesis in batch

General batch procedure for homoallylic alcohol synthesis:

To a solution of aldehyde (0.3 mmol, 1.0 equiv.) and vinylboronic acid (0.36 mmol, 1.2 equiv.) in CH_2Cl_2/THF (4:1, 3 mL) was added (trimethylsilyl)diazomethane (0.3 mL, 2 M in hexanes, 0.6 mmol, 2.0 equiv.) and the mixture subsequently stirred for 2 h. The mixture was quenched with SiO_2 and stirred for a further 10 min, filtered and evaporated under reduced pressure. The residue was purified by silica gel column chromatography to provide the desired homoallylic alcohol.

1-(4-bromophenyl)-2-(*p*-tolyl)but-3-en-1-ol (4a):

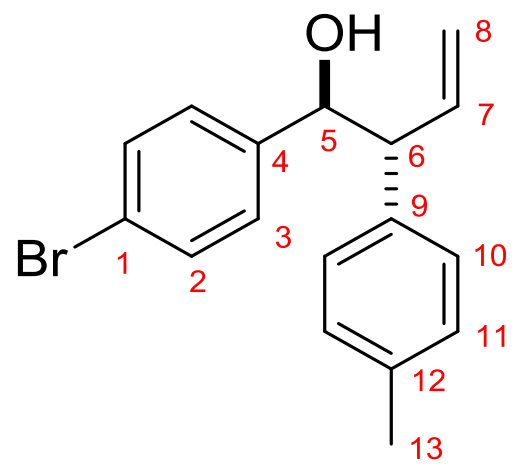

Isolated as a colourless oil (67.8 mg, 0.214 mmol, 71%) after silica gel column chromatography (eluent: 10% → 15% EtOAc/hexane), following the general batch procedure for homoallylic alcohol synthesis. Spectral data consistent with results from flow procedure.

1-(4-bromophenyl)-2-(4-fluorophenyl)but-3-en-1-ol (4b):

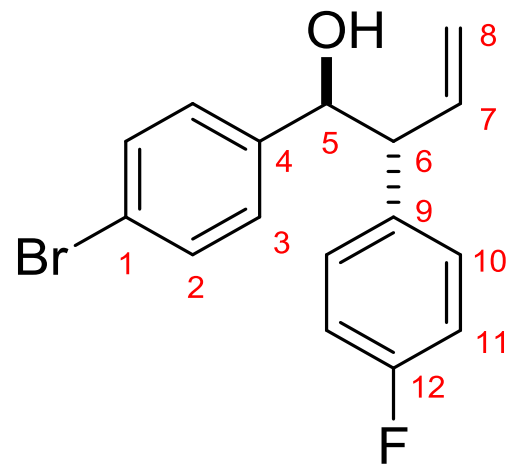

Isolated as a colourless oil (70.3 mg, 0.219 mmol, 73%) after silica gel column chromatography (eluent: 10% → 15% EtOAc/hexane), following the general batch procedure for homoallylic alcohol synthesis. Spectral data consistent with results from flow procedure.

1-(4-bromophenyl)-2-(chloromethyl)but-3-en-1-ol (4n):

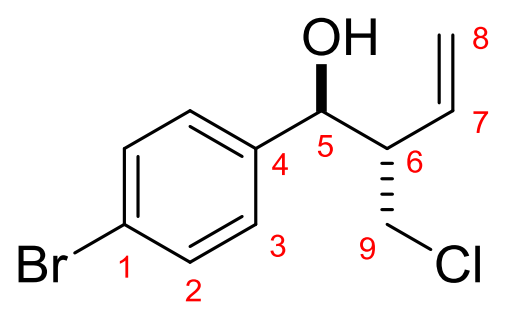

Isolated as a colourless oil (65.2 mg, 0.237 mmol, 79%) after silica gel column chromatography (eluent: 10% EtOAc/hexane), following the general batch procedure for homoallylic alcohol synthesis.

^{1}H NMR (600 MHz, $CDCl_3$): δ 7.47 (d, *J* = 8.3 Hz, 2 H, H2), 7.23 (d, *J* = 8.3 Hz, 2 H, H3), 5.83 (ddd, *J* = 17.3, 10.4, 8.7 Hz, 1 H, H7), 5.28 (dd, *J* = 10.4, 0.5 Hz, 1 H, $H8_{cis}$), 5.16 (dd, *J* = 17.3, 0.5 Hz, 1 H, $H8_{trans}$), 4.87 (d, *J* = 5.9 Hz, 1 H, H5), 3.62 (dd, *J* = 11.0, 6.2 Hz, 1 H, $H9_a$), 3.38 (dd, *J* = 11.0, 5.5 Hz, 1 H, $H9_b$), 2.63 – 2.57 (m, 1 H, H6), 2.19 (br s, 1 H, OH).
^{13}C NMR (150 MHz, $CDCl_3$): δ 140.9 (C1), 134.4 (C7), 131.6 (C2), 128.2 (C3), 121.8 (C4), 120.6 (C8), 72.6 (C5), 53.5 (C6), 45.6 (C9).

FTIR (ν_{max}, cm^{-1}): 3438 (w, OH), 2961 (w), 1676 (w), 1639 (w, C=C), 1591 (w), 1488 (m), 1406 (w), 1293 (w), 1191 (w), 1071 (s), 1010 (s), 925 (m), 866 (w), 825 (s), 776 (w).
HRMS (ESI): calculated for $C_{11}H_{11}BrClO$ $[M+H-H_2]^+$ 272.9676, found 272.9680.
R_f = 0.27 (10% EtOAc/hexane).

1-(4-bromophenyl)-2-vinylhexan-1-ol (4o):

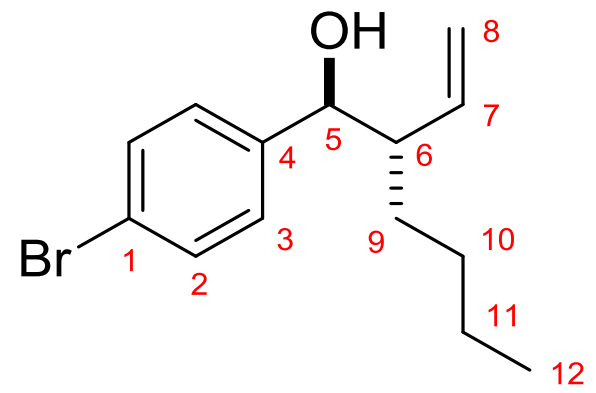

Isolated as a colourless oil (63.5 mg, 0.224 mmol, 75%) after silica gel column chromatography (eluent: 10% EtOAc/hexane), following the general batch procedure for homoallylic alcohol synthesis.

^{1}H NMR (600 MHz, CDCl$_3$): δ 7.46 (d, *J* = 8.3 Hz, 2 H, H2), 7.19 (d, *J* = 8.3 Hz, 2 H, H3), 5.62 (ddd, *J* = 17.2, 10.2, 9.3 Hz, 1 H, H7), 5.25 (dd, *J* = 10.2, 1.8 Hz, 1 H, $H8_{cis}$), 5.17 (ddd, *J* = 17.2, 1.8, 0.6 Hz, 1 H, $H8_{trans}$), 4.35 (dd, *J* = 7.7, 1.2 Hz, 1 H, H5), 2.27 – 2.16 (m, 2 H, H6 and $H9_a$), 1.30 – 1.04 (m, 6 H, $H9_b$, H10, H11 and OH), 0.80 (t, *J* = 7.1 Hz, 3 H, H12).
^{13}C NMR (150 MHz, CDCl$_3$): δ 141.7 (C1), 139.1 (C7), 131.4 (C2), 128.8 (C3), 121.5 (C4), 119.3 (C8), 76.1 (C5), 52.9 (C6), 30.2 (C9), 29.5 (C10), 22.6 (C11), 14.1 (C12).
FTIR (ν_{max}, cm^{-1}): 3430 (w, OH), 2956 (m), 2930 (m), 2858 (m), 1639 (w, C=C), 1593 (w), 1487 (m), 1466 (w), 1405 (w), 1379 (w), 1307 (w), 1190 (w), 1121 (w), 1070 (m), 1041 (m), 1010 (s), 915 (m), 821 (s).
HRMS (ESI): calculated for $C_{14}H_{18}BrO$ $[M+H-H_2]^+$ 281.0536, found 281.0541.
R_f = 0.23 (10% EtOAc/hexane).

2-benzyl-1-(4-bromophenyl)but-3-en-1-ol (4p):

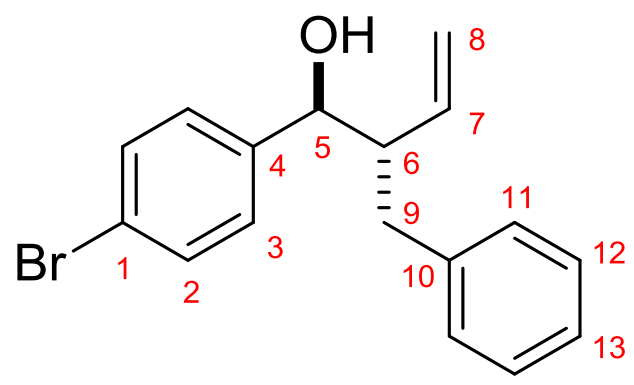

Isolated as a colourless oil (69.7 mg, 0.220 mmol, 73%) after silica gel column chromatography (eluent: 15% EtOAc/hexane), following the general batch procedure for homoallylic alcohol synthesis.

^{1}H NMR (600 MHz, CDCl$_3$): δ 7.48 (d, *J* = 8.4 Hz, 2 H, H2), 7.27 (t, *J* = 7.3 Hz, 2 H, H12), 7.22 – 7.17 (m, 3 H, H3 and H13), 7.10 (d, *J* = 7.3 Hz, 2 H, H11), 5.69 (ddd, *J* = 17.2, 10.3, 8.4 Hz, 1 H, H7), 5.15 (dd, *J* = 10.3, 1.4 Hz, 1 H, $H8_{cis}$), 4.98 (d, *J* = 17.2 Hz, 1 H, $H8_{trans}$), 4.50 (d, *J* = 5.8 Hz, 1 H, H5), 2.72 (dd, *J* = 12.7, 4.9 Hz, 1 H, $H9_a$), 2.64 – 2.53 (m, 2 H, H6 and $H9_b$), 2.27 (br s, 1 H, OH).
^{13}C NMR (150 MHz, CDCl$_3$): δ 141.7 (C1), 139.8 (C10), 137.3 (C7), 131.4 (C2), 129.2 (C11), 128.5 (C3), 128.3 (C12), 126.2 (C13), 121.5 (C4), 119.5 (C8), 74.8 (C5), 53.9 (C6), 37.2 (C9).
FTIR (ν_{max}, cm^{-1}): 3423 (w, OH), 3062 (w), 3027 (w), 2920 (w), 1638 (w, C=C), 1593 (w), 1488 (m), 1455 (w), 1405 (w), 1308 (w), 1229 (w), 1192 (w), 1103 (w), 1071 (m), 1031 (w), 1009 (s), 918 (m), 836 (m), 820 (m), 784 (w).
HRMS (ESI): calculated for $C_{17}H_{16}Br$ $[M+H-H_2O]^+$ 299.0430, found 299.0437.

$\boldsymbol{R_f}$ = 0.43 (15% EtOAc/hexane).

tert-butyl (2-(2-(4-fluorophenyl)-1-hydroxybut-3-en-1-yl)phenyl)carbamate (4q):

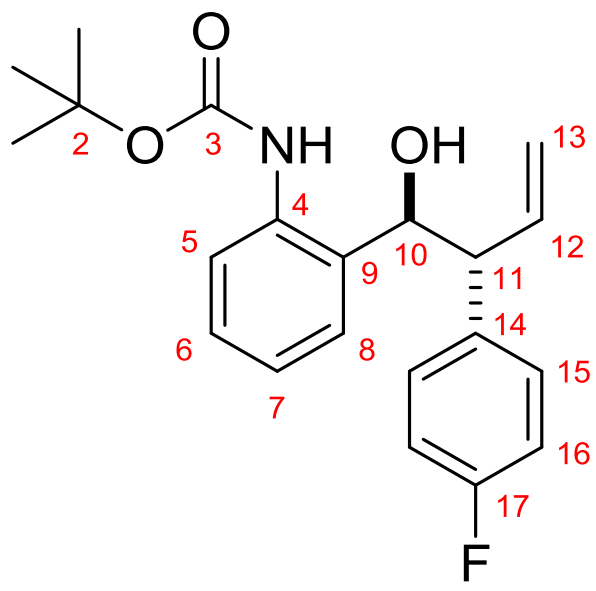

Isolated as a colourless oil (77.2 mg, 0.216 mmol, 72%) after silica gel column chromatography (eluent: 15% EtOAc/hexane), following the general batch procedure for homoallylic alcohol synthesis.

^{1}H NMR (600 MHz, $CDCl_3$): δ 7.87 (br s, 1 H, NH), 7.78 (d, J = 7.5 Hz, 1 H, H8), 7.15 (t, J = 7.5 Hz, 1 H, H7), 6.91 (dd, J = 8.4, 5.5 Hz, 2 H, H15), 6.83 (t, J = 8.6 Hz, 2 H, H16), 6.76 (t, J = 7.5 Hz, 1 H, H6), 6.64 (d, J = 7.5 Hz, 1 H, H5), 6.23 (dt, J = 17.1, 9.6 Hz, 1 H, H12), 5.35 (d, J = 9.6 Hz, 1 H, $H13_{cis}$), 5.31 (d, J = 17.1 Hz, 1 H, $H13_{trans}$), 4.74 (d, J = 9.6 Hz, 1 H, H10), 3.76 (t, J = 9.6 Hz, 1 H, H11), 3.00 (br s, 1 H, OH), 1.54 (s, 9 H, H1).
^{13}C NMR (150 MHz, $CDCl_3$): δ 161.6 (d, J = 245.2 Hz, C17), 153.5 (C3), 137.7 (C12), 137.0 (C4), 135.9 (d, J = 3.2 Hz, C14), 129.6 (d, J = 7.9 Hz, C15), 129.1 (two overlapping signals, C5 and C9), 128.4 (C7), 122.9 (C6), 122.3 (C8), 119.0 (C13), 115.3 (d, J = 21.2 Hz, C16), 80.3 (C2), 78.3 (C10), 55.6 (C11), 28.5 (C1).
^{19}F NMR (376 MHz, $CDCl_3$): δ -115.8 (s, 1 F, F17).
FTIR (ν_{max}, cm^{-1}): 3358 (w, OH), 2980 (w), 1698 (m, C=O), 1637 (w, C=C), 1590 (m), 1508 (s), 1479 (w), 1446 (m), 1393 (w), 1368 (m), 1302 (m), 1226 (s), 1156 (s), 1050 (m), 1024 (m), 908 (m), 860 (w), 830 (m), 755 (m).
HRMS (ESI): calculated for $C_{21}H_{25}FNO_3$ $[M+H]^+$ 358.1813, found 358.1808.
$\boldsymbol{R_f}$ = 0.29 (15% EtOAc/hexane).

1-(5-bromo-2-chlorophenyl)-2-(4-fluorophenyl)but-3-en-1-ol (4r):

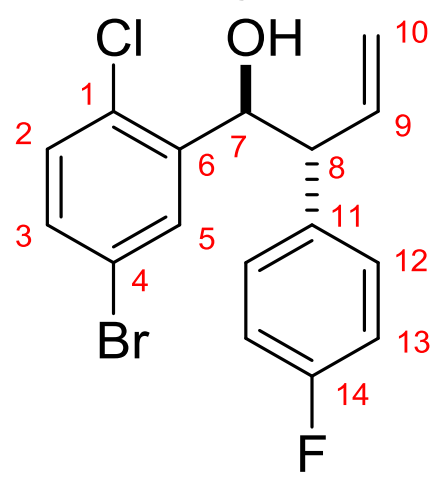

Isolated as a colourless oil (88.0 mg, 0.247 mmol, 82%) after silica gel column chromatography (eluent: 5% EtOAc/hexane), following the general batch procedure for homoallylic alcohol synthesis.

^{1}H NMR (600 MHz, $CDCl_3$): δ 7.68 (d, J = 2.3 Hz, 1 H, H5), 7.30 (dd, J = 8.5, 2.3 Hz, 1 H, H3), 7.24 (dd, J = 8.6, 5.5 Hz, 2 H, H12), 7.13 (d, J = 8.5 Hz, 1 H, H2), 6.98 (t, J = 8.6 Hz, 2 H, H13), 6.21 (ddd, J = 17.1, 10.3, 9.0 Hz, 1 H, H9), 5.27 – 5.24 (m, 1 H, H7), 5.22 (d, J = 10.3 Hz, 1 H, $H10_{cis}$), 5.00 (d, J = 17.1 Hz, 1 H, $H10_{trans}$), 3.69 (dd, J = 9.0, 5.0 Hz, 1 H, H8), 2.25 (d, J = 3.4 Hz, 1 H, OH).

^{13}C NMR (150 MHz, CDCl$_3$): δ 161.9 (d, *J* = 245.3 Hz, C14), 141.7 (C6), 136.6 (d, *J* = 3.2 Hz, C11), 135.3 (C9), 131.7 (C5), 131.5 (C3), 131.0 (C1), 130.7 (C2), 129.8 (d, *J* = 7.9 Hz, C12), 120.7 (C4), 119.5 (C10), 115.4 (d, *J* = 21.2 Hz, C13), 73.4 (C7), 55.1 (C8).
^{19}F NMR (376 MHz, CDCl$_3$): δ -115.8 (s, 1 F, F14).
FTIR (ν_{max}, cm^{-1}): 3455 (w, OH), 1637 (w, C=C), 1603 (w), 1508 (s), 1456 (m), 1390 (w), 1222 (s), 1182 (w), 1159 (m), 1136 (w), 1085 (m), 1042 (s), 1016 (m), 995 (m), 908 (m), 858 (m), 829 (s), 810 (s).
HRMS (ESI): calculated for $C_{16}H_{14}BrClFO$ [M+H]$^+$ 354.9895, found 354.9899.
R$_f$ = 0.16 (5% EtOAc/hexane).

1-(4-(thiophen-2-yl)phenyl)-2-(4-(trifluoromethyl)phenyl)but-3-en-1-ol (4s):

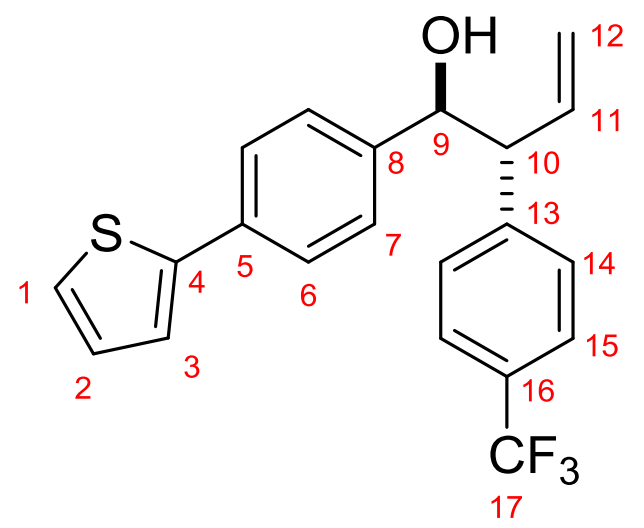

Isolated as a yellow oil (69.6 mg, 0.166 mmol, 62%) after silica gel column chromatography (eluent: 30% Et$_2$O/hexane), following the general batch procedure for homoallylic alcohol synthesis.

^{1}H NMR (600 MHz, CDCl$_3$): δ 7.48 (two superimposed d, *J* = 8.0 Hz, 4 H, H6 and H15), 7.29 (d, *J* = 3.7 Hz, 1 H, H3), 7.26 (d, *J* = 5.0 Hz, 1 H, H1), 7.21 (d, *J* = 8.0 Hz, 2 H, H14), 7.14 (d, *J* = 8.0 Hz, 2 H, H7), 7.07 (dd, *J* = 5.0, 3.7 Hz, 1 H, H2), 6.24 (ddd, *J* = 17.1, 10.2, 8.1 Hz, 1 H, H11), 5.32 (d, *J* = 10.2 Hz, 1 H, H12$_{cis}$), 5.24 (d, *J* = 17.1 Hz, 1 H, H12$_{trans}$), 4.87 (d, *J* = 8.1 Hz, 1 H, H9), 3.64 (t, *J* = 8.1 Hz, 1 H, H10), 2.35 (br s, 1 H, OH).
^{13}C NMR (150 MHz, CDCl$_3$): δ 144.8 (C13), 144.0 (C4), 140.8 (C8), 137.0 (C11), 133.9 (C5), 129.0 (q, *J* = 32.4 Hz, C16), 128.9 (C14), 128.1 (C2), 127.3 (C7), 125.7 (C6), 125.4 (q, *J* = 3.7 Hz, C15), 125.0 (C1), 124.3 (q, *J* = 272.0 Hz, C17), 123.2 (C3), 119.3 (C12), 76.9 (C9), 58.9 (C10).
^{19}F NMR (376 MHz, CDCl$_3$): δ -62.4 (s, 3 F, F17).
FTIR (ν_{max}, cm^{-1}): 3432 (w, OH), 1617 (w, C=C), 1537 (w), 1501 (w), 1414 (w), 1323 (s), 1260 (w), 1162 (m), 1118 (s), 1067 (s), 1017 (m), 959 (w), 924 (w), 818 (s), 767 (w).
HRMS (ESI): calculated for $C_{21}H_{18}F_3OS$ [M+H]$^+$ 375.1025, found 375.1021.
R$_f$ = 0.31 (30% Et$_2$O/hexane).

2-(4-methoxyphenyl)-1-(perfluorophenyl)but-3-en-1-ol (4t):

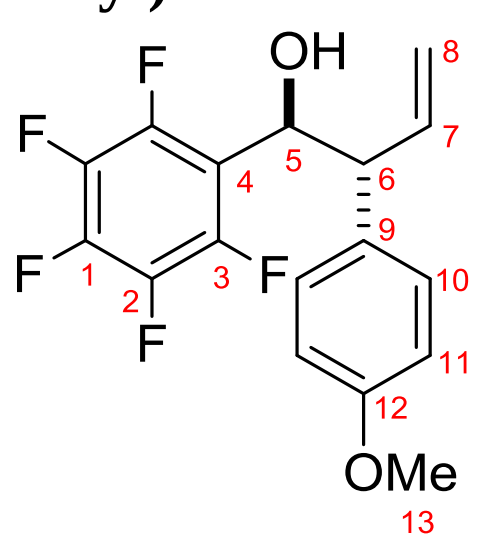

Isolated as a colourless oil (77.5 mg, 0.225 mmol, 75%) after silica gel column chromatography (eluent: 15% EtOAc/hexane), following the general batch procedure for homoallylic alcohol synthesis.

^{1}H NMR (600 MHz, $CDCl_3$): δ 7.00 (d, *J* = 8.6 Hz, 2 H, H10), 6.75 (d, *J* = 8.6 Hz, 2 H, H11), 6.18 (dt, *J* = 17.2, 10.1 Hz, 1 H, H7), 5.33 (d, *J* = 17.2 Hz, 1 H, $H8_{trans}$), 5.31 (d, *J* = 10.1 Hz, 1 H, $H8_{cis}$), 5.18 (dd, *J* = 10.1, 4.7 Hz, 1 H, H5), 3.85 – 3.80 (m, 1 H, H6), 3.74 (s, 3 H, H13), 2.56 (d, *J* = 4.7 Hz, 1 H, OH).
^{13}C NMR (150 MHz, $CDCl_3$): δ 158.8 (C12), 144.9 (approx. d, *J* = 248.4 Hz, C2), 140.6 (dtt, *J* = 253.8, 13.0, 5.2 Hz, C1), 138.3 (C7), 137.4 (approx. d, *J* = 249.9 Hz, C3), 131.3 (C9), 128.6 (C10), 118.8 (C8), 115.3 (approx. t, *J* = 15.2 Hz, C4), 114.3 (C11), 69.1 (C5), 55.6 (C6), 55.3 (C13).
^{19}F NMR (376 MHz, $CDCl_3$): δ -142.2 (dd, *J* = 21.8, 7.6 Hz, 2 F, F3), -154.8 (t, *J* = 20.9 Hz, 1 F, F1), -162.1 – -162.4 (m, 2 F, F2).
FTIR (ν_{max}, cm^{-1}): 3449 (w, OH), 2939 (w), 2841 (w), 1654 (w, C=C), 1611 (w), 1585 (w), 1521 (m), 1500 (s), 1467 (w), 1444 (w), 1418 (w), 1303 (w), 1245 (m), 1179 (m), 1143 (w), 1120 (m), 1033 (m), 991 (s), 927 (m), 856 (w), 828 (m), 776 (w).
HRMS (ESI): calculated for $C_{17}H_{12}F_5O_2$ $[M+H-H_2]^+$ 343.0752, found 343.0752.
R_f = 0.33 (15% EtOAc/hexane).

1-(furan-2-yl)-2-(4-methoxyphenyl)but-3-en-1-ol (4u):

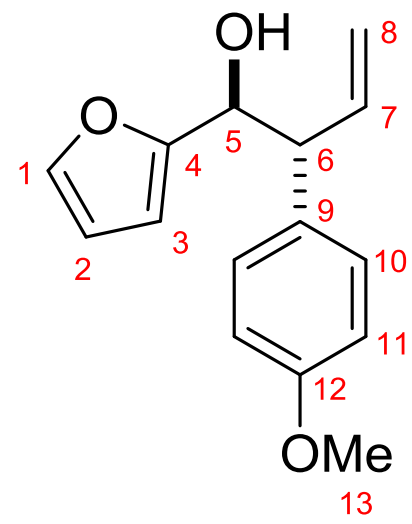

Isolated as a yellow oil (49.7 mg, 0.203 mmol, 68%) after silica gel column chromatography (eluent: 20% EtOAc/hexane), following the general batch procedure for homoallylic alcohol synthesis.

^{1}H NMR (600 MHz, $CDCl_3$): δ 7.32 (d, *J* = 1.8 Hz, 1 H, H1), 7.06 (d, *J* = 8.7 Hz, 2 H, H10), 6.78 (d, *J* = 8.7 Hz, 2 H, H11), 6.23 – 6.16 (m, 2 H, H2 and H7), 6.06 (d, *J* = 3.2 Hz, 1 H, H3), 5.25 (d, *J* = 10.3 Hz, 1 H, $H8_{cis}$), 5.23 (d, *J* = 17.1 Hz, 1 H, $H8_{trans}$), 4.85 (d, *J* = 8.0 Hz, 1 H, H5), 3.79 (t, *J* = 8.0 Hz, 1 H, H6), 3.75 (s, 3 H, H13), 2.34 (br s, 1 H, OH).
^{13}C NMR (150 MHz, $CDCl_3$): δ 158.4 (C12), 154.4 (C4), 141.9 (C1), 138.0 (C7), 132.5 (C9), 129.1 (C10), 118.2 (C8), 113.9 (C11), 110.2 (C2), 107.6 (C3), 71.1 (C5), 55.2 (C13), 55.0 (C6).
FTIR (ν_{max}, cm^{-1}): 3437 (w, OH), 2916 (w), 2837 (w), 1637 (w, C=C), 1611 (m), 1584 (w), 1511 (s), 1465 (w), 1442 (w), 1414 (w), 1303 (w), 1245 (s), 1178 (m), 1149 (m), 1033 (m), 1009 (m), 912 (m), 884 (w), 856 (w), 826 (m).
HRMS (ESI): calculated for $C_{15}H_{15}O_3$ $[M+H-H_2]^+$ 243.1016, found 243.1026.
R_f = 0.22 (20% EtOAc/hexane).

4-(1-hydroxy-3-phenylbut-3-en-1-yl)benzonitrile (4v):

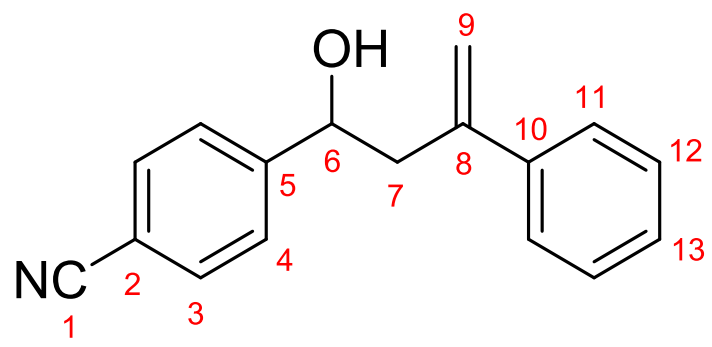

Isolated as a colourless oil (56.9 mg, 0.228 mmol, 76%) after silica gel column chromatography (eluent: 30% EtOAc/hexane), following the general batch procedure for homoallylic alcohol synthesis.

^{1}H NMR (600 MHz, $CDCl_3$): δ 7.62 (d, J = 8.1 Hz, 2 H, H3), 7.45 (d, J = 8.1 Hz, 2 H, H4), 7.42 (d, J = 7.4 Hz, 2 H, H11), 7.38 (t, J = 7.4 Hz, 2 H, H12), 7.33 (t, J = 7.4 Hz, 1 H, H13), 5.44 (s, 1 H, $H9_a$), 5.16 (s, 1 H, $H9_b$), 4.76 (dd, J = 9.0, 4.2 Hz, 1 H, H6), 3.00 (dd, J = 14.2, 4.2 Hz, 1 H, $H7_a$), 2.80 (dd, J = 14.2, 9.0 Hz, 1 H, $H7_b$), 2.20 (br s, 1 H, OH).
^{13}C NMR (150 MHz, $CDCl_3$): δ 149.2 (C5), 144.3 (C10), 139.8 (C8), 132.4 (C3), 128.8 (C12), 128.2 (C13), 126.6 (C4), 126.4 (C11), 119.0 (C1), 116.7 (C9), 111.3 (C2), 71.4 (C6), 46.2 (C7).
FTIR (ν_{max}, cm^{-1}): 3422 (w, OH), 3055 (w), 2947 (w), 2228 (m, C≡N), 1627 (w, C=C), 1609 (m), 1574 (w), 1495 (m), 1445 (m), 1408 (m), 1303 (w), 1201 (w), 1056 (m), 1017 (m), 951 (w), 905 (m), 879 (w), 838 (s), 780 (s).
HRMS (ESI): calculated for $C_{17}H_{16}NO$ $[M+H]^+$ 250.1226, found 250.1236.
R_f = 0.22 (30% EtOAc/hexane).

1,5-diphenylhex-5-en-1-yn-3-ol (4w):

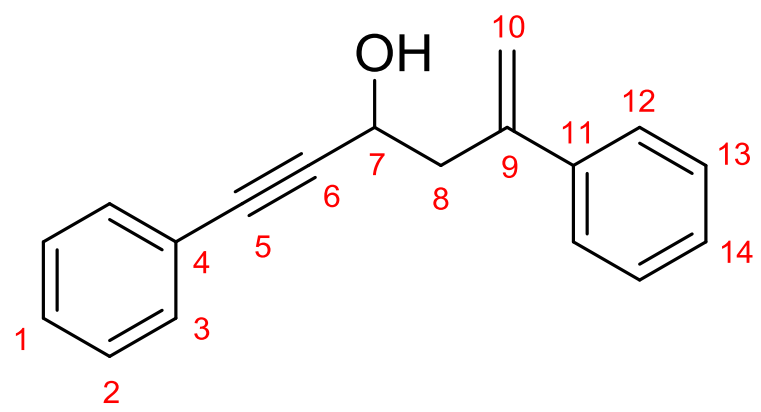

Isolated as a yellow oil (53.2 mg, 0.214 mmol, 71%) after silica gel column chromatography (eluent: 15% EtOAc/hexane), following the general batch procedure for homoallylic alcohol synthesis.

^{1}H NMR (600 MHz, $CDCl_3$): δ 7.48 – 7.45 (m, 2 H, ArH), 7.38 – 7.34 (m, 4 H, ArH), 7.30 (m, 4 H, ArH), 5.48 (d, J = 1.1 Hz, 1 H, $H10_a$), 5.30 (d, J = 1.1 Hz, 1 H, $H10_b$), 4.68 (ddd, J = 7.5, 5.9, 5.3 Hz, 1 H, H7), 3.08 (ddd, J = 14.2, 5.9, 0.9 Hz, 1 H, $H8_a$), 3.03 (ddd, J = 14.2, 7.5, 0.7 Hz, 1 H, $H8_b$), 2.02 (d, J = 5.3 Hz, 1 H, OH).
^{13}C NMR (150 MHz, $CDCl_3$): δ 144.0 (C11), 140.4 (C9), 131.8 (C2/C3/C12/C13), 128.6 (C2/C3/C12/C13), 128.5 (C1/C14), 128.4 (C2/C3/C12/C13), 127.9 (C1/C14), 126.5 (C2/C3/C12/C13), 122.7 (C4), 116.5 (C10), 89.6 (C6), 85.5 (C5), 61.6 (C7), 44.1 (C8).
FTIR (ν_{max}, cm^{-1}): 3374 (w, OH), 3057 (w), 1629 (w, C=C), 1599 (w), 1574 (w), 1490 (m), 1443 (m), 1341 (w), 1029 (s), 982 (w), 904 (m), 779 (m), 756 (s).
HRMS (ESI): calculated for $C_{18}H_{15}O$ $[M+H-H_2]^+$ 247.1117, found 247.1129.
R_f = 0.28 (15% EtOAc/hexane).

1-(3-(-1-hydroxy-2-(*p*-tolyl)but-3-en-1-yl)-1*H*-indol-1-yl)ethan-1-one (4x):

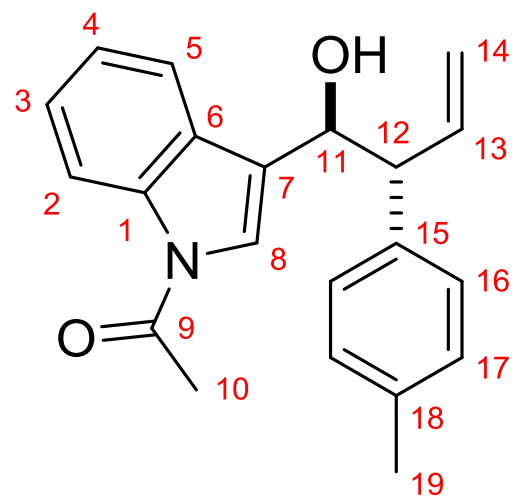

Isolated as an orange oil (57.3 mg, 0.179 mmol, 60%) after silica gel column chromatography (eluent: 40% EtOAc/hexane), following the general batch procedure for homoallylic alcohol synthesis.

^{1}H NMR (600 MHz, CDCl$_3$): δ 8.39 (d, *J* = 7.7 Hz, 1 H, H5), 7.61 (d, *J* = 7.7 Hz, 1 H, H2), 7.33 (t, *J* = 7.7 Hz, 1 H, H4), 7.25 (t, *J* = 7.7 Hz, 1 H, H3), 7.06 (appears as s, 4 H, H16 and H17), 7.04 (br s, 1 H, H8), 6.26 (ddd, *J* = 17.1, 10.3, 9.0 Hz, 1 H, H13), 5.26 (d, *J* = 10.3 Hz, 1 H, $H14_{cis}$), 5.19 (d, *J* = 17.1 Hz, 1 H, $H14_{trans}$), 5.11 (d, *J* = 6.9 Hz, 1 H, H11), 3.86 – 3.80 (m, 1 H, H12), 2.58 (br s, 1 H, OH), 2.42 (s, 3 H, H10), 2.29 (s, 3 H, H19).
^{13}C NMR (150 MHz, CDCl$_3$): δ 168.7 (C9), 138.0 (C15), 137.4 (C13), 136.5 (C18), 136.0 (C1), 129.3 (C17), 128.7 (C6), 128.1 (C16), 125.3 (C4), 123.56 (C3), 123.55 (C7), 123.0 (C8), 119.9 (C2), 118.5 (C14), 116.7 (C5), 71.4 (C11), 56.3 (C12), 23.9 (C10), 21.1 (C19).
FTIR (ν_{max}, cm^{-1}): 3434 (w, OH), 2923 (w), 1705 (s, C=O), 1606 (w, C=C), 1570 (w), 1513 (w), 1450 (s), 1382 (s), 1348 (s), 1329 (s), 1249 (s), 1219 (s), 1147 (w), 1123 (m), 1087 (w), 1034 (m), 1020 (m), 935 (m), 845 (s), 813 (m).
HRMS (ESI): calculated for $C_{21}H_{22}NO_2$ $[M+H]^+$ 320.1645, found 320.1631.
R$_f$ = 0.43 (40% EtOAc/hexane).

6,10-dimethyl-3-(*p*-tolyl)undeca-1,9-dien-4-ol (4y):

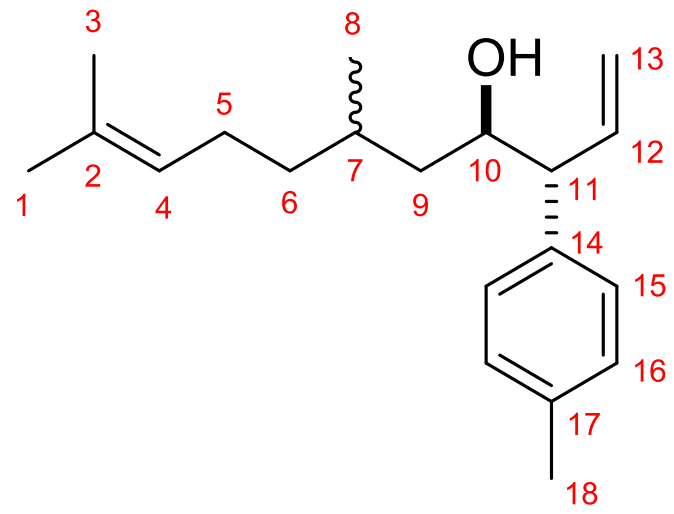

Isolated as an inseparable mixture of regioisomers (1:1) as a colourless oil (56.9 mg, 0.203 mmol, 67%) after silica gel column chromatography (eluent: 5% EtOAc/hexane), following the general batch procedure for homoallylic alcohol synthesis.

^{1}H NMR (600 MHz, CDCl$_3$): δ 7.13 (two superimposed d, *J* = 7.9 Hz, 2 H, H16), 7.11 – 7.07 (m, 2 H, H15), 6.14 – 6.06 (m, 1 H, H12), 5.23 – 5.16 (m, 2 H, $H13_{cis}$ and $H13_{trans}$), 5.09 – 5.05 (m, 1 H, H4), 3.89 – 3.84 (m, 1 H, H10), 3.20 – 3.14 (m, 1 H, H11), 2.33 (two superimposed s, 3 H, H18), 1.97 – 1.83 (m, 2 H, H5), 1.77 (s, 1 H, OH), 1.71 – 1.63 (m, 1 H, H7), 1.68 and 1.66 (two s, 3 H, H1/H3), 1.58 (two superimposed s, 3 H, H1/H3), 1.42 – 1.35 (m, 1 H, H6/H9), 1.31 – 1.08 (m, 2 H, H6/H9), 1.07 – 0.96 (m, 1 H, H6/H9), 0.88 and 0.83 (two d, *J* = 6.6 Hz, 3 H, H8).
^{13}C NMR (150 MHz, CDCl$_3$): δ 138.9 and 138.5 (C12), 138.8 and 138.7 (C14), 136.3 (C17), 131.26 and 131.24 (C2), 129.5 (C16), 127.9 (C15), 124.99 and 124.93 (C4), 117.9 and 117.8 (C13), 72.1 and 71.6 (C10), 58.0 and 57.4 (C11), 42.0 and 41.9 (C6/C9), 38.2 and 36.0 (C6/C9), 29.3 and 29.0 (C7), 25.88 and 25.87 (C1/C3), 25.7 and 25.3 (C5), 21.2 (C18), 20.6 and 18.9 (C8), 17.8 (C1/C3).
FTIR (ν_{max}, cm^{-1}): 3483 (br w, OH), 2923 (s), 1726 (w), 1637 (w, C=C), 1514 (m), 1457 (m), 1377 (m), 1251 (w), 1112 (m), 1063 (m), 1022 (m), 993 (m), 916 (s), 840 (s), 812 (s), 781 (w).
HRMS (ESI): calculated for $C_{20}H_{31}O$ $[M+H]^+$ 287.2369, found 287.2370.
R$_f$ = 0.19 (5% EtOAc/hexane).

3-benzyl-7-(5,5-dimethyl-1,3-dioxan-2-yl)hept-1-en-4-ol (4z):

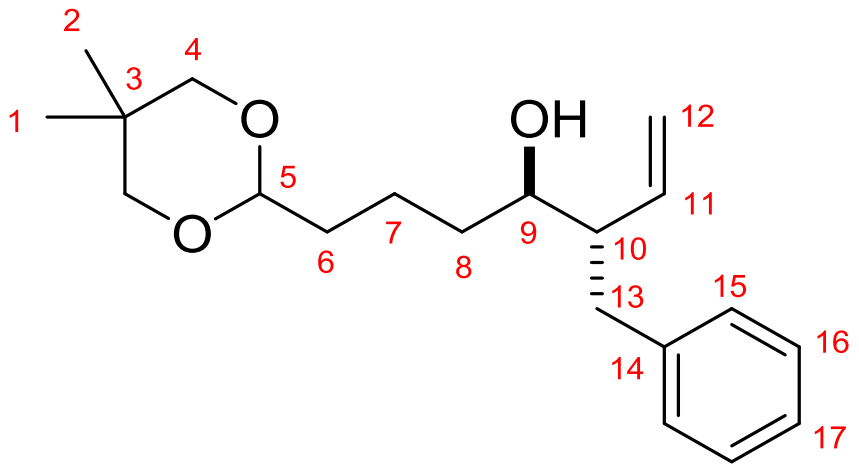

Isolated as a colourless oil (58.4 mg, 0.183 mmol, 61%) after silica gel column chromatography (eluent: 15% EtOAc/hexane), following the general batch procedure for homoallylic alcohol synthesis.

^{1}H NMR (600 MHz, $CDCl_3$): δ 7.26 (t, J = 7.5 Hz, 2 H, H16), 7.20 – 7.14 (m, 3 H, H15 and H17), 5.75 (ddd, J = 17.3, 10.3, 9.0 Hz, 2 H, H11), 5.11 (dd, J = 10.3, 1.8 Hz, 1 H, $H12_{cis}$), 4.98 (dd, J = 17.3, 1.1 Hz, 1 H, $H12_{trans}$), 4.40 (t, J = 5.0 Hz, 1 H, H, H5), 3.59 (d, J = 11.0 Hz, 2 H, $H4_a$), 3.51 (m, 1 H, H9), 3.40 (d, J = 11.0 Hz, 2 H, $H4_b$), 2.87 (dd, J = 13.6, 6.7 Hz, 1 H, $H13_a$), 2.67 (dd, J = 13.6, 8.3 Hz, 1 H, $H13_b$), 2.40 – 2.32 (m, 1 H, H10), 1.69 – 1.58 (m, 2 H, H6), 1.58 – 1.52 (m, 2 H, $H7_a$ and OH), 1.51 – 1.46 (m, 2 H, H8), 1.46 – 1.39 (m, 1 H, $H7_b$), 1.18 (s, 3 H, H1/H2), 0.71 (s, 3 H, H1/H2).
^{13}C NMR (150 MHz, $CDCl_3$): δ 140.4 (C14), 137.6 (C11), 129.4 (C15), 128.3 (C16), 126.0 (C17), 118.2 (C12), 102.2 (C5), 77.33 (C4, almost overlaps with $CDCl_3$ peak), 72.5 (C9), 51.3 (C10), 37.7 (C13), 35.1 (C8), 34.7 (C6), 30.3 (C3), 23.1 (C1/C2), 22.0 (C1/C2), 20.5 (C7).
FTIR (ν_{max}, cm^{-1}): 3465 (w, OH), 3027 (w), 2952 (m), 2850 (m), 1734 (w), 1638 (w, C=C), 1603 (w), 1496 (w), 1455 (m), 1394 (m), 1363 (w), 1311 (w), 1237 (w), 1176 (w), 1135 (s), 1080 (m), 1015 (s), 972 (m), 913 (s), 837 (w), 791 (w).
HRMS (ESI): calculated for $C_{20}H_{30}O_3Na$ $[M+Na]^+$ 341.2087, found 341.2081.
R_f = 0.20 (15% EtOAc/hexane).

(*E*)-1-phenyl-4-(*p*-tolyl)hexa-1,5-dien-3-ol (4aa):

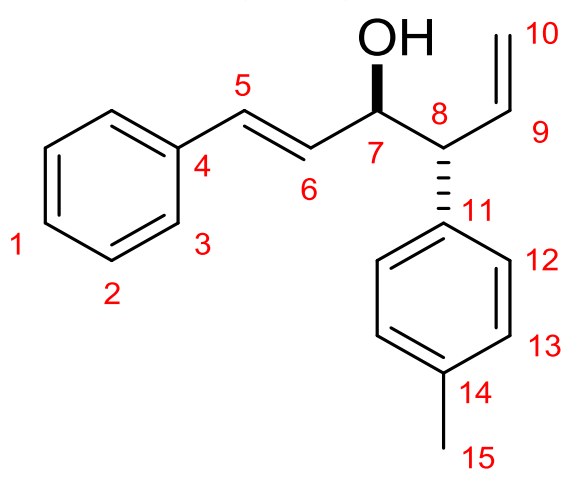

Isolated as a yellow oil (49.3 mg, 0.186 mmol, 62%) after silica gel column chromatography (eluent: 15% EtOAc/hexane), following the general batch procedure for homoallylic alcohol synthesis.

^{1}H NMR (600 MHz, $CDCl_3$): δ 7.32 – 7.27 (m, 4 H, H2 and H3), 7.25 – 7.21 (m, 1 H, H1), 7.18 – 7.13 (m, 4 H, H12 and H13), 6.57 (dd, J = 15.9, 1.2 Hz, 1 H, H5), 6.21 (ddd, J = 17.1, 10.3, 8.8 Hz, 1 H, H9), 6.13 (dd, J = 15.9, 6.1 Hz, 1 H, H6), 5.30 – 5.22 (m, 2 H, $H10_{cis}$ and $H10_{trans}$), 4.51 (m, 1 H, H7), 3.49 – 3.43 (m, 1 H, H8), 2.34 (s, 3 H, H15), 2.05 (d, J = 3.6 Hz, 1 H, OH).
^{13}C NMR (150 MHz, $CDCl_3$): δ 138.1 (C9), 137.6 (C11), 137.0 (C4), 136.5 (C14), 131.1 (C5), 130.0 (C6), 129.4 (C13), 128.6 (C3), 128.4 (C12), 127.6 (C1), 126.6 (C2), 118.1 (C10), 75.1 (C7), 57.3 (C8), 21.2 (C15).

FTIR (ν_{max}, cm^{-1}): 3420 (br w, OH), 3025 (w), 2923 (w), 1670 (w, C=C), 1637 (w, C=C), 1600 (w), 1514 (m), 1495 (m), 1449 (m), 1381 (w), 1297 (w), 1112 (m), 1070 (m), 966 (s), 919 (m), 812 (s), 781 (w).
HRMS (ESI): calculated for $C_{19}H_{19}O$ $[M+H-H_2]^+$ 263.1430, found 263.1438.
R_f = 0.36 (15% EtOAc/hexane).

Methyl 4-(2-(chloromethyl)-1-hydroxybut-3-en-1-yl)benzoate (4ab):

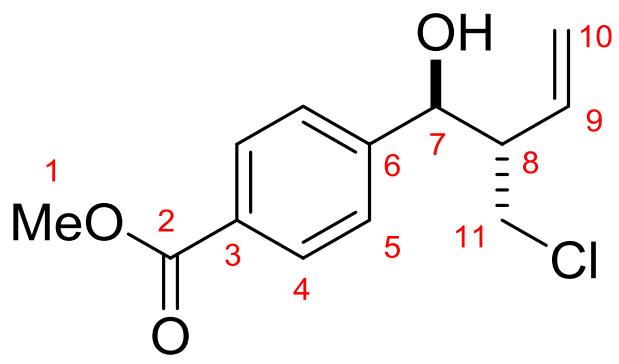

Isolated as a colourless oil (49.7 mg, 0.195 mmol, 66%) after silica gel column chromatography (eluent: 15% EtOAc/hexane), following the general batch procedure for homoallylic alcohol synthesis.

^{1}H NMR (600 MHz, $CDCl_3$): δ 8.01 (d, *J* = 8.3 Hz, 2 H, H4), 7.43 (d, *J* = 8.3 Hz, 2 H, H5), 5.82 (ddd, *J* = 17.3, 10.4, 8.7 Hz, 1 H, H9), 5.25 (dd, *J* = 10.4, 1.0 Hz, 1 H, $H10_{cis}$), 5.12 (d, *J* = 17.3 Hz, 1 H, $H10_{trans}$), 5.01 (dd, *J* = 5.1, 3.7 Hz, 1 H, H7), 3.91 (s, 3 H, H1), 3.66 (dd, *J* = 11.0, 6.6 Hz, 1 H, $H11_a$), 3.41 (dd, *J* = 11.0, 5.5 Hz, 1 H, $H11_b$), 2.69 – 2.62 (m, 1 H, H8), 2.25 (d, *J* = 3.7 Hz, 1 H, OH).
^{13}C NMR (150 MHz, $CDCl_3$): δ 167.0 (C2), 147.2 (C6), 134.1 (C9), 129.8 (C4), 129.7 (C3), 126.4 (C5), 120.6 (C10), 72.8 (C7), 53.6 (C8), 52.3 (C1), 45.5 (C11).
FTIR (ν_{max}, cm^{-1}): 3468 (w, OH), 2955 (w), 1703 (m, C=O), 1611 (w, C=C), 1577 (w), 1437 (m), 1418 (w), 1311 (m), 1277 (s), 1193 (w), 1178 (w), 1111 (m), 1076 (w), 1018 (w), 996 (w), 966 (w), 924 (w), 860 (w), 824 (w), 775 (w), 755 (w).
HRMS (ESI): calculated for $C_{13}H_{16}ClO_3$ $[M+H]^+$ 255.0782, found 255.0790.
R_f = 0.24 (15% EtOAc/hexane).

2-(chloromethyl)-1-(3-nitrophenyl)but-3-en-1-ol (4ac):

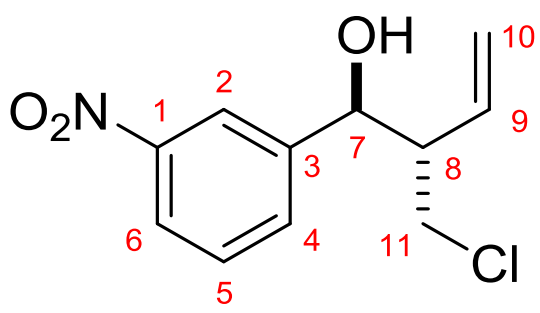

Isolated as a colourless oil (66.7 mg, 0.276 mmol, 92%) after silica gel column chromatography (eluent: 40% Et_2O/hexane), following the general batch procedure for homoallylic alcohol synthesis.

^{1}H NMR (600 MHz, $CDCl_3$): δ 8.22 (t, *J* = 1.5 Hz, 1 H, H2), 8.12 (dd, *J* = 7.9, 1.5 Hz, 1 H, H6), 7.68 (d, *J* = 7.9 Hz, 1 H, H4), 7.52 (t, *J* = 7.9 Hz, 1 H, H5), 5.81 (ddd, *J* = 17.3, 10.3, 8.9 Hz, 1 H, H9), 5.25 (d, *J* = 10.4 Hz, 1 H, $H10_{cis}$), 5.14 – 5.06 (m, 2 H, H7 and $H10_{trans}$), 3.71 (dd, *J* = 11.0, 7.1 Hz, 1 H, $H11_a$), 3.45 (dd, *J* = 11.0, 5.3 Hz, 1 H, $H11_b$), 2.71 – 2.61 (m, 1 H, H8), 2.40 (br s, 1 H, OH).
^{13}C NMR (150 MHz, $CDCl_3$): δ 148.4 (C1), 144.4 (C3), 133.4 (C9), 132.6 (C4), 129.4 (C5), 122.8 (C6), 121.4 (C2), 121.1 (C10), 72.0 (C7), 53.5 (C8), 45.3 (C11).
FTIR (ν_{max}, cm^{-1}): 3518 (w, OH), 3080 (w), 1638 (w, C=C), 1583 (w), 1526 (s, N=O), 1480 (w), 1440 (w), 1422 (w), 1348 (s, N=O), 1313 (m), 1255 (w), 1204 (w), 1092 (w), 1067 (w), 995 (w), 926 (m), 908 (m), 838 (w), 810 (m), 788 (w).
HRMS (ESI): calculated for $C_{11}H_{13}ClNO_3$ $[M+H]^+$ 242.0578, found 242.0580.
R_f = 0.39 (40% Et_2O/hexane).

2-benzyl-1-(2-chloro-6-methoxyquinolin-3-yl)but-3-en-1-ol (4ad):

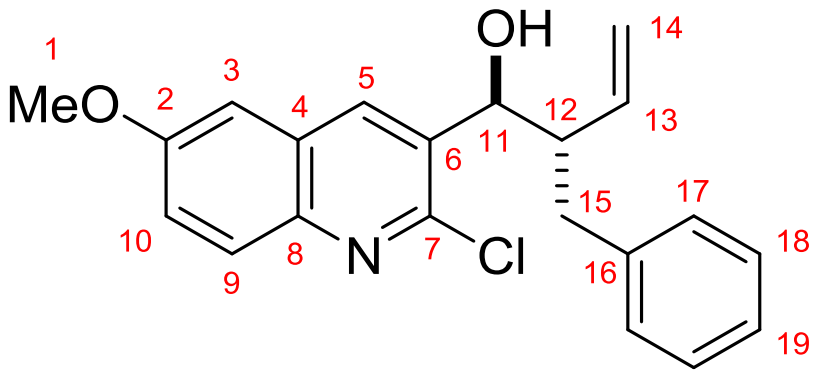

Isolated as a white solid (88.0 mg, 0.249 mmol, 83%) after silica gel column chromatography (eluent: 15% EtOAc/hexane), following the general batch procedure for homoallylic alcohol synthesis.

^{1}H NMR (600 MHz, $CDCl_3$): δ 8.06 (s, 1 H, H5), 7.82 (d, *J* = 9.2 Hz, 1 H, H9), 7.33 – 7.26 (m, 3 H, H10 and H18), 7.24 (d, *J* = 7.0 Hz, 2 H, H17), 7.20 (t, *J* = 7.2 Hz, 1 H, H19), 6.98 (d, *J* = 2.7 Hz, 1 H, H3), 5.79 (ddd, *J* = 17.3, 10.4, 8.3 Hz, 1 H, H13), 5.08 (t, *J* = 3.7 Hz, 1 H, H11), 5.01 (dd, *J* = 10.4, 1.1 Hz, 1 H, $H14_{cis}$), 4.74 (d, *J* = 17.3 Hz, 1 H, $H14_{trans}$), 3.89 (s, 3 H, H1), 3.00 – 2.91 (m, 2 H, H15), 2.85 (qd, *J* = 8.3, 3.7 Hz, 1 H, H12), 2.70 (d, *J* = 3.7 Hz, 1 H, OH).
^{13}C NMR (150 MHz, $CDCl_3$): δ 158.2 (C2), 146.0 (C7), 142.8 (C8), 139.6 (C16), 135.92 (C5/C13), 135.91 (C5/C13), 135.3 (C6), 129.4 (two overlapping signals, C9 and C17), 128.4 (C18), 128.2 (C4), 126.3 (C19), 123.0 (C10), 119.0 (C14), 105.2 (C3), 70.9 (C11), 55.6 (C1), 50.5 (C12), 38.2 (C15).
FTIR (ν_{max}, cm^{-1}): 3364 (w, OH), 3065 (w), 3027 (w), 2915 (w), 1623 (m, C=C), 1591 (m), 1496 (s), 1465 (w), 1455 (m), 1418 (w), 1380 (w), 1339 (m), 1295 (w), 1227 (s), 1165 (m), 1125 (w), 1085 (w), 1049 (s), 1030 (s), 998 (w), 959 (w), 911 (s), 829 (s), 793 (w).
HRMS (ESI): calculated for $C_{21}H_{21}ClNO_2$ $[M+H]^+$ 354.1255, found 354.1267.
R_f = 0.20 (15% EtOAc/hexane).

1-(2-chloro-6-methoxyquinolin-3-yl)-2-vinylhexan-1-ol (4ae):

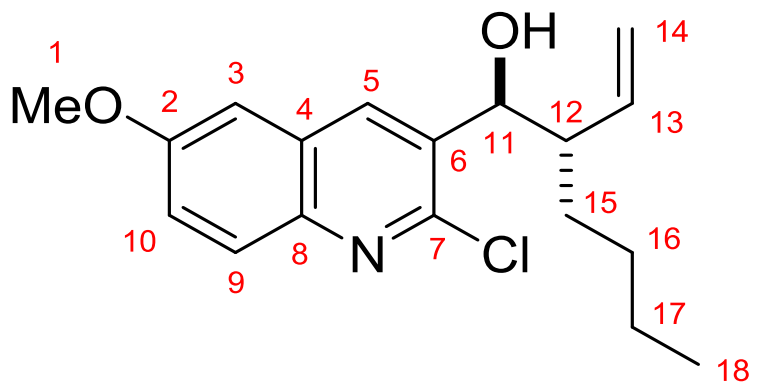

Isolated as a white solid (62.6 mg, 0.194 mmol, 65%) after silica gel column chromatography (eluent: 40% EtOAc/hexane), following the general batch procedure for homoallylic alcohol synthesis.

^{1}H NMR (600 MHz, $CDCl_3$): δ 8.11 (s, 1 H, H5), 7.88 (d, *J* = 9.2 Hz, 1 H, H9), 7.34 (dd, *J* = 9.2, 2.8 Hz, 1 H, H10), 7.06 (d, *J* = 2.8 Hz, 1 H, H3), 5.71 (ddd, *J* = 17.3, 10.2, 9.0 Hz, 1 H, H13), 5.18 – 5.10 (m, 2 H, H11 and $H14_{cis}$), 4.92 (dd, *J* = 17.3, 1.0 Hz, 1 H, $H14_{trans}$), 3.91 (s, 3 H, H1), 2.52 – 2.45 (m, 1 H, H12), 2.38 (d, *J* = 3.8 Hz, 1 H, OH), 1.62 – 1.52 (m, 1 H, $H15_a$), 1.52 – 1.43 (m, 1 H, $H15_b$), 1.40 – 1.33 (m, 1 H, $H16_a$), 1.33 – 1.23 (m, 3 H, $H16_b$ and H17), 0.86 (t, *J* = 7.1 Hz, 3 H, H18).
^{13}C NMR (150 MHz, $CDCl_3$): δ 158.3 (C2), 146.6 (C7), 143.0 (C8), 137.3 (C13), 135.8 (C5), 135.2 (C6), 129.6 (C9), 128.4 (C4), 123.1 (C10), 119.1 (C14), 105.3 (C3), 72.4 (C11), 55.7 (C1), 50.6 (C12), 31.1 (C15), 29.7 (C16), 22.7 (C17), 14.1 (C18).
FTIR (ν_{max}, cm^{-1}): 3359 (w, OH), 2957 (m), 2931 (m), 2858 (m), 1623 (m, C=C), 1591 (m), 1497 (s), 1465 (w), 1420 (w), 1379 (w), 1338 (m), 1228 (s), 1166 (m), 1118 (w), 1046 (m), 1001 (w), 959 (w), 917 (m), 830 (m).
HRMS (ESI): calculated for $C_{18}H_{23}NO_2Cl$ $[M+H]^+$ 320.1412, found 320.1411.

$\boldsymbol{R_f}$ = 0.35 (40% EtOAc/hexane).

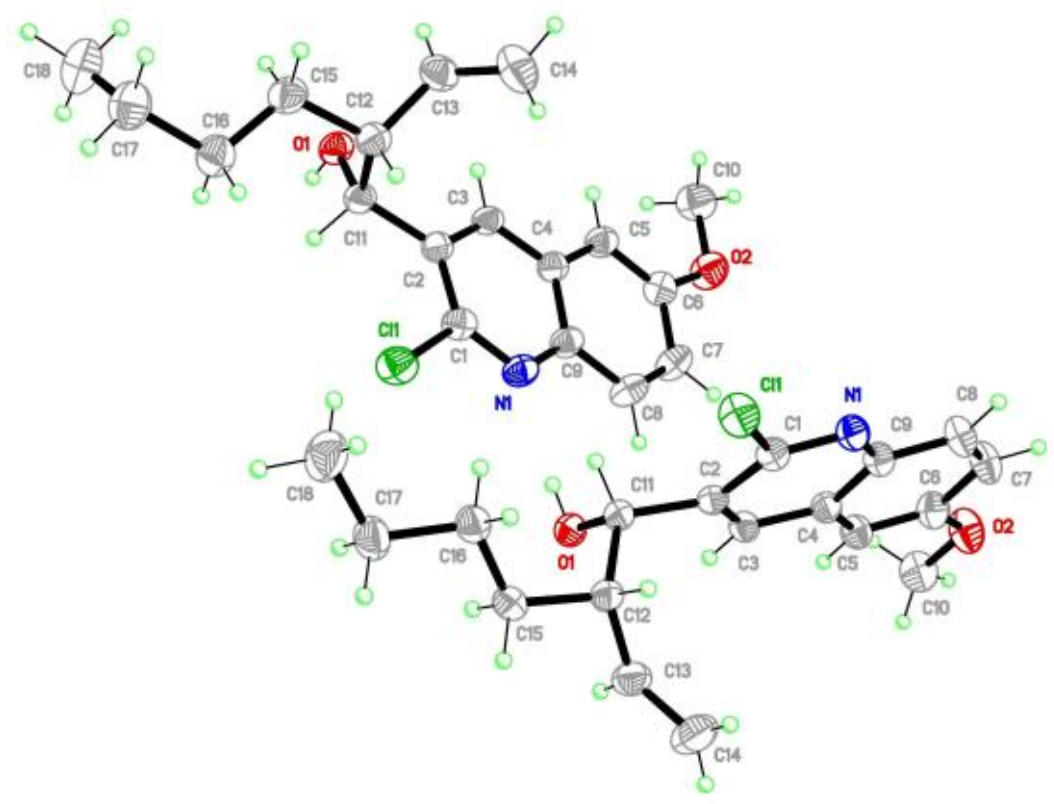

Summary of Data **CCDC 1483699**

Compound Name: Formula: $C_{18}H_{22}ClNO_2$

Unit Cell Parameters: a 9.46790(10) b 11.3192(2) c 16.4547(3) P-1

2.3. Iterative polyol sequence

1-(4-bromophenyl)-2-(chloromethyl)but-3-en-1-yl)oxy)triethylsilane (5):

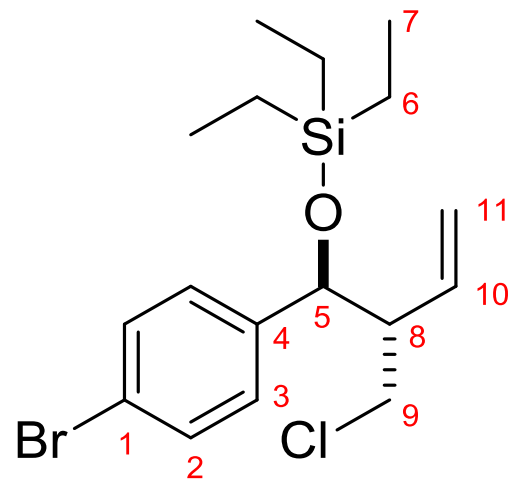

To a solution of **4n** (43.5 mg, 0.156 mmol, 1 equiv.) in anhydrous CH_2Cl_2 (1 mL) at 0 °C was added DIPEA (0.06 mL, 0.312 mmol, 2 equiv.) then triethylsilyl trifluoromethanesulfonate (0.06 mL, 0.234 mmol, 1.5 equiv.). The mixture was warmed to r.t. and stirred for 15 min, then partitioned between EtOAc (15 mL) and sat. aq. $NaHCO_3$ solution (15 mL). The organic layer was separated and the aqueous layer extracted further with EtOAc (3 × 10 mL). The combined organic extracts were dried ($MgSO_4$) and evaporated under reduced pressure. The residue was purified by silica gel column chromatography (eluent: hexane) to provide the title product **5** (58.2 mg, 0.149 mmol, 95%) as a colourless oil.

^{1}H NMR (600 MHz, $CDCl_3$): δ 7.42 (d, J = 8.3 Hz, 2 H, H2), 7.16 (d, J = 8.3 Hz, 2 H, H3), 5.74 (ddd, J = 17.4, 10.2, 9.0 Hz, 1 H, H10), 5.11 (dd, J = 10.4, 0.7 Hz, 1 H, $H11_{cis}$), 4.98 (d, J = 3.7 Hz, 1 H, H5), 4.93 (dd, J = 17.3, 0.7 Hz, 1 H, $H11_{trans}$), 3.70 (dd, J = 10.7, 7.7 Hz, 1 H, $H9_a$), 3.37 (dd, J = 10.7, 5.8 Hz, 1 H, $H9_b$), 2.52 – 2.45 (m, 1 H, H8), 0.89 (t, J = 8.0 Hz, 9 H, H7), 0.54 (q, J = 8.0 Hz, 6 H, H6).
^{13}C NMR (150 MHz, $CDCl_3$): δ 142.3 (C1), 134.3 (C10), 131.1 (C2), 128.3 (C3), 121.1 (C4), 119.4 (C11), 73.5 (C5), 55.0 (C8), 45.8 (C9), 6.9 (C7), 4.9 (C6).
FTIR (ν_{max}, cm^{-1}): 2956 (w), 2912 (w), 2877 (w), 1640 (w, C=C), 1592 (w), 1486 (m), 1458 (w), 1406 (w), 1363 (w), 1294 (w), 1239 (w), 1201 (w), 1107 (m), 1085 (s), 1003 (s), 922 (m), 872 (w), 826 (m), 788 (w).
HRMS (ESI): calculated for $C_{17}H_{27}BrClOSi$ $[M+H]^+$ 389.0698, found 389.0703.
$\boldsymbol{R_f}$ = 0.49 (hexane).

1-(4-bromophenyl)-2-(chloromethyl)-4-(4-fluorophenyl)-1-((triethylsilyl)oxy)hex-5-en-3-ol (5a):

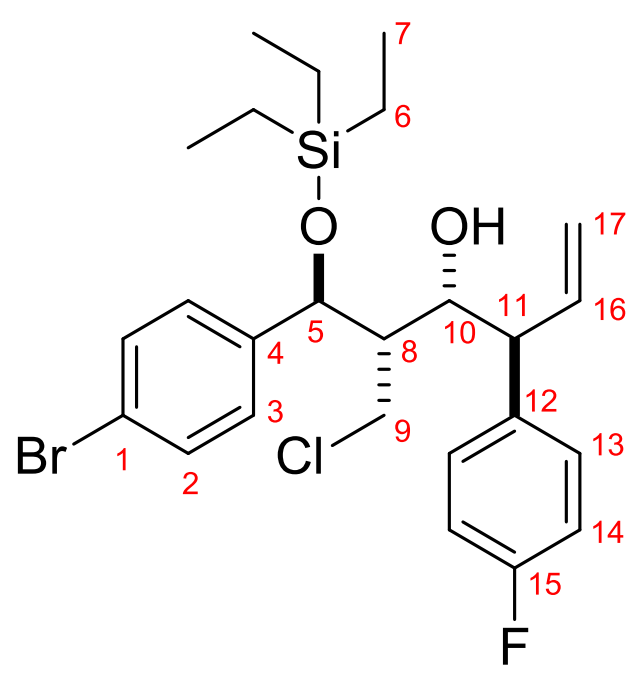

To a solution of **5** (55.8 mg, 0.143 mmol) in CH_2Cl_2/MeOH (9:1, 15 mL) was bubbled O_3/O_2 at -78 °C until the reaction mixture turned blue (ca. 5-10 min). Argon was then bubbled through the solution until residual O_3 had disappeared. Polymer-supported PPh_3 (3 mmol/g loading, 144 mg, ca. 0.42 mmol) was then added and the mixture stirred at r.t. for 1 h. The polymer-supported reagent was then filtered off and the filtrate evaporated under reduced pressure to provide the crude aldehyde, which was used immediately without further purification.

To a solution of the crude aldehyde and *trans*-2-(4-fluorophenyl)vinylboronic acid (35.7 mg, 0.21 mmol, 1.5 equiv.) in CH_2Cl_2/THF (4:1, 1.5 mL) was added (trimethylsilyl)diazomethane (0.14 mL, 2 M in hexanes, 0.28 mmol, 2.0 equiv.) and the mixture subsequently stirred for 2 h. The mixture was quenched with SiO_2 and stirred for a further 10 min, filtered and evaporated under reduced pressure. The residue was purified by silica gel column chromatography (eluent: 5% EtOAc/hexane) to provide the title product **5a** (57.6 mg, 0.140 mmol, 98% over 2 steps) as a colourless viscous oil.

^{1}H NMR (600 MHz, $CDCl_3$): δ 7.45 (d, *J* = 8.3 Hz, 2 H, H2), 7.02 (d, *J* = 8.3 Hz, 2 H, H3), 6.82 (t, *J* = 8.6 Hz, 2 H, H14), 6.62 (dd, *J* = 8.6, 5.4 Hz, 2 H, H13), 5.94 (ddd, *J* = 17.1, 10.3, 7.8 Hz, 1 H, H16), 5.28 (s, 1 H, H5), 5.06 (d, *J* = 10.3 Hz, 1 H, $H17_{cis}$), 4.96 (d, *J* = 17.1 Hz, 1 H, $H17_{trans}$), 3.99 (d, *J* = 10.3 Hz, 1 H, H10), 3.85 (dd, *J* = 11.5, 3.4 Hz, 1 H, $H9_a$), 3.79 (t, *J* = 11.5 Hz, 1 H, $H9_b$), 3.52 (s, 1 H), 3.31 (dd, *J* = 10.3, 7.8 Hz, 1 H, H11), 1.51 – 1.46 (m, 1 H, H8), 0.92 (t, *J* = 8.0 Hz, 9 H, H7), 0.61 (q, *J* = 8.0 Hz, 6 H, H6).
^{13}C NMR (150 MHz, $CDCl_3$): δ 161.6 (d, *J* = 245.4 Hz, C15), 141.5 (C1), 139.4 (C16), 136.2 (d, *J* = 3.3 Hz, C12), 131.4 (C2), 129.1 (d, *J* = 7.8 Hz, C13), 127.5 (C3), 121.2 (C4), 116.4 (C17), 115.7 (d, *J* = 21.2 Hz, C14), 74.0 (C5), 71.6 (C10), 53.2 (C11), 49.7 (C8), 41.9 (C9), 6.9 (C7), 4.7 (C6).
^{19}F NMR (376 MHz, $CDCl_3$): δ -115.7 (s, 1 F, F15).
FTIR (ν_{max}, cm^{-1}): 3509 (br w, OH), 2957 (w), 2878 (w), 1637 (w, C=C), 1604 (w), 1509 (m), 1486 (w), 1458 (w), 1440 (w), 1224 (m), 1194 (w), 1159 (w), 1067 (s), 995 (s), 919 (m), 828 (s), 799 (w).
HRMS (ESI): calculated for $C_{25}H_{34}BrClFO_2Si$ $[M+H]^+$ 527.1179, found 527.1184.
R_f = 0.30 (5% EtOAc/hexane).

4-(4-bromophenyl)-5-(chloromethyl)-6-(1-(4-fluorophenyl)allyl)-2,2-dimethyl-1,3-dioxane (6):

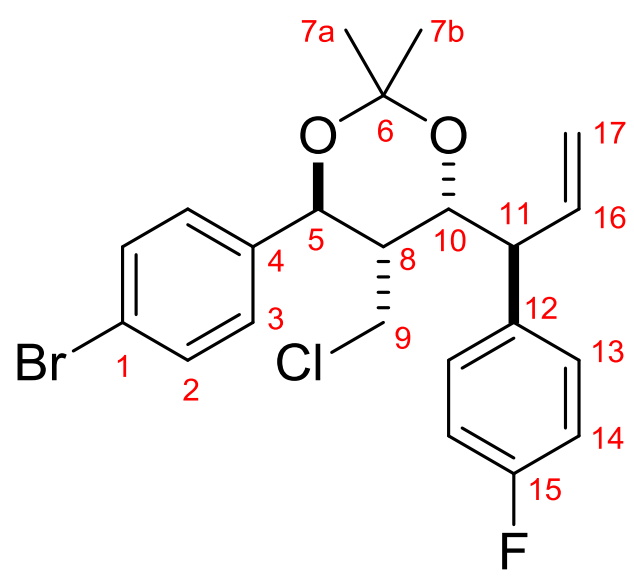

To a solution of **5a** (102 mg, 0.193 mmol) in acetone/2,2-dimethoxypropane (4:1, 4 mL) was added (±)-camphorsulfonic acid (9.0 mg, 0.039 mmol). The mixture was stirred at r.t. for 16 h. The reaction was then quenched with Et_3N (4 drops) and the solvent removed under reduced pressure. The residue was purified by silica gel column chromatography (eluent: 5% EtOAc/hexane) to provide the title product **6** (79.8 mg, 0.176 mmol, 91%) as a white foam.

^{1}H NMR (600 MHz, $CDCl_3$): δ 7.46 (d, *J* = 8.4 Hz, 2 H, H2), 7.26 (d, *J* = 8.4 Hz, 2 H, H3), 7.21 (dd, *J* = 8.6, 5.3 Hz, 2 H, H13), 7.04 (t, *J* = 8.6 Hz, 2 H, H14), 6.12 (ddd, *J* = 17.2, 10.3, 6.2 Hz, 1 H, H16), 5.09 (d, *J* = 10.3 Hz, 1 H, $H17_{cis}$), 4.89 (d, *J* = 6.9 Hz, 1 H, H5), 4.85 (d, *J* = 17.2 Hz, 1 H, $H17_{trans}$), 4.44 (dd, *J* = 10.9, 4.5 Hz, 1 H, H10), 3.67 (dd, *J* = 11.6, 7.5 Hz, 1 H, $H9_a$), 3.61 (dd, *J* = 10.9, 6.2 Hz, 1 H, H11), 3.46 (dd, *J* = 11.6, 3.5 Hz, 1 H, $H9_b$), 2.11 – 2.06 (m, 1 H, H8), 1.54 (s, 3 H, $H7_a$/$H7_b$), 1.44 (s, 3 H, $H7_a$/$H7_b$).
^{13}C NMR (150 MHz, $CDCl_3$): δ 161.9 (d, *J* = 246.1 Hz, C15), 140.7 (C1), 139.7 (d, *J* = 0.9 Hz, C16), 135.6 (d, *J* = 3.4 Hz, C12), 131.8 (C2), 129.7 (d, *J* = 7.9 Hz, C13), 129.0 (C3),

121.9 (C4), 116.4 (C17), 116.1 (d, *J* = 21.3 Hz, C14), 101.9 (C6), 72.9 (C5), 71.0 (C10), 49.1 (C11), 45.8 (C8), 42.7 (C9), 25.0 ($C7_a/C7_b$), 24.6 ($C7_a/C7_b$).
^{19}F NMR (376 MHz, $CDCl_3$): δ -115.0 (s, 1 F, F15).
FTIR (ν_{max}, cm^{-1}): 2986 (w), 2936 (w), 1637 (w, C=C), 1604 (w), 1509 (s), 1489 (m), 1445 (w), 1409 (w), 1380 (m), 1281 (w), 1220 (s), 1159 (s), 1092 (m), 1071 (m), 1053 (m), 1009 (s), 910 (m), 889 (w), 832 (s), 804 (m), 774 (w).
HRMS (ESI): calculated for $C_{22}H_{24}BrClFO_2$ $[M+H]^+$ 453.0627, found 453.0616.
R_f = 0.24 (5% EtOAc/hexane).

1-(6-(4-bromophenyl)-5-(chloromethyl)-2,2-dimethyl-1,3-dioxan-4-yl)-1-(4-fluoro-phenyl)-3-(*p*-tolyl)pent-4-en-2-ol (6a):

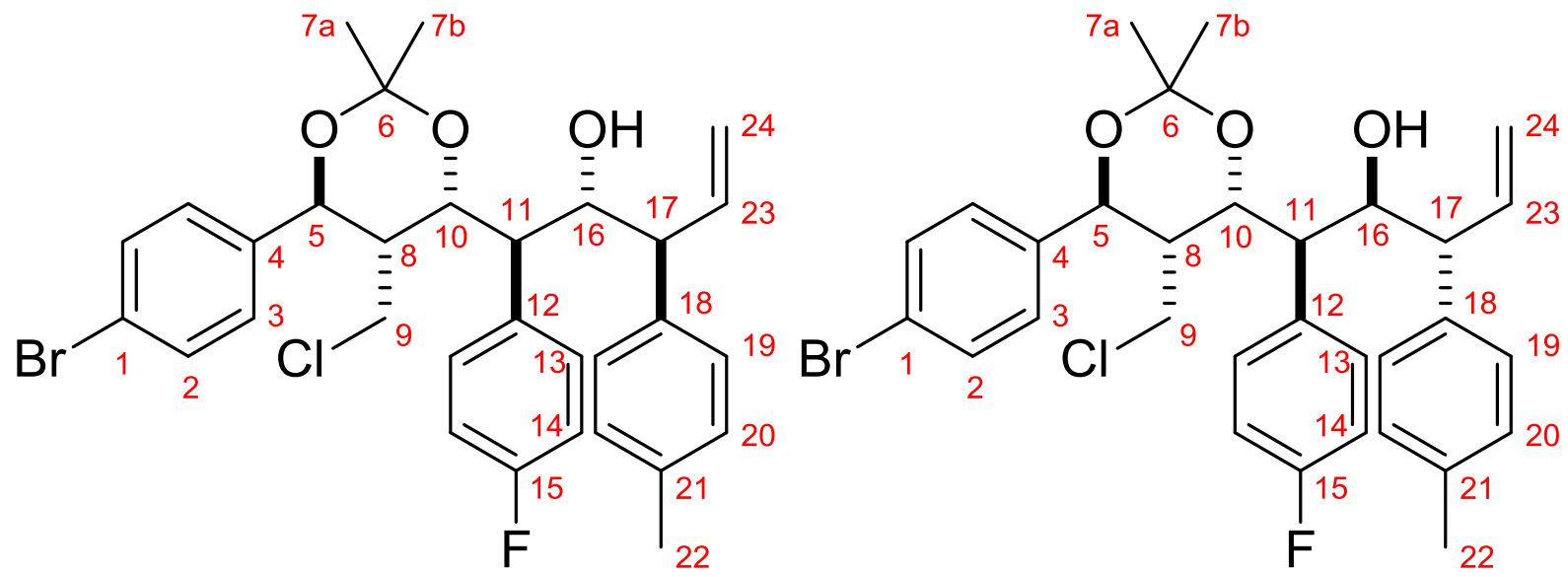

To a solution of **6** (79.8 mg, 0.176 mmol) in CH_2Cl_2/MeOH (9:1, 15 mL) was bubbled O_3/O_2 at -78 °C until the reaction mixture turned blue (ca. 5 min). Argon was then bubbled through the solution until residual O_3 had disappeared. Polymer-supported PPh_3 (3 mmol/g loading, 175 mg, ca. 0.53 mmol) was then added and the mixture stirred at r.t. for 1 h. The polymer-supported reagent was then filtered off and the filtrate evaporated under reduced pressure to provide the crude aldehyde, which was used immediately without further purification.

To a solution of the crude aldehyde and *trans*-2-(4-methylphenyl)vinylboronic acid (43.7 mg, 0.27 mmol, 1.5 equiv.) in CH_2Cl_2/THF (4:1, 2 mL) was added (trimethylsilyl)diazomethane (0.18 mL, 2 M in hexanes, 0.36 mmol, 2.0 equiv.) and the mixture subsequently stirred for 2 h. The mixture was quenched with SiO_2 and stirred for a further 10 min, filtered and evaporated under reduced pressure. The residue was purified by silica gel column chromatography (eluent: 10% EtOAc/hexane) to provide the title products **6a** as separable diastereomers (major diastereomer: 63.7 mg, 0.108 mmol; minor diastereomer: 10.1 mg, 0.017 mmol; combined yield 71% over 2 steps, d.r. 6:1) as white foams.

Major diastereomer:
^{1}H NMR (600 MHz, $CDCl_3$): δ 7.44 (d, *J* = 8.4 Hz, 2 H, H2), 7.23 – 7.17 (m, 4 H, H3 and H13), 7.10 (d, *J* = 8.0 Hz, 2 H, H20), 7.09 – 7.04 (m, 4 H, H14 and H19), 6.30 (dt, *J* = 17.3, 10.0 Hz, 1 H, H23), 5.24 (dd, *J* = 10.0, 1.4 Hz, 1 H, $H24_{cis}$), 4.93 (d, *J* = 17.3 Hz, 1 H, $H24_{trans}$), 4.90 (d, *J* = 6.1 Hz, 1 H, H5), 4.77 (dd, *J* = 11.0, 3.9 Hz, 1 H, H10), 4.11 (dd, *J* = 7.2, 3.5 Hz, 1 H, H16), 4.07 (s, 1 H, OH), 3.74 (dd, *J* = 11.4, 8.5 Hz, 1 H, $H9_a$), 3.52 (dd, *J* = 11.4, 3.3 Hz, 1 H, $H9_b$), 3.12 (dd, *J* = 10.0, 3.5 Hz, 1 H, H17), 3.07 (dd, *J* = 11.0, 7.2 Hz, 1 H, H11), 2.32 (s, 3 H, H22), 1.89 – 1.84 (m, 1 H, H8), 1.55 (s, 3 H, $H7_a/H7_b$), 1.50 (s, 3 H, $H7_a/H7_b$).
^{13}C NMR (150 MHz, $CDCl_3$): δ 162.1 (d, *J* = 247.0 Hz, C15), 140.5 (C1), 140.0 (C18), 137.0 (C23), 136.0 (C21), 134.4 (d, *J* = 3.3 Hz, C12), 131.8 (C2), 130.2 – 129.8 (br, C13), 129.2 (C20), 128.8 (C3), 128.1 (C19), 122.0 (C4), 118.2 (C24), 116.2 (d, *J* = 21.2 Hz, C14), 102.1 (C6), 80.1 (C16), 73.1 (C5/C10), 73.0 (C5/C10), 52.7 (C17), 47.6 (C11), 45.1 (C8), 42.6 (C9), 25.6 ($C7_a/C7_b$), 25.0 ($C7_a/C7_b$), 21.2 (C22).
^{19}F NMR (376 MHz, $CDCl_3$): δ -114.0 (s, 1 F, F15).

FTIR (ν_{max}, cm^{-1}): 3487 (br w, OH), 2989 (w), 2939 (w), 1606 (w, C=C), 1509 (s), 1490 (w), 1445 (w), 1409 (w), 1382 (m), 1223 (s), 1160 (m), 1113 (m), 1097 (m), 1054 (m), 1010 (m), 973 (w), 910 (m), 835 (s), 812 (m).
HRMS (ESI): calculated for $C_{31}H_{33}BrClFO_3Na$ $[M+Na]^+$ 609.1178, found 609.1165.
R_f = 0.37 (10% EtOAc/hexane).

Minor diastereomer:
^{1}H NMR (600 MHz, $CDCl_3$): δ 7.41 (d, *J* = 8.3 Hz, 2 H, H2), 7.21 (d, *J* = 8.3 Hz, 2 H, H3), 7.16 (d, *J* = 7.8 Hz, 2 H, H19), 6.98 (br s, 2 H, H14), 6.95 (d, *J* = 7.8 Hz, 2 H, H20), 5.87 (dt, *J* = 17.0, 10.0 Hz, 1 H, H23), 5.04 (d, *J* = 10.0 Hz, 1 H, $H24_{cis}$), 4.97 (d, *J* = 17.0 Hz, 1 H, $H24_{trans}$), 4.80 – 4.76 (m, 2 H, H5 and H10), 4.52 – 4.48 (m, 1 H, H16), 3.47 (dd, *J* = 11.3, 8.6 Hz, 1 H, $H9_a$), 3.20 (dd, *J* = 11.3, 3.3 Hz, 1 H, $H9_b$), 2.78 (d, *J* = 11.5 Hz, 1 H, H11), 2.67 (t, *J* = 10.0 Hz, 1 H, H17), 2.37 (s, 3 H, H22), 1.86 – 1.81 (m, 1 H, H8), 1.77 (d, *J* = 3.6 Hz, 1 H, OH), 1.525 (s, 3 H, $H7_a$/$H7_b$), 1.517 (s, 3 H, $H7_a$/$H7_b$). (N.B. 2 H corresponding to H13 appear broadened over 7.50 – 6.50 region).
^{13}C NMR (150 MHz, $CDCl_3$): δ 162.2 (d, *J* = 246.1 Hz, C15), 141.1 (C1), 140.7 (C23), 137.3 (C18), 136.6 (C21), 131.7 (d, *J* = 3.4 Hz, C12), 131.7 (C2), 129.7 (C20), 129.1 (C3), 128.2 (C19), 121.8 (C4), 117.3 (C24), 115.5 – 115.0 (br, C14), 101.9 (C6), 73.3 (C5), 70.0 (C16), 68.1 (C10), 55.2 (C17), 45.9 (C11), 45.1 (C8), 43.0 (C9), 25.3 ($C7_a$/$C7_b$), 24.8 ($C7_a$/$C7_b$), 21.3 (C22). (N.B. signal corresponding to C13 too broadened to distinguish accurately).
^{19}F NMR (376 MHz, $CDCl_3$): δ -114.9 (s, 1 F, F15).
FTIR (ν_{max}, cm^{-1}): 3569 (br w, OH), 2990 (w), 2934 (w), 1606 (w, C=C), 1509 (s), 1490 (w), 1445 (w), 1380 (m), 1286 (w), 1223 (s), 1161 (m), 1102 (w), 1070 (m), 1010 (m), 974 (w), 910 (w), 845 (w), 812 (m), 784 (w).
HRMS (ESI): calculated for $C_{31}H_{33}BrClFO_3Na$ $[M+Na]^+$ 609.1178, found 609.1167.
R_f = 0.27 (10% EtOAc/hexane).

((1-(6-(4-bromophenyl)-5-(chloromethyl)-2,2-dimethyl-1,3-dioxan-4-yl)-1-(4-fluorophenyl)-3-(*p*-tolyl)pent-4-en-2-yl)oxy)triethylsilane (7):

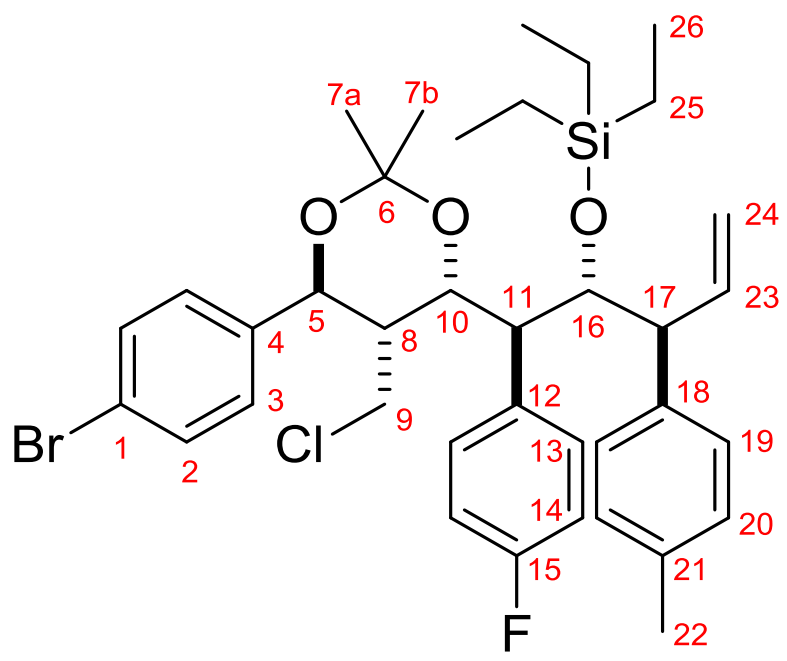

To a solution of **7** (44.1 mg, 0.075 mmol, 1 equiv.) in anhydrous CH_2Cl_2 (1 mL) at 0 °C was added DIPEA (0.04 mL, 0.150 mmol, 2 equiv.) then triethylsilyl trifluoromethanesulfonate (0.04 mL, 0.225 mmol, 1.5 equiv.). The mixture was warmed to r.t. and stirred for 30 min, then partitioned between EtOAc (15 mL) and sat. aq. $NaHCO_3$ solution (15 mL). The organic layer was separated and the aqueous layer extracted further with EtOAc (3 × 10 mL). The combined organic extracts were dried ($MgSO_4$) and evaporated under reduced pressure. The residue was purified by silica gel column chromatography (eluent: 5% EtOAc/hexane) to provide the title product **7** (50.8 mg, 0.072 mmol, 96%) as a white foam.

^{1}H NMR (600 MHz, $CDCl_3$): δ 7.46 (d, *J* = 8.4 Hz, 2 H, H2), 7.28 (d, *J* = 8.4 Hz, 2 H, H3), 7.19 (d, *J* = 7.8 Hz, 2 H, H19), 7.11 (d, *J* = 7.8 Hz, 2 H, H20), 6.93 (t, *J* = 8.6 Hz, 2 H, H14),

6.88 (br s, 2 H, H13), 5.97 (ddd, J = 16.8, 9.7, 9.4 Hz, 1 H, H23), 5.00 – 4.95 (m, 3 H, H10, $H24_{cis}$ and $H24_{trans}$), 4.84 (d, J = 6.8 Hz, 1 H, H5), 4.07 (t, J = 9.4 Hz, 1 H, H17), 4.00 (d, J = 9.4 Hz, 1 H, H16), 3.28 (dd, J = 11.5, 7.9 Hz, 1 H, $H9_a$), 3.02 (dd, J = 11.5, 3.5 Hz, 1 H, $H9_b$), 2.93 (d, J = 11.4 Hz, 1 H, H11), 2.38 (s, 3 H, H22), 2.08 – 2.03 (m, 1 H, H8), 1.60 (s, 3 H, $H7_a/H7_b$), 1.59 (s, 3 H, $H7_a/H7_b$), 0.89 (t, J = 8.0 Hz, 9 H, H26), 0.50 (q, J = 8.0 Hz, 6 H, H25).
^{13}C NMR (150 MHz, $CDCl_3$): δ 161.7 (d, J = 245.8 Hz, C15), 141.3 (C1), 140.9 (C23), 140.1 (C18), 137.0 – 136.8 (br, C12), 136.1 (C21), 131.7 (C2), 130.2 – 129.2 (br, C13), 129.5 (C19), 129.0 (C3), 128.9 (C20), 121.8 (C4), 116.4 (C24), 115.6 (br d, J = 20.7 Hz, C14), 101.7 (C6), 82.7 – 82.4 (br, C16), 73.1 (C5), 67.1 (C10), 56.1 (C17), 45.7 (C8), 45.8 – 45.5 (br, C11), 43.0 (C9), 25.6 ($C7_a/C7_b$), 24.8 ($C7_a/C7_b$), 21.3 (C22), 7.2 (C26), 5.9 (C25).
^{19}F NMR (376 MHz, $CDCl_3$): δ -115.5 (s, 1 F, F15).
FTIR (ν_{max}, cm^{-1}): 2953 (m), 2877 (m), 1607 (w, C=C), 1510 (s), 1490 (w), 1458 (w), 1411 (w), 1380 (m), 1222 (s), 1160 (m), 1113 (s), 1089 (s), 1056 (m), 1010 (s), 975 (w), 911 (w), 832 (w), 777 (w).
HRMS (ESI): calculated for $C_{37}H_{46}BrClFO_2Si$ $[M+H]^+$ 701.2150, found 701.2129.
R_f = 0.40 (5% EtOAc/hexane).

7-(6-(4-bromophenyl)-5-(chloromethyl)-2,2-dimethyl-1,3-dioxan-4-yl)-7-(4-fluorophenyl)-3-phenyl-5-(*p*-tolyl)-6-((triethylsilyl)oxy)hept-1-en-4-ol (8):

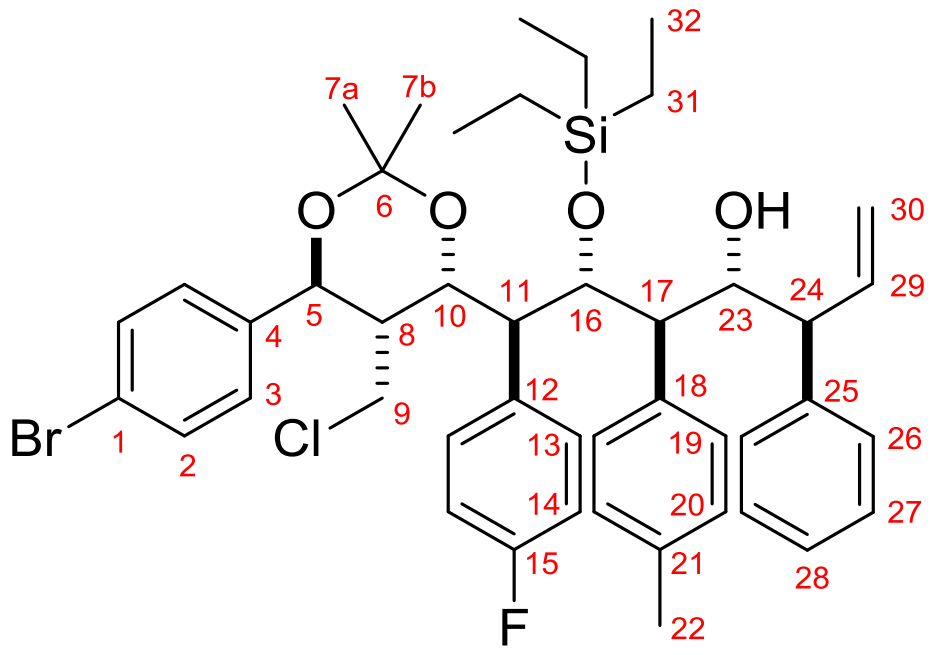

To a solution of **7** (50.8 mg, 0.072 mmol) in CH_2Cl_2/MeOH (9:1, 15 mL) was bubbled O_3/O_2 at -78 °C until the reaction mixture turned blue (ca. 3 min). Argon was then bubbled through the solution until residual O_3 had disappeared. Polymer-supported PPh_3 (3 mmol/g loading, 72 mg, ca. 0.21 mmol) was then added and the mixture stirred at r.t. for 1 h. The polymer-supported reagent was then filtered off and the filtrate evaporated under reduced pressure to provide the crude aldehyde, which was used immediately without further purification.

To a solution of the crude aldehyde and *trans*-2-phenylvinylboronic acid (16.0 mg, 0.108 mmol, 2 equiv.) in CH_2Cl_2/THF (4:1, 1 mL) was added (trimethylsilyl)diazomethane (0.07 mL, 2 M in hexanes, 0.144 mmol, 2.0 equiv.) and the mixture subsequently stirred for 2 h. The mixture was quenched with SiO_2 and stirred for a further 10 min, filtered and evaporated under reduced pressure. The residue was purified by silica gel column chromatography (eluent: 10% EtOAc/hexane) to provide the title product **8** (50.8 mg, 0.062 mmol, 86% over 2 steps) as a white foam.

^{1}H NMR (600 MHz, $CDCl_3$): δ 7.42 (d, J = 8.4 Hz, 2 H, H2), 7.34 (t, J = 7.4 Hz, 2 H, H27), 7.27 (t, J = 7.4 Hz, 1 H, H28), 7.20 (d, J = 8.4 Hz, 2 H, H3), 7.06 (d, J = 7.4 Hz, 2 H, H26), 6.90 (t, J = 8.6 Hz, 2 H, H14), 6.78 (br s, 2 H, H13), 5.93 (dt, J = 17.0, 10.0 Hz, 1 H, H29), 5.11 (dd, J = 10.1, 1.1 Hz, 1 H, $H30_{cis}$), 5.05 (d, J = 17.0 Hz, 1 H, $H30_{trans}$), 4.84 – 4.79 (m, 1 H, H10), 4.63 (d, J = 6.9 Hz, 1 H, H5), 4.31 (d, J = 10.0 Hz, 1 H, H23), 4.18 (d, J = 10.7 Hz, 1 H, H16), 3.30 (br d, J = 10.7 Hz, 1 H, H17), 3.08 (dd, J = 11.4, 8.2 Hz, 1 H, $H9_a$), 2.85 (dd,

J = 11.4, 3.5 Hz, 1 H, H9$_b$), 2.80 (t, J = 10.0 Hz, 1 H, H24), 2.63 (d, J = 11.4 Hz, 1 H, H11), 2.43 (s, 3 H, H22), 1.99 – 1.94 (m, 1 H, H8), 1.78 (s, 1 H, OH), 1.18 (s, 3 H, H7$_a$/H7$_b$), 1.05 (s, 3 H, H7$_a$/H7$_b$), 0.95 (t, J = 8.0 Hz, 9 H, H32), 0.61 – 0.48 (m, 6 H, H31). (N.B. 4 H corresponding to H19/H20 appear broadened over 7.60 – 7.10 region).
^{13}C NMR (150 MHz, CDCl$_3$): δ 161.6 (d, J = 245.4 Hz, C15), 141.4 (C1), 141.2 (C25), 140.5 (C29), 137.5 (br, C12), 136.3 (C18/C21), 136.2 (C18/C21), 131.7 (C2), 130.2 – 128.5 (br, C13, C19 and C20), 129.1 (C3), 128.8 (C27), 128.6 (C26), 126.9 (C28), 121.7 (C4), 118.0 (C30), 115.5 (br, C14), 101.5 (C6), 80.7 (C16), 73.1 (C5), 71.5 (C23), 66.8 (C10), 56.3 (C24), 50.9 (C17), 45.7 (C8), 44.8 (br, C11), 43.0 (C9), 24.8 (C7$_a$/C7$_b$), 24.0 (C7$_a$/C7$_b$), 21.4 (C22), 7.4 (C32), 5.7 (C31).
^{19}F NMR (376 MHz, CDCl$_3$): δ -115.8 (s, 1 F, F15).
FTIR (ν_{max}, cm^{-1}): 3569 (w, OH), 2954 (w), 2876 (w), 1606 (w, C=C), 1510 (s), 1490 (m), 1454 (w), 1417 (w), 1380 (m), 1222 (s), 1160 (m), 1102 (s), 1074 (s), 1056 (m), 1009 (s), 961 (w), 908 (m), 833 (s), 803 (m), 778 (w).
HRMS (ESI): calculated for $C_{45}H_{56}BrClFO_4Si$ $[M+H]^+$ 821.2798, found 821.2804.
R_f = 0.59 (10% EtOAc/hexane).

Confirming relative stereochemistry of 2nd/3rd iteration products:

Analysis of the ^{1}H/^{13}C shifts of the acetonide for the 2nd iteration product **6** confirms a twist-boat structure, indicating that the reaction proceeds through Felkin-Anh selectivity.

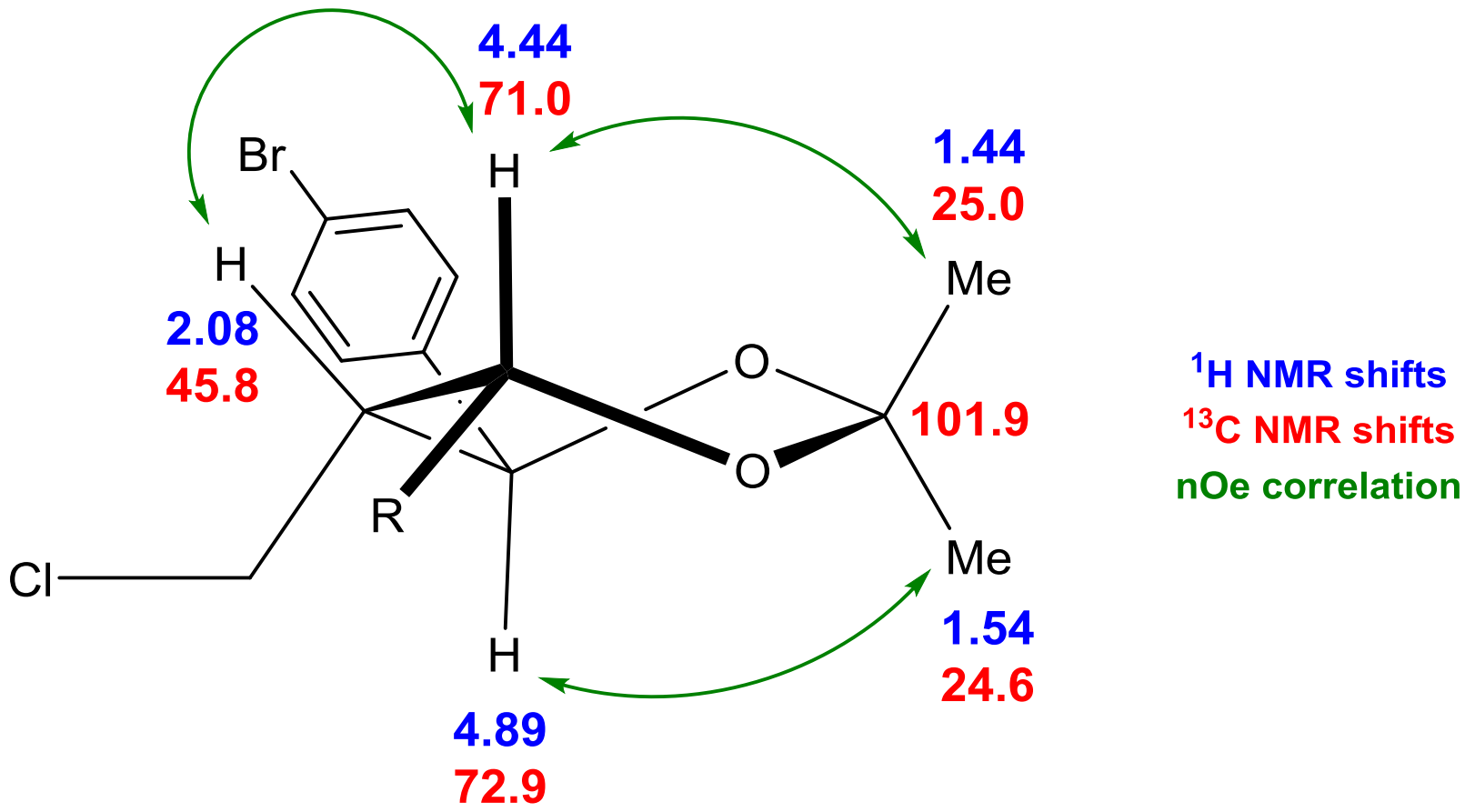

Deprotection of the TES protecting group with TBAF allowed a spontaneous 6-ring cyclisation to the corresponding pyran. Analysis of the coupling constants once again confirms Felkin-Anh selectivity.

4-(4-bromophenyl)-8-(4-fluorophenyl)-2,2-dimethyl-7-((1-(*p*-tolyl)allyl)tetrahydro-4*H*,5*H*-pyrano[4,3-*d*][1,3]dioxine (9):

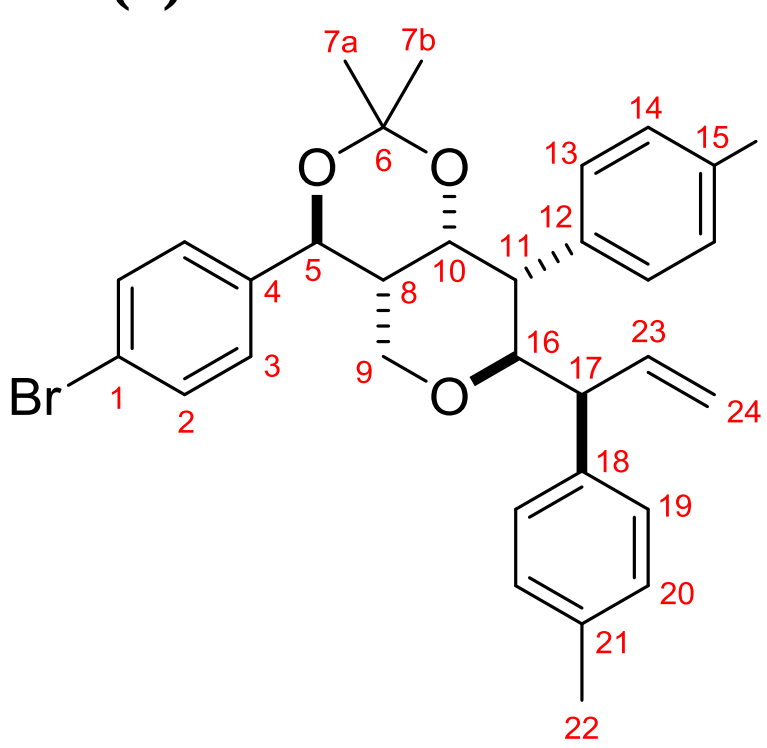

To a solution of **7** (0.250 g, 0.356 mmol, 1 equiv.) in anhydrous THF (8.5 mL) at 0 °C was added a solution of TBAF (1.42 mL, 1.0 M in THF, 1.42 mmol, 4 equiv.). The mixture was then stirred at r.t. for 16 h. The mixture was diluted with brine (5 mL) and EtOAc (5 mL) and the organic layer separated. The aqueous layer was extracted further with EtOAc (3 × 5 mL) and the combined organic extracts dried ($MgSO_4$) and evaporated under reduced pressure. The residue was purified by silica gel column chromatography (50% CH_2Cl_2/hexane) to provide the title product **9** (0.194 g, 0.352 mmol, 99%) as a white foam.

^{1}H NMR (600 MHz, $CDCl_3$): δ 7.51 (d, *J* = 8.4 Hz, 2 H, H2), 7.29 (br dd, *J* = 7.5, 5.8 Hz, 2 H, H13), 7.24 (d, *J* = 8.4 Hz, 2 H, H3), 7.18 (d, *J* = 8.0 Hz, 2 H, H19), 7.13 (d, *J* = 8.0 Hz, 2 H, H20), 7.06 (appears t, *J* = 8.7 Hz, 2 H, H14), 6.16 (dt, *J* = 17.2, 10.0 Hz, 1 H, H23), 5.16 (dd, *J* = 10.0, 1.7 Hz, 1 H, $H24_{cis}$), 4.84 (dd, *J* = 17.2, 1.7 Hz, 1 H, $H24_{trans}$), 4.27 (d, *J* = 11.0 Hz, 1 H, H16), 4.24 (d, *J* = 6.1 Hz, 1 H, H5), 4.17 (t, *J* = 3.1 Hz, 1 H, H10), 4.01 (dd, *J* = 11.2, 5.9 Hz, 1 H, $H9_a$), 3.93 (t, *J* = 11.2 Hz, 1 H, $H9_b$), 3.09 (d, *J* = 9.6 Hz, 1 H, H17), 2.97 (dd, *J* = 11.0, 3.1 Hz, 1 H, H11), 2.36 (s, 3 H, H22), 2.27 – 2.20 (m, 1 H, H8), 1.44 (s, 3 H, $H7_a/H7_b$), 1.09 (s, 3 H, $H7_a/H7_b$).
^{13}C NMR (150 MHz, $CDCl_3$): δ 161.9 (d, *J* = 244.6 Hz, C15), 141.5 (C1), 140.4 (C18), 136.3 (C23), 135.9 (C21), 135.1 (d, *J* = 3.2 Hz, C12), 131.8 (C2), 131.3 (d, *J* = 7.6 Hz, C13), 129.1 (C20), 128.0 (C19), 127.5 (C3), 121.5 (C4), 117.7 (C24), 114.9 (d, *J* = 20.9 Hz, C14), 101.5 (C6), 78.6 (C16), 71.6 (C5), 66.9 (C9), 66.7 (C10), 50.6 (C17), 47.5 (C11), 44.2 (C8), 25.0 ($C7_a/C7_b$), 24.1 ($C7_a/C7_b$), 21.1 (C22).
^{19}F NMR (376 MHz, $CDCl_3$): δ -116.1 (s, 1 F, F15).
FTIR (ν_{max}, cm^{-1}): 2985 (w), 2925 (w), 1636 (w, C=C), 1605 (w), 1510 (s), 1489 (m), 1456 (w), 1379 (m), 1283 (w), 1219 (s), 1159 (m), 1135 (m), 1112 (m), 1096 (m), 1073 (m), 1058 (m), 1004 (m), 982 (m), 957 (w), 941 (w), 908 (s), 857 (m), 830 (s), 790 (m).
HRMS (ESI): calculated for $C_{31}H_{33}BrFO_3$ $[M+H]^+$ 551.1592, found 551.1583.
R_f = 0.36 (50% CH_2Cl_2/hexane).

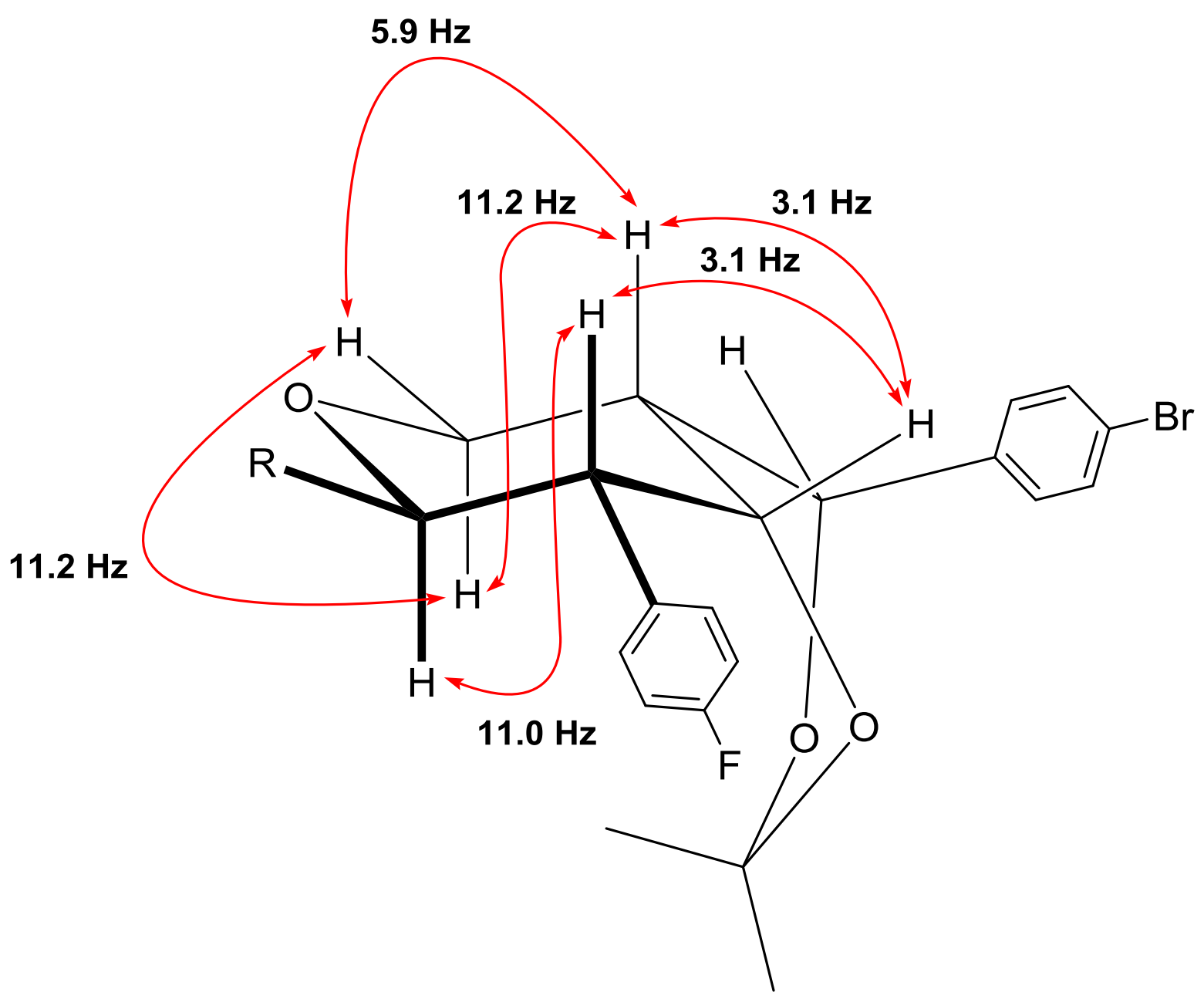

3. COSY ^{1}H-NMR studies

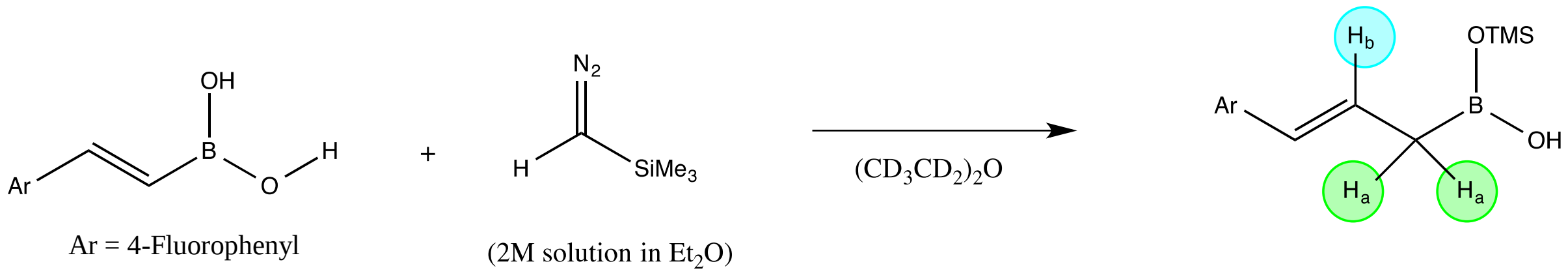

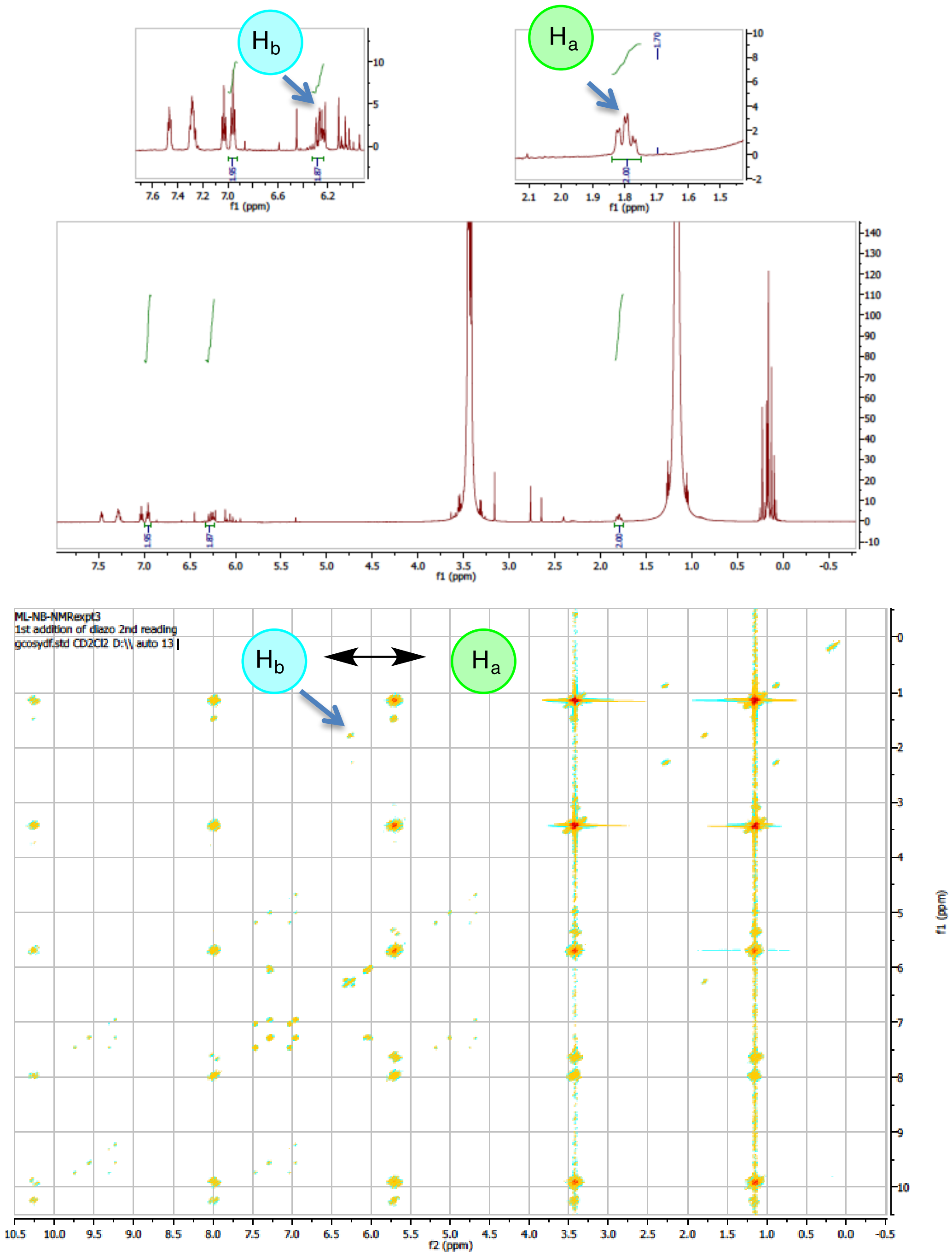

4. ^{1}H and ^{13}C NMR spectra of compound 11

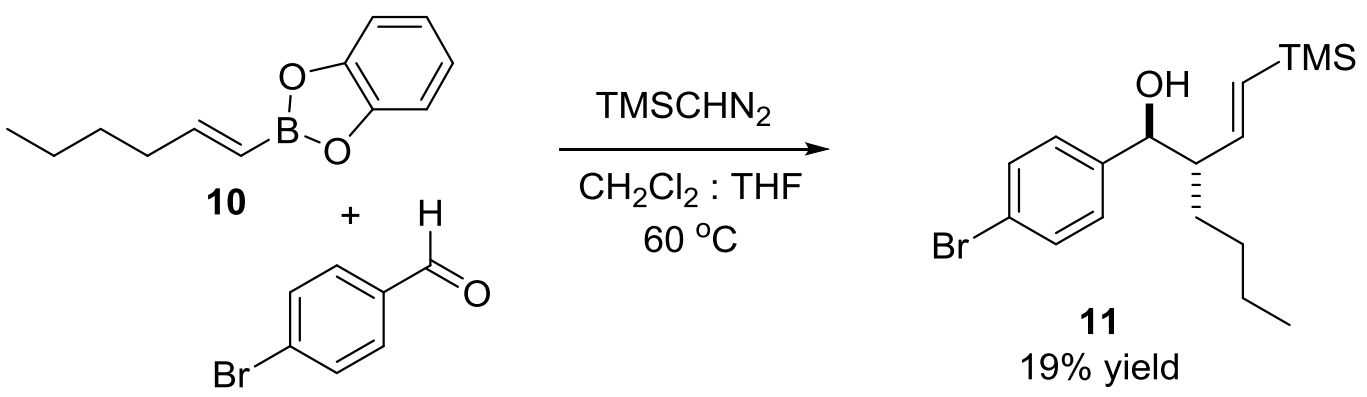

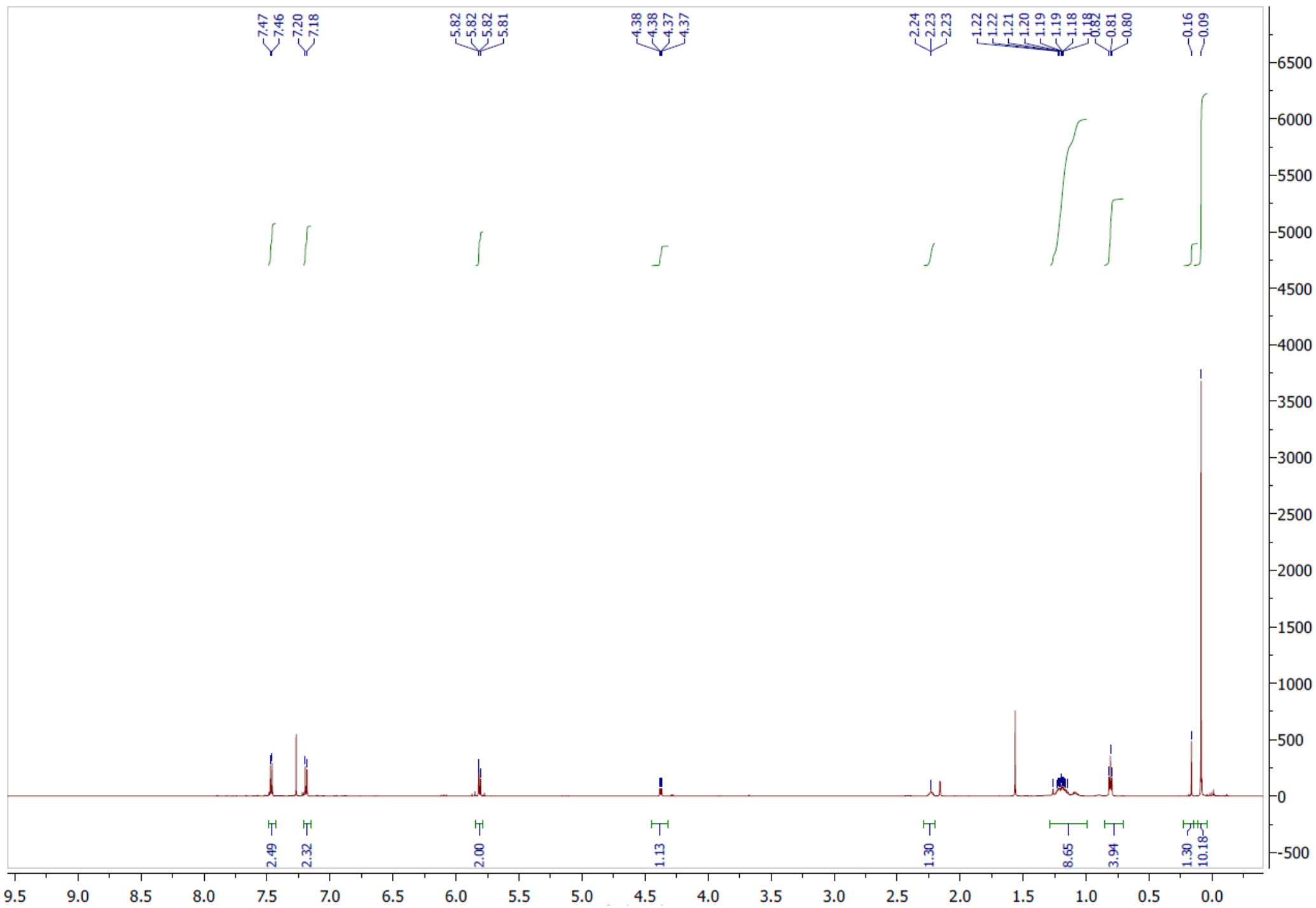

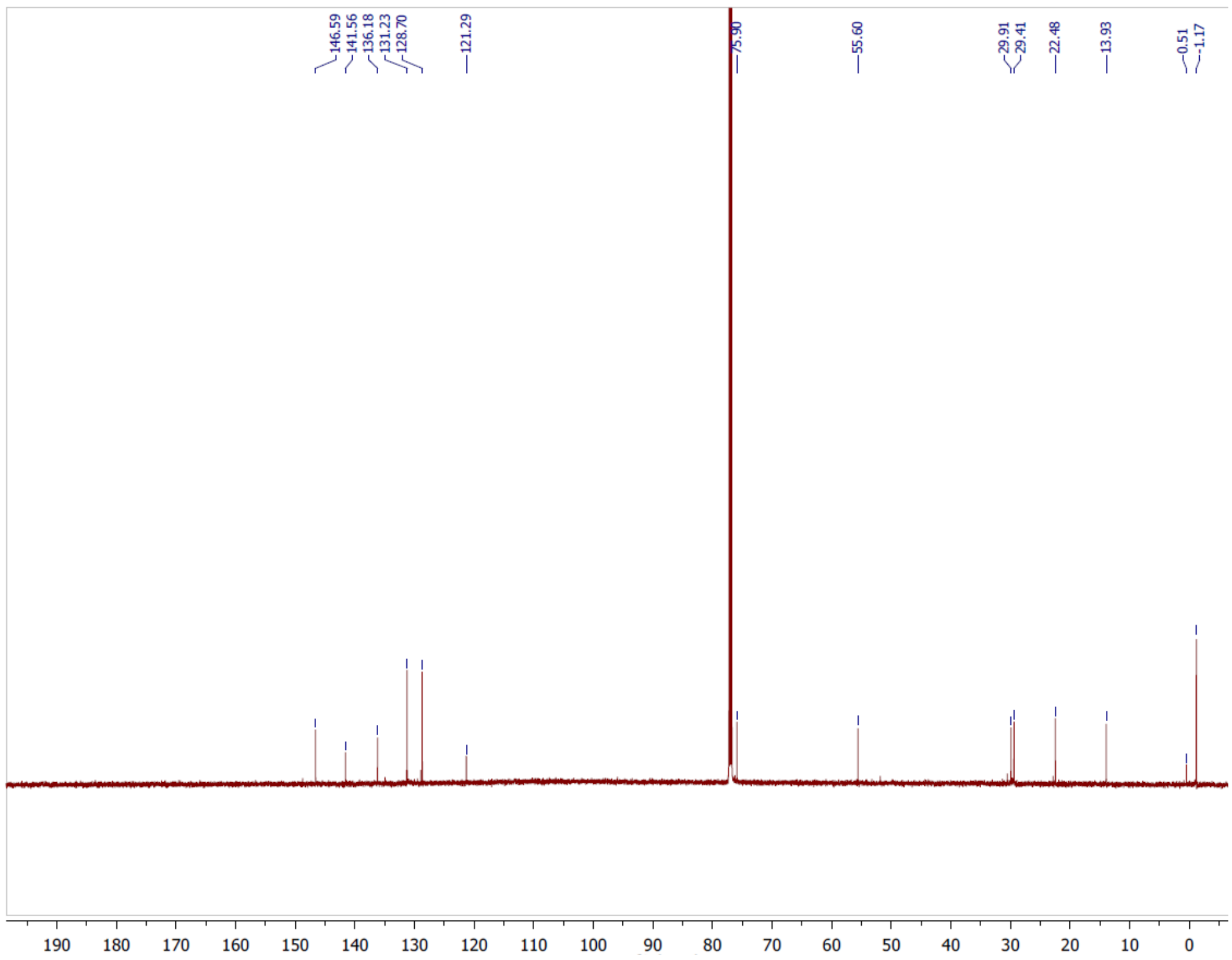

5. ^{1}H and ^{13}C-NMR spectra

1-(4-bromophenyl)-2-(*p*-tolyl)but-3-en-1-ol (4a):

^{1}H NMR, 600 MHz, $CDCl_3$:

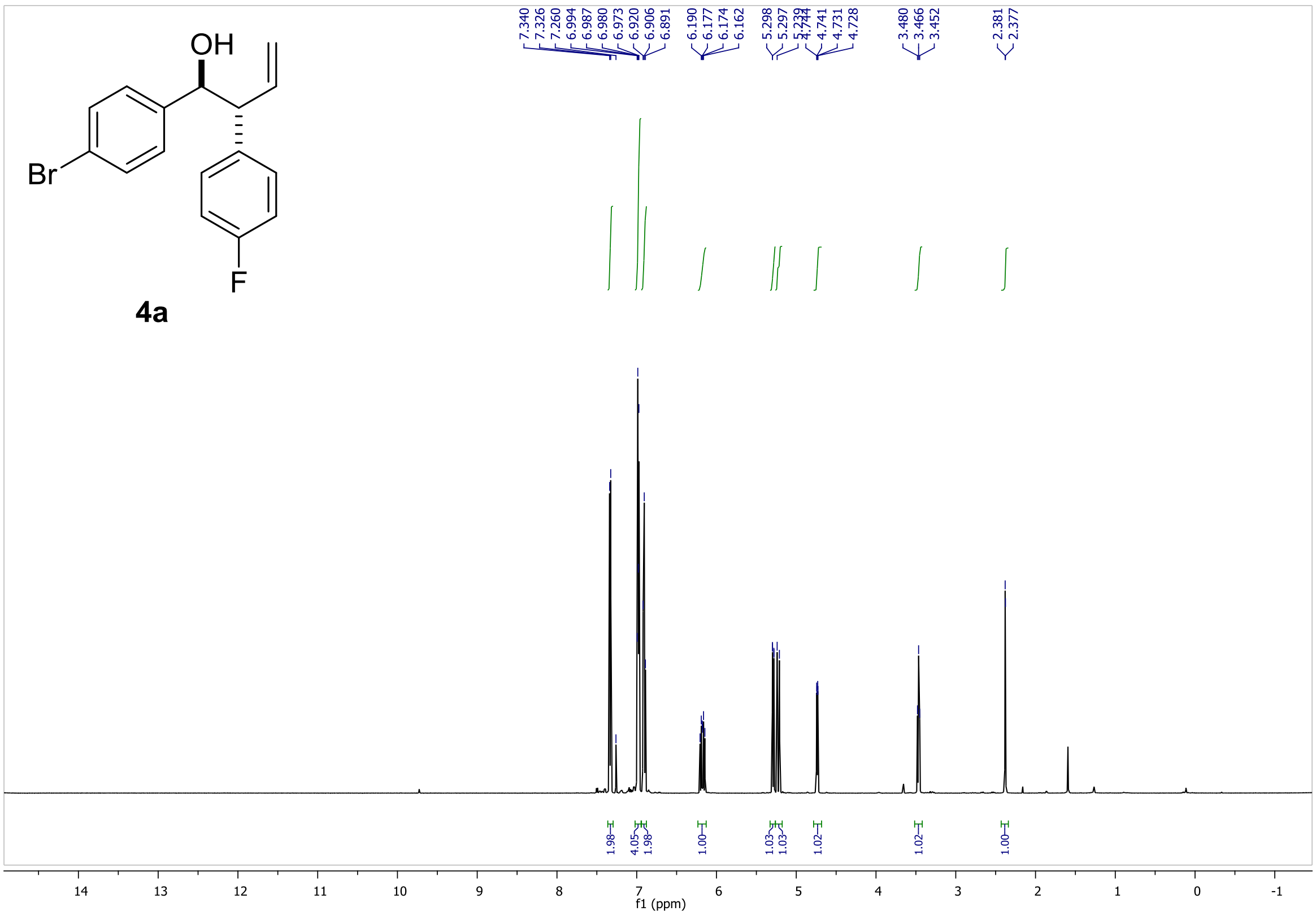

^{13}C NMR, 150 MHz, $CDCl_3$:

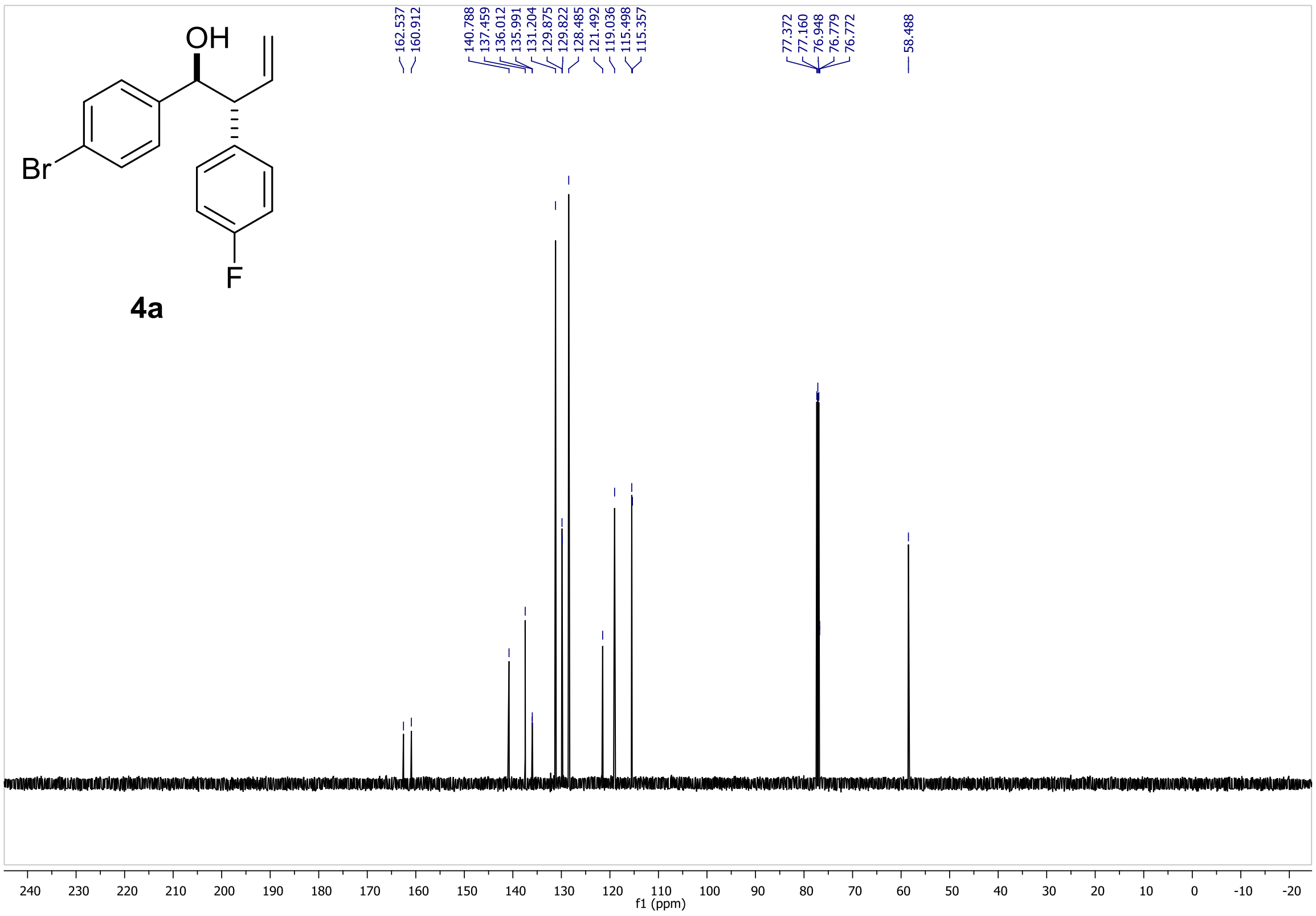

^{19}F NMR, 376 MHz, CDCl$_3$:

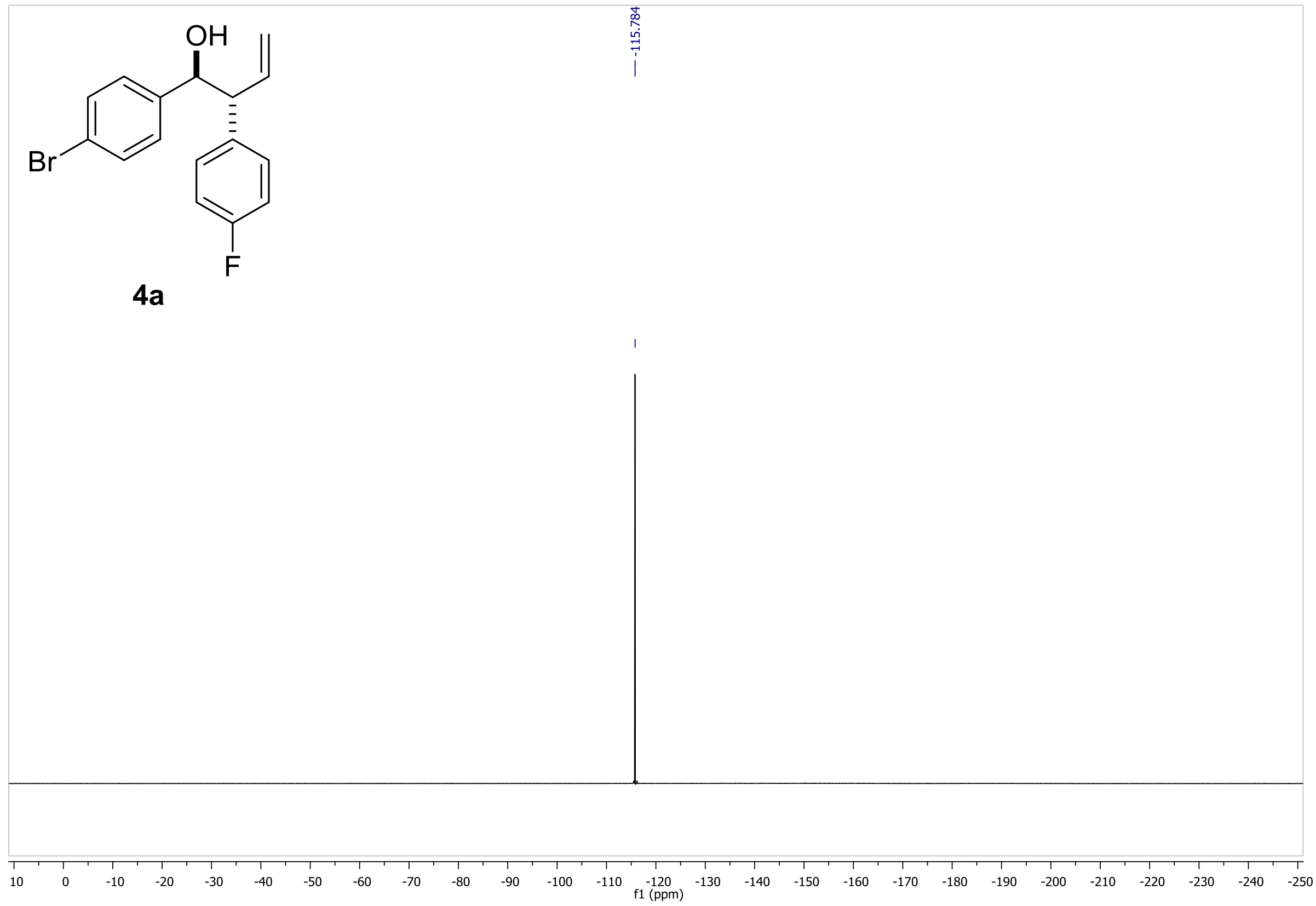

1-(4-bromophenyl)-2-(*p*-tolyl)but-3-en-1-ol (4b):

^{1}H NMR, 600 MHz, $CDCl_3$:

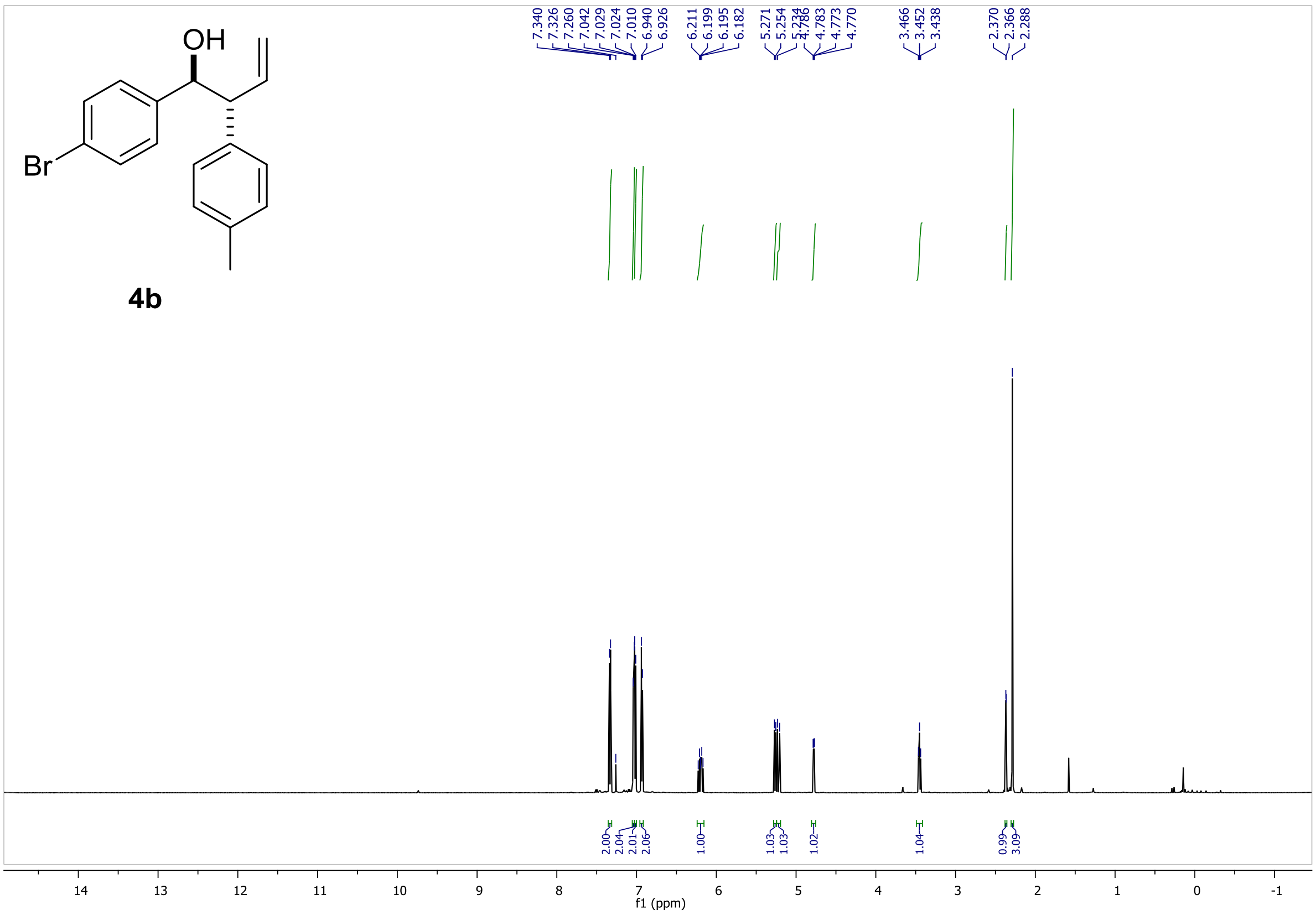

^{13}C NMR, 150 MHz, $CDCl_3$:

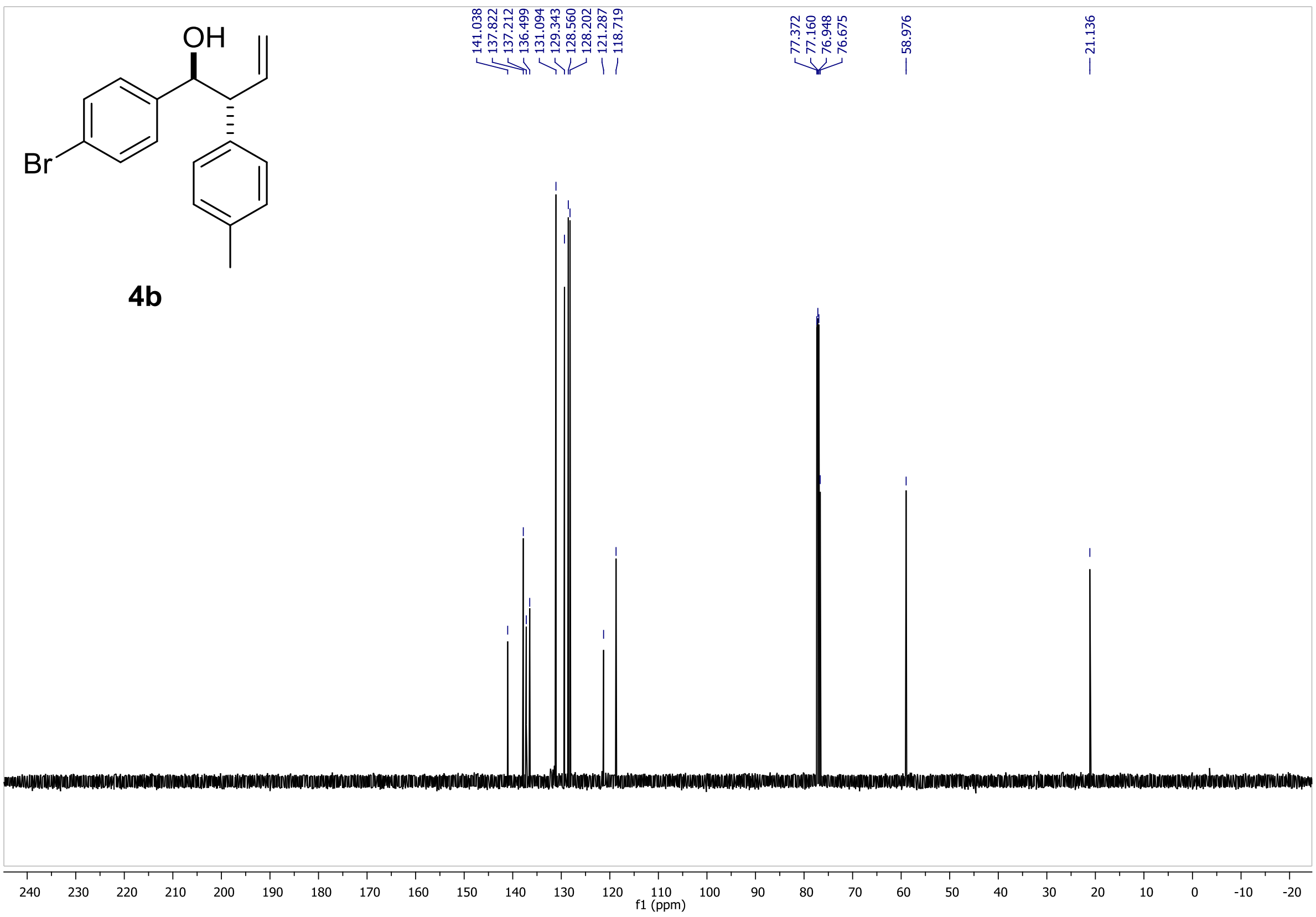

1-(4-bromophenyl)-2-(4-(trifluoromethyl)phenyl)but-3-en-1-ol (4c):

^{1}H NMR, 600 MHz, $CDCl_3$:

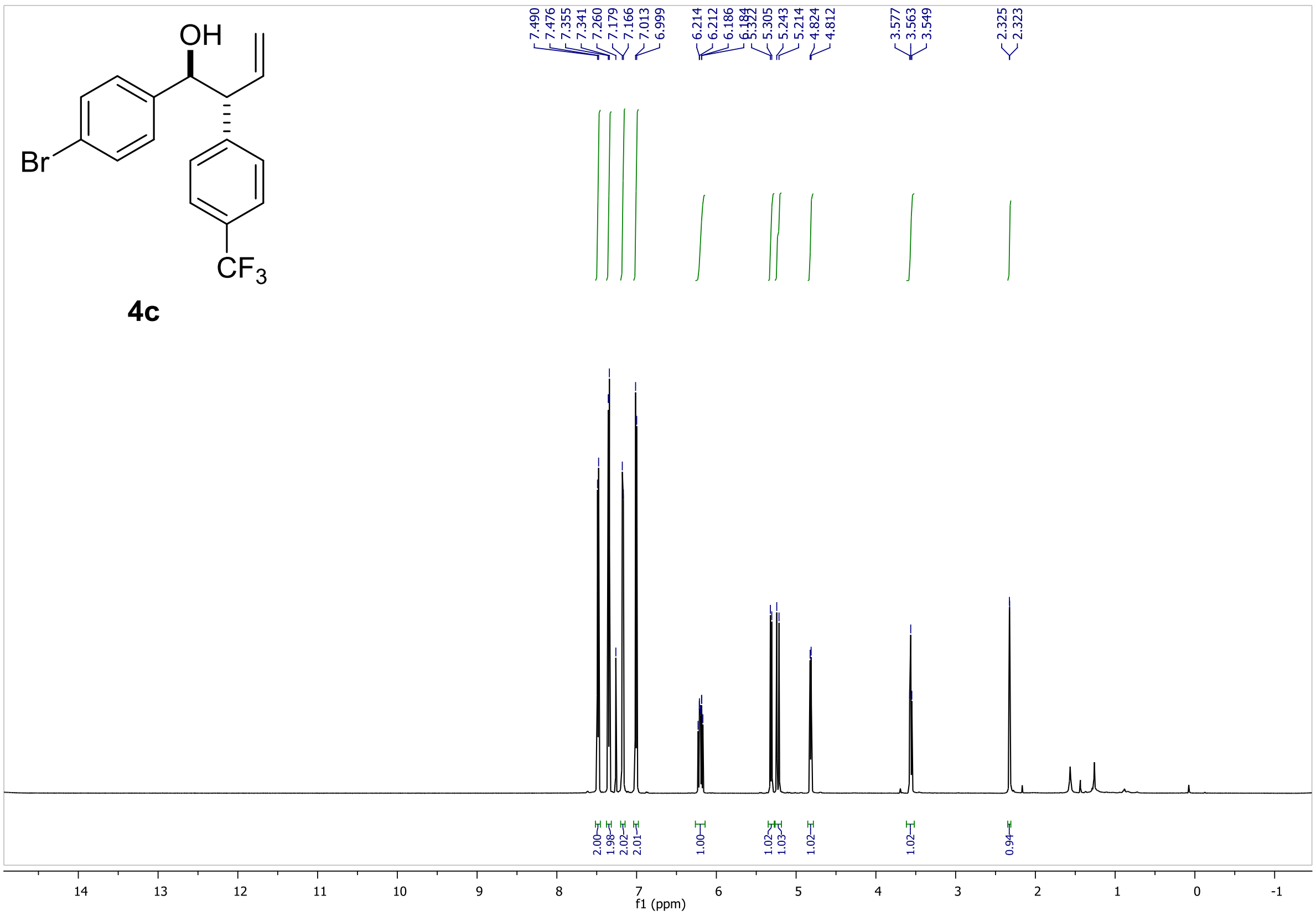

^{13}C NMR, 150 MHz, $CDCl_3$:

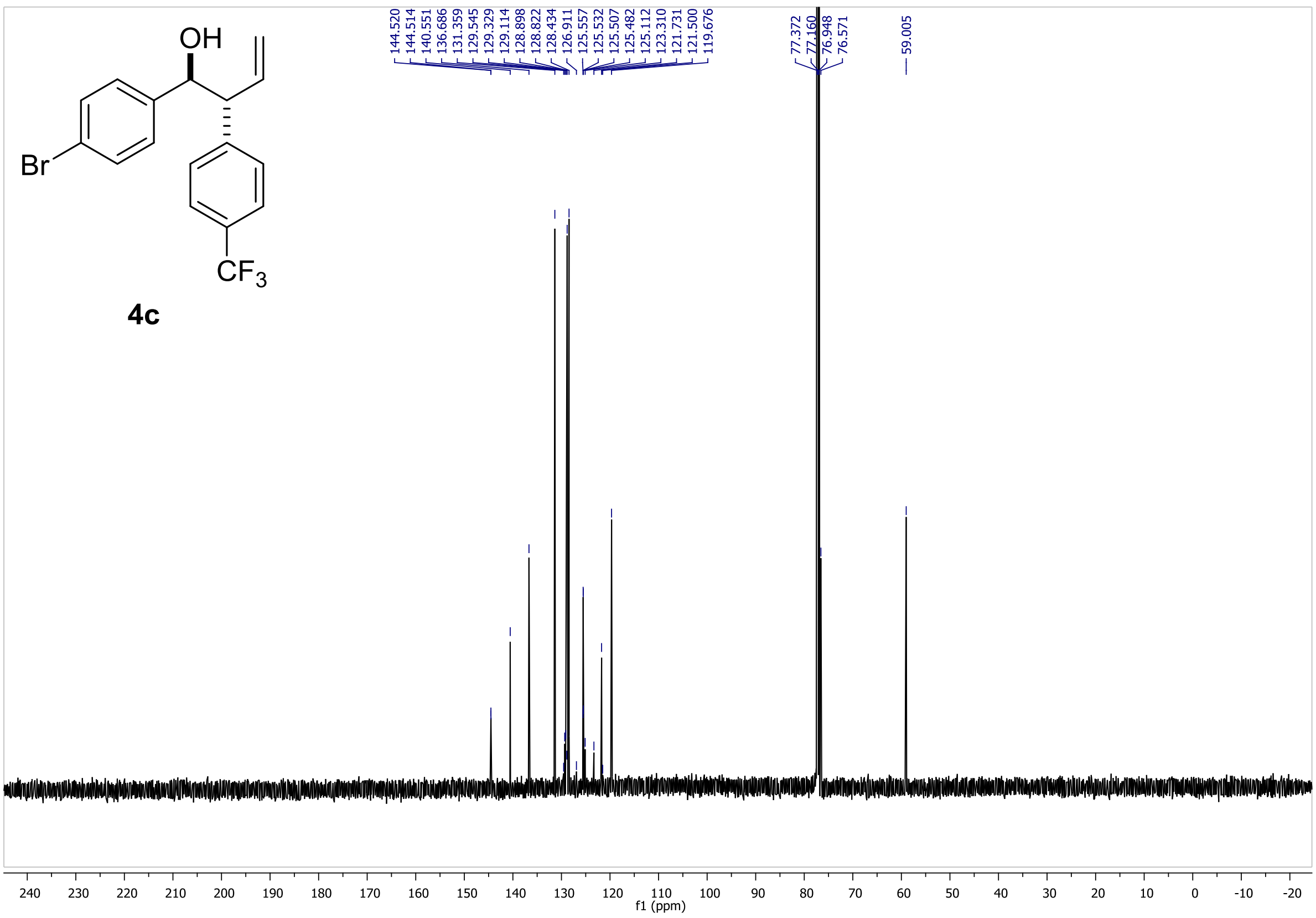

^{19}F NMR, 376 MHz, $CDCl_3$:

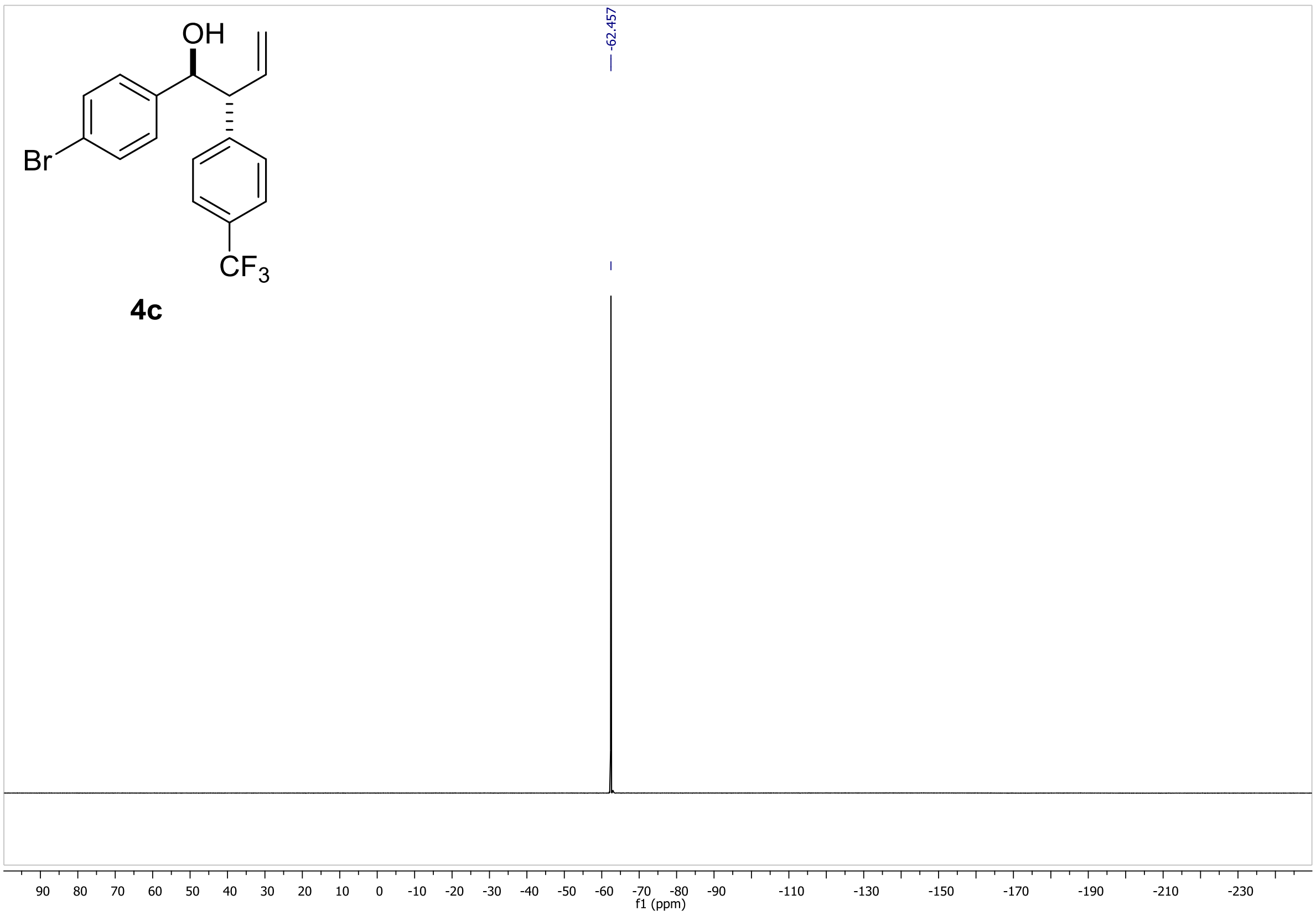

1-(pyridin-3-yl)-2-(4-(trifluoromethyl)phenyl)but-3-en-1-ol (4d):

^{1}H NMR, 600 MHz, $CDCl_3$:

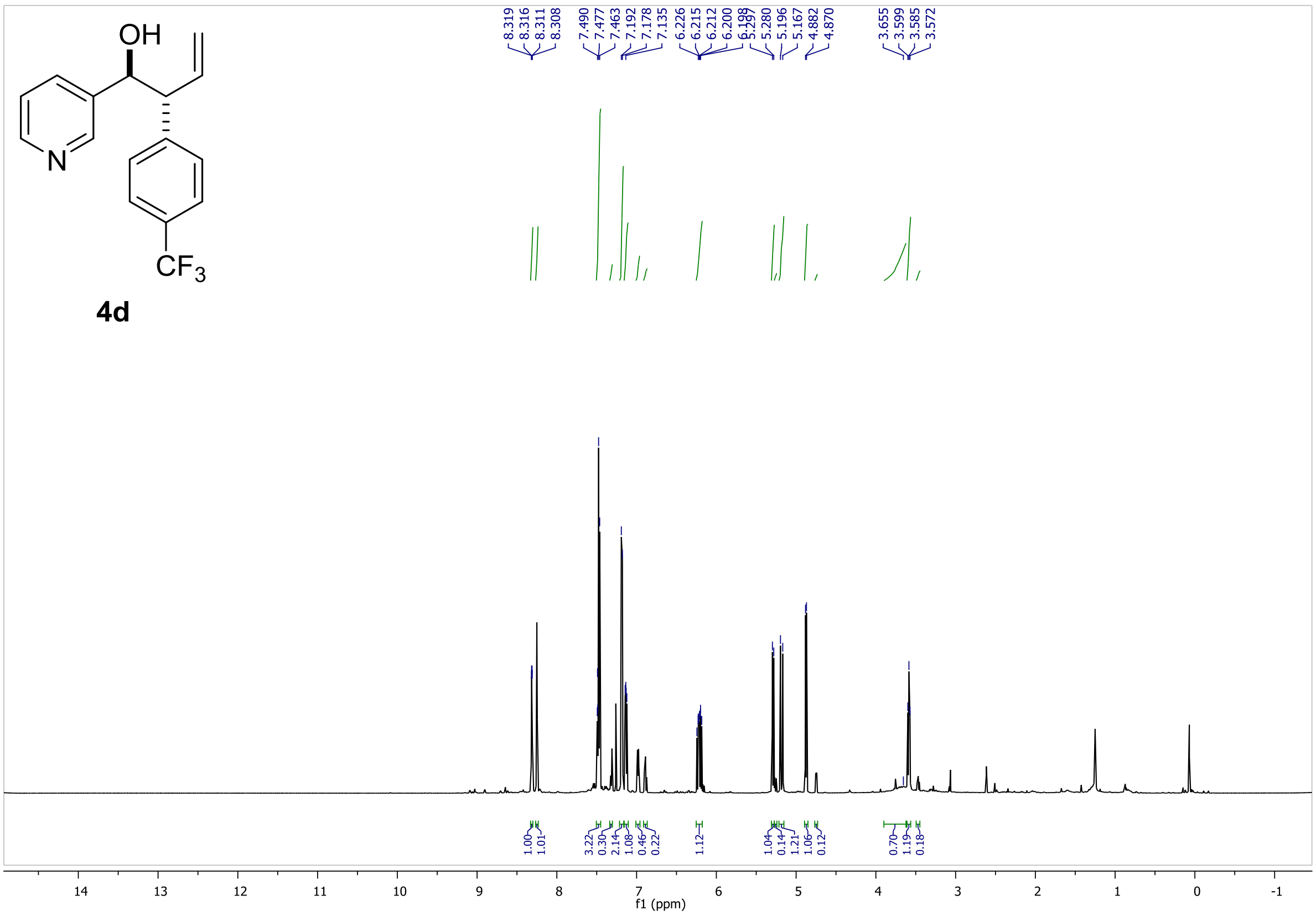

^{13}C NMR, 150 MHz, $CDCl_3$:

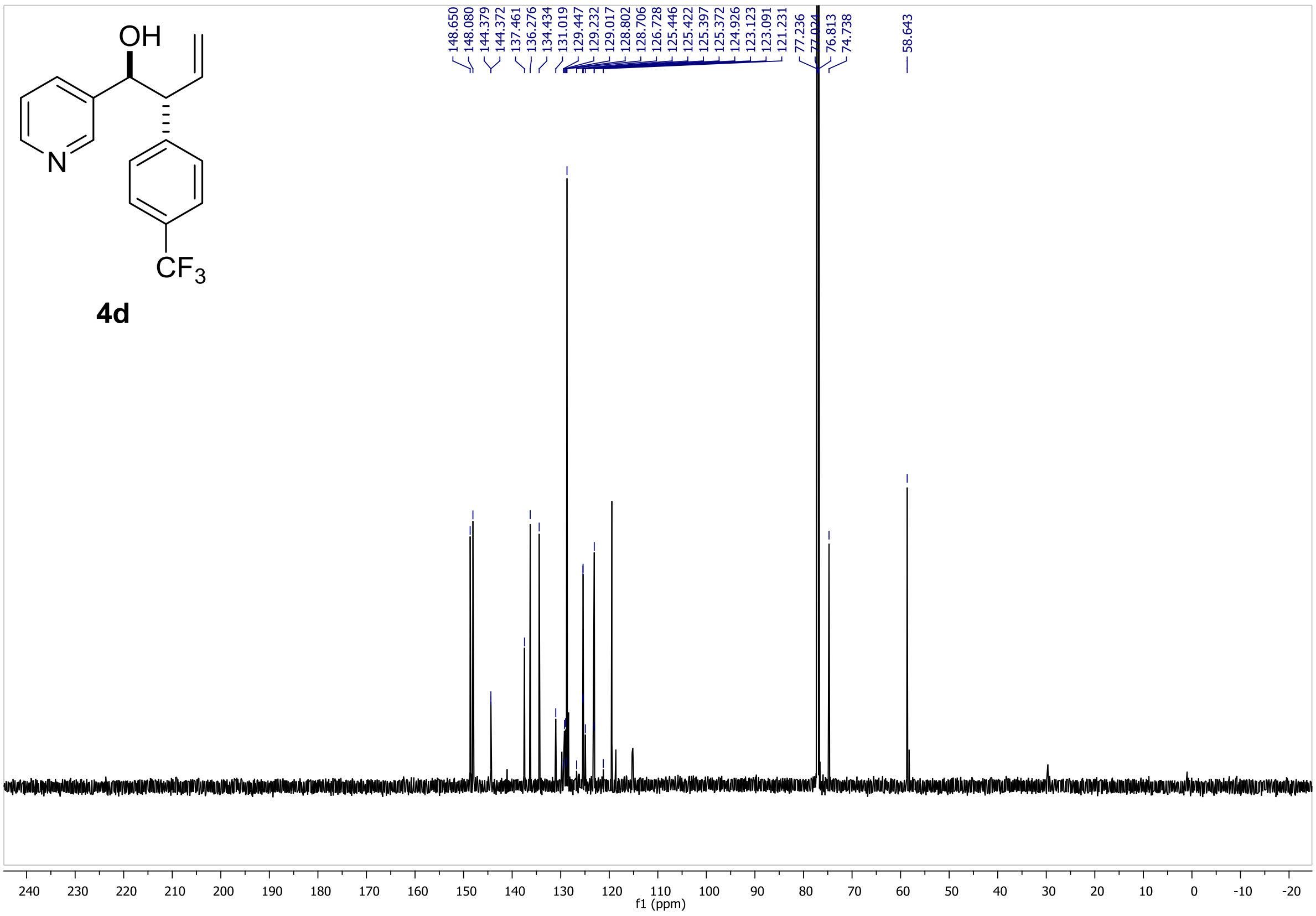

^{19}F NMR, 376 MHz, $CDCl_3$:

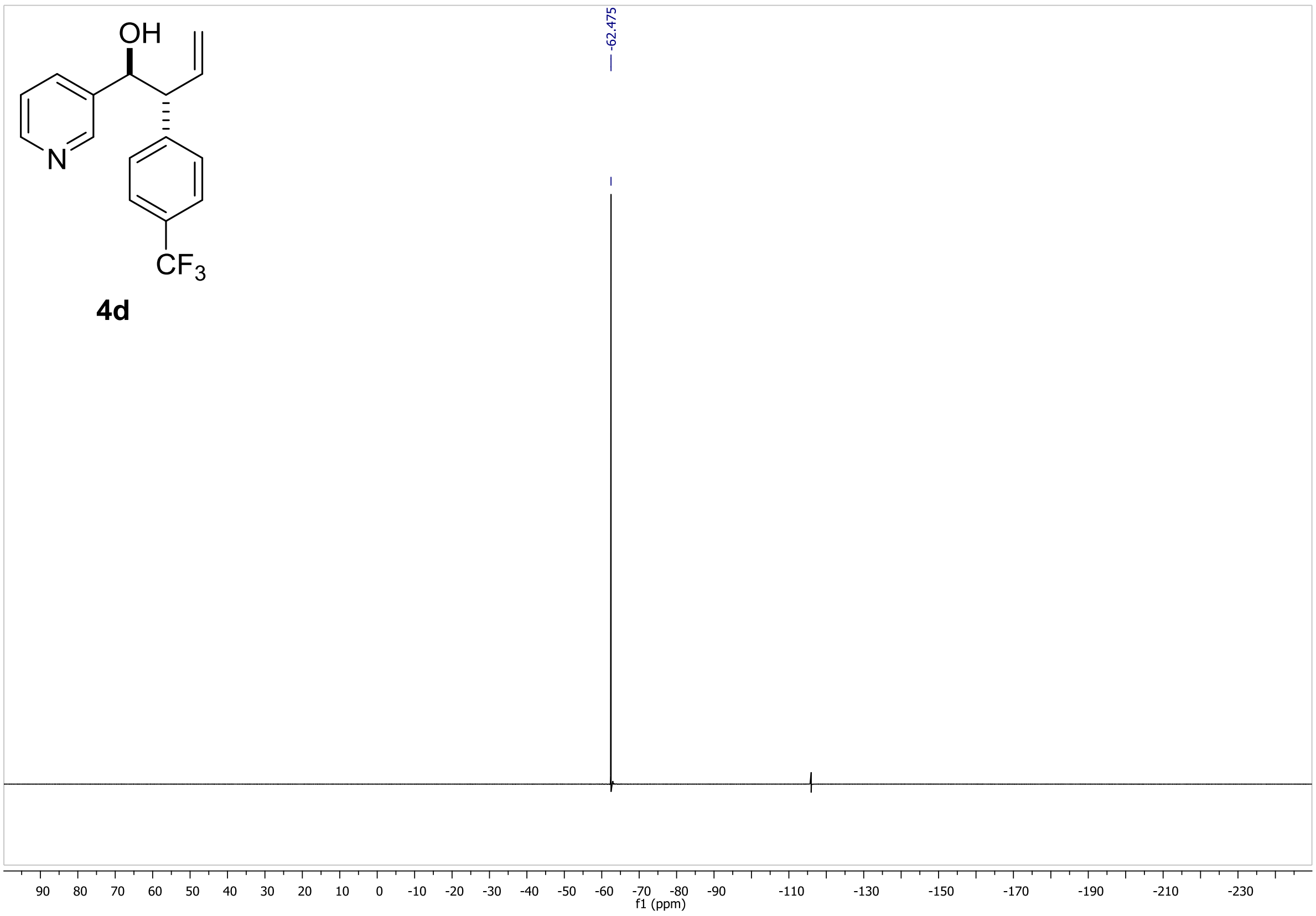

2-(4-fluorophenyl)-1-(pyridin-3-yl)but-3-en-1-ol (4e):

^{1}H NMR, 600 MHz, $CDCl_3$:

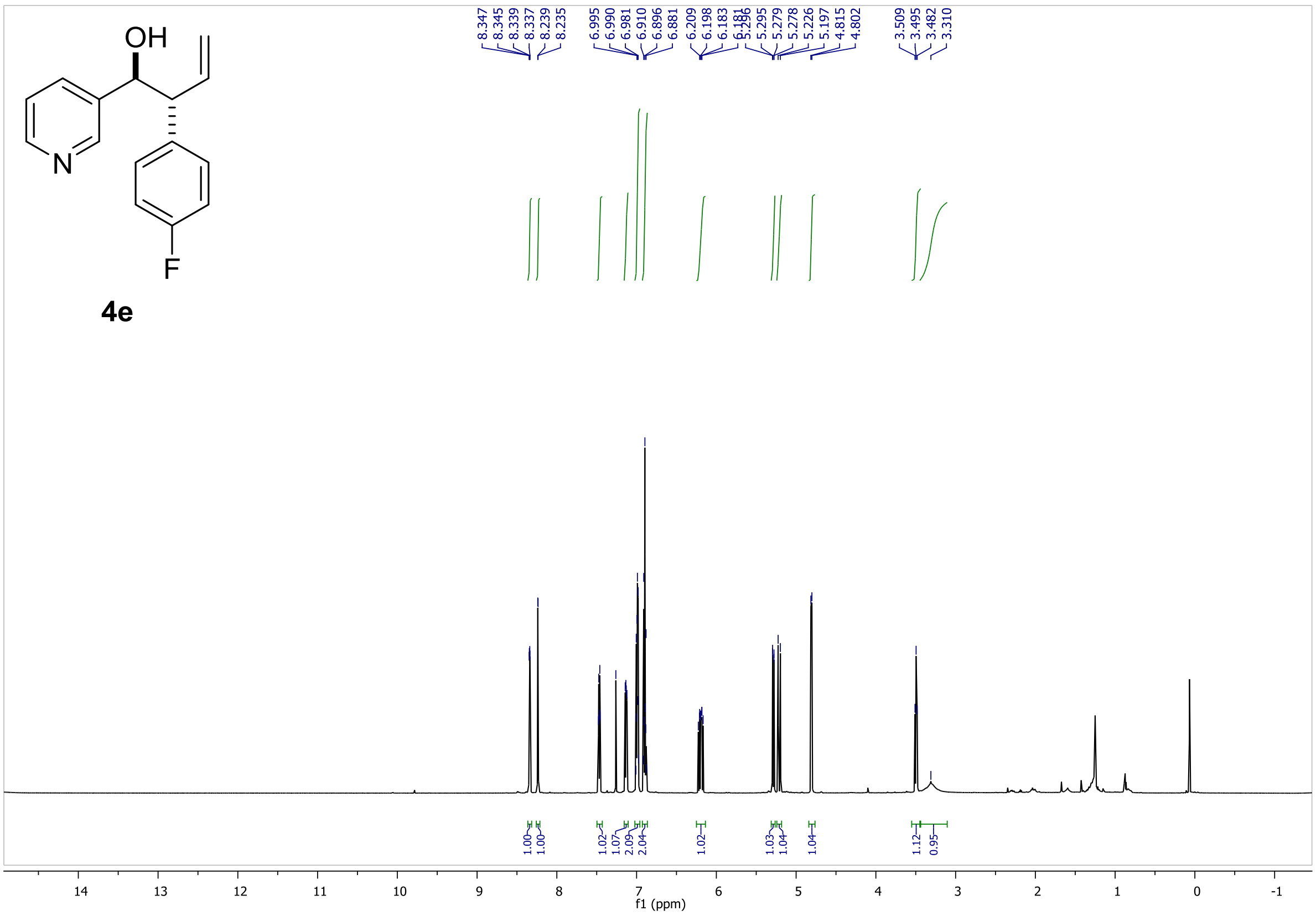

^{13}C NMR, 150 MHz, $CDCl_3$:

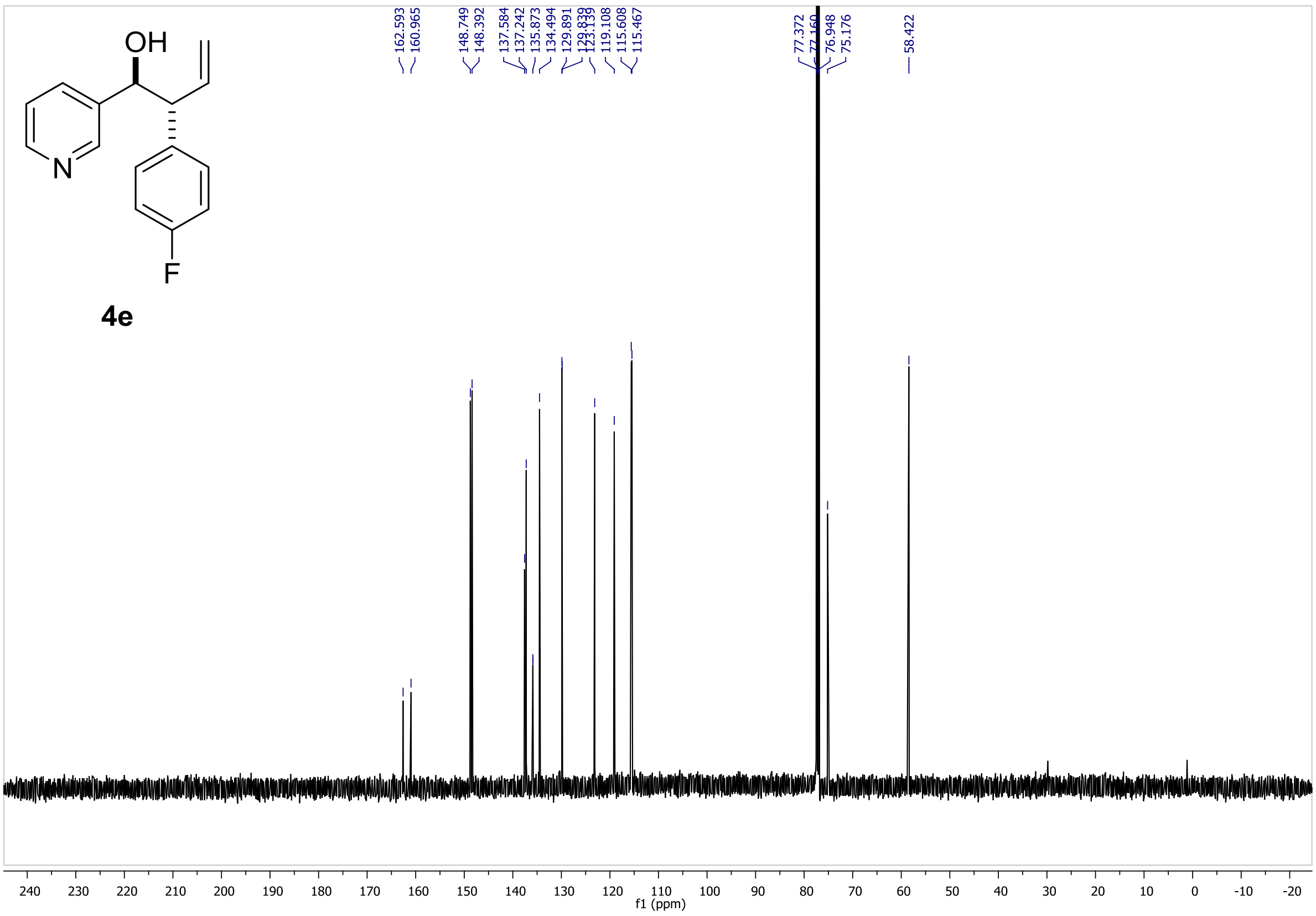

^{19}F NMR, 376 MHz, $CDCl_3$:

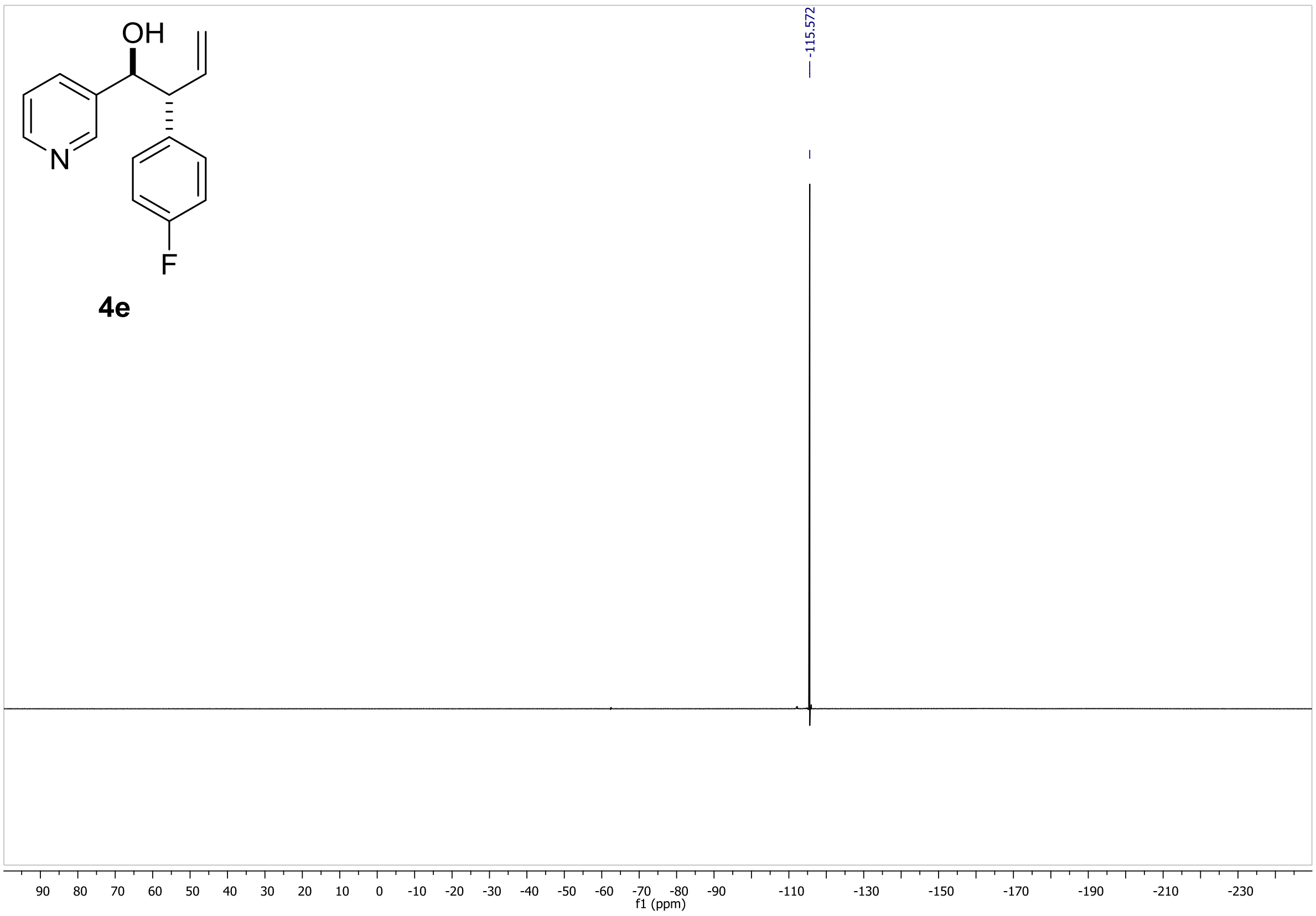

(*E*)-3-(4-(trifluoromethyl)phenyl)hepta-1,5-dien-4-ol (4f):

^{1}H NMR, 600 MHz, $CDCl_3$:

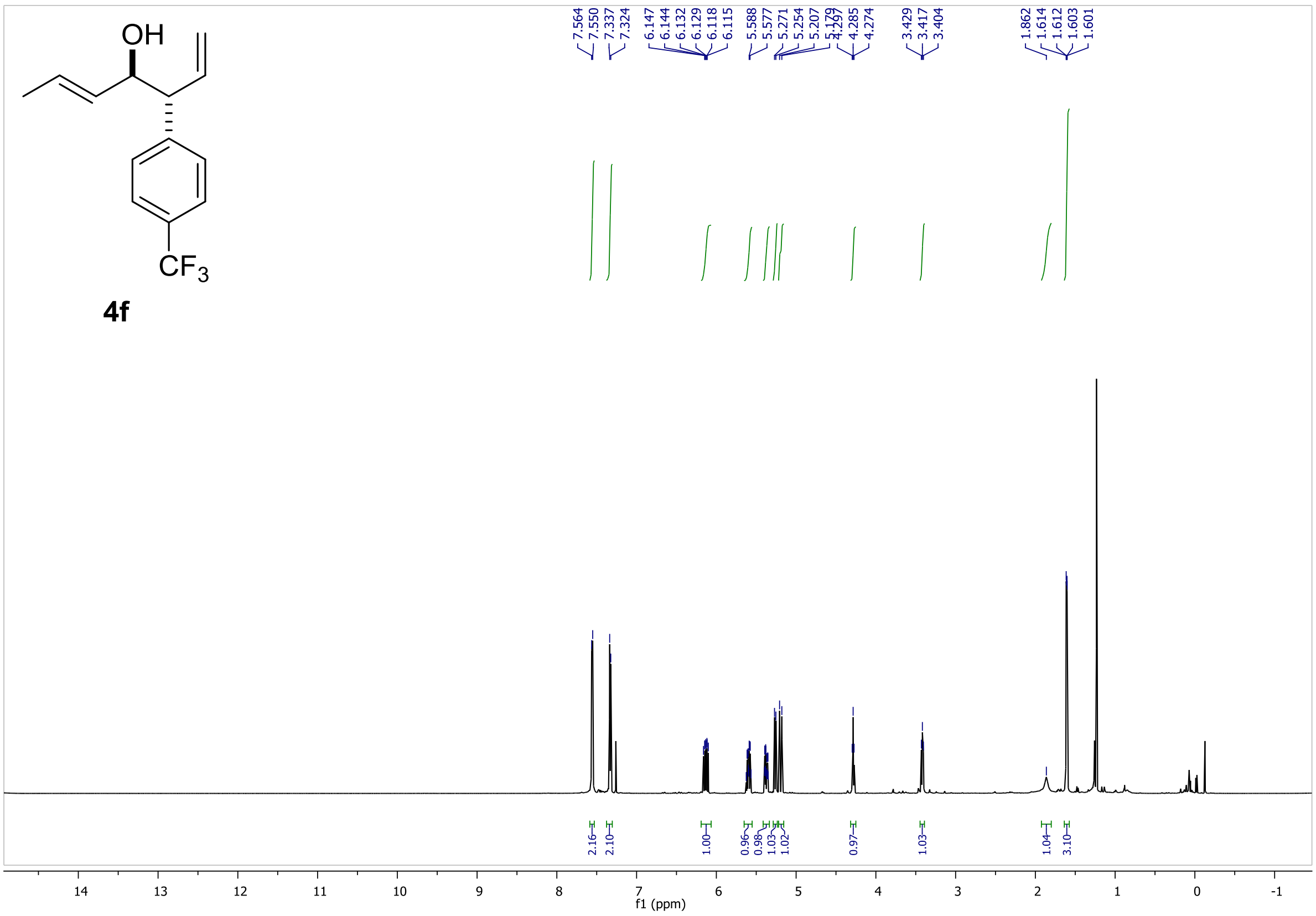

^{13}C NMR, 150 MHz, $CDCl_3$:

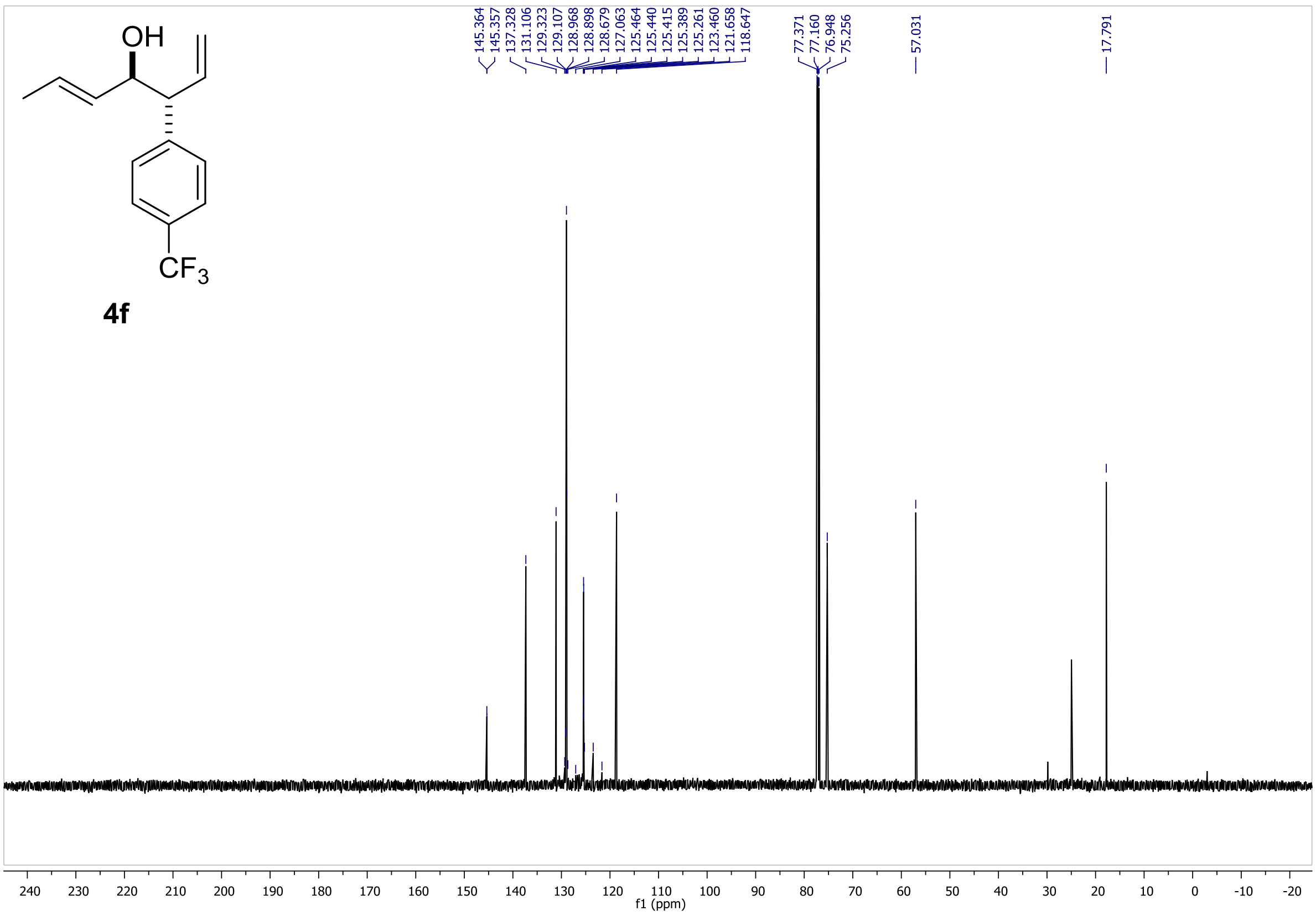

^{19}F NMR, 376 MHz, CDCl$_3$:

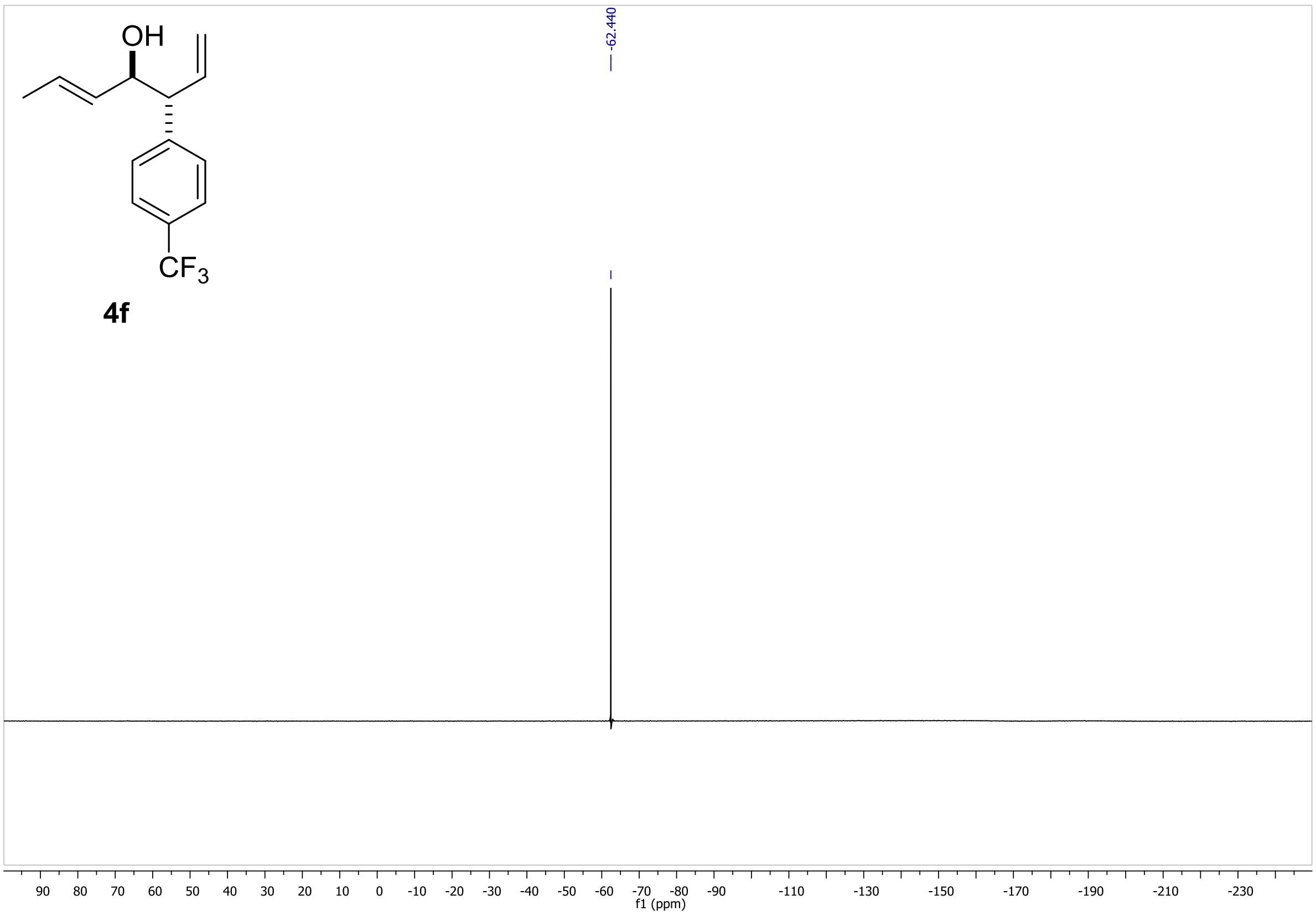

(*E*)-3-(4-methoxyphenyl)hepta-1,5-dien-4-ol (4g):

^{1}H NMR, 600 MHz, CDCl$_3$:

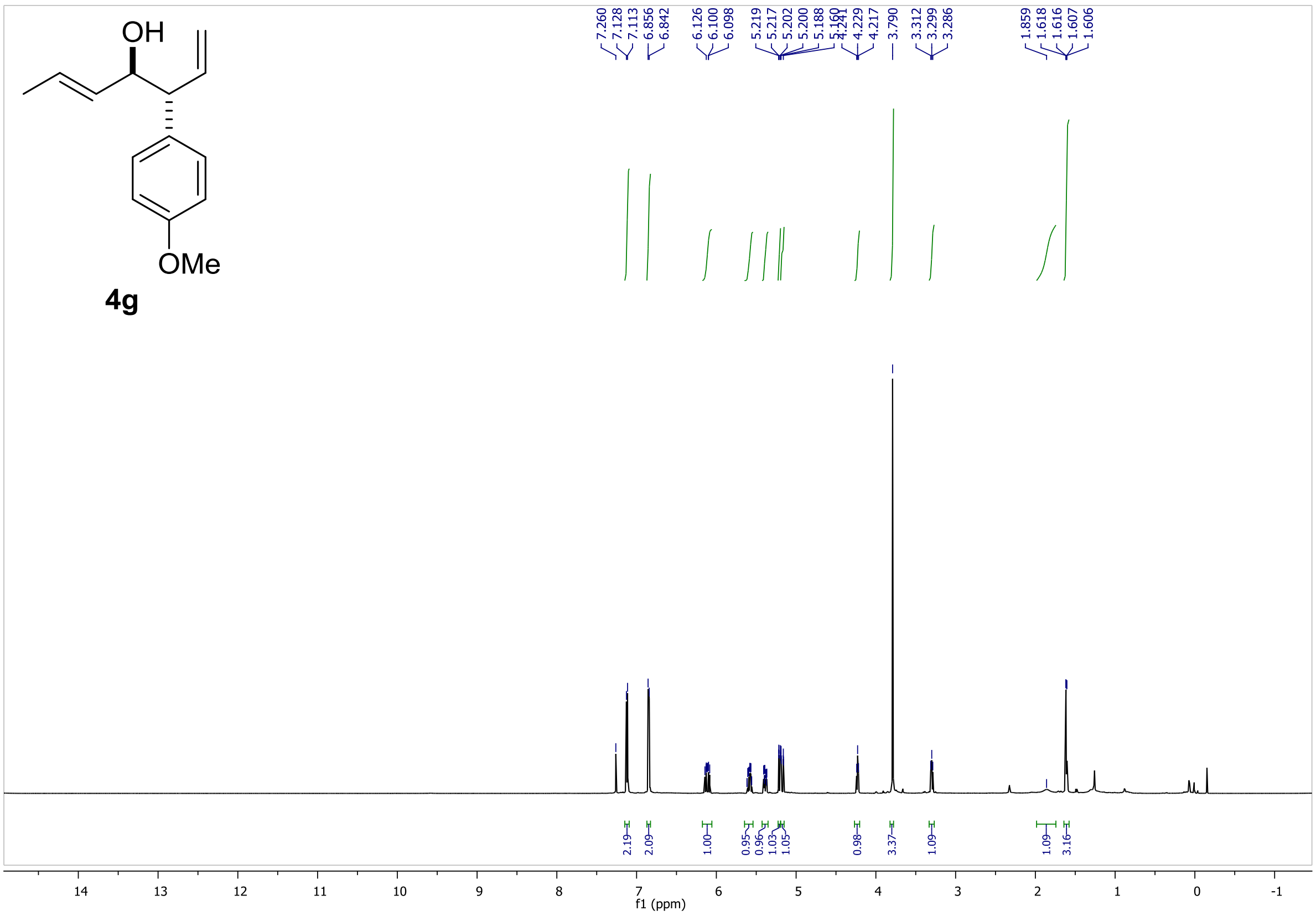

^{13}C NMR, 150 MHz, CDCl$_3$:

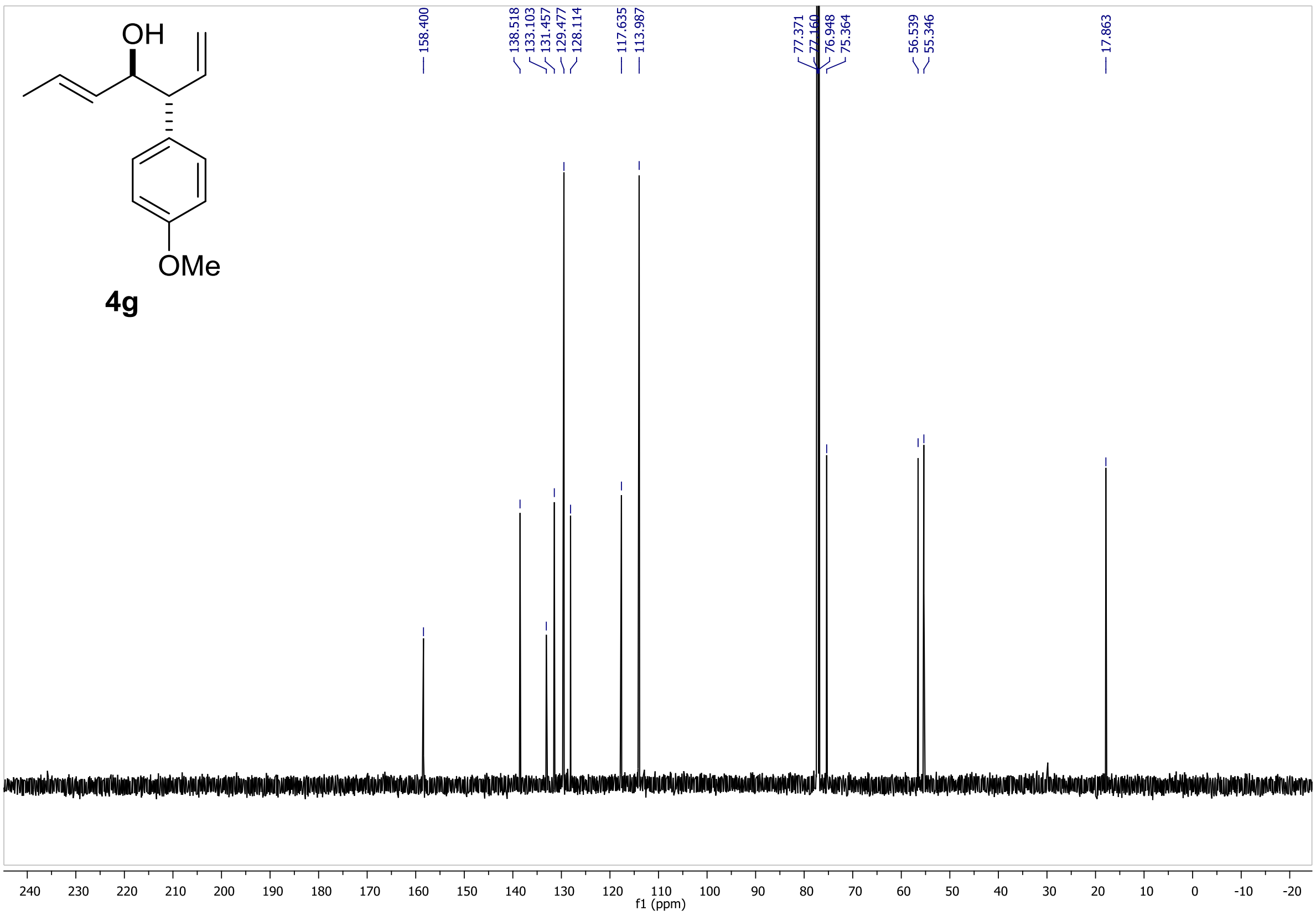

2-(4-fluorophenyl)-1-(furan-2-yl)but-3-en-1-ol (4h):

^{1}H NMR, 600 MHz, CDCl$_3$:

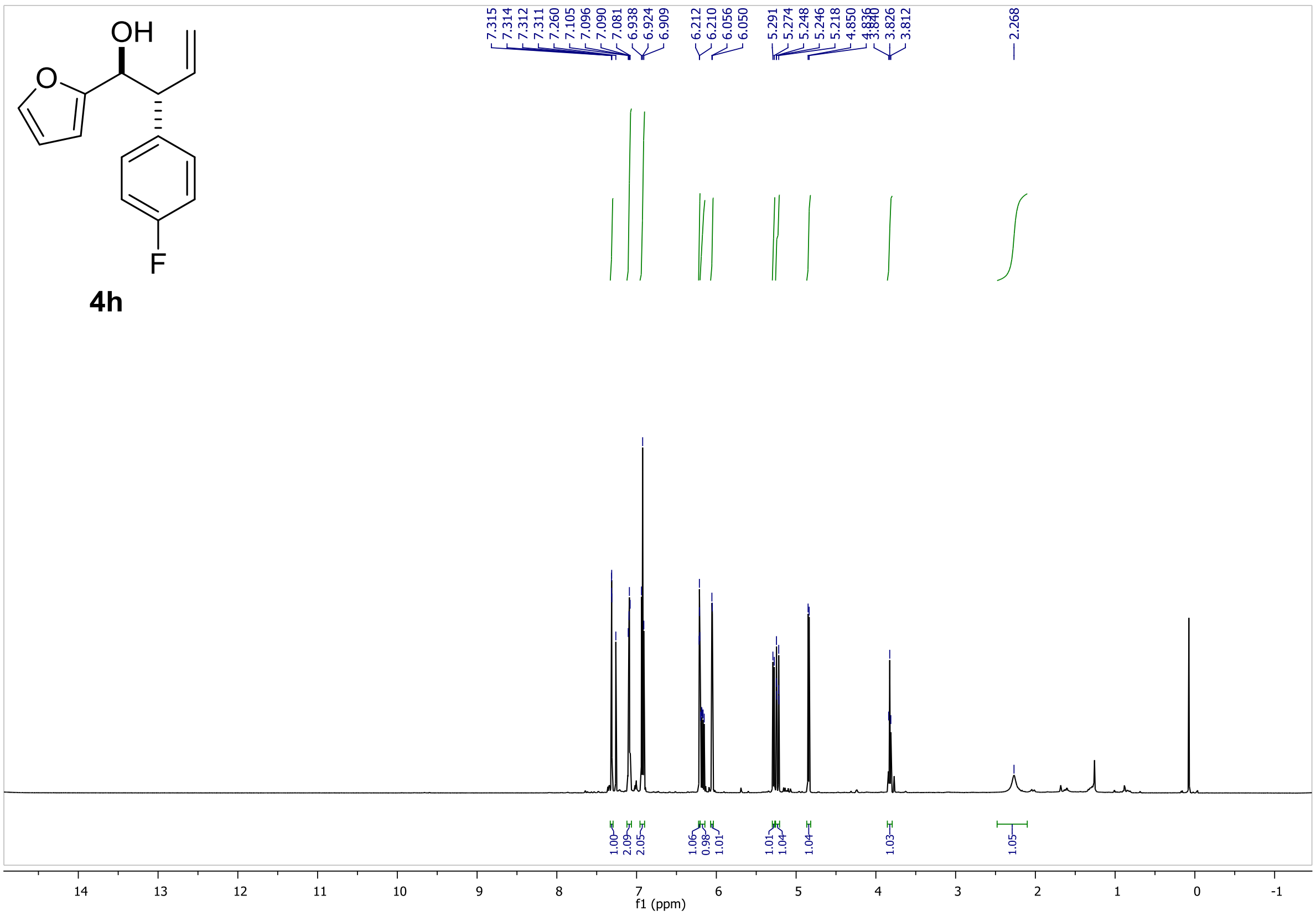

^{13}C NMR, 150 MHz, CDCl$_3$:

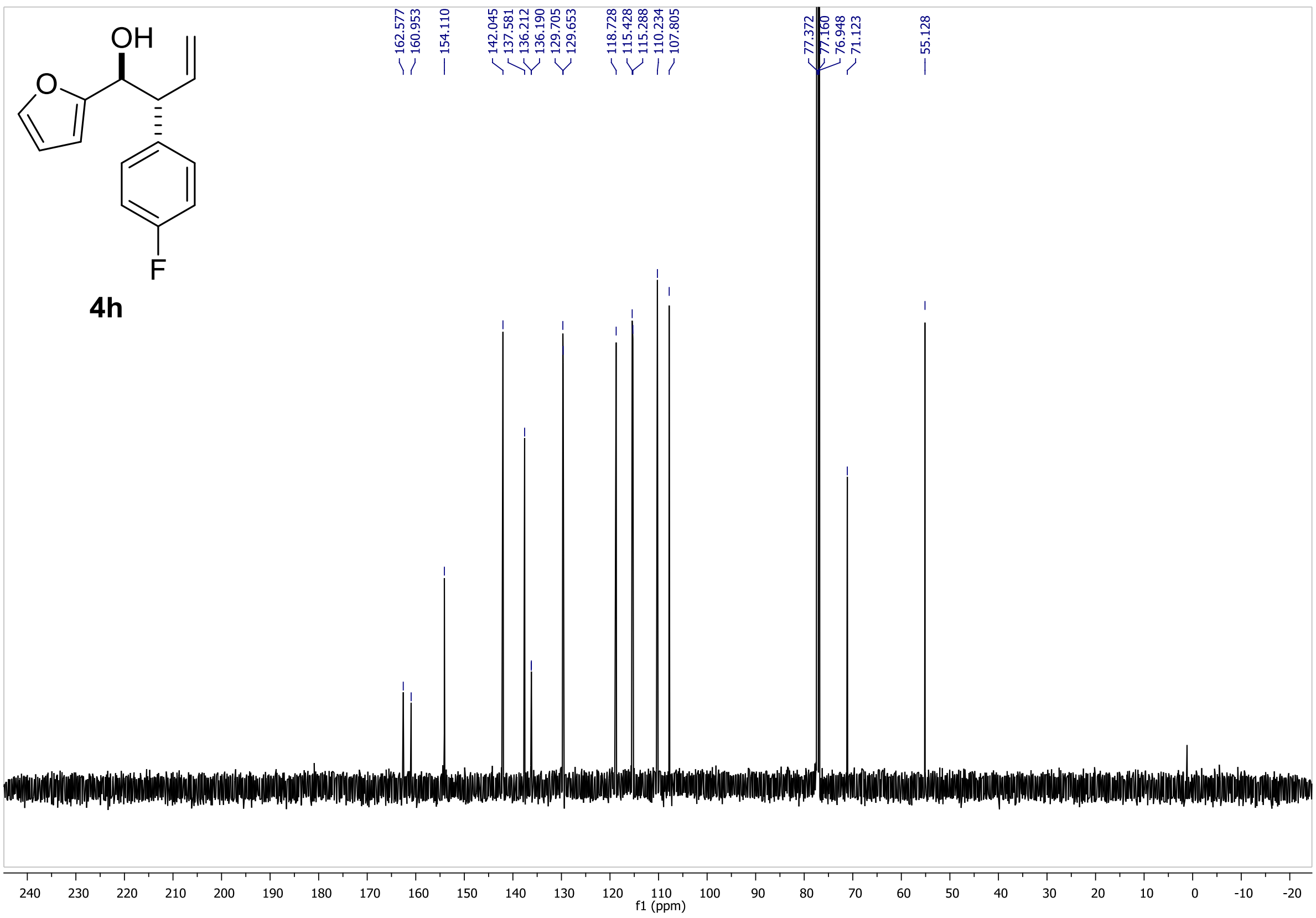

^{19}F NMR, 376 MHz, CDCl$_3$:

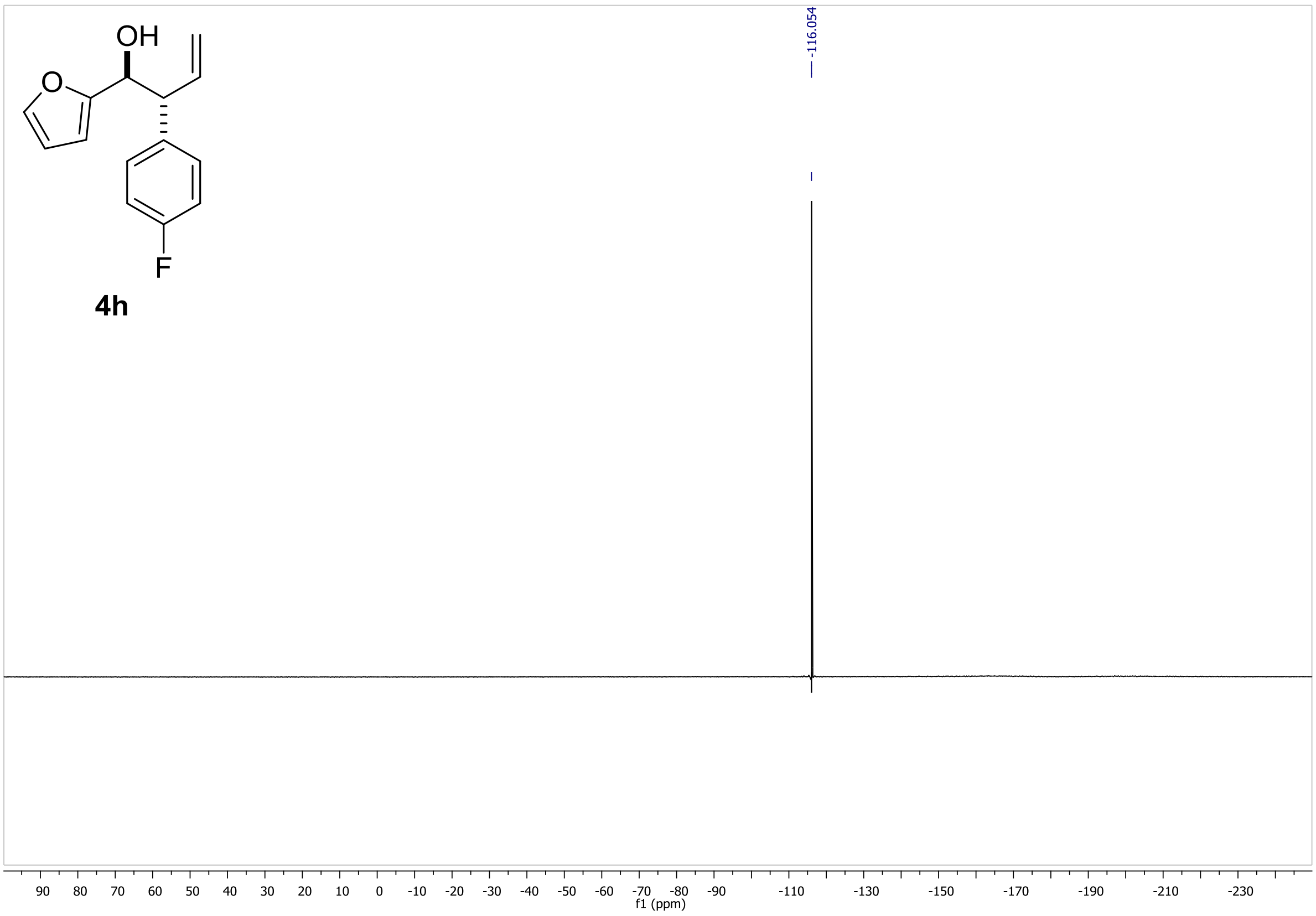

2-([1,1'-biphenyl]-4-yl)-1-(2-methoxyphenyl)but-3-en-1-ol (4i):

^{1}H NMR, 600 MHz, CDCl$_3$:

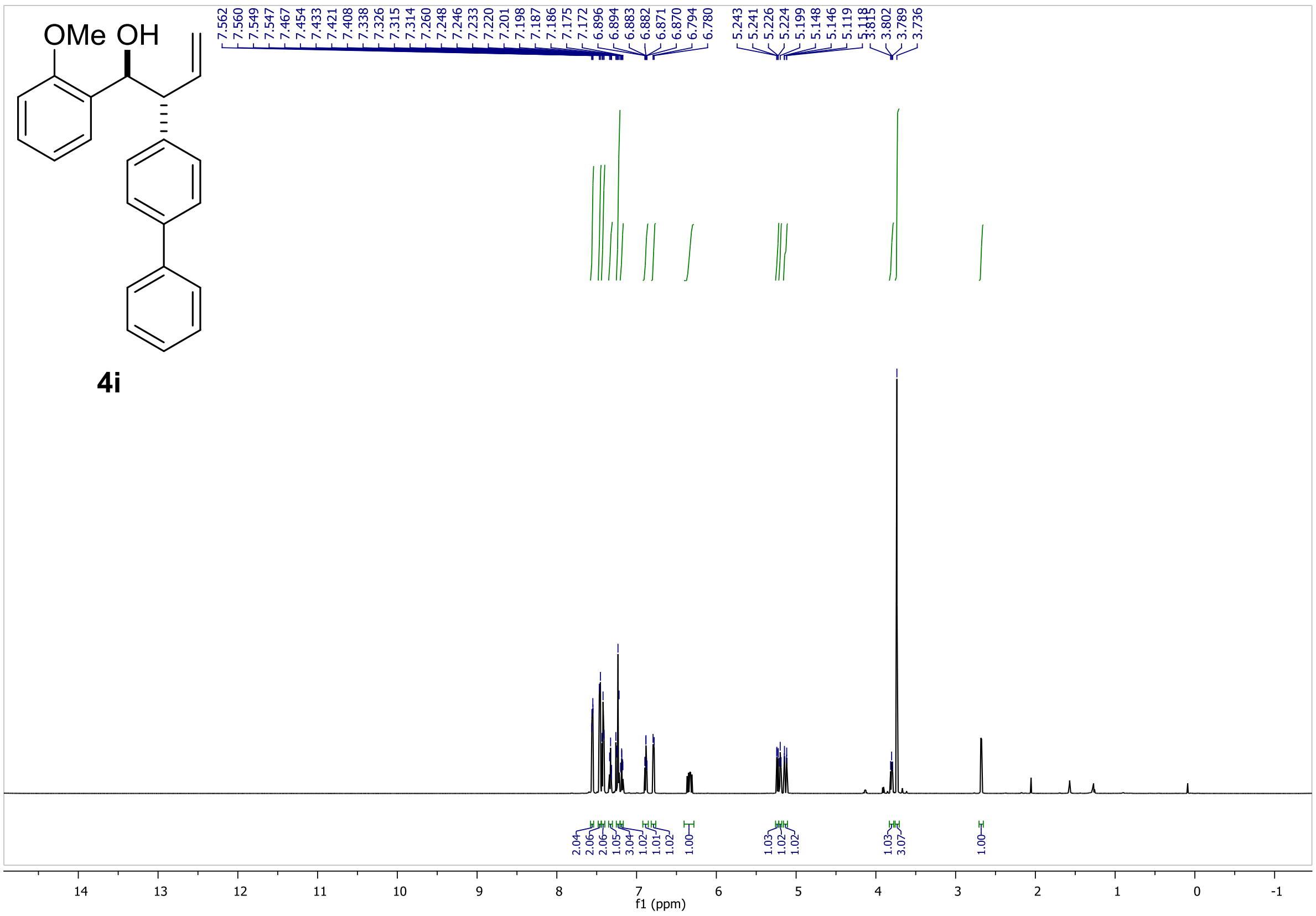

^{13}C NMR, 150 MHz, CDCl$_3$:

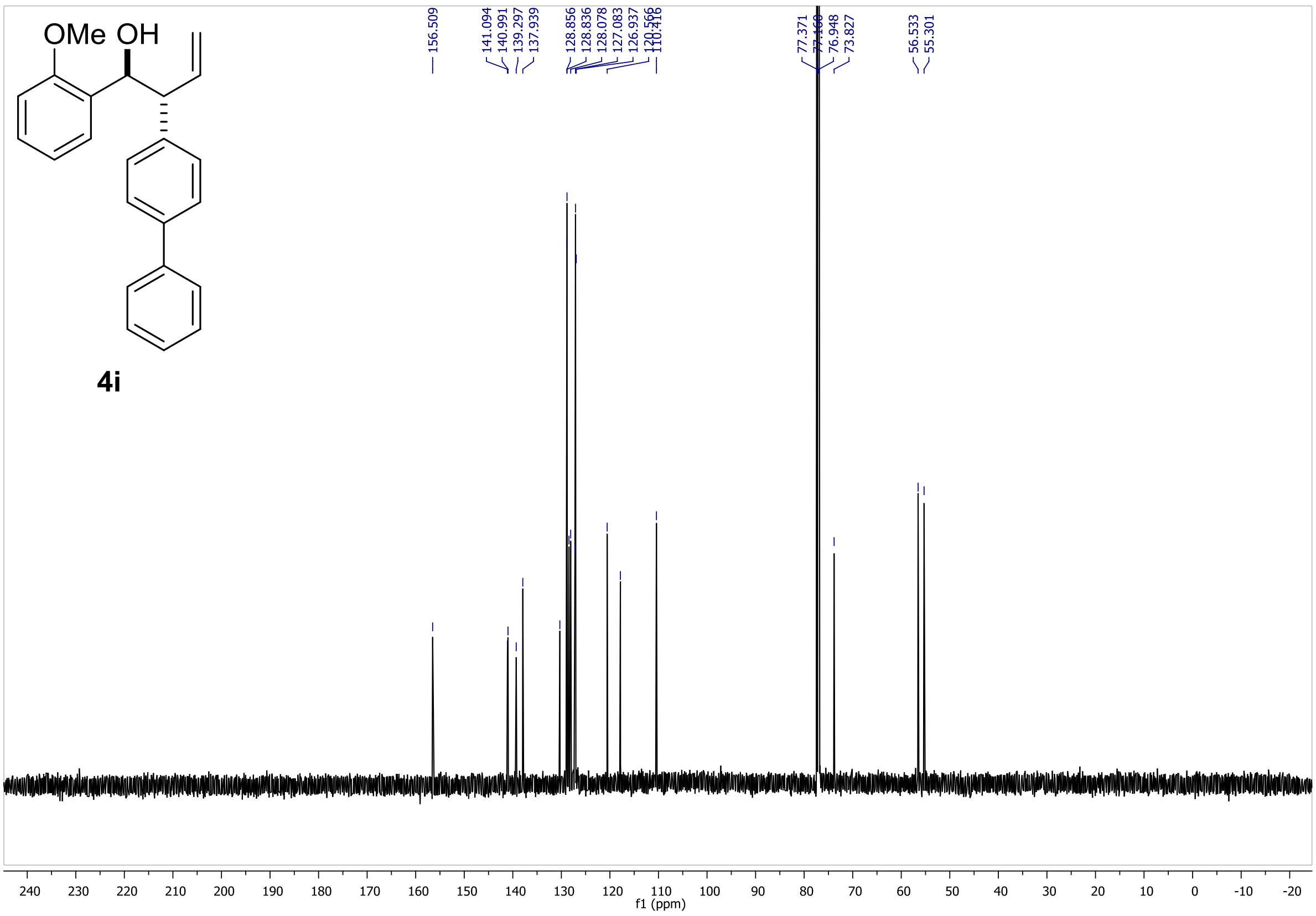

4-(3-fluorophenyl)hex-5-en-3-ol (4j):

^{1}H NMR, 600 MHz, CDCl$_3$:

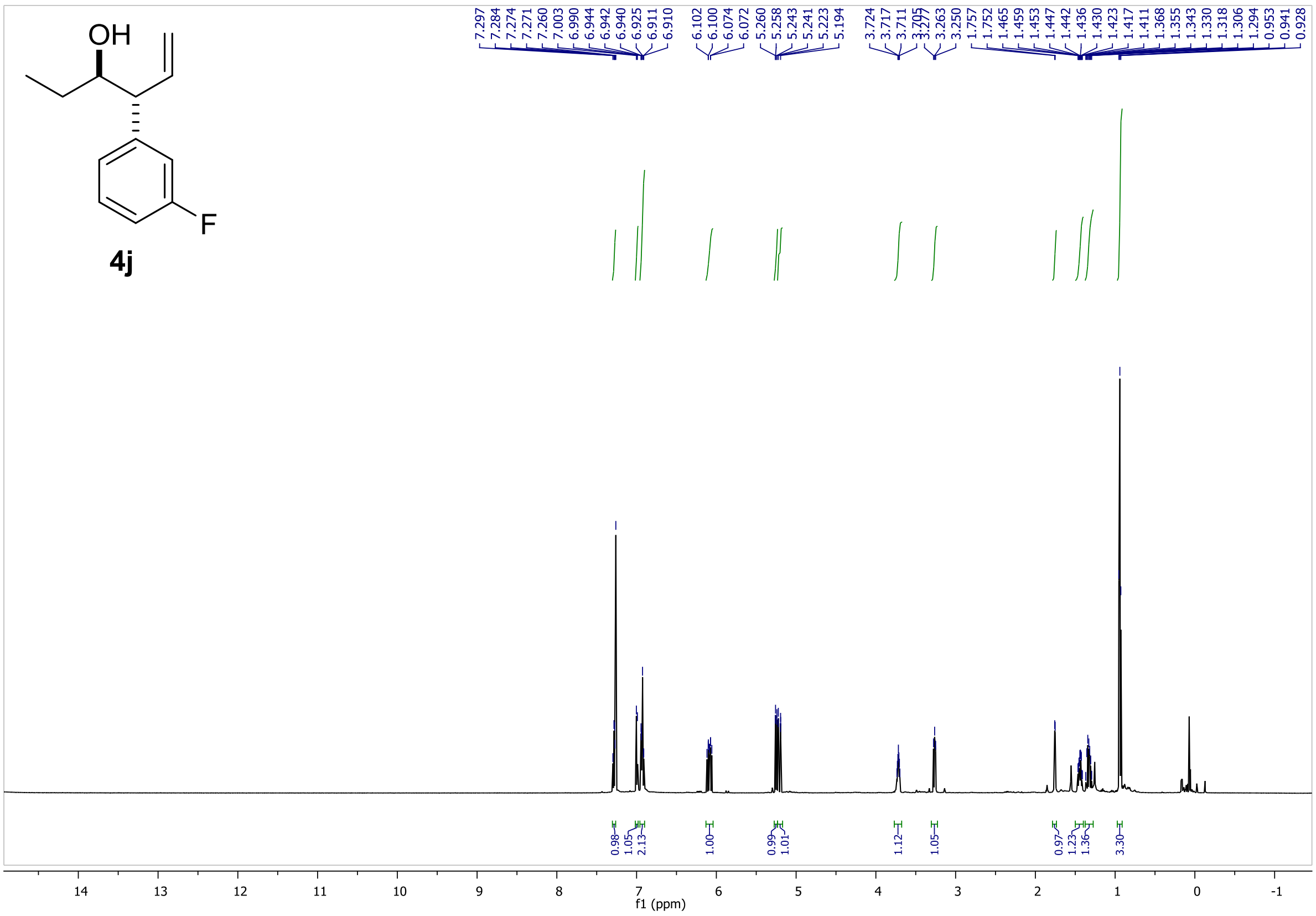

^{13}C NMR, 150 MHz, CDCl$_3$:

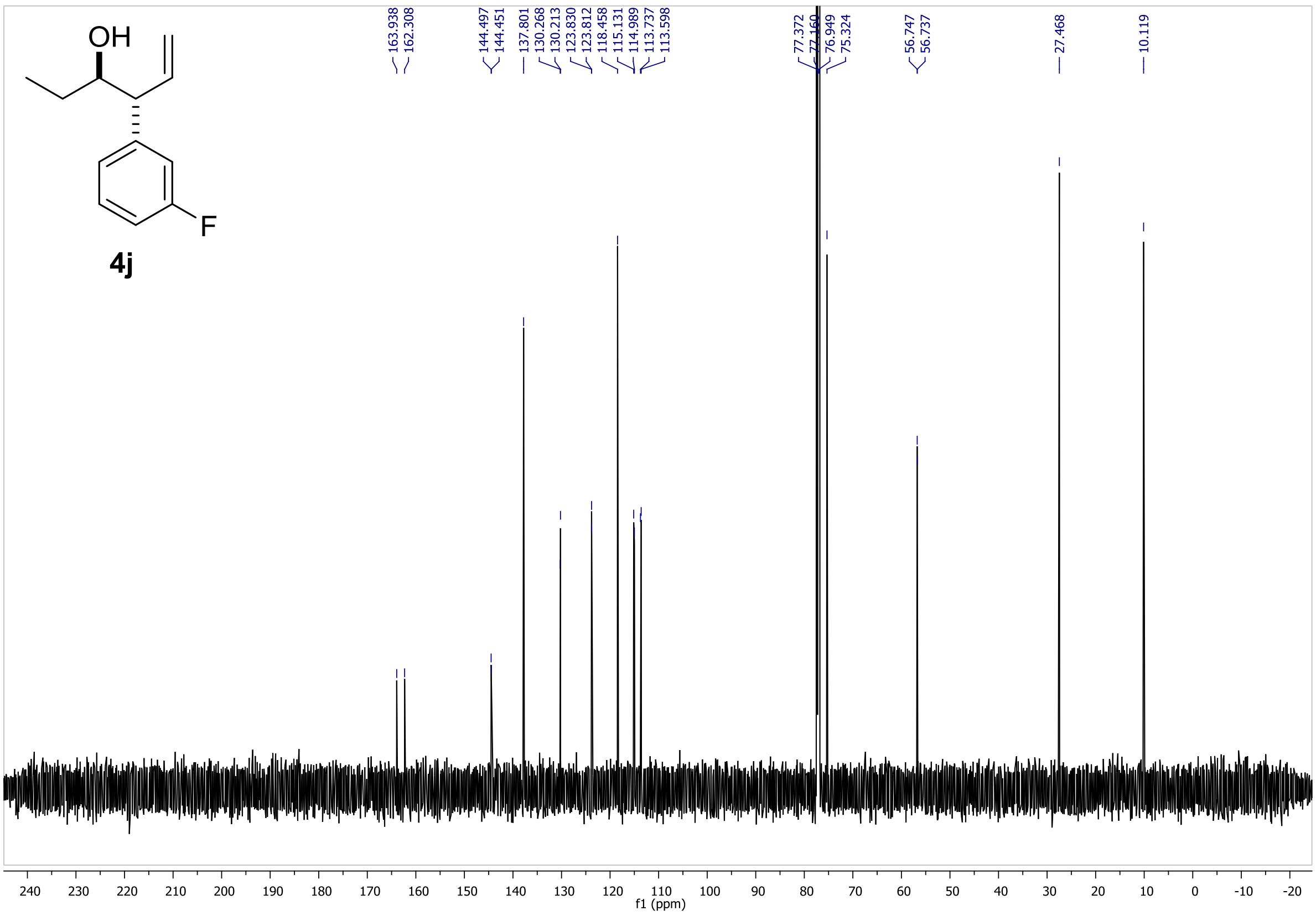

^{19}F NMR, 376 MHz, $CDCl_3$:

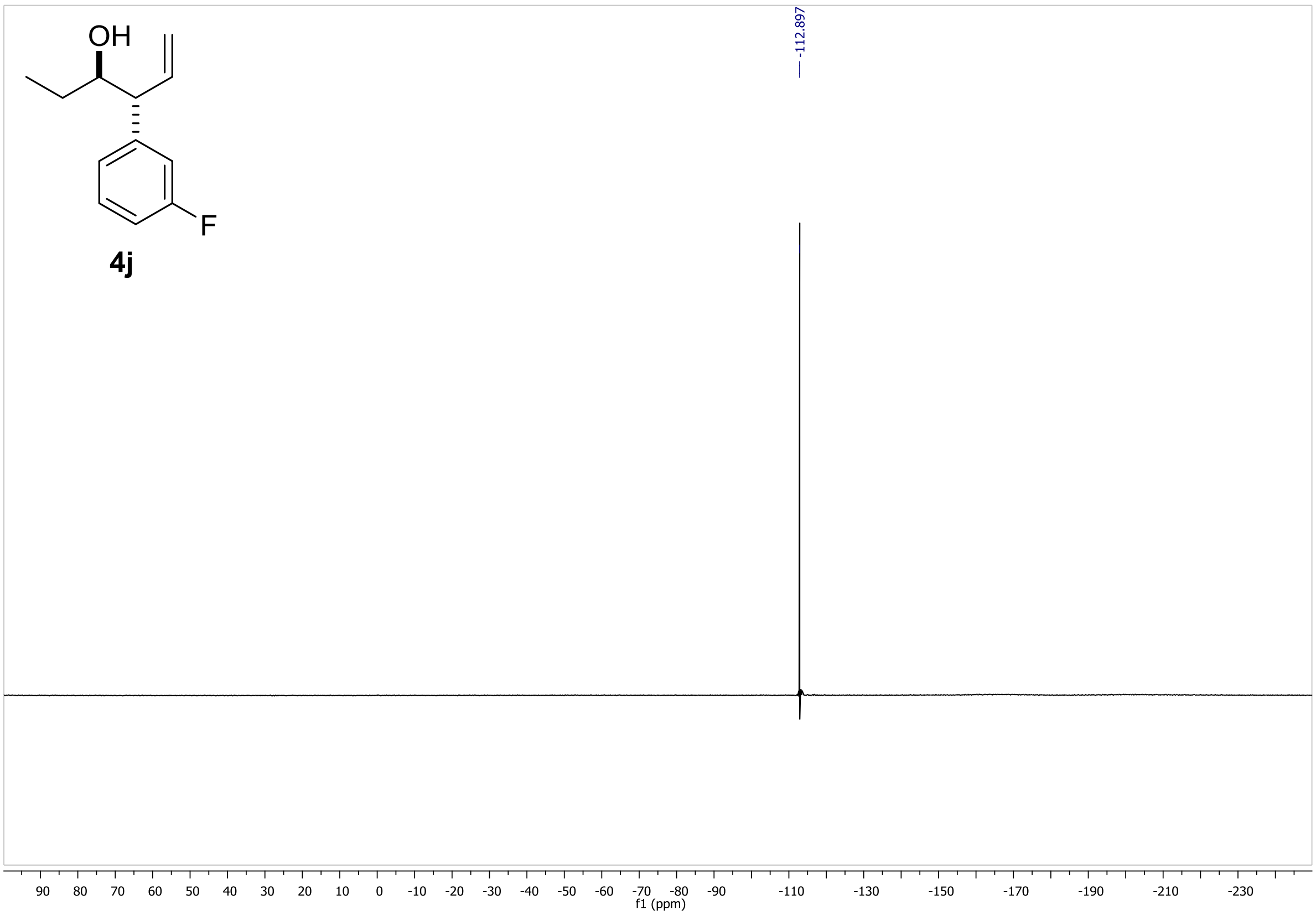

6-methyl-3-phenylhept-1-en-4-ol (4k):

^{1}H NMR, 600 MHz, $CDCl_3$:

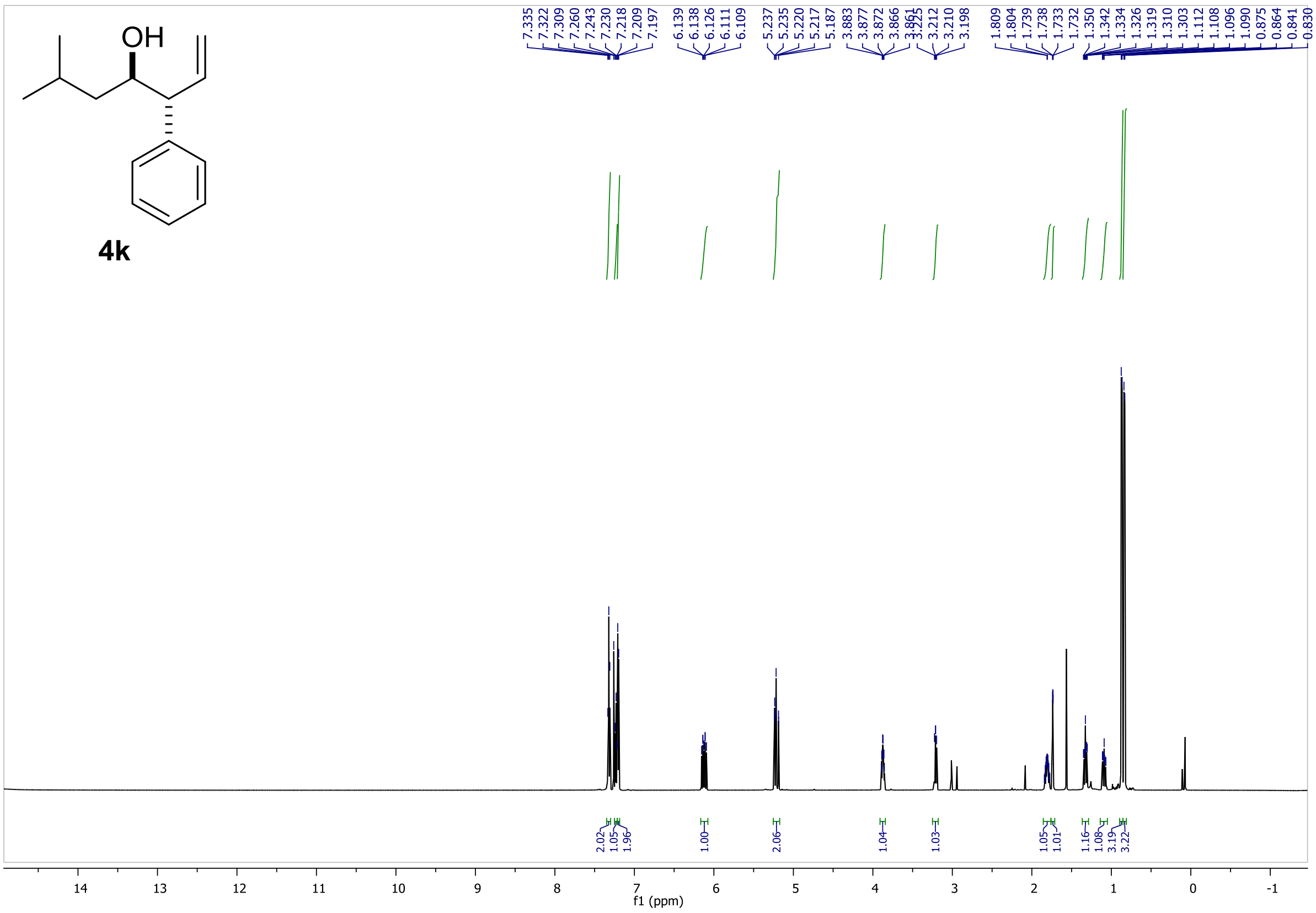

^{13}C NMR, 150 MHz, $CDCl_3$:

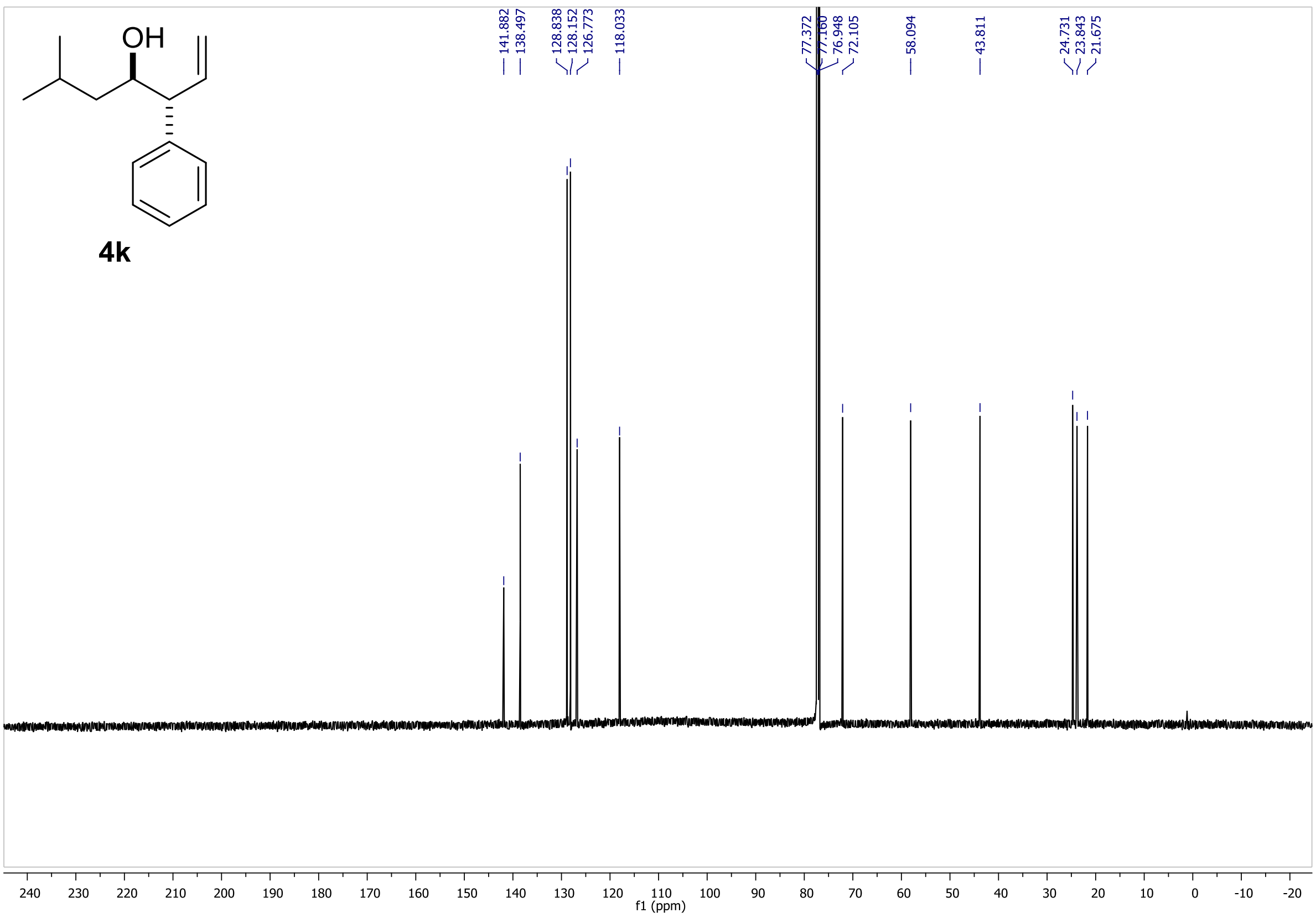

(4-fluorophenyl)-6-methylhept-1-en-4-ol (4l):

^{1}H NMR, 600 MHz, $CDCl_3$:

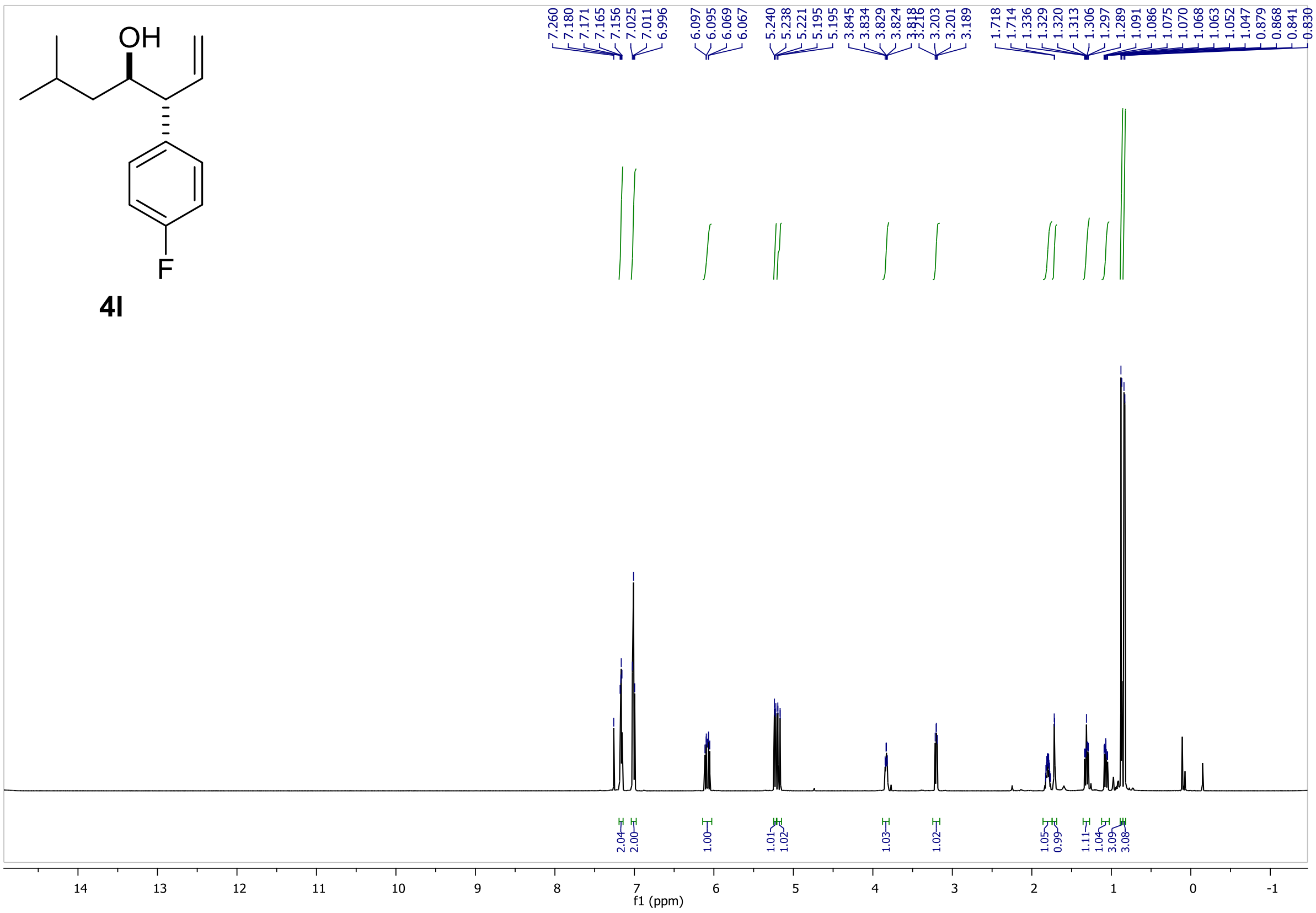

^{13}C NMR, 150 MHz, $CDCl_3$:

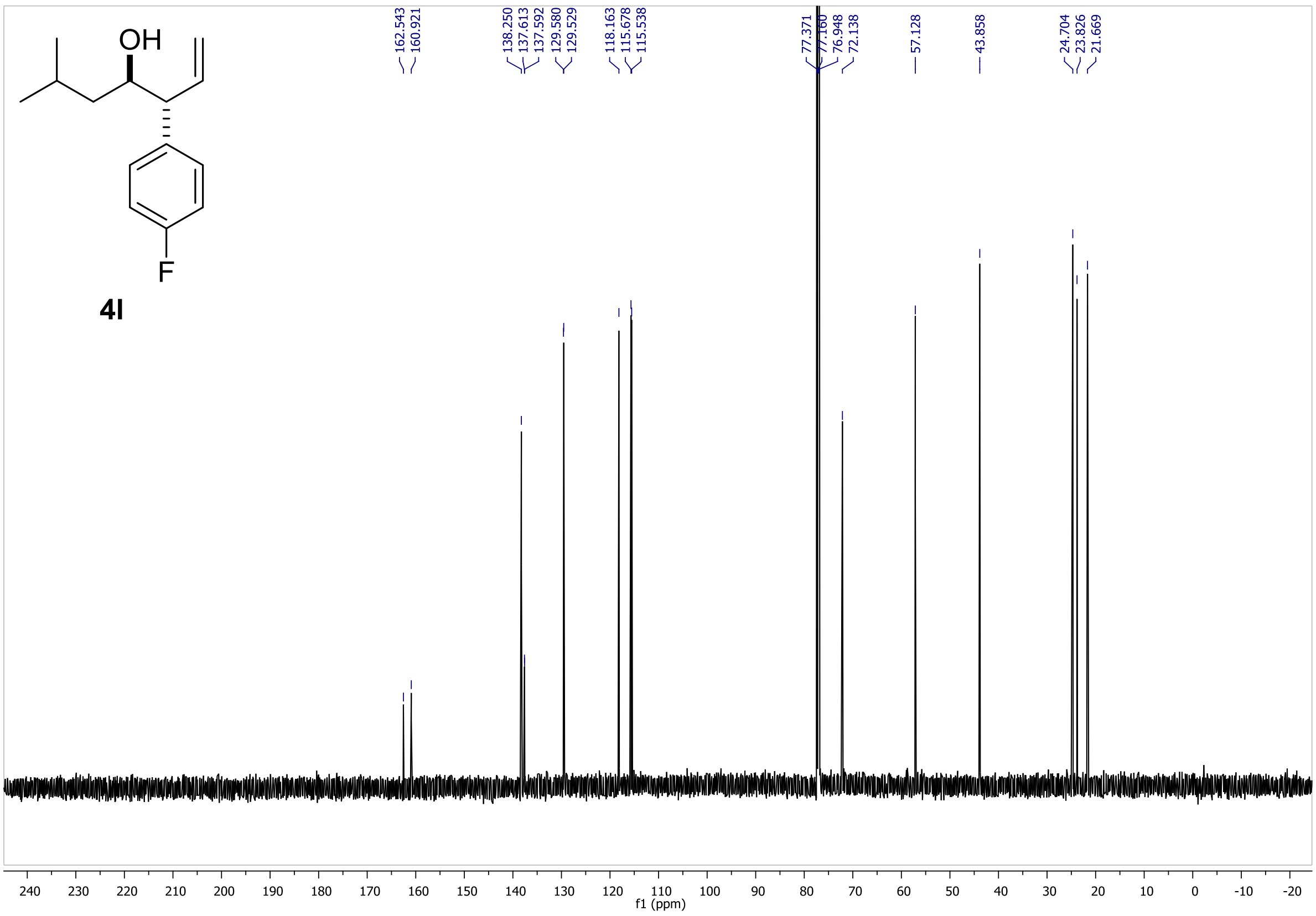

^{19}F NMR, 376 MHz, $CDCl_3$:

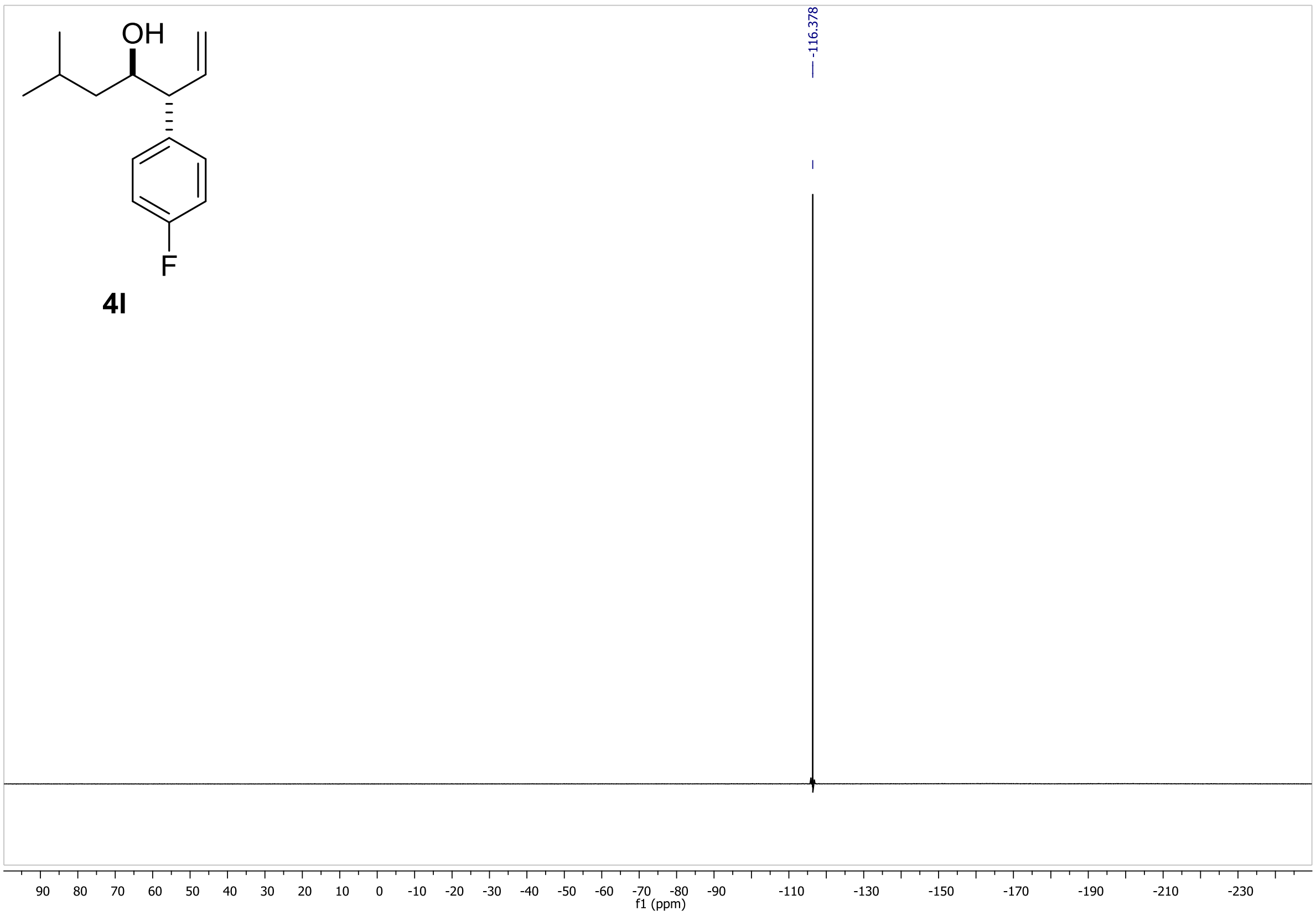

(*E*)-3-(4-fluorophenyl)-6,10-dimethylundeca-1,5,9-trien-4-ol (4m-*E*):

^{1}H NMR, 600 MHz, $CDCl_3$:

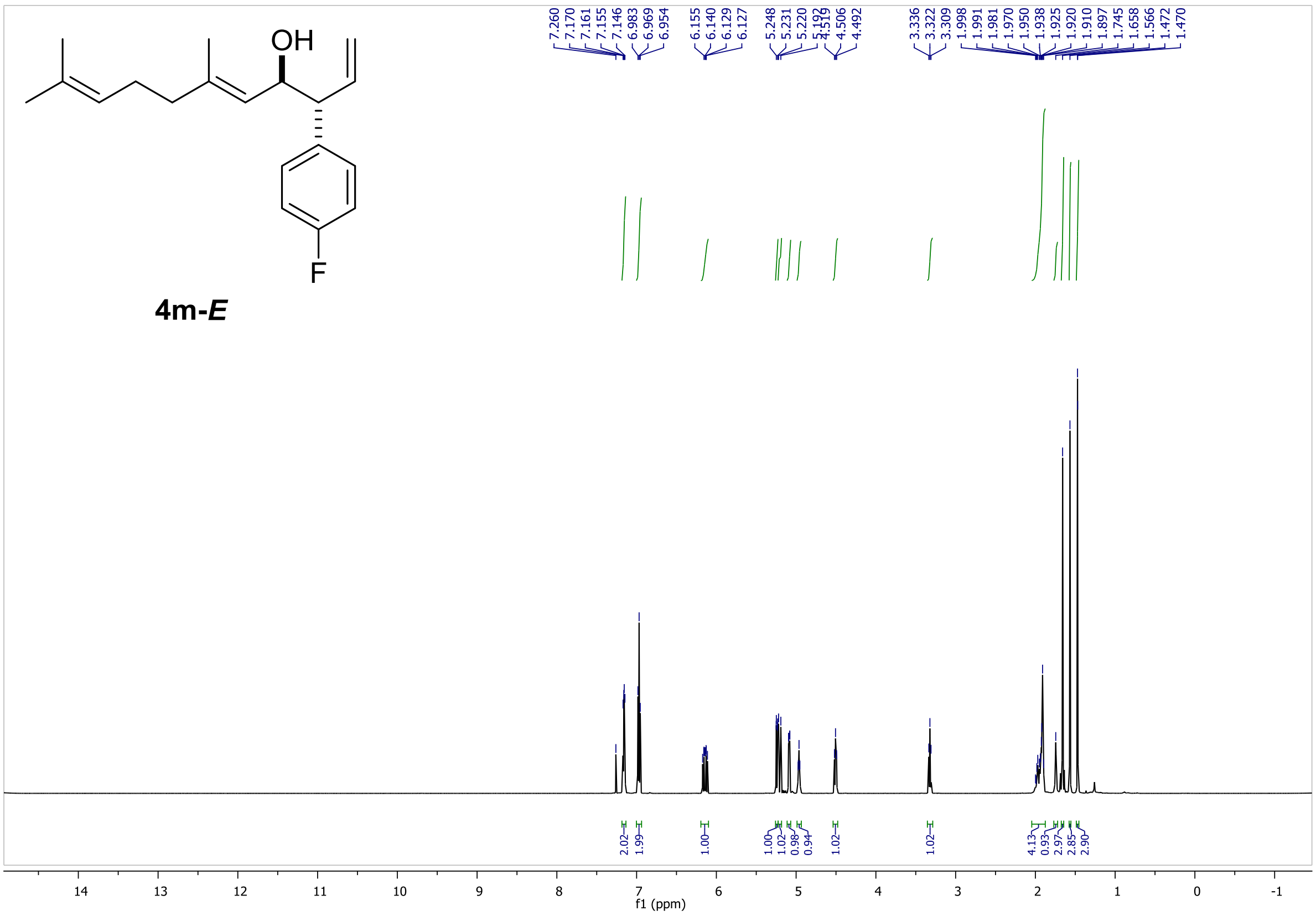

^{13}C NMR, 150 MHz, $CDCl_3$:

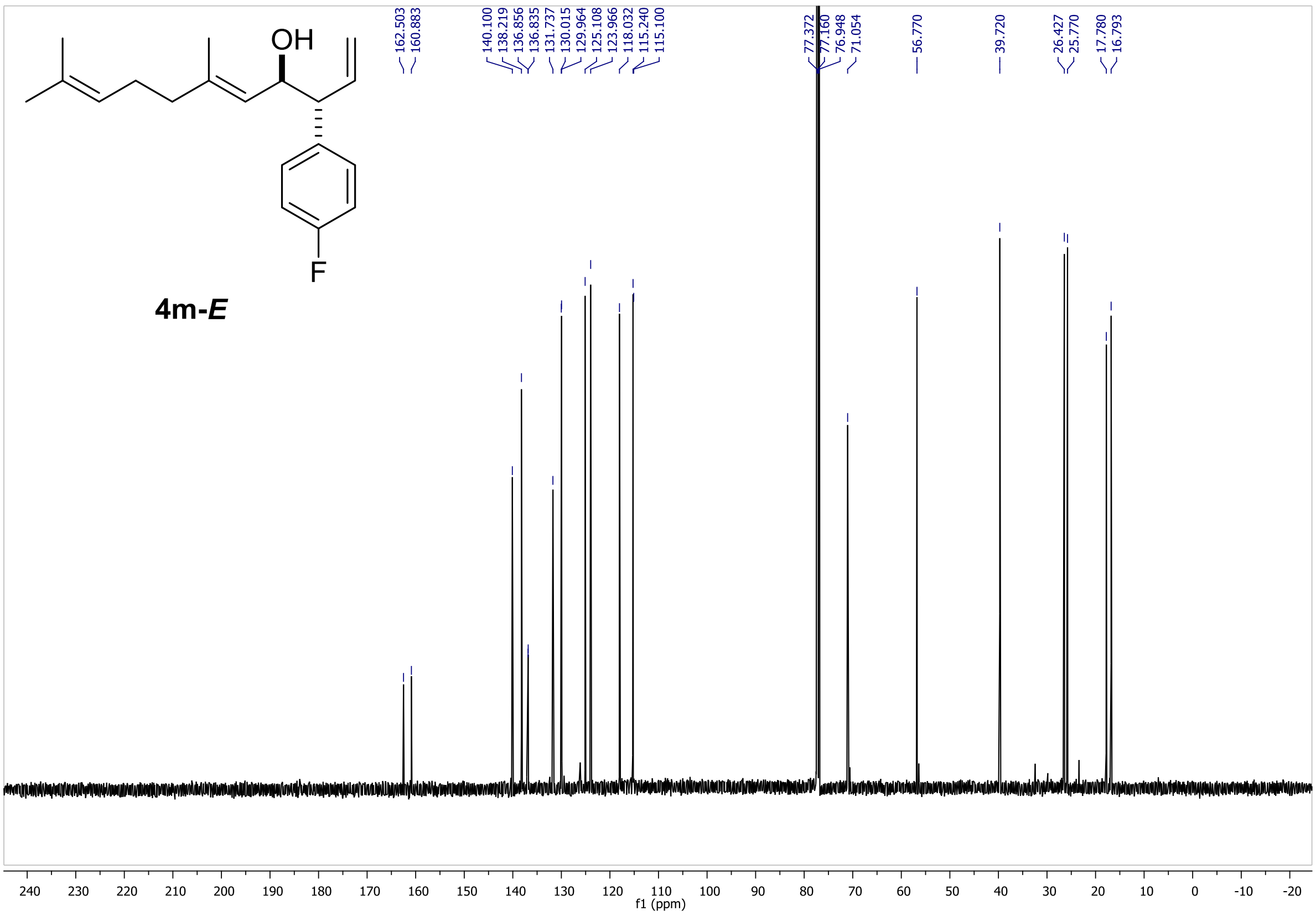

^{19}F NMR, 376 MHz, $CDCl_3$:

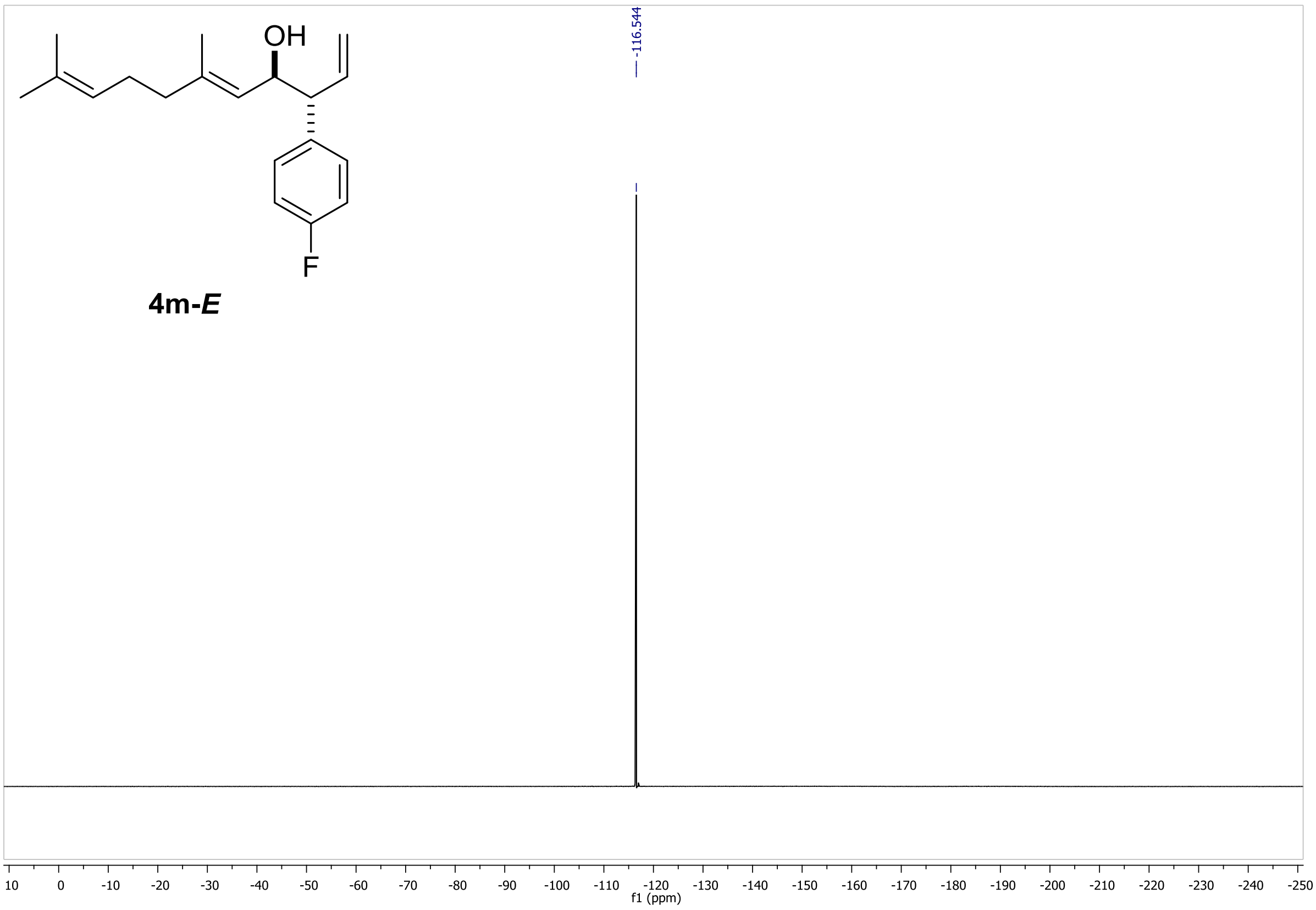

(*Z*)-3-(4-fluorophenyl)-6,10-dimethylundeca-1,5,9-trien-4-ol (4m-*Z*):

^{1}H NMR, 600 MHz, $CDCl_3$:

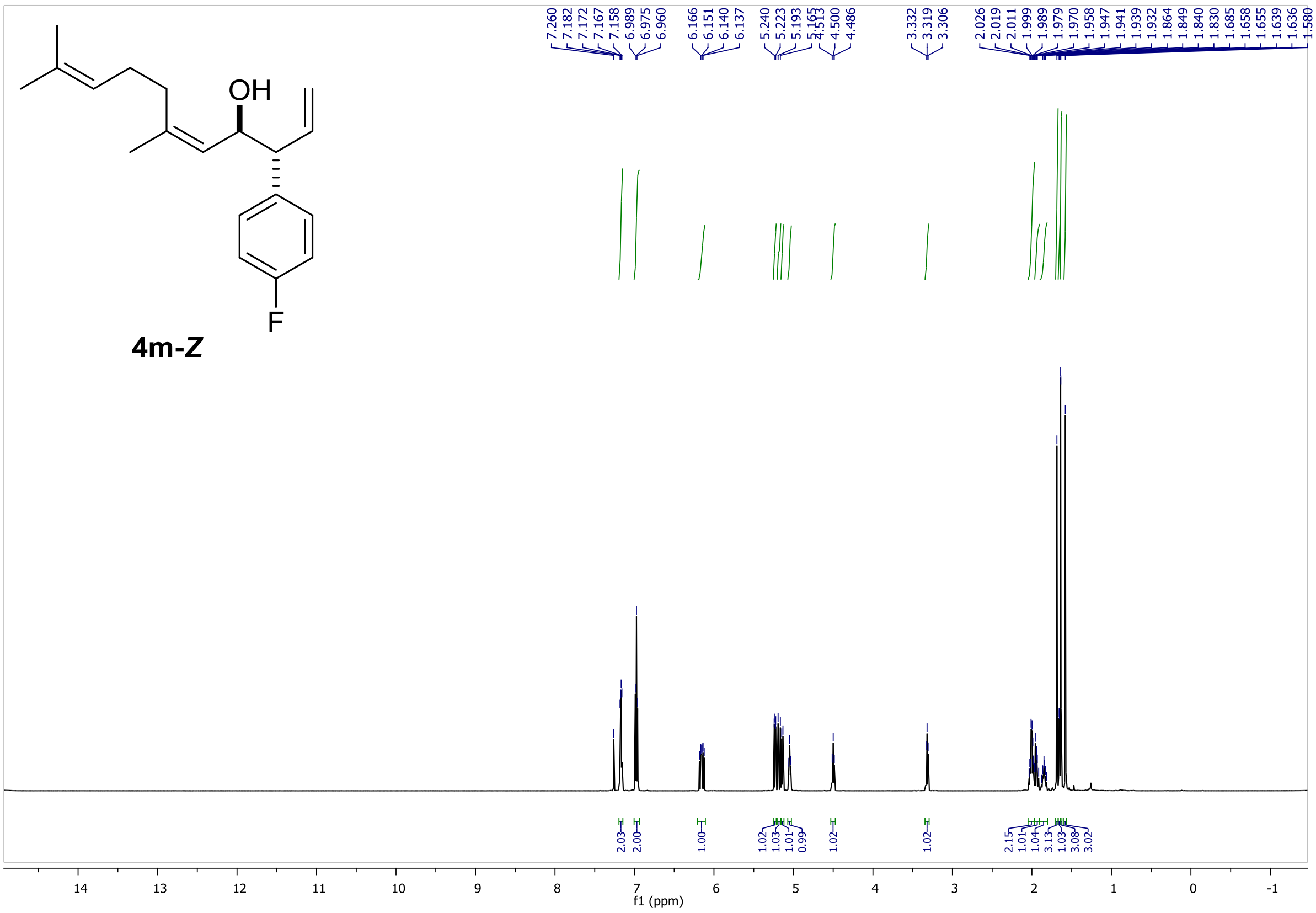

^{13}C NMR, 150 MHz, $CDCl_3$:

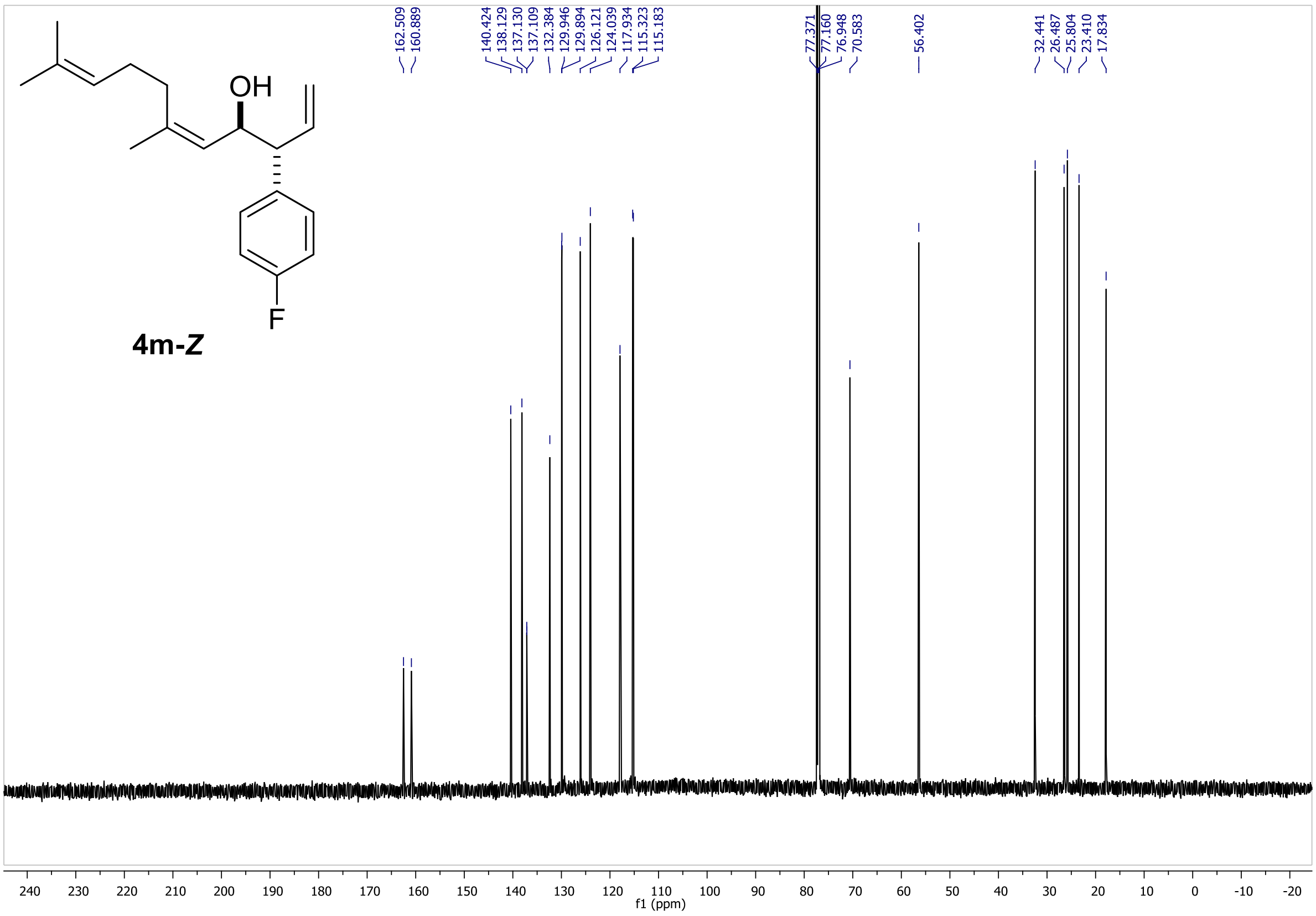

^{19}F NMR, 376 MHz, $CDCl_3$:

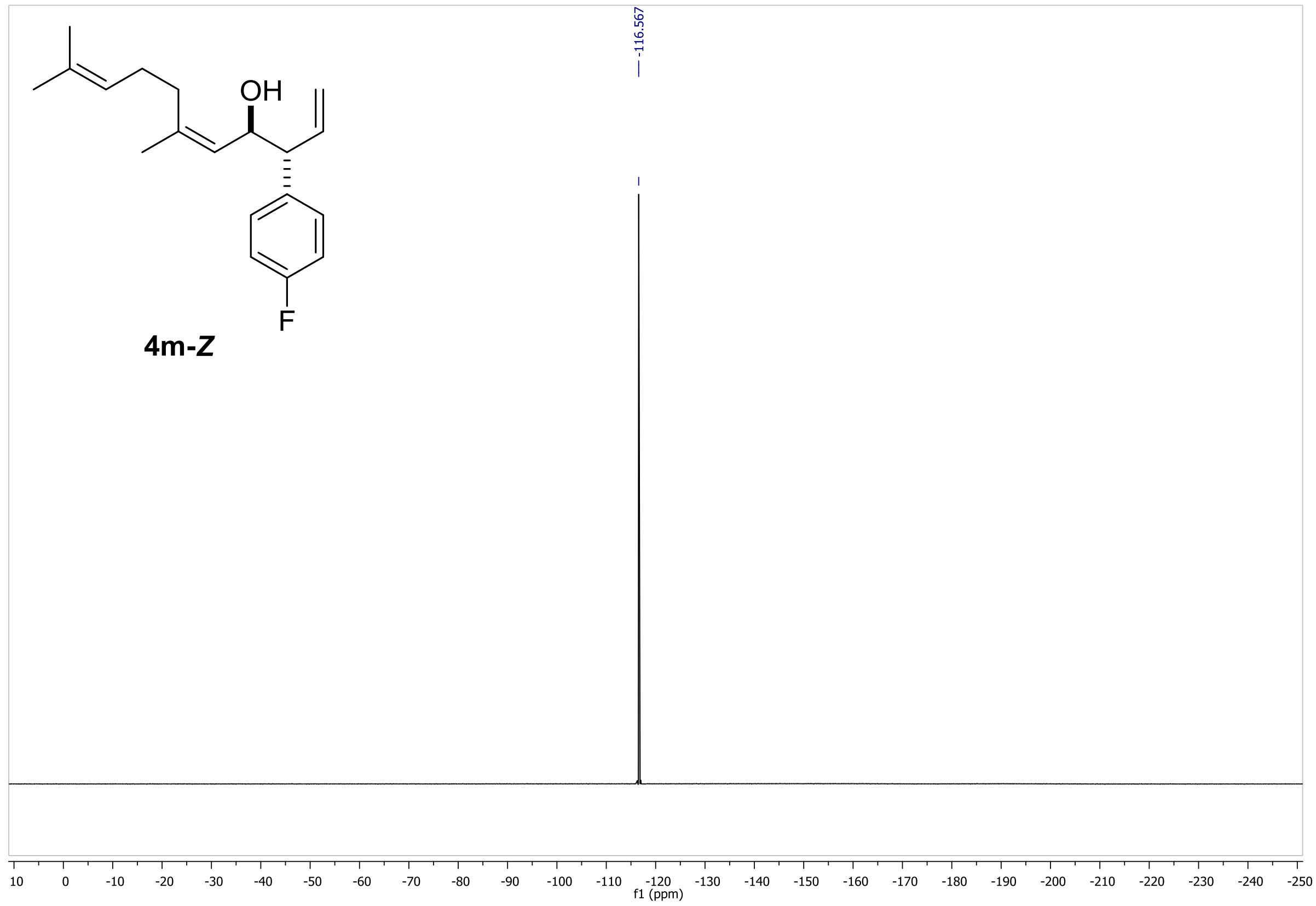

1-(4-bromophenyl)-2-(chloromethyl)but-3-en-1-ol (4n):

^{1}H NMR, 600 MHz, $CDCl_3$:

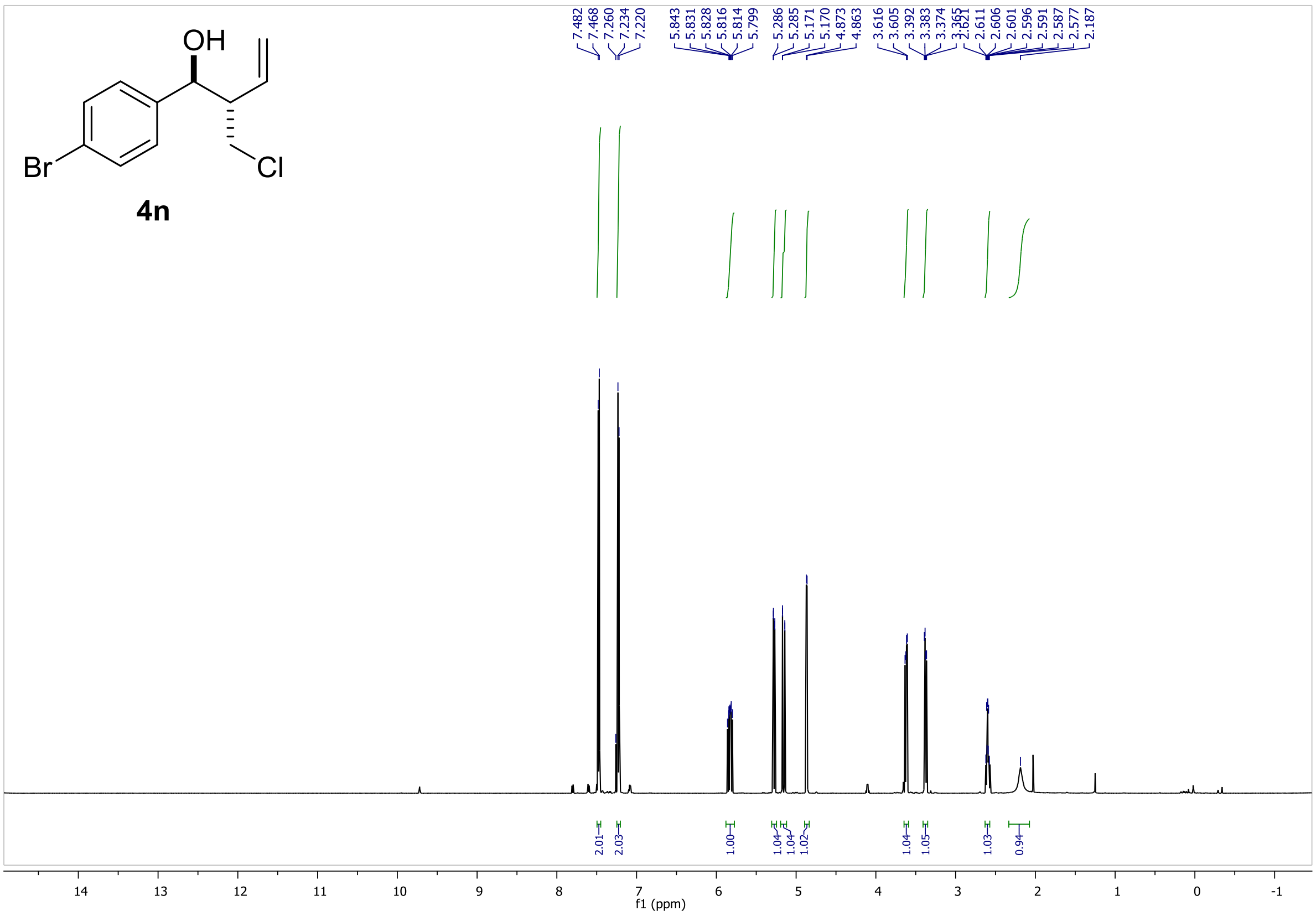

^{13}C NMR, 150 MHz, $CDCl_3$:

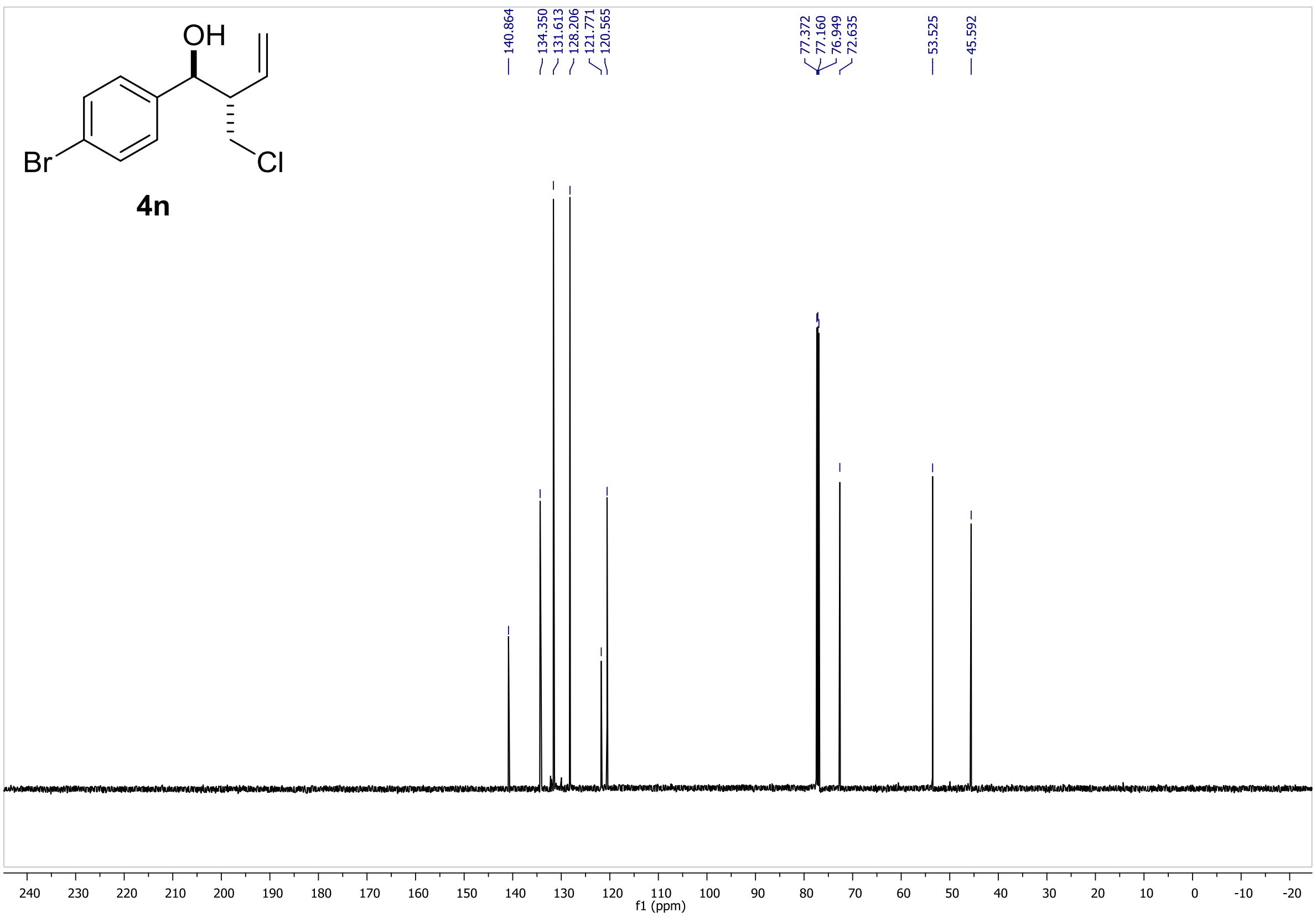

1-(4-bromophenyl)-2-vinylhexan-1-ol (4o):

^{1}H NMR, 600 MHz, $CDCl_3$:

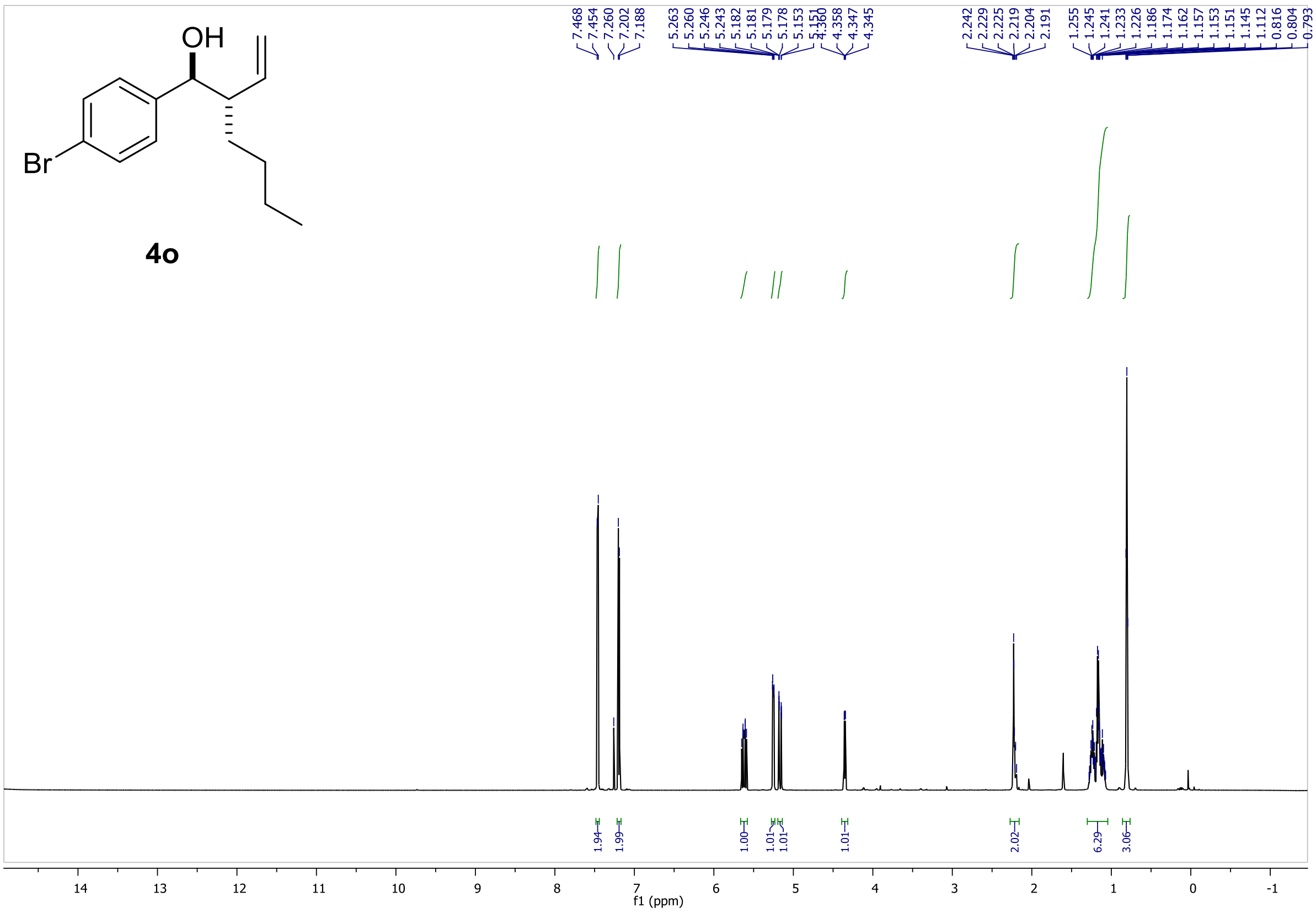

^{13}C NMR, 150 MHz, $CDCl_3$:

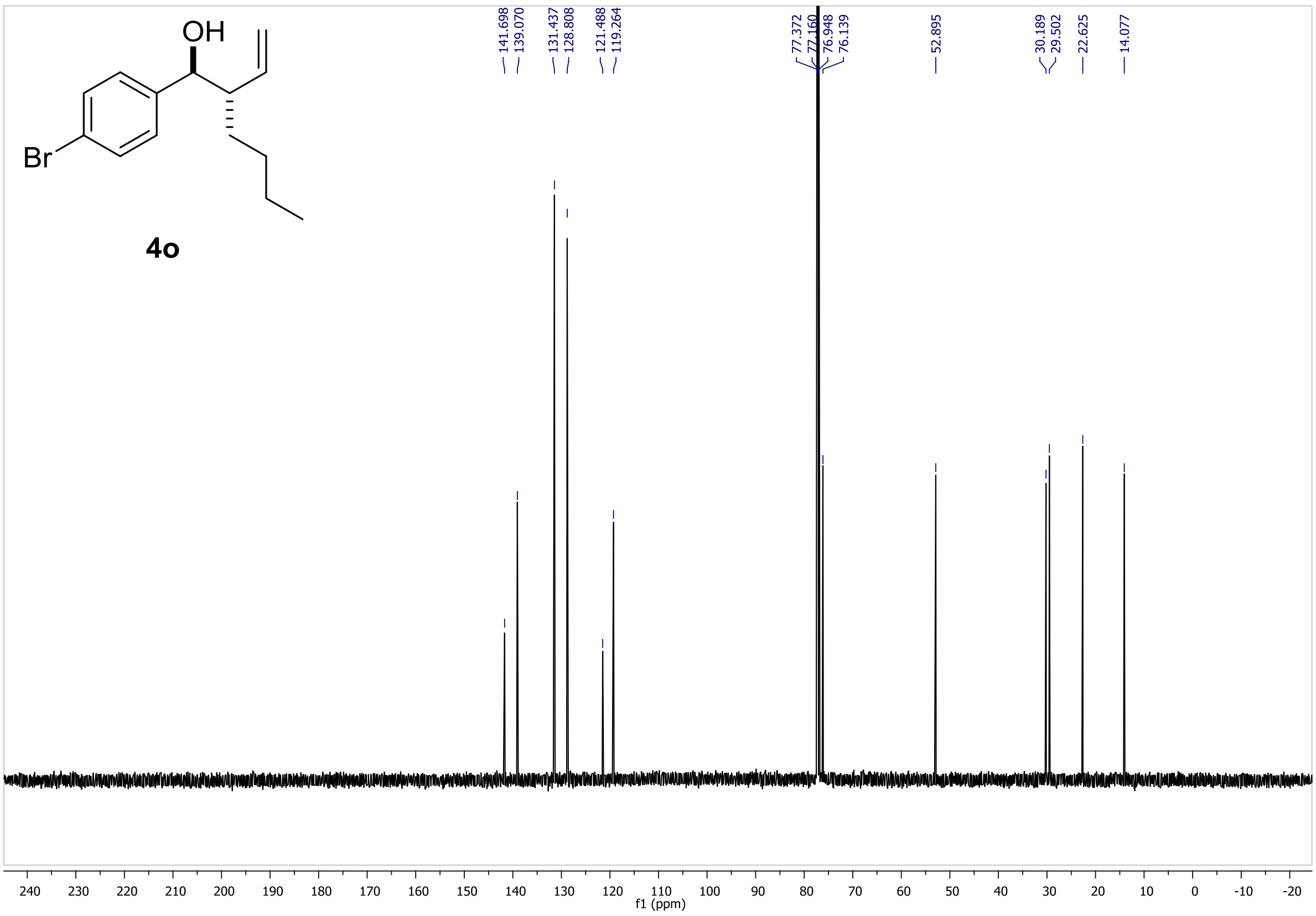

2-benzyl-1-(4-bromophenyl)but-3-en-1-ol (4p):

^{1}H NMR, 600 MHz, $CDCl_3$:

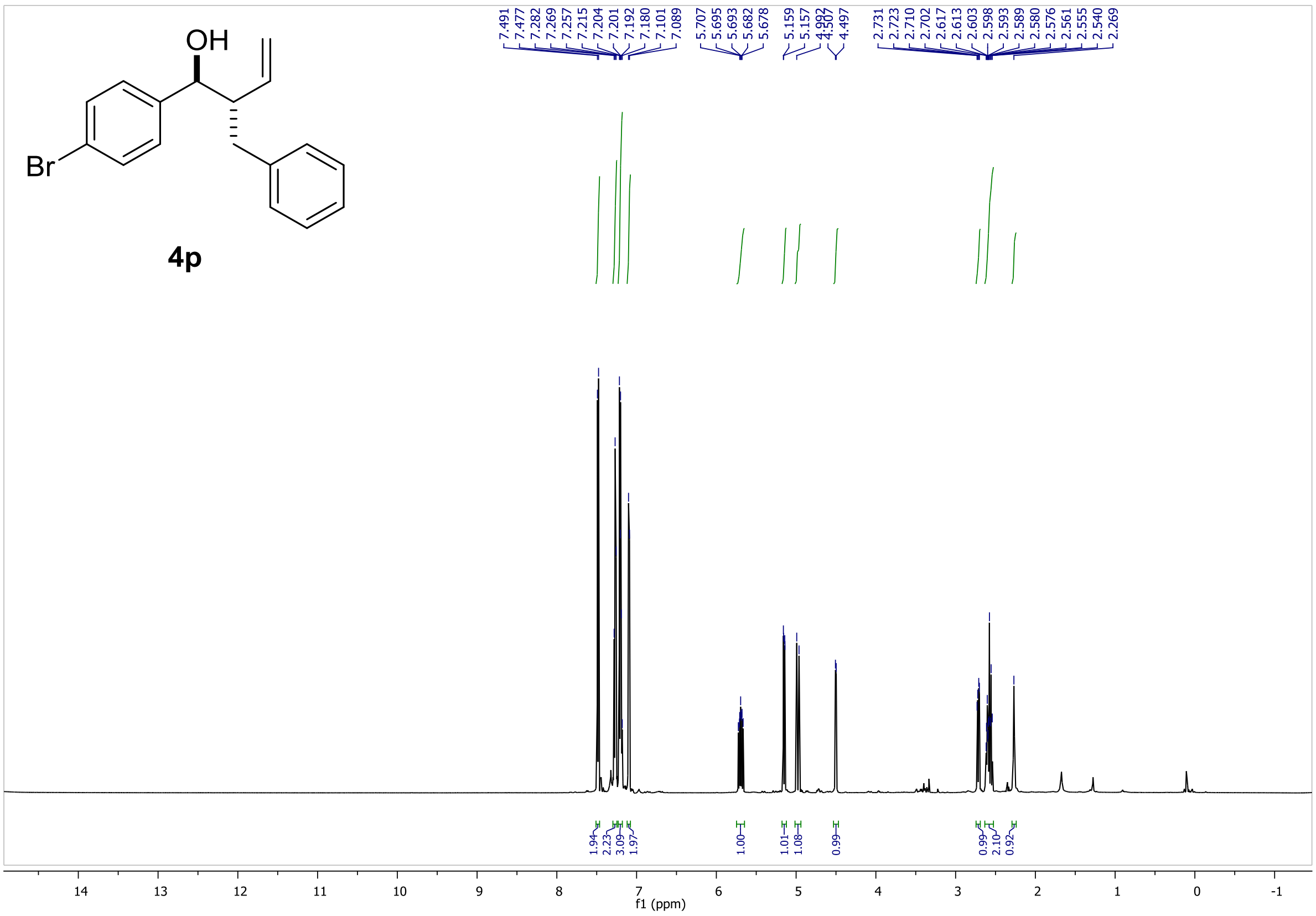

^{13}C NMR, 150 MHz, $CDCl_3$:

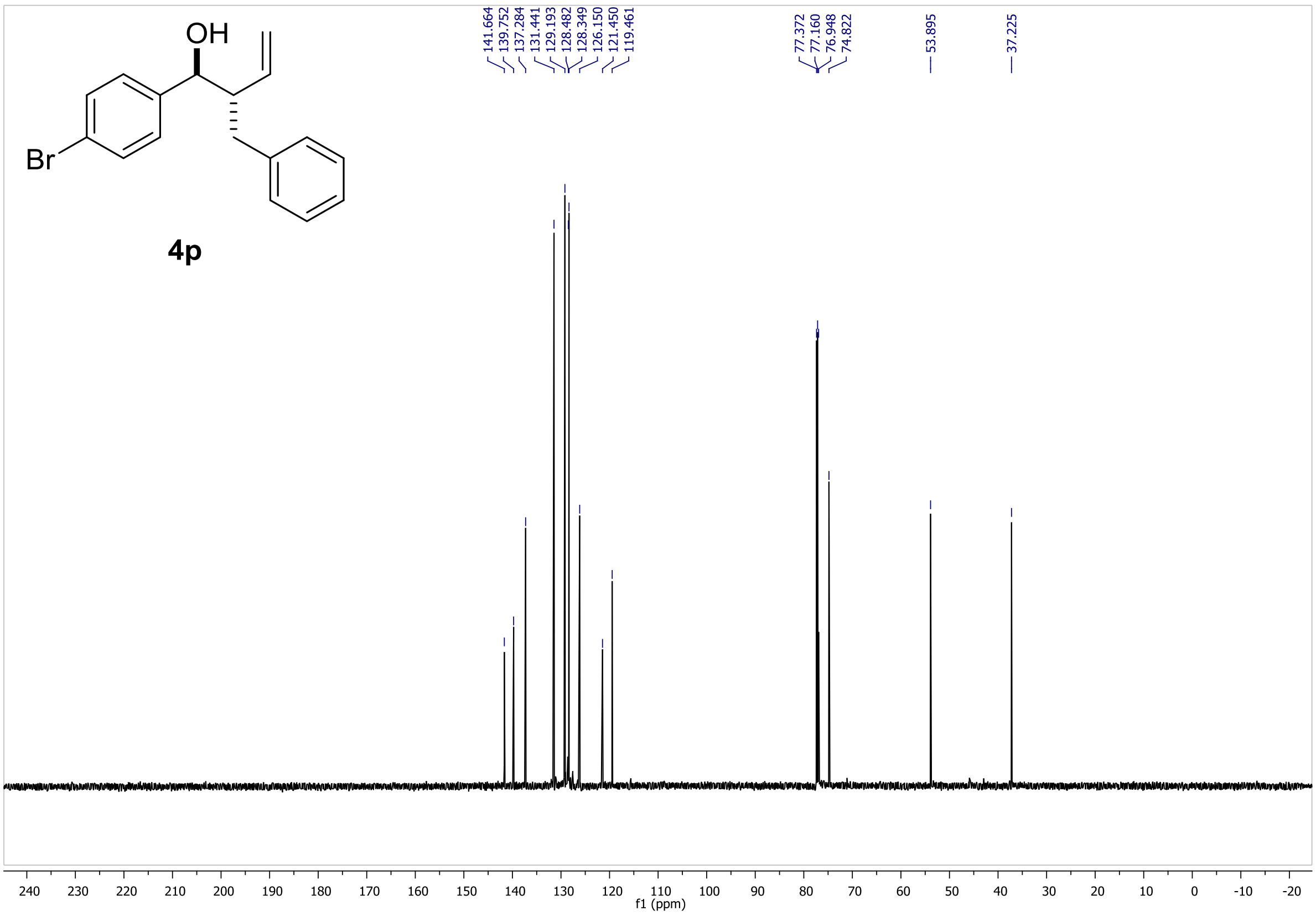

***tert*-butyl (2-(2-(4-fluorophenyl)-1-hydroxybut-3-en-1-yl)phenyl)carbamate (4q):**

^{1}H NMR, 600 MHz, $CDCl_3$:

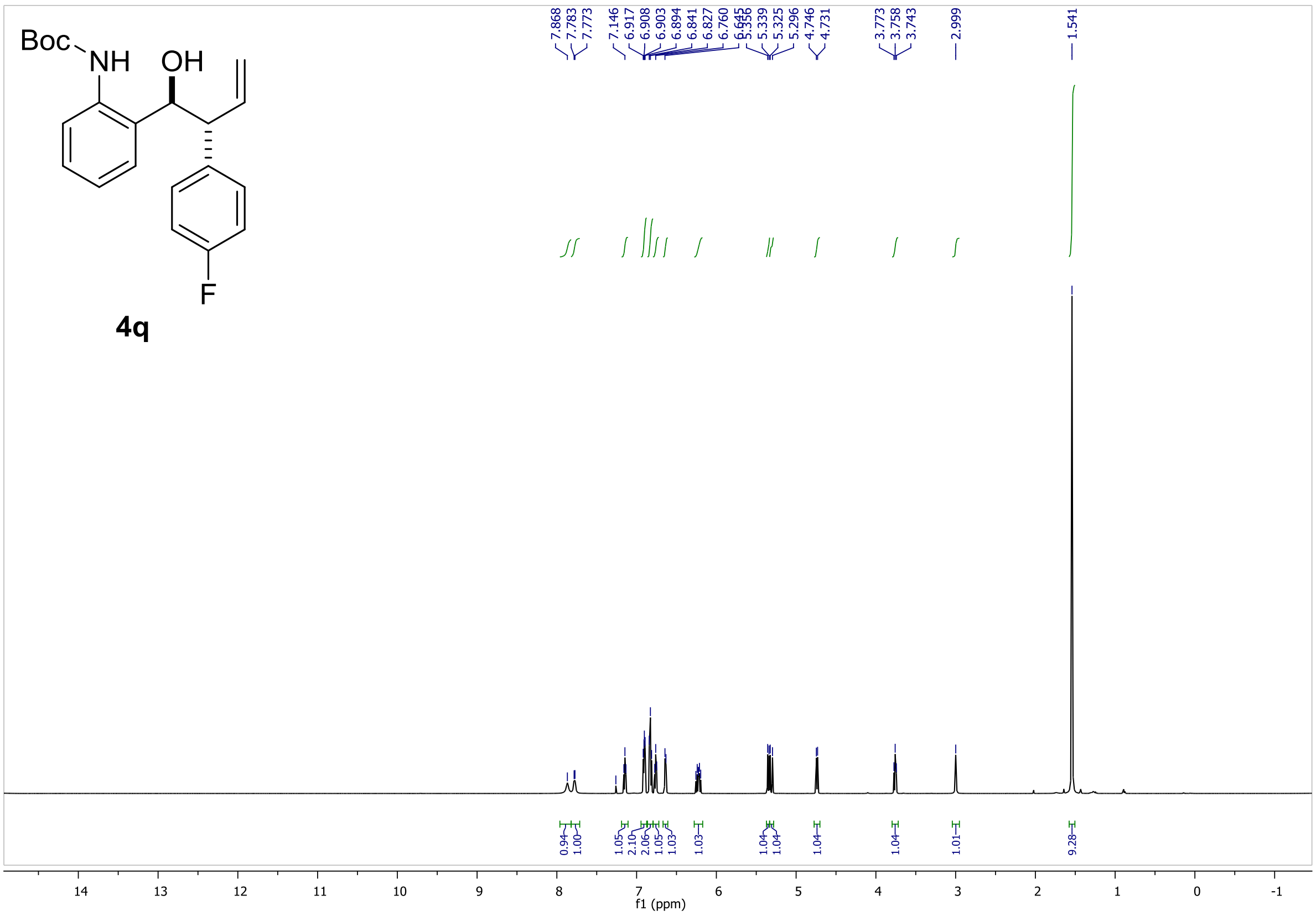

^{13}C NMR, 150 MHz, $CDCl_3$:

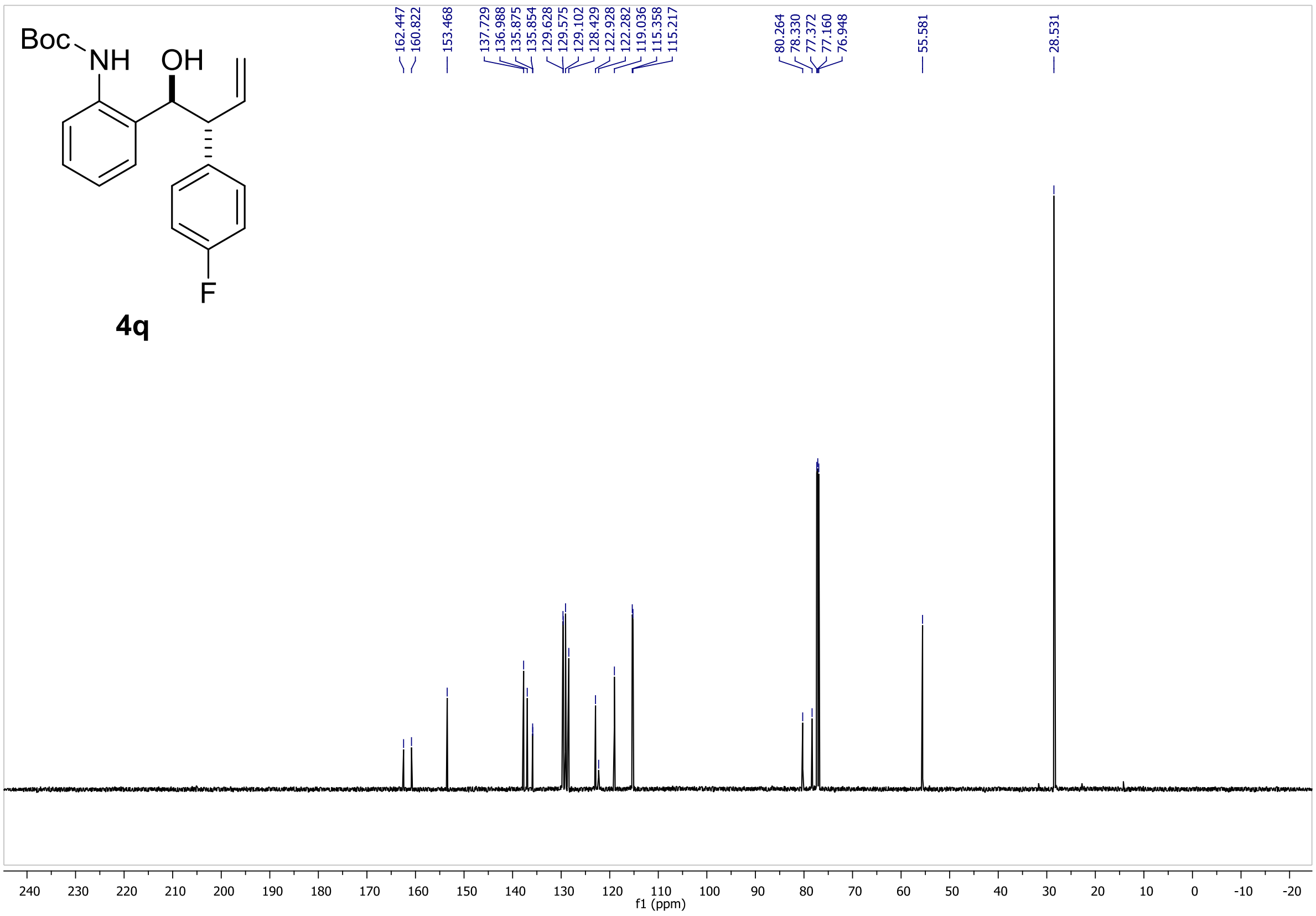

^{19}F NMR, 376 MHz, $CDCl_3$:

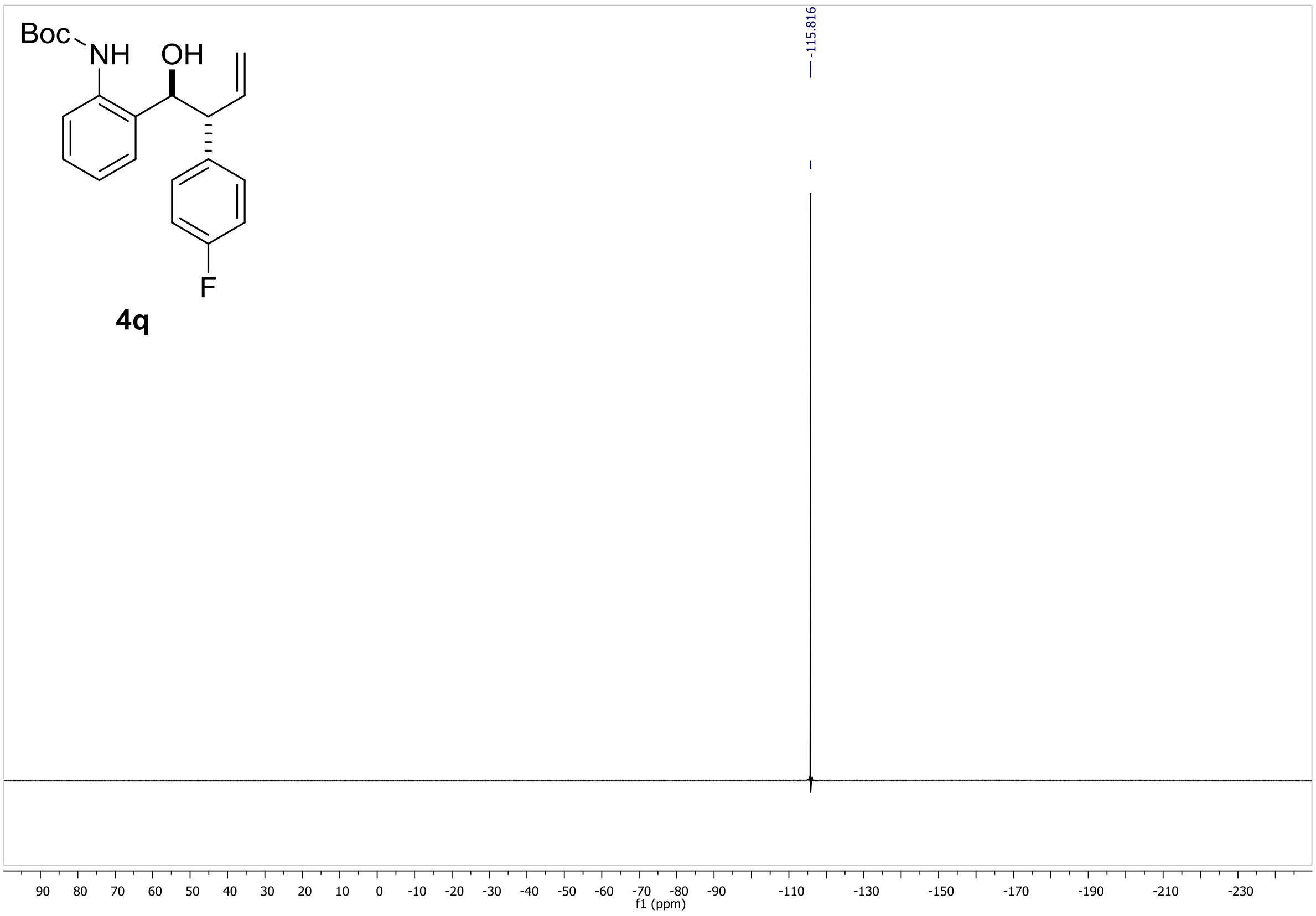

1-(5-bromo-2-chlorophenyl)-2-(4-fluorophenyl)but-3-en-1-ol (4r):

^{1}H NMR, 600 MHz, $CDCl_3$:

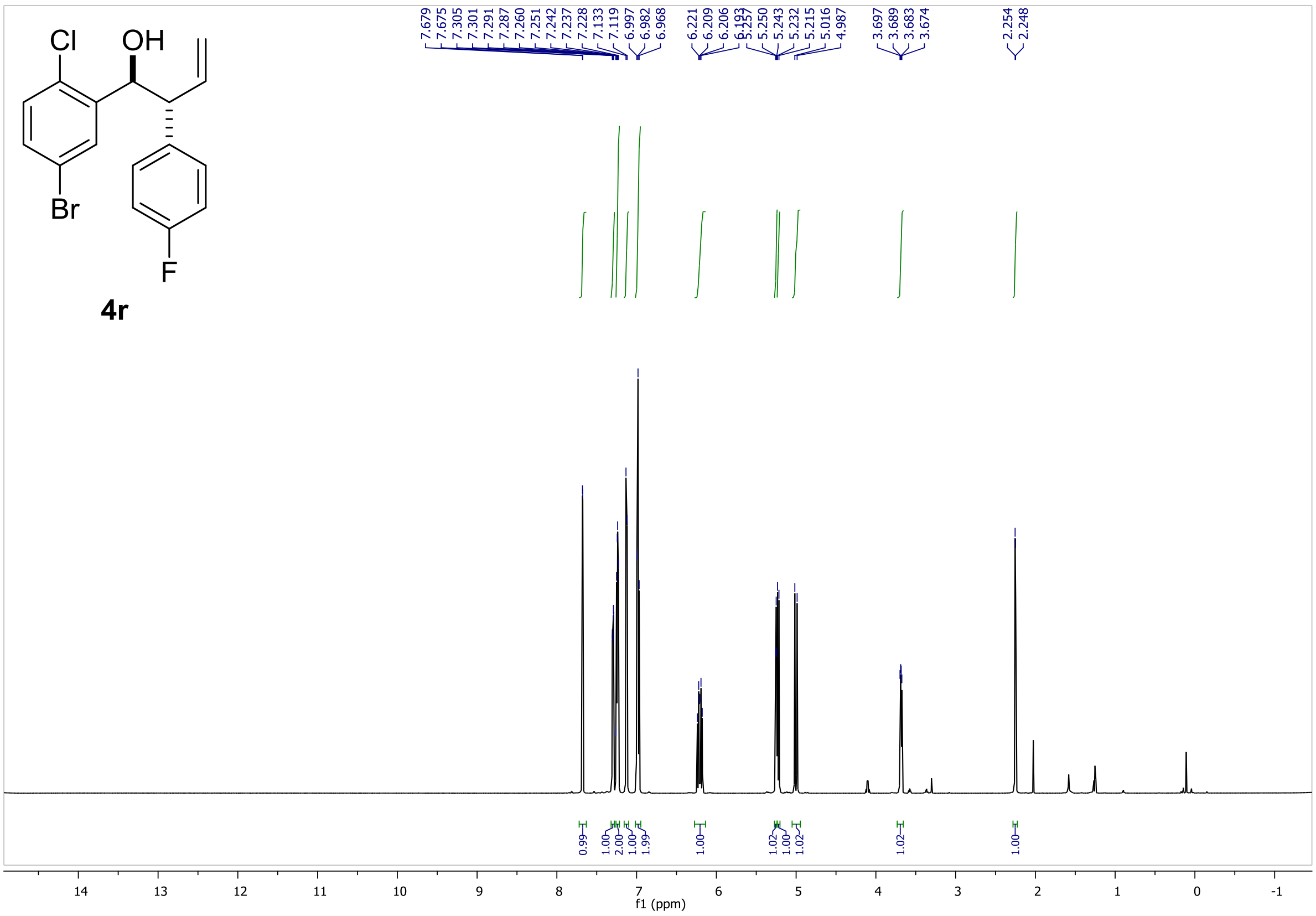

^{13}C NMR, 150 MHz, $CDCl_3$:

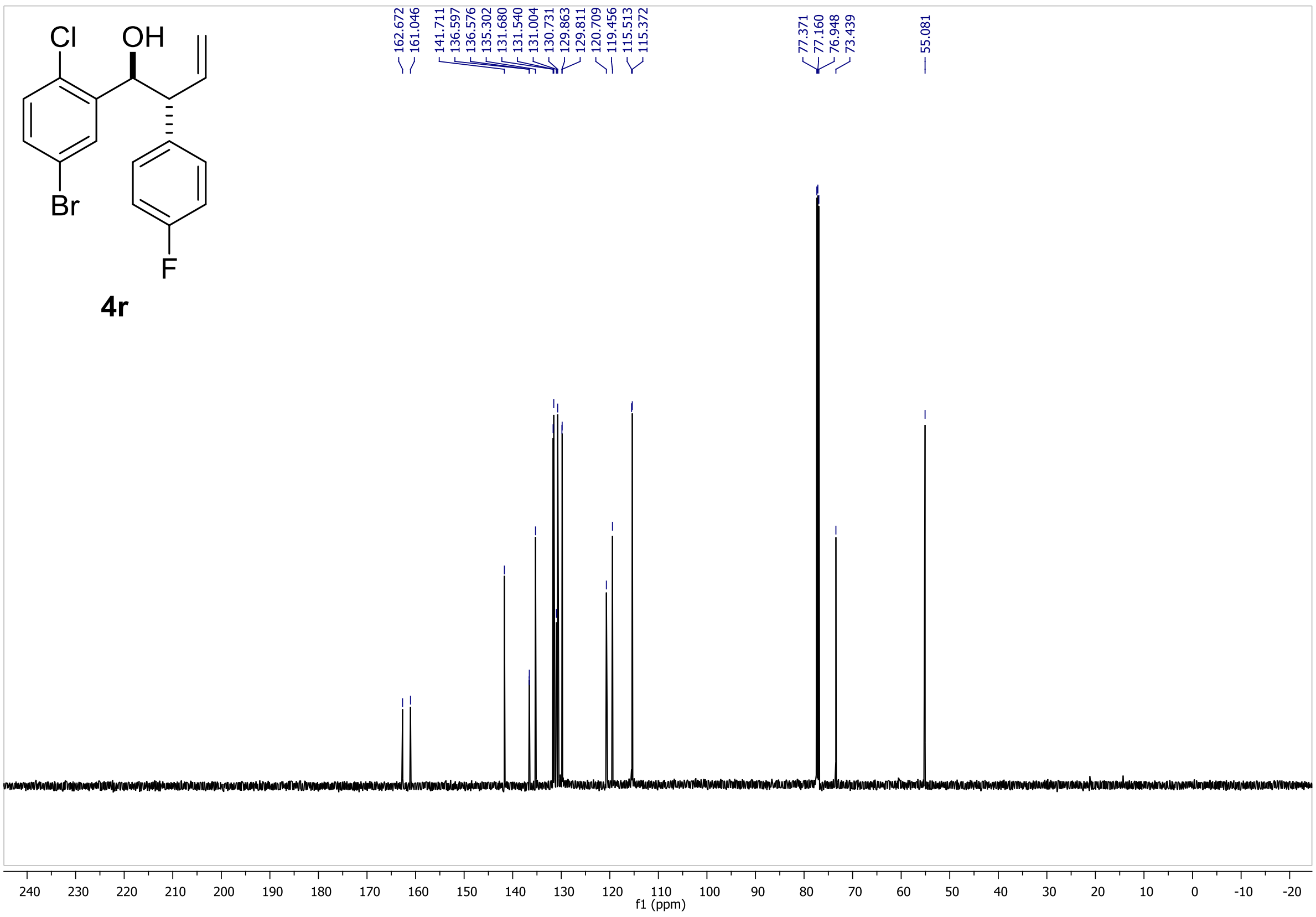

^{19}F NMR, 376 MHz, CDCl$_3$:

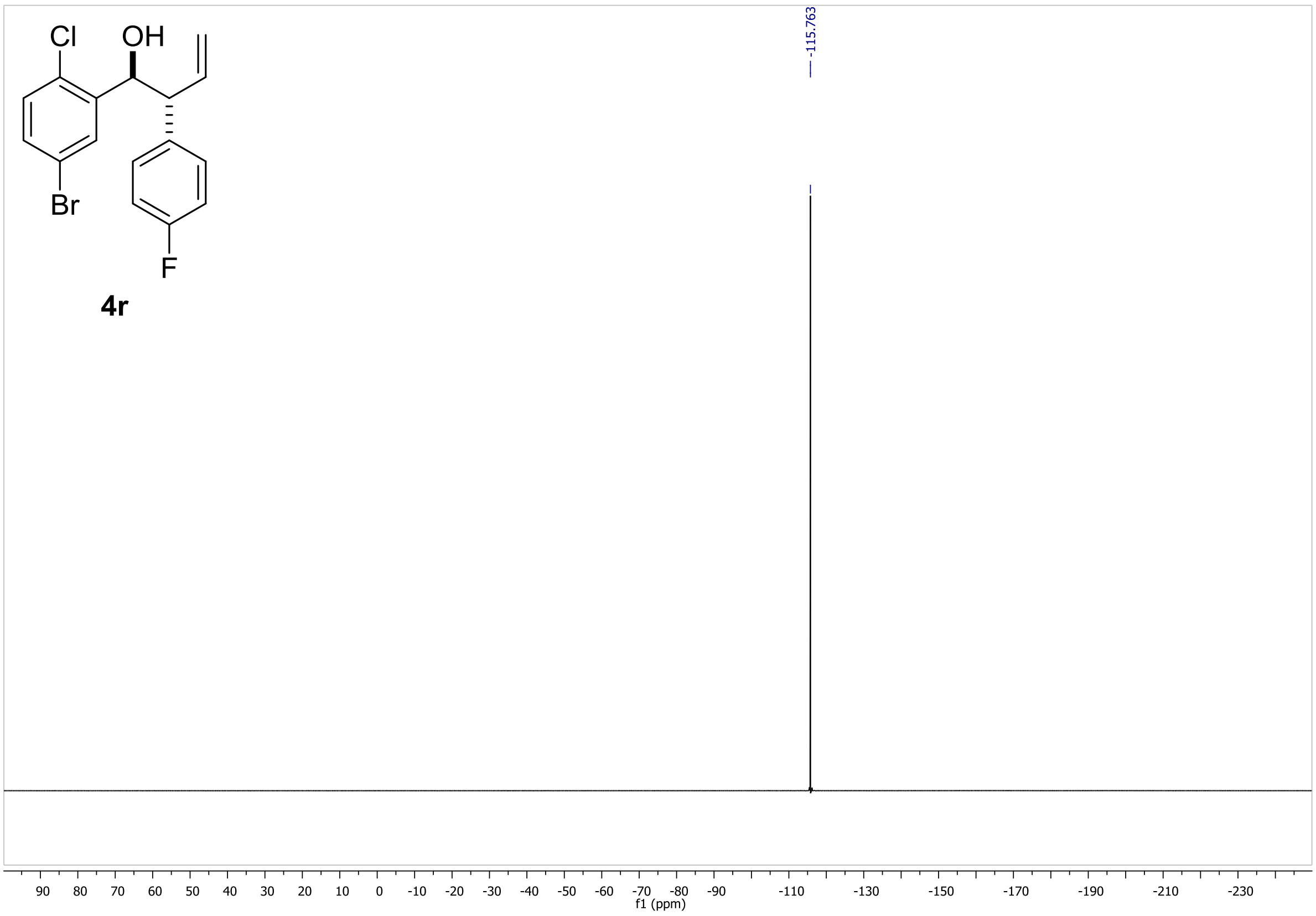

1-(4-(thiophen-2-yl)phenyl)-2-(4-(trifluoromethyl)phenyl)but-3-en-1-ol (4s):

^{1}H NMR, 600 MHz, CDCl$_3$:

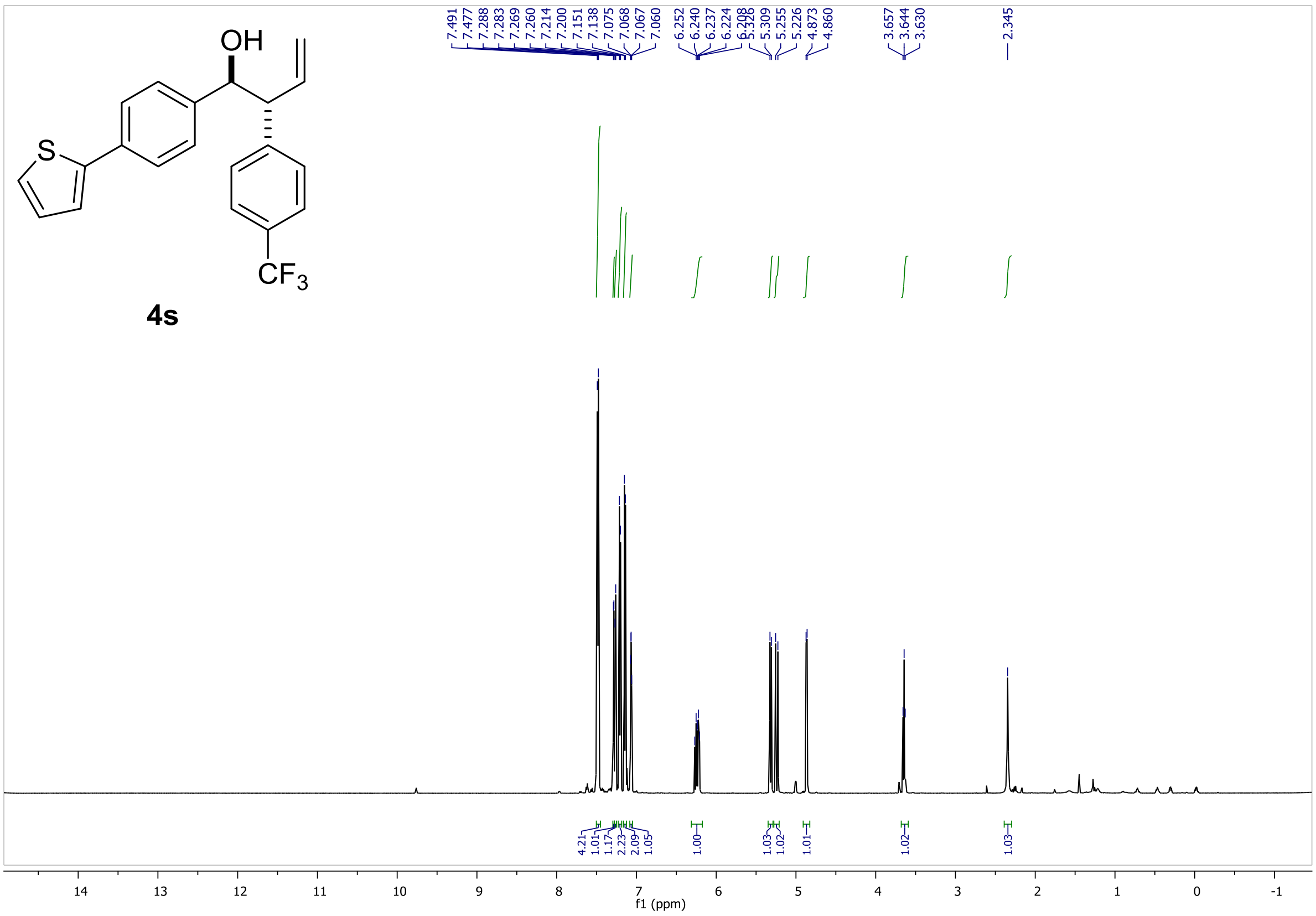

^{13}C NMR, 150 MHz, CDCl$_3$:

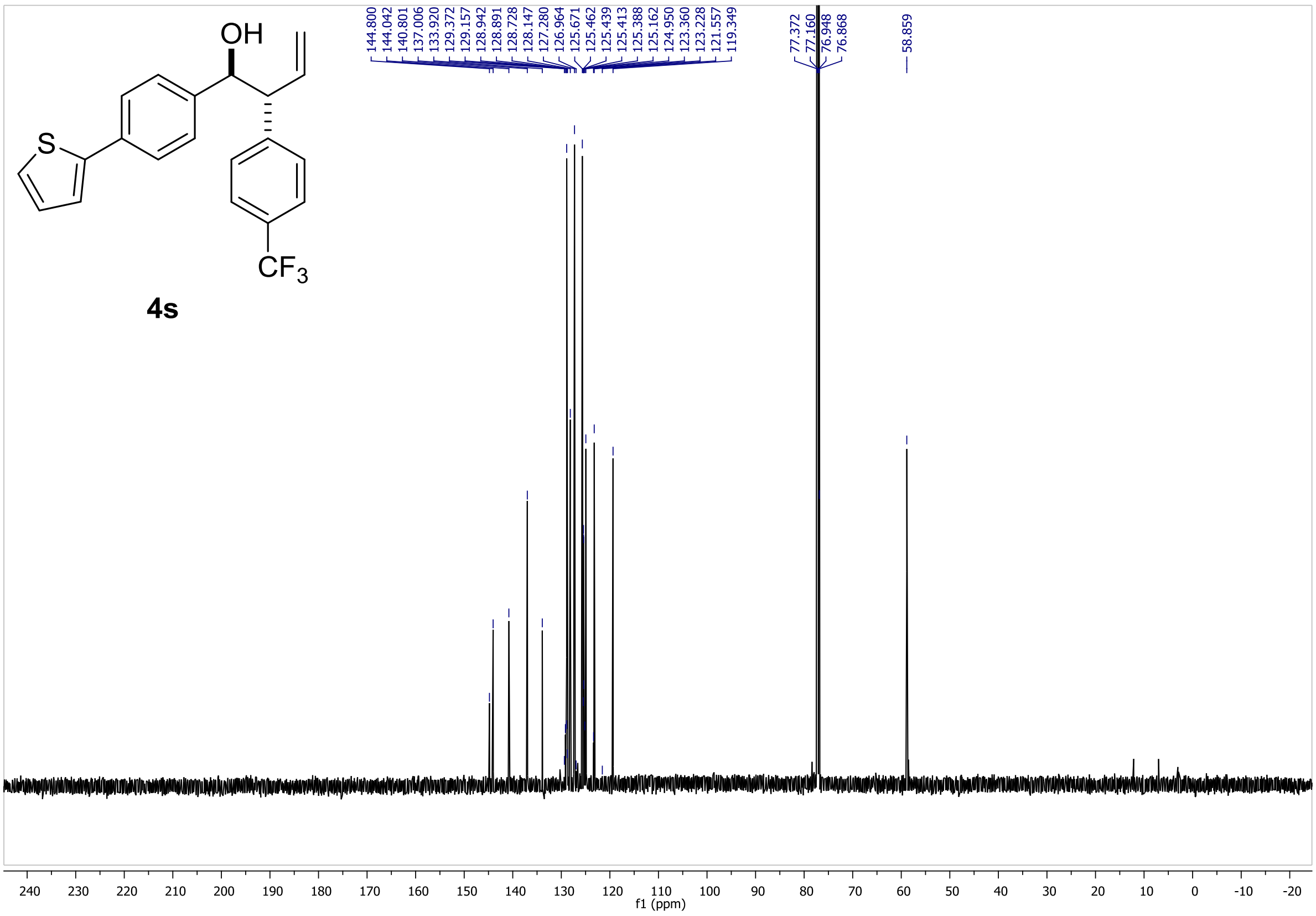

^{19}F NMR, 376 MHz, $CDCl_3$:

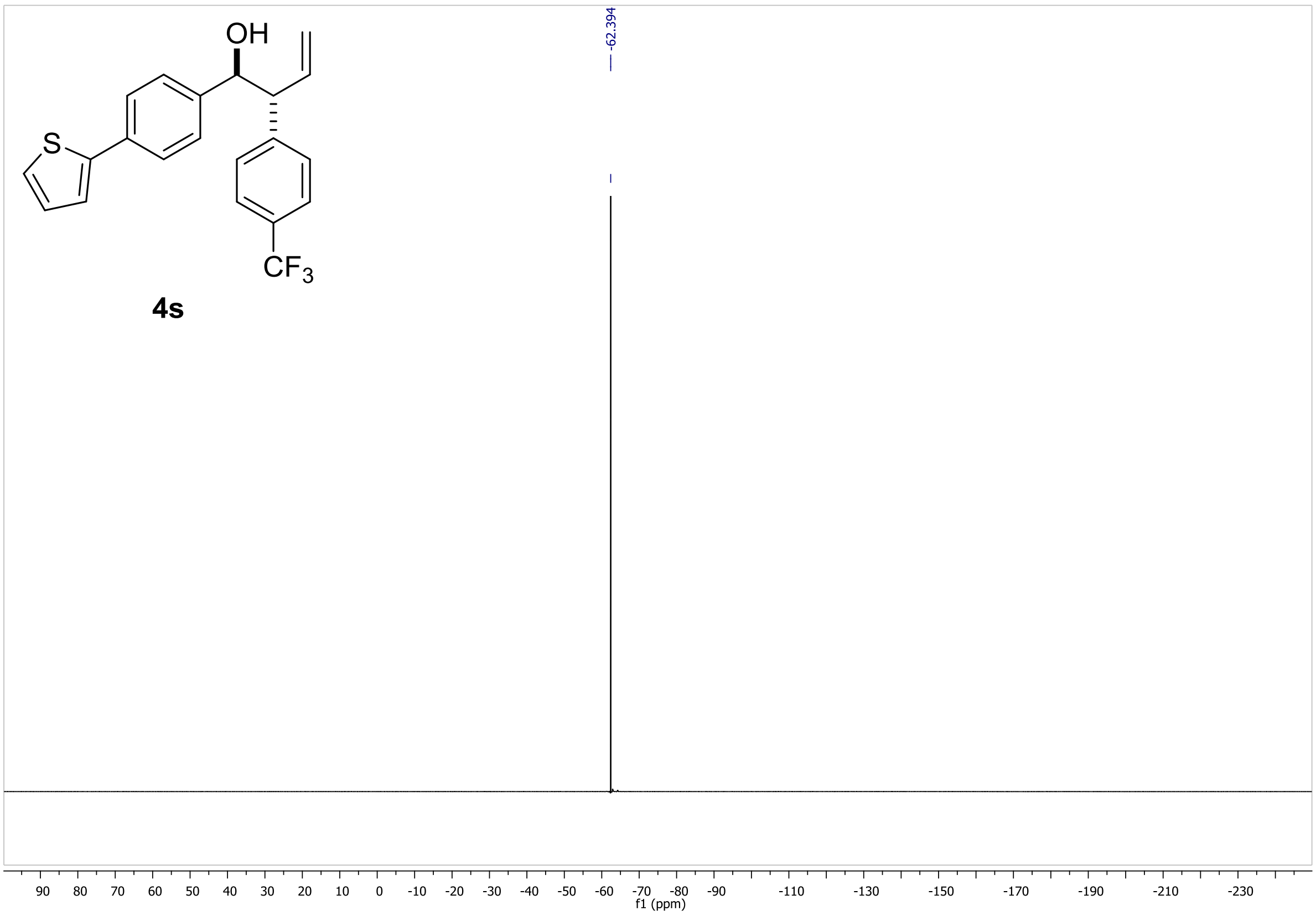

2-(4-methoxyphenyl)-1-(perfluorophenyl)but-3-en-1-ol (4t):

¹H NMR, 600 MHz, CDCl₃:

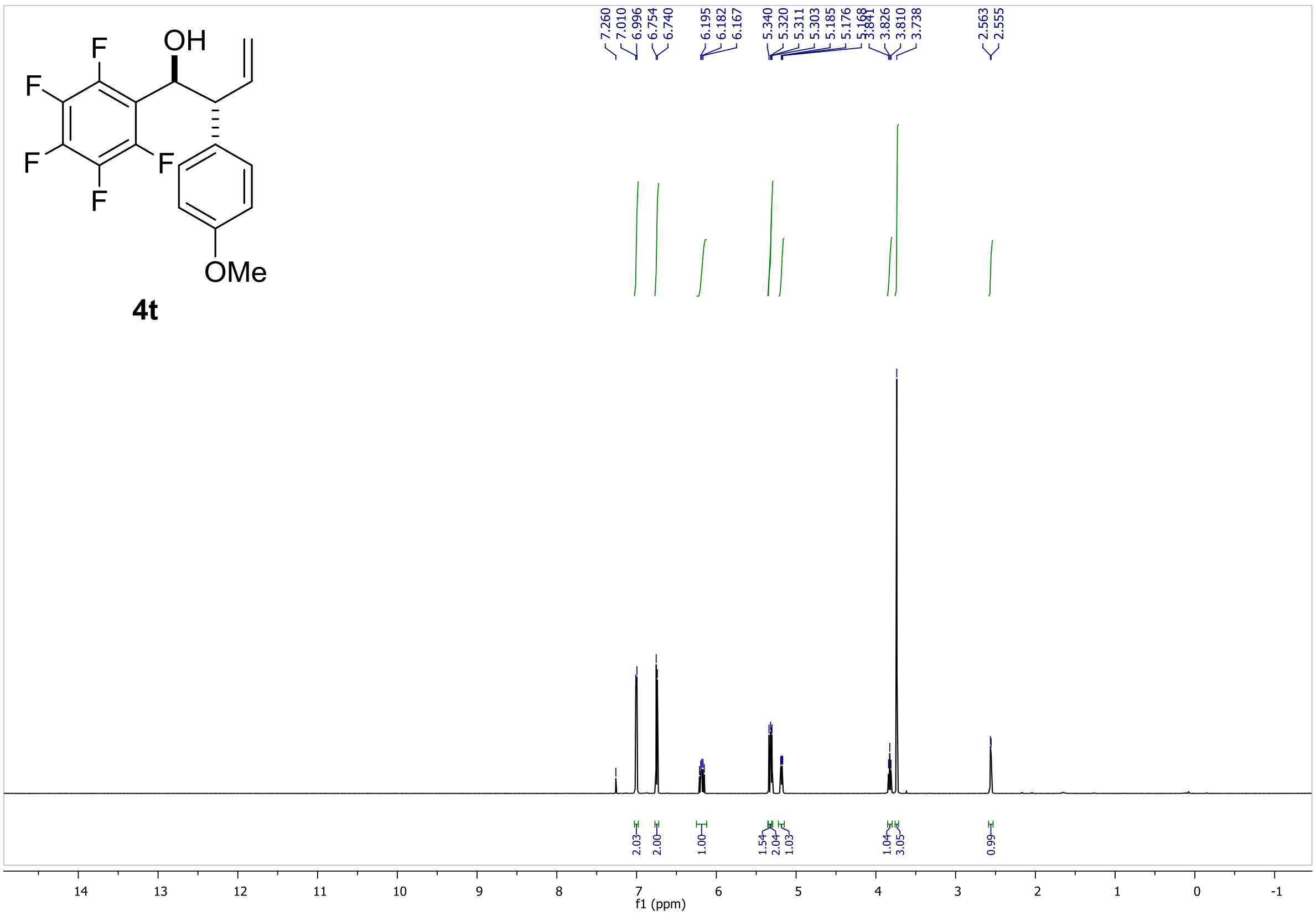

¹³C NMR, 150 MHz, CDCl₃:

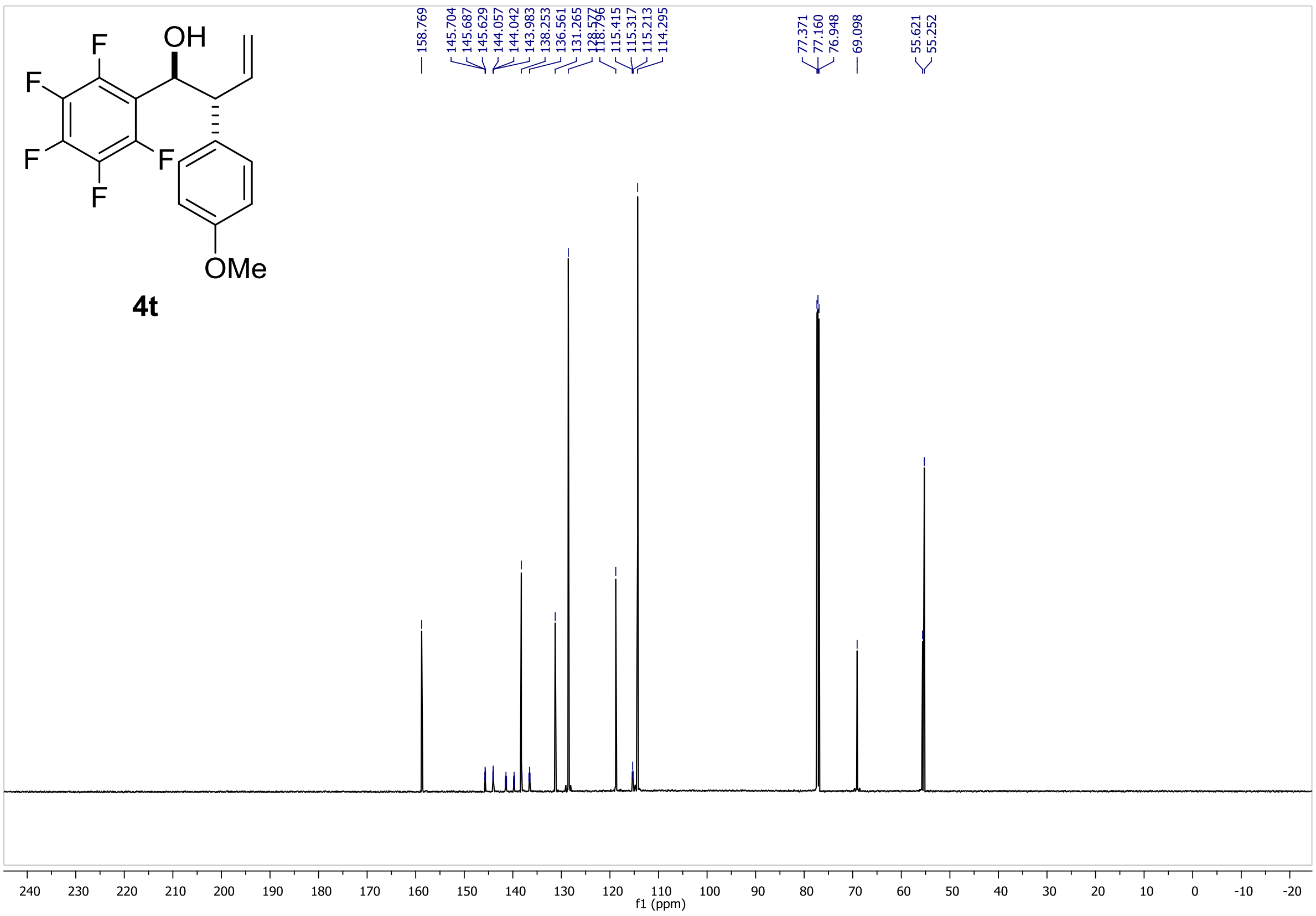

^{19}F NMR, 376 MHz, CDCl$_3$:

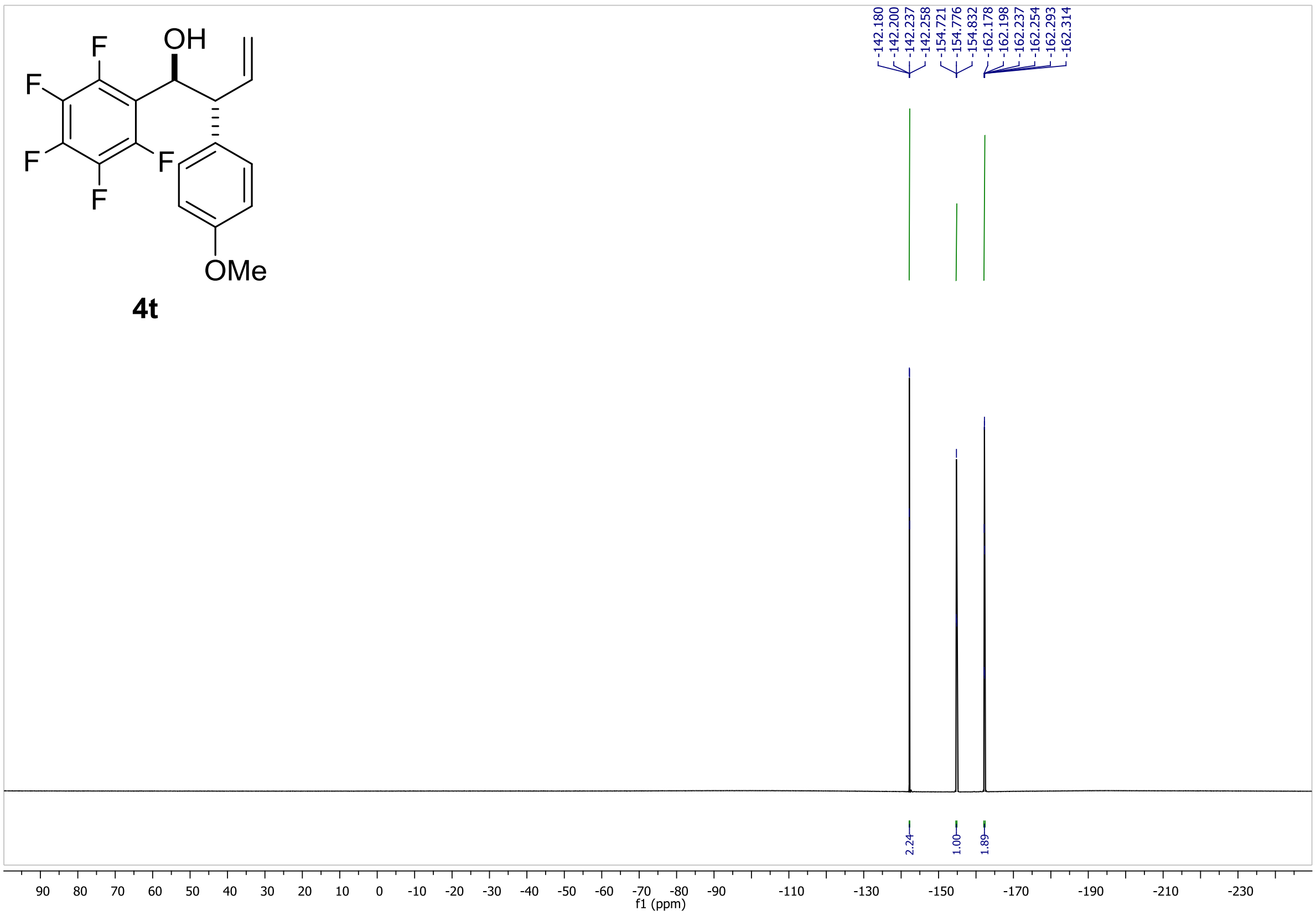

1-(furan-2-yl)-2-(4-methoxyphenyl)but-3-en-1-ol (4u):

^{1}H NMR, 600 MHz, $CDCl_3$:

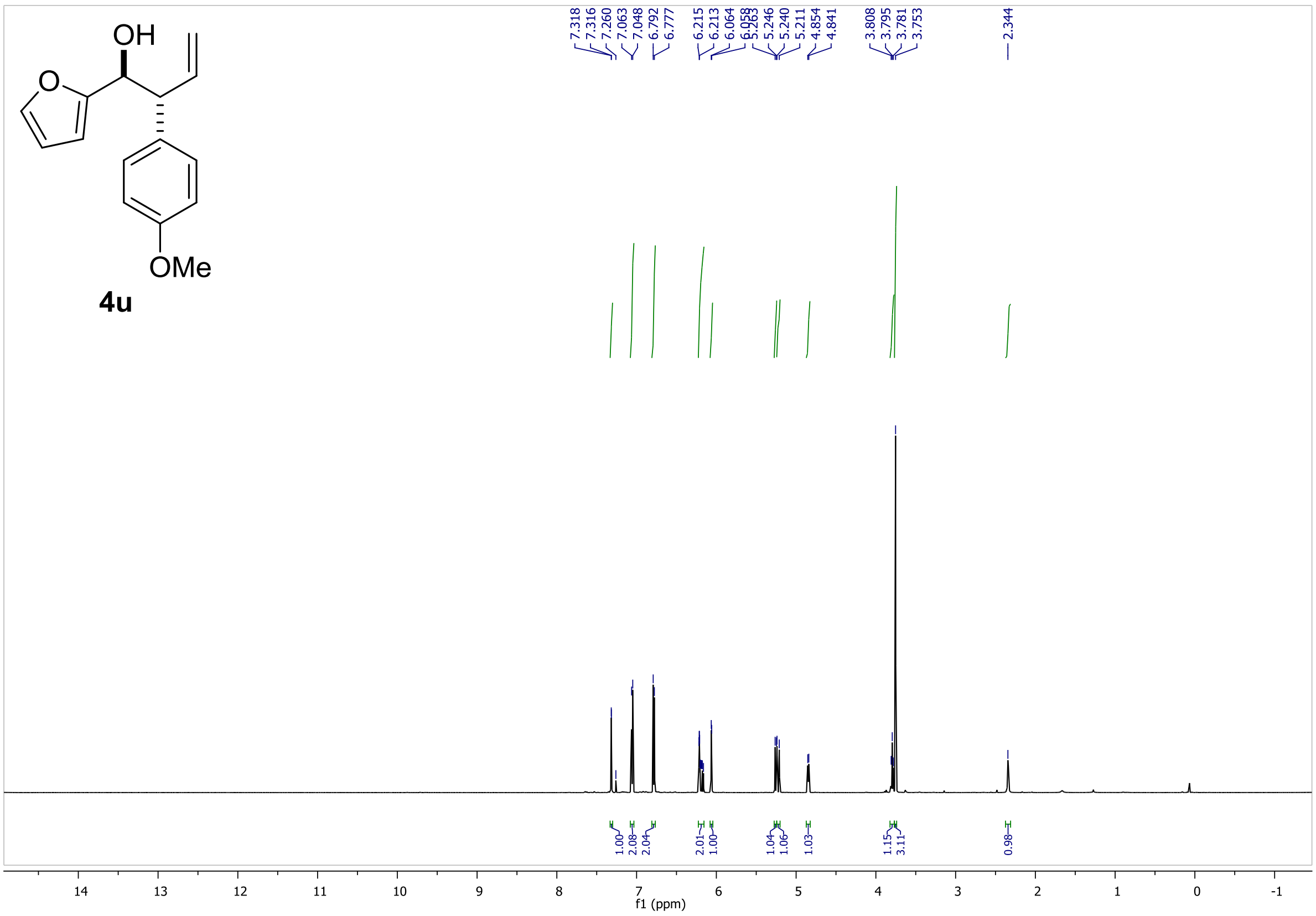

^{13}C NMR, 150 MHz, $CDCl_3$:

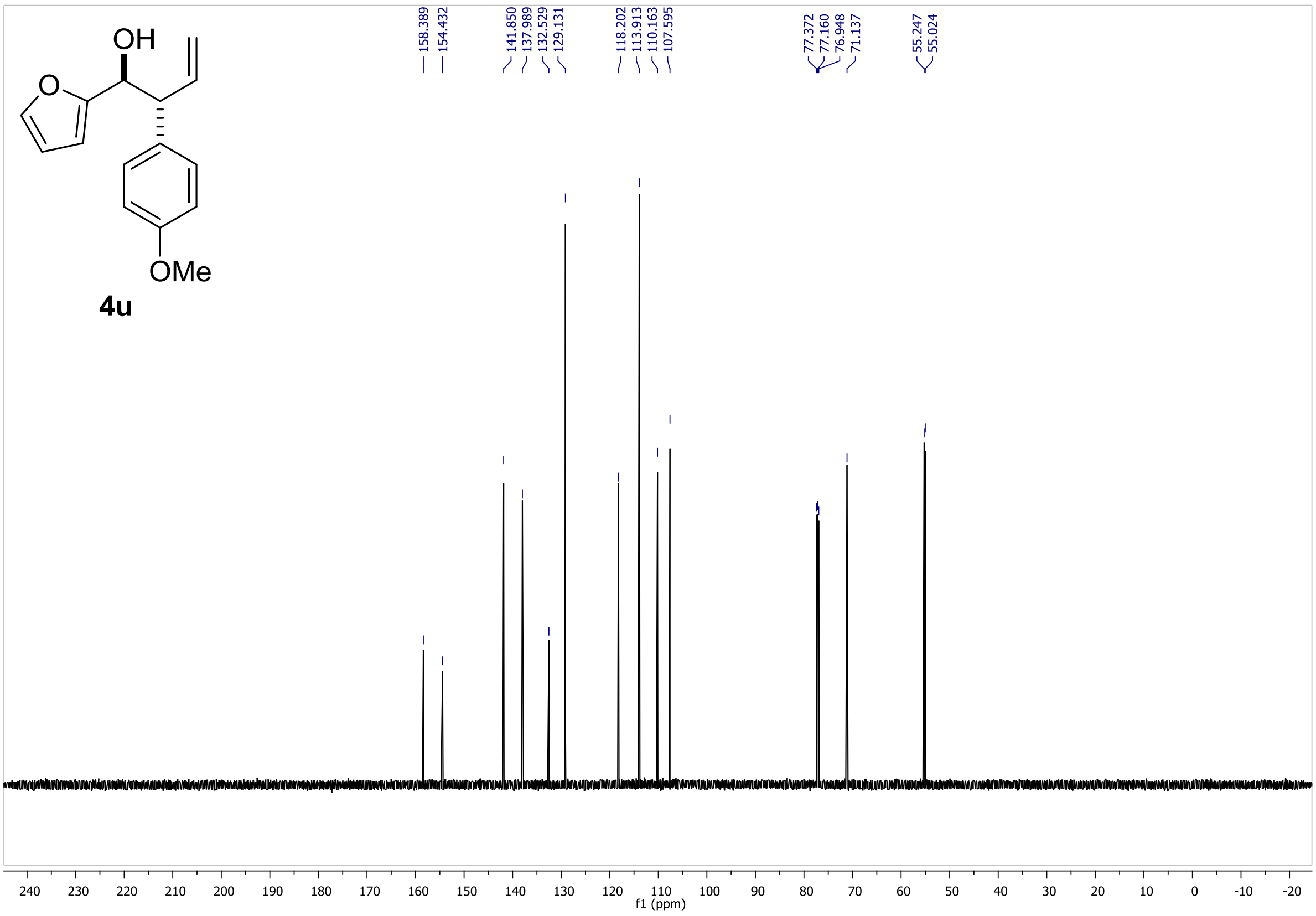

4-(1-hydroxy-3-phenylbut-3-en-1-yl)benzonitrile (4v):

^{1}H NMR, 600 MHz, $CDCl_3$:

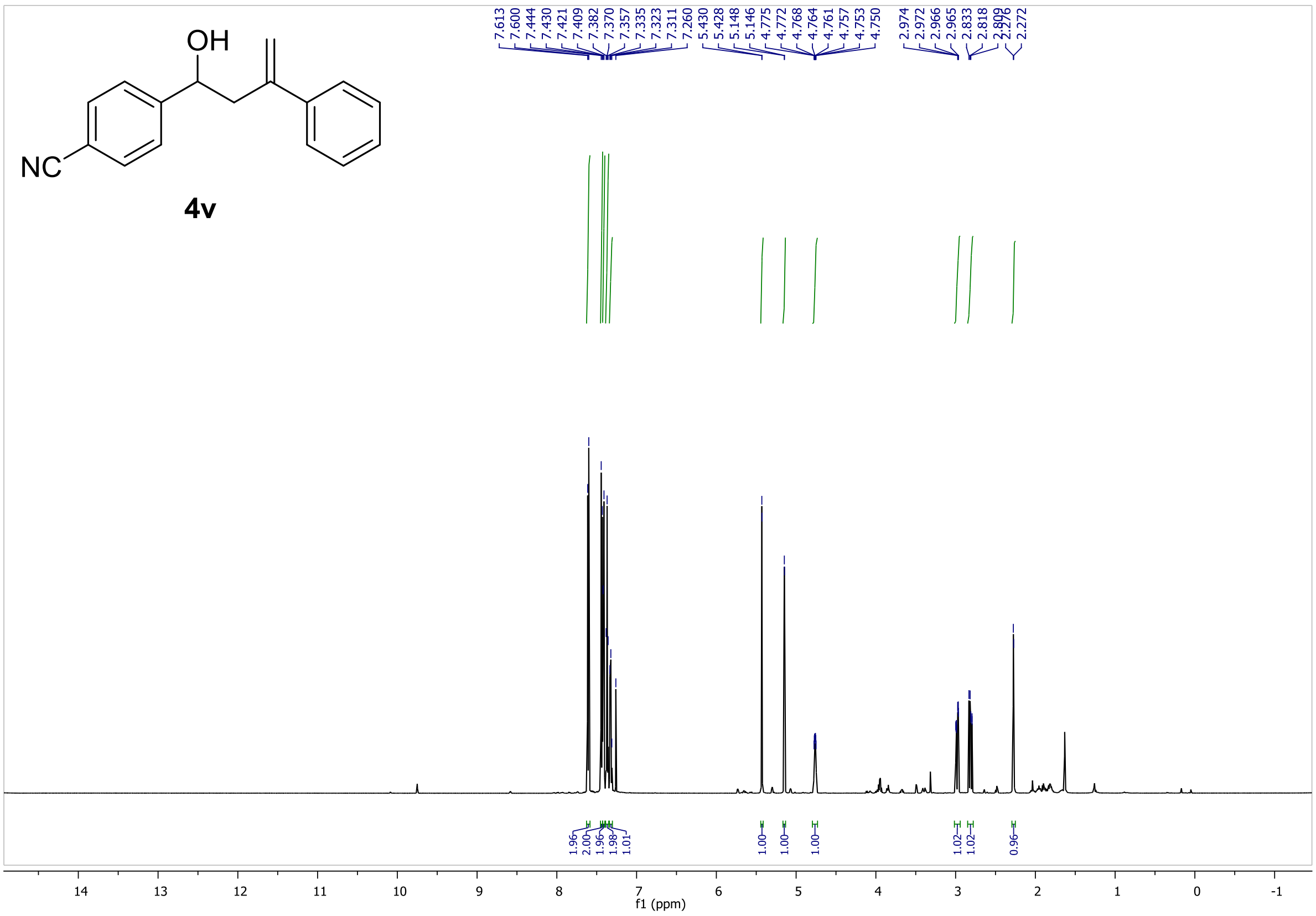

^{13}C NMR, 150 MHz, $CDCl_3$:

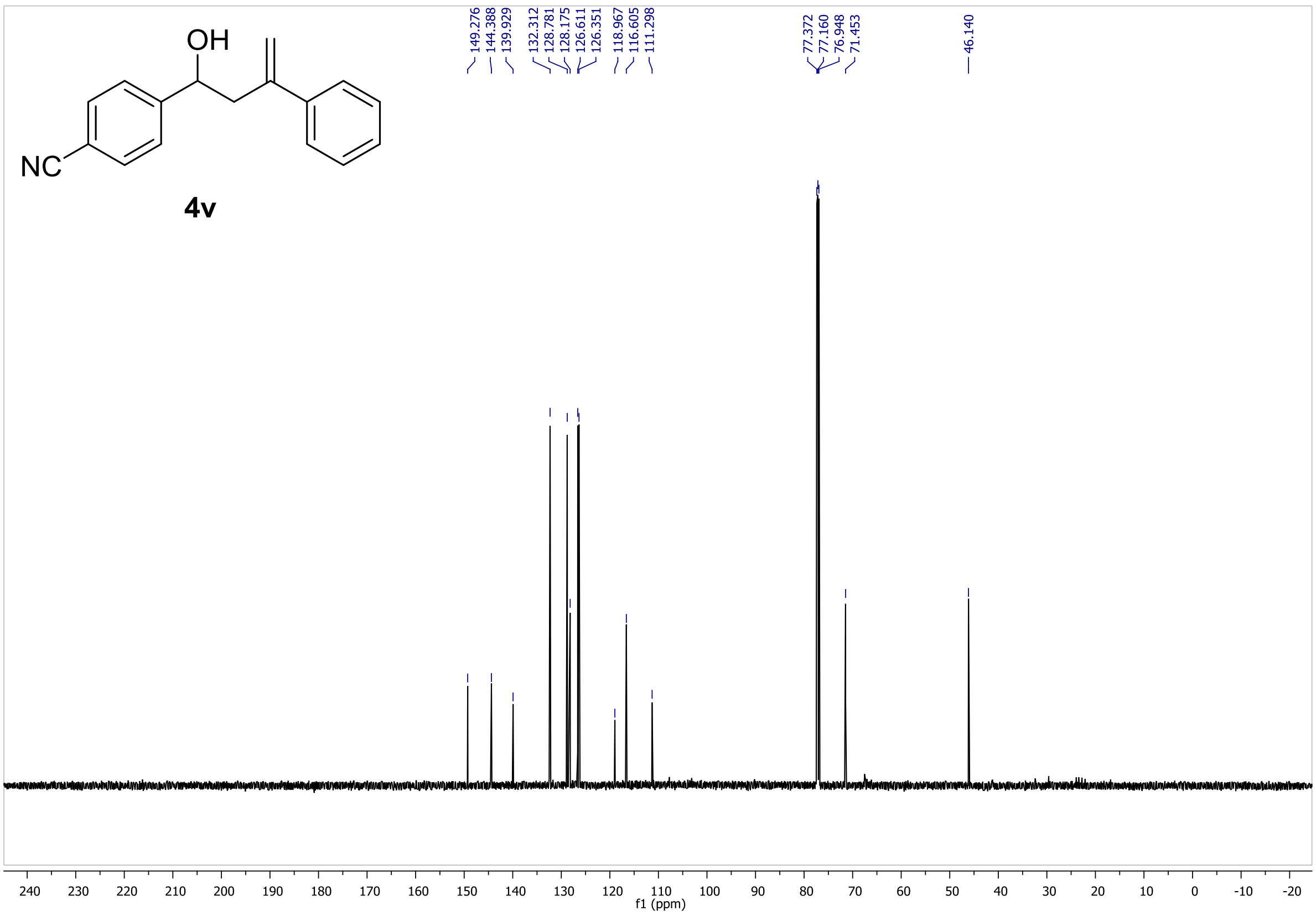

1,5-diphenylhex-5-en-1-yn-3-ol (4w):

^{1}H NMR, 600 MHz, $CDCl_3$:

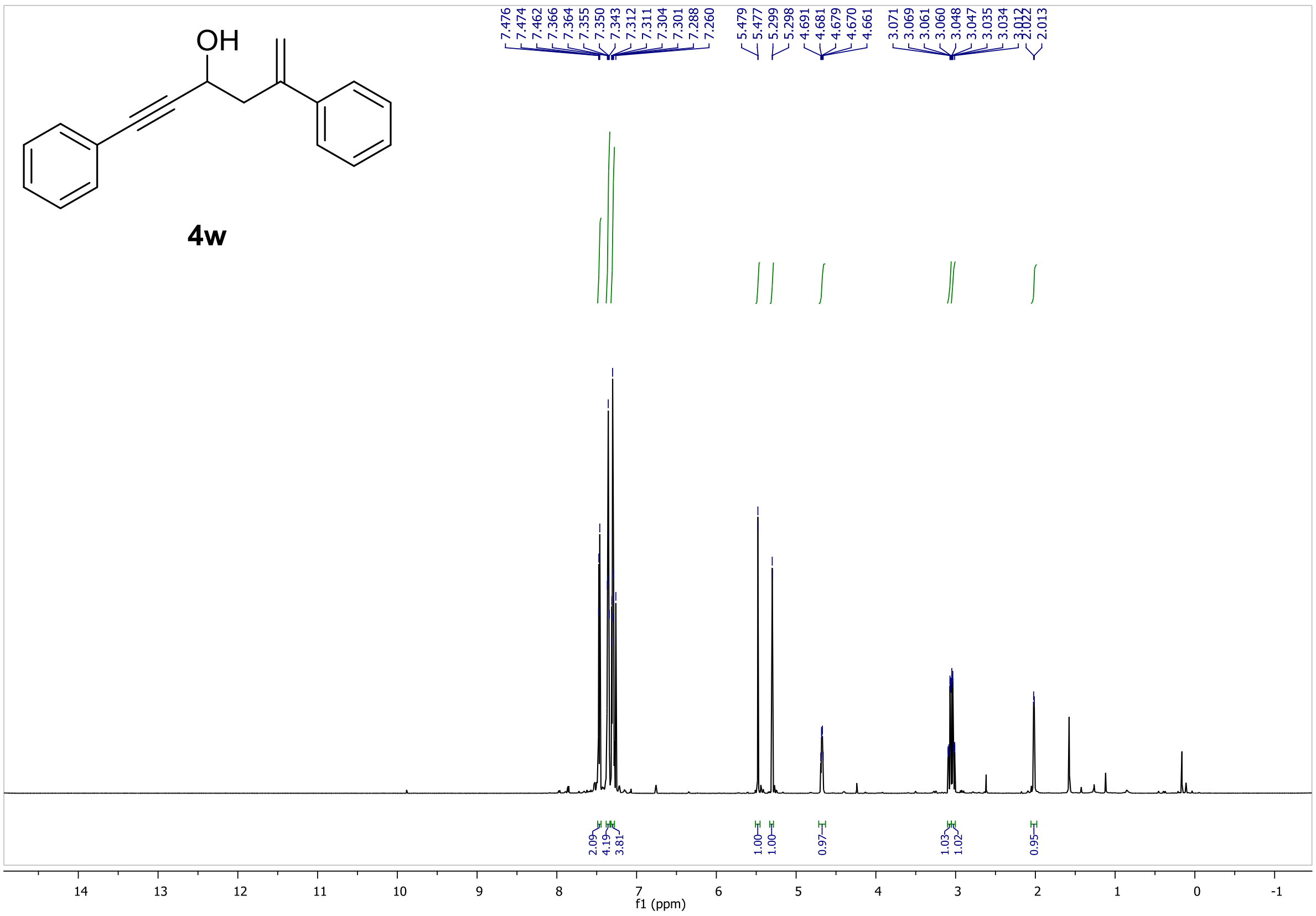

^{13}C NMR, 150 MHz, $CDCl_3$:

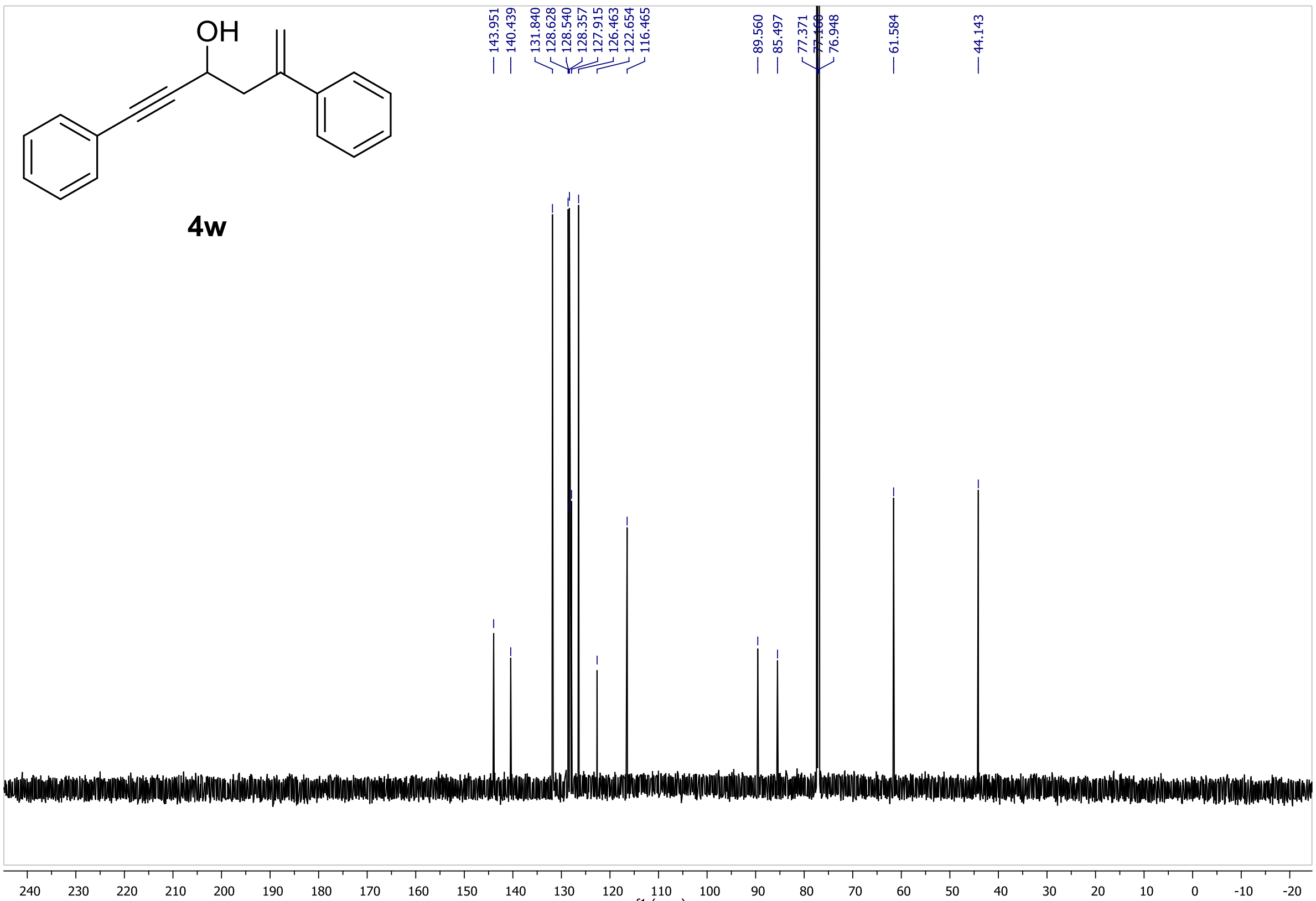

1-(3-(-1-hydroxy-2-(*p*-tolyl)but-3-en-1-yl)-1*H*-indol-1-yl)ethan-1-one (4x):

^{1}H NMR, 600 MHz, CDCl$_3$:

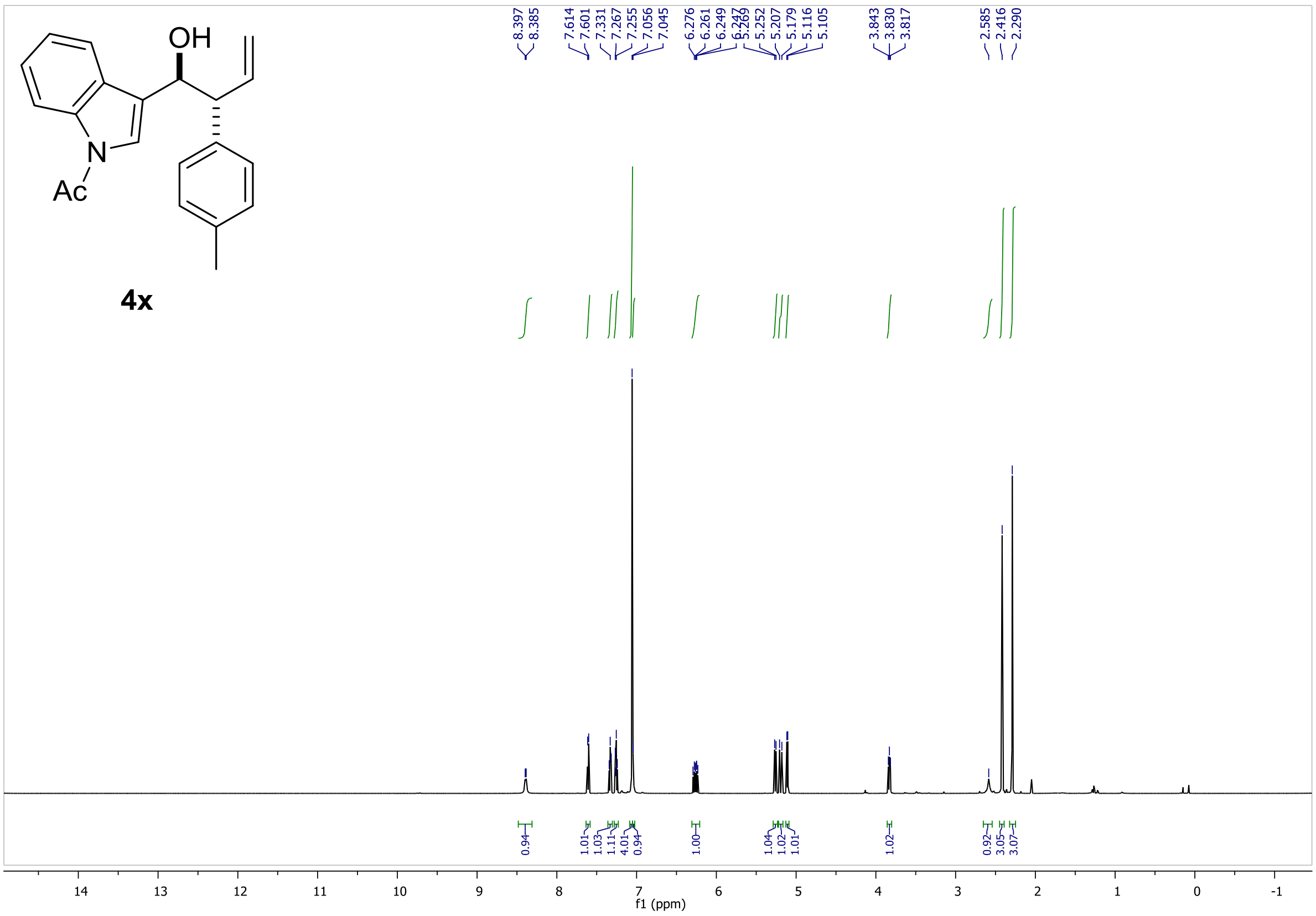

^{13}C NMR, 150 MHz, CDCl$_3$:

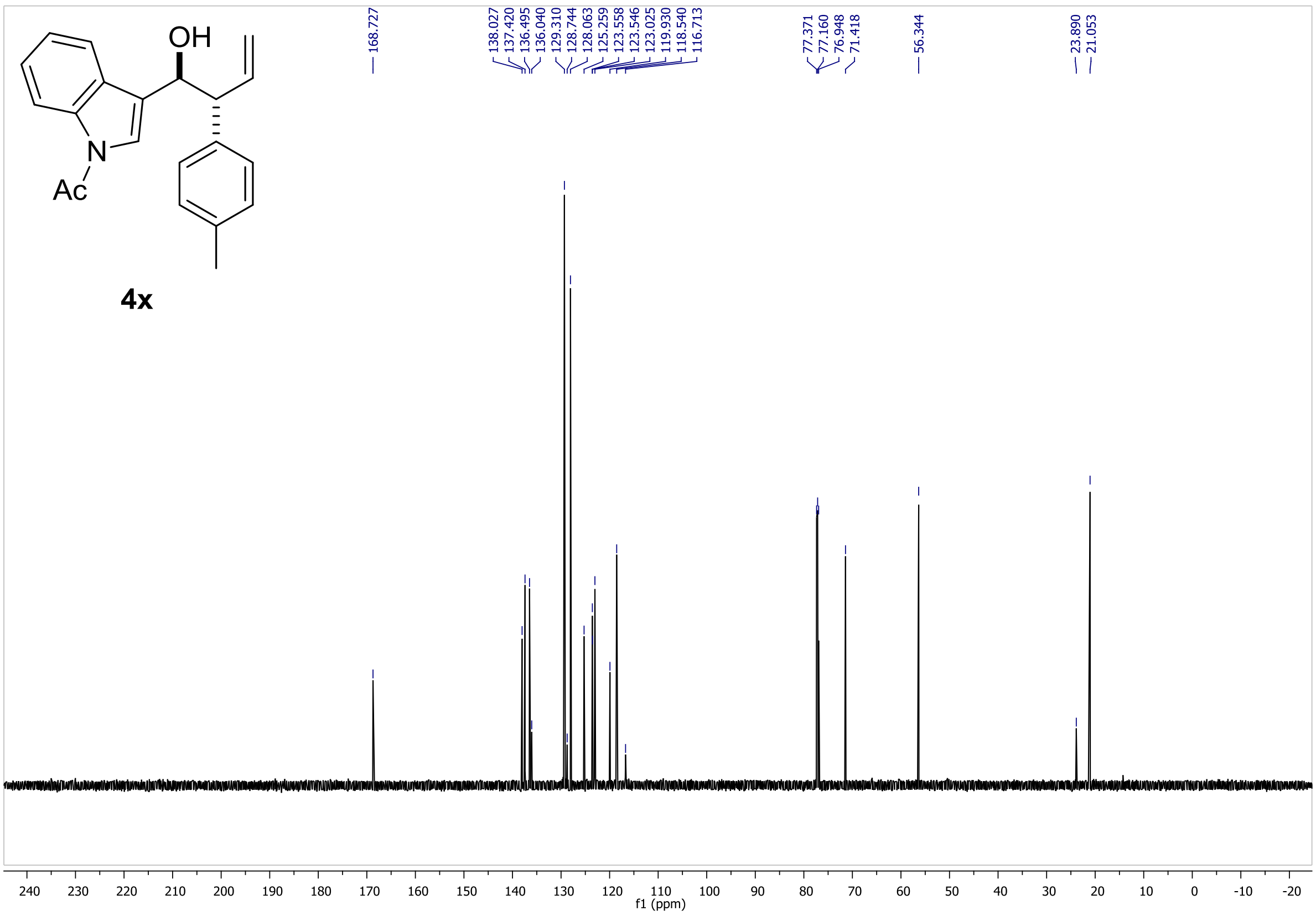

6,10-dimethyl-3-(*p*-tolyl)undeca-1,9-dien-4-ol (4y):

^{1}H NMR, 600 MHz, $CDCl_3$:

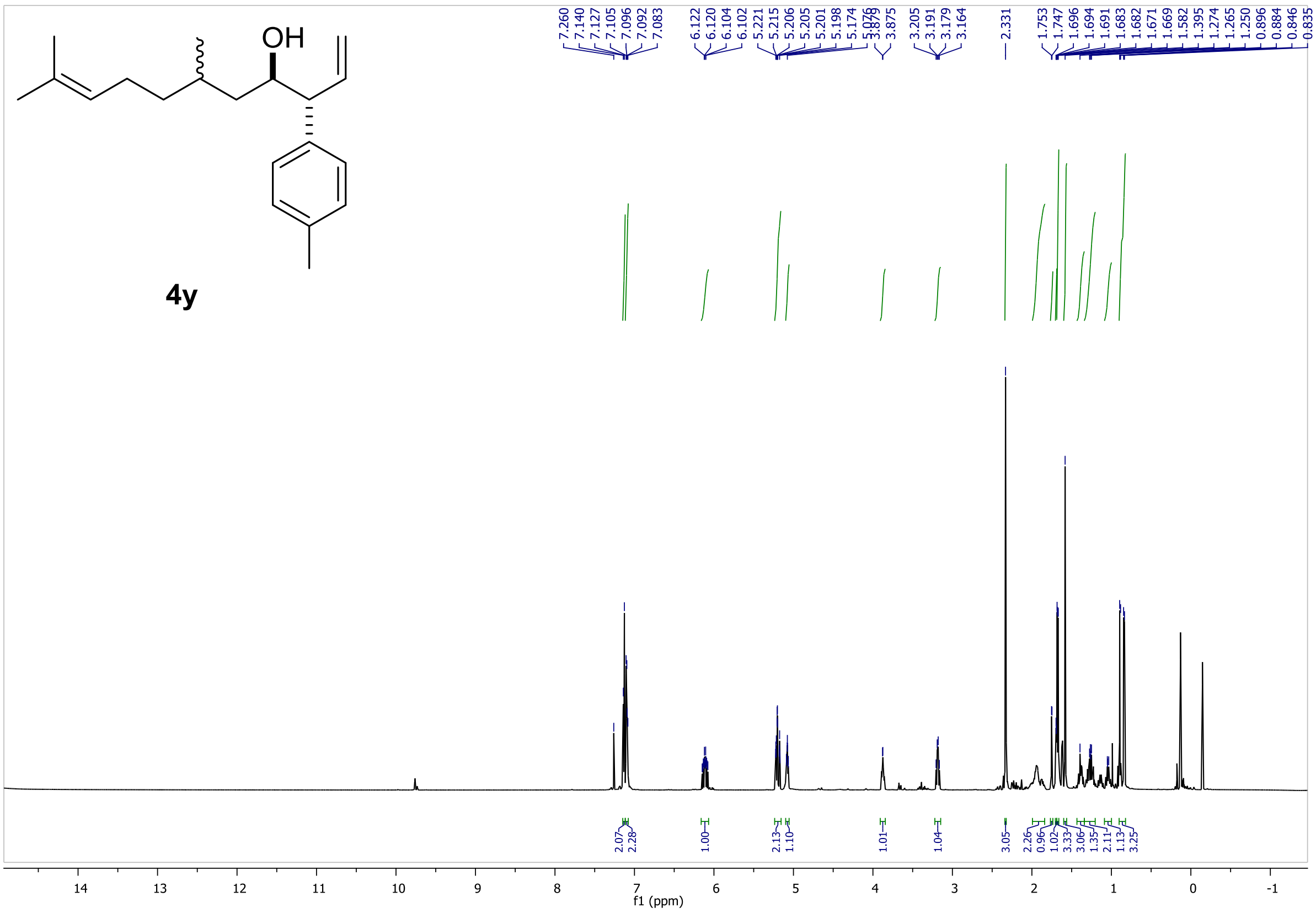

^{13}C NMR, 150 MHz, $CDCl_3$:

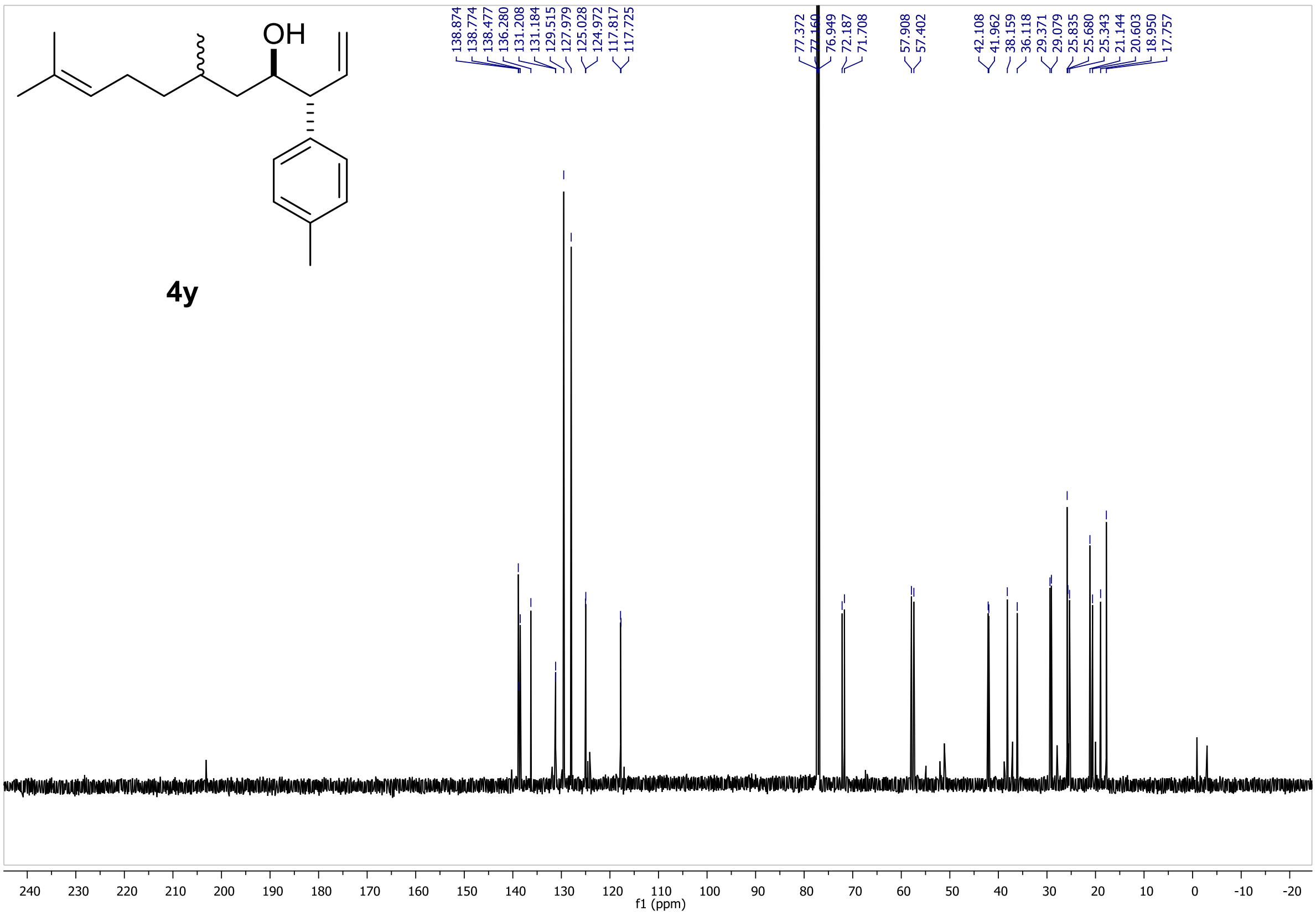

3-benzyl-7-(5,5-dimethyl-1,3-dioxan-2-yl)hept-1-en-4-ol (4z):

^{1}H NMR, 600 MHz, $CDCl_3$:

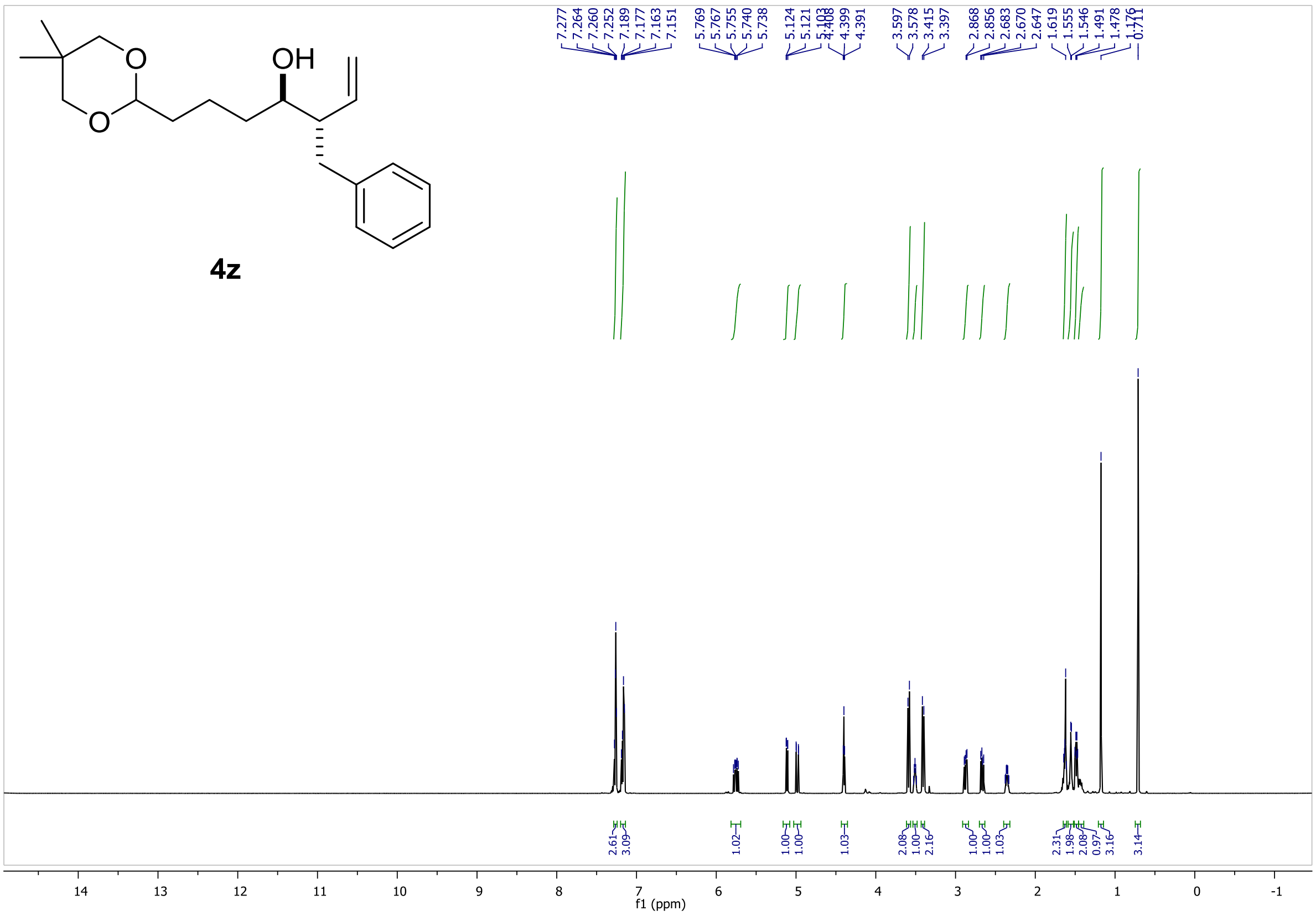

^{13}C NMR, 150 MHz, $CDCl_3$:

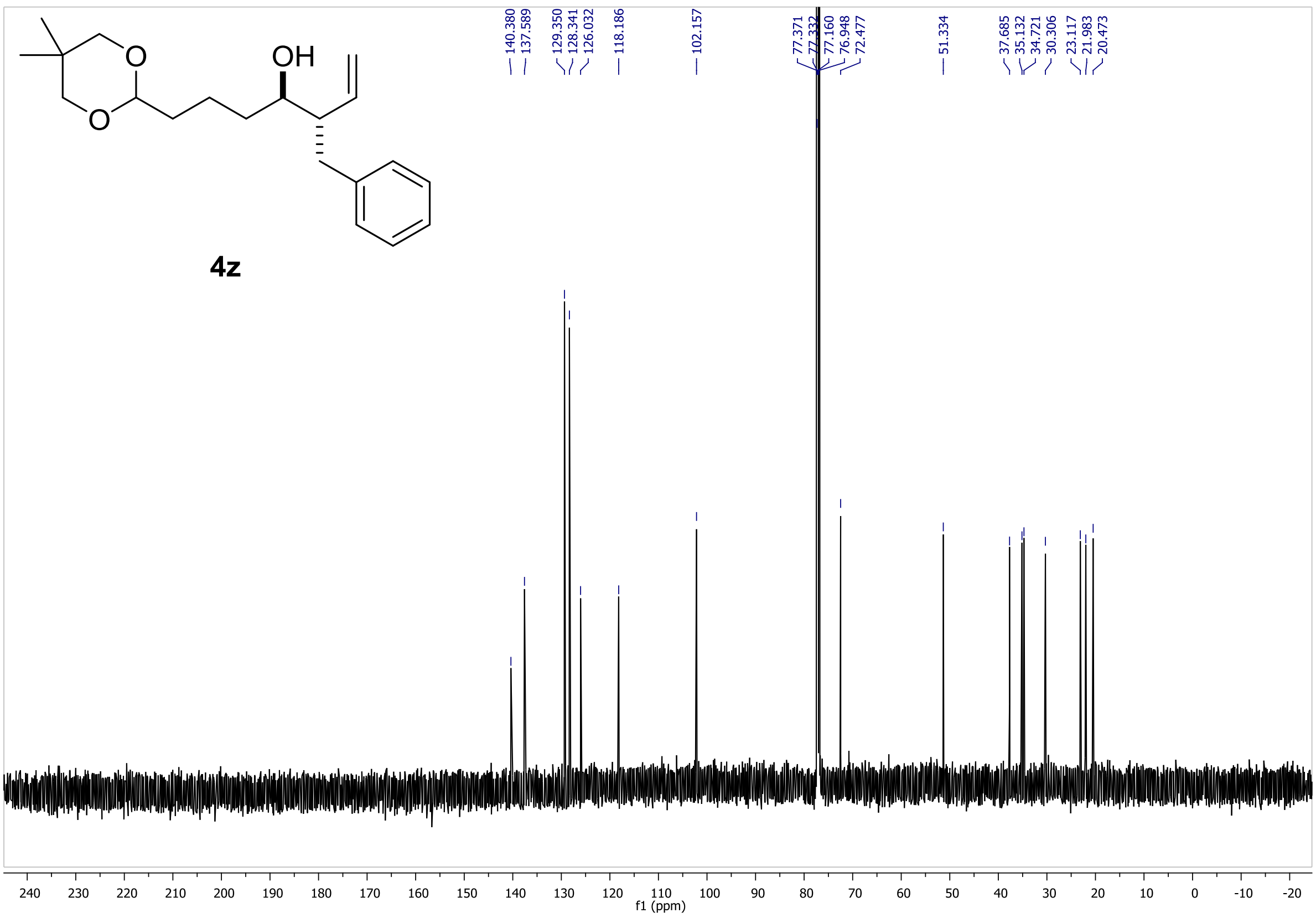

(*E*)-1-phenyl-4-(*p*-tolyl)hexa-1,5-dien-3-ol (4aa):

^{1}H NMR, 600 MHz, $CDCl_3$:

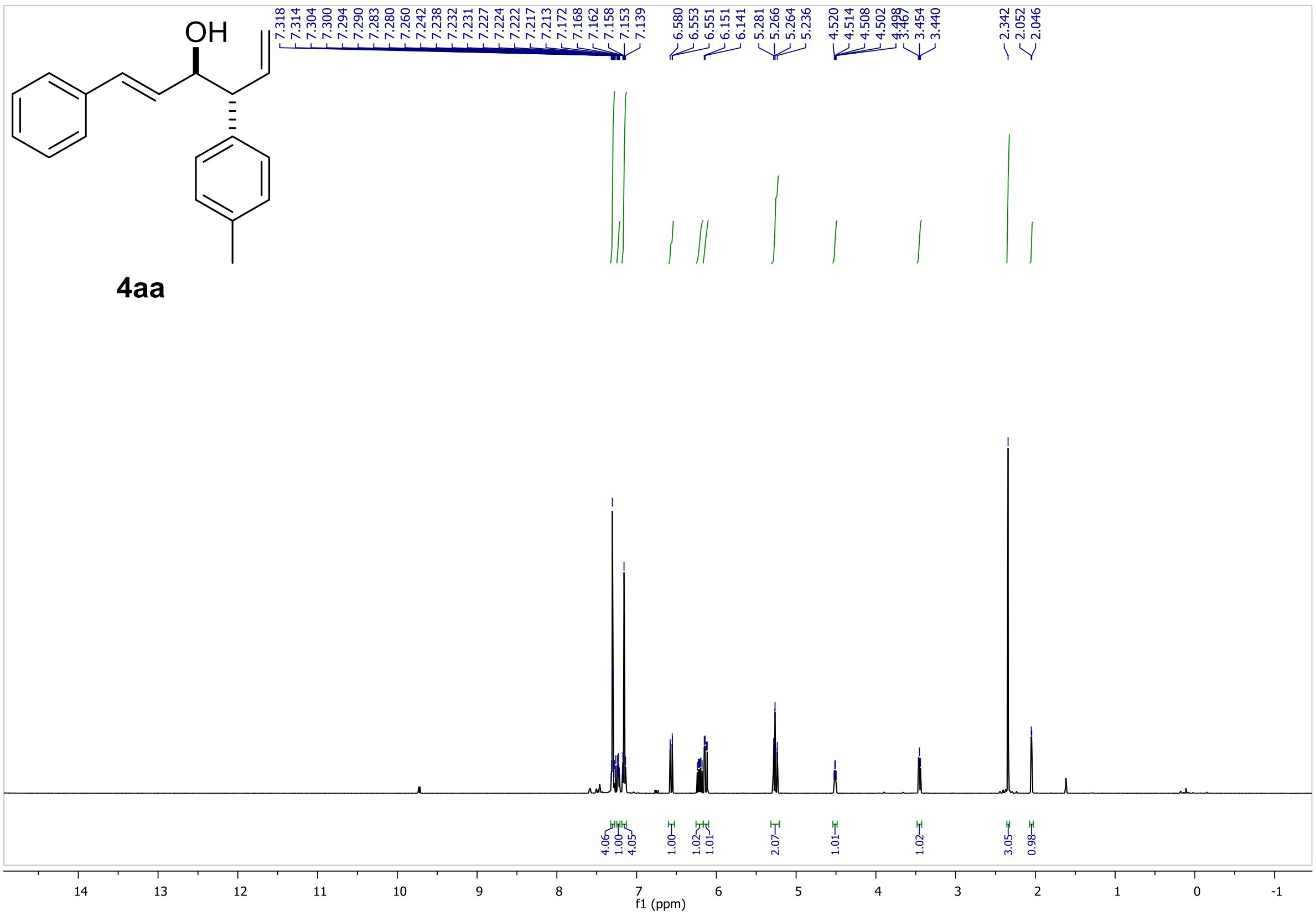

^{13}C NMR, 150 MHz, $CDCl_3$:

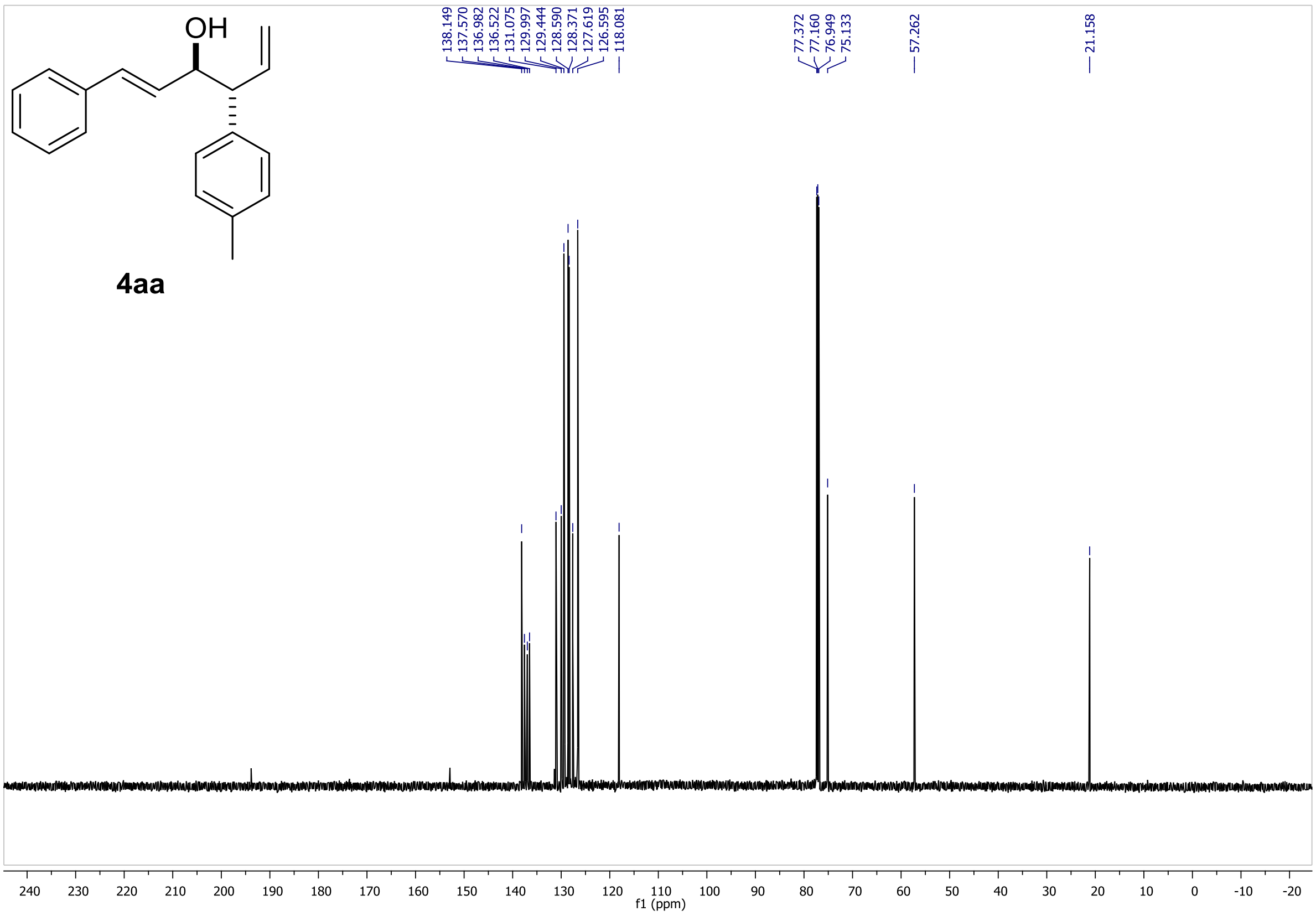

Methyl 4-(2-(chloromethyl)-1-hydroxybut-3-en-1-yl)benzoate (4ab):

^{1}H NMR, 600 MHz, $CDCl_3$:

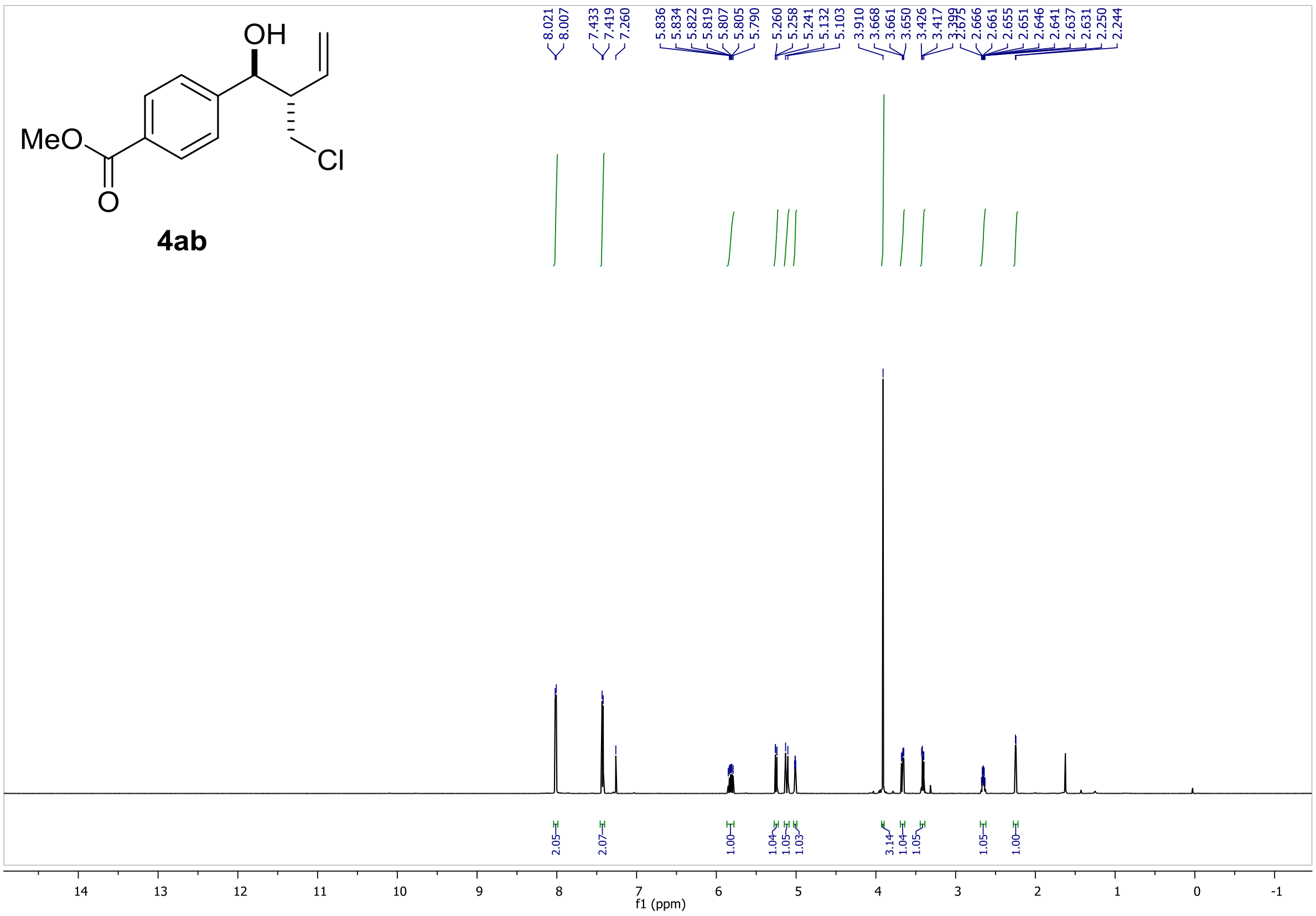

^{13}C NMR, 150 MHz, $CDCl_3$:

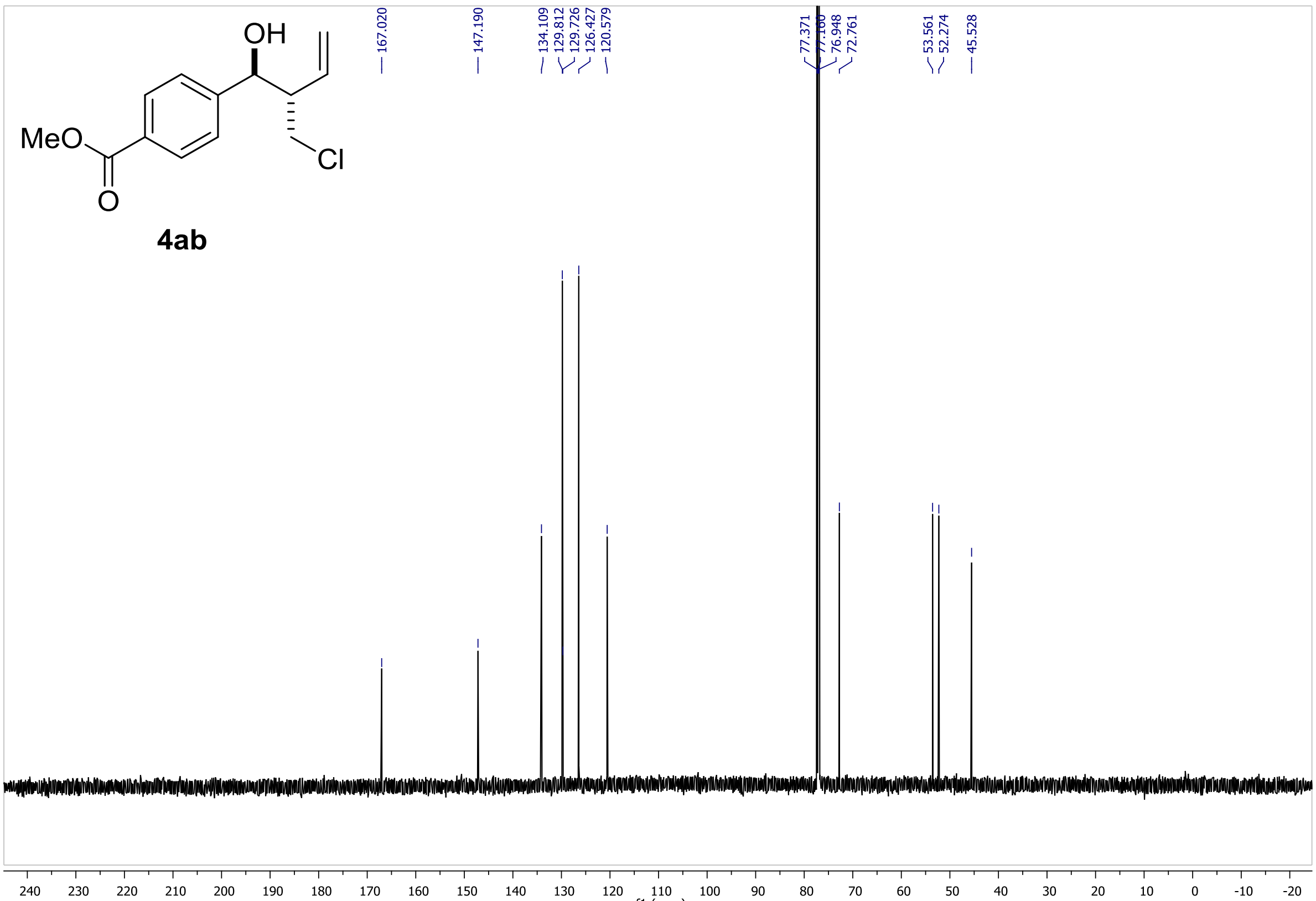

2-(chloromethyl)-1-(3-nitrophenyl)but-3-en-1-ol (4ac):

^{1}H NMR, 600 MHz, $CDCl_3$:

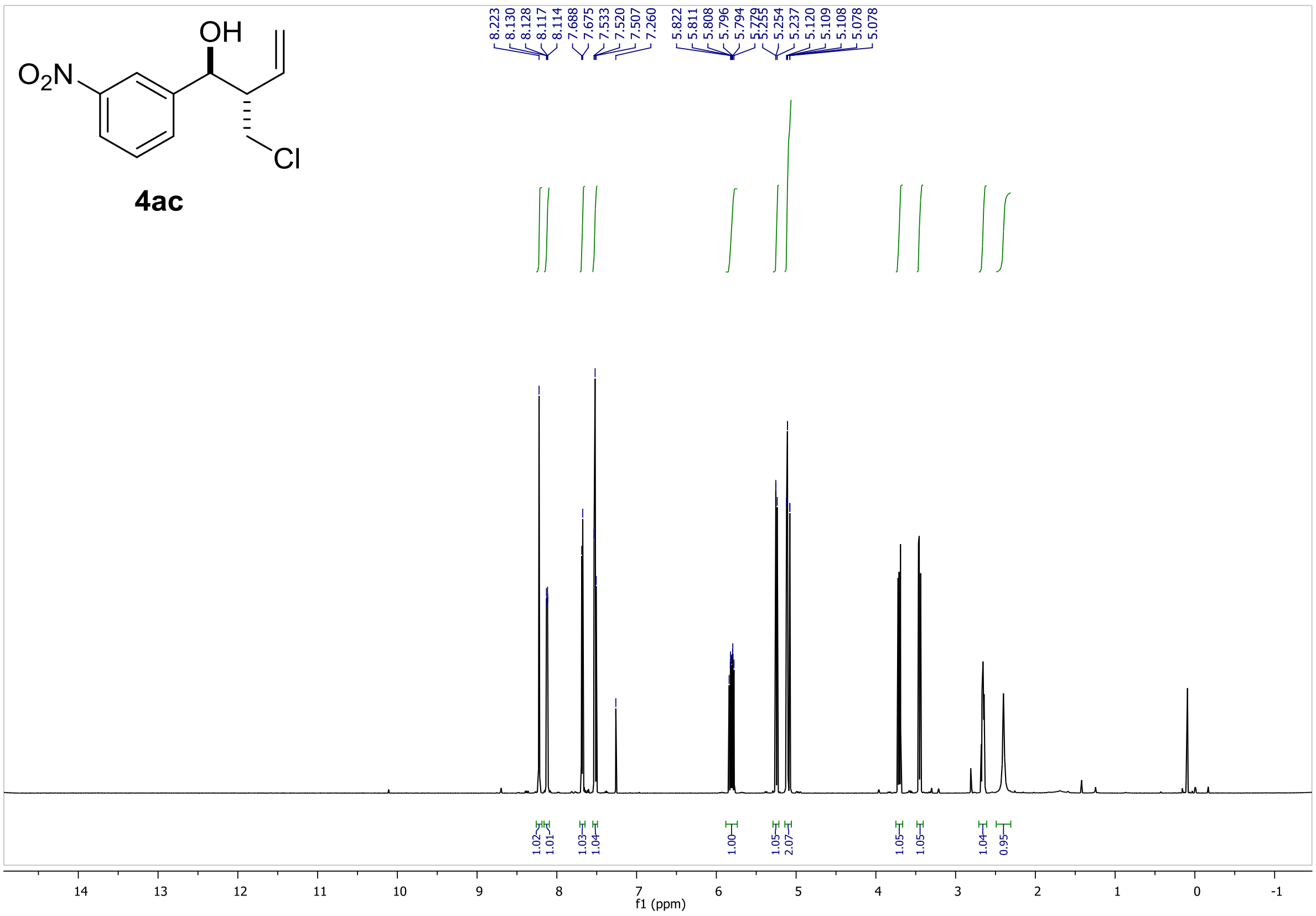

^{13}C NMR, 150 MHz, $CDCl_3$:

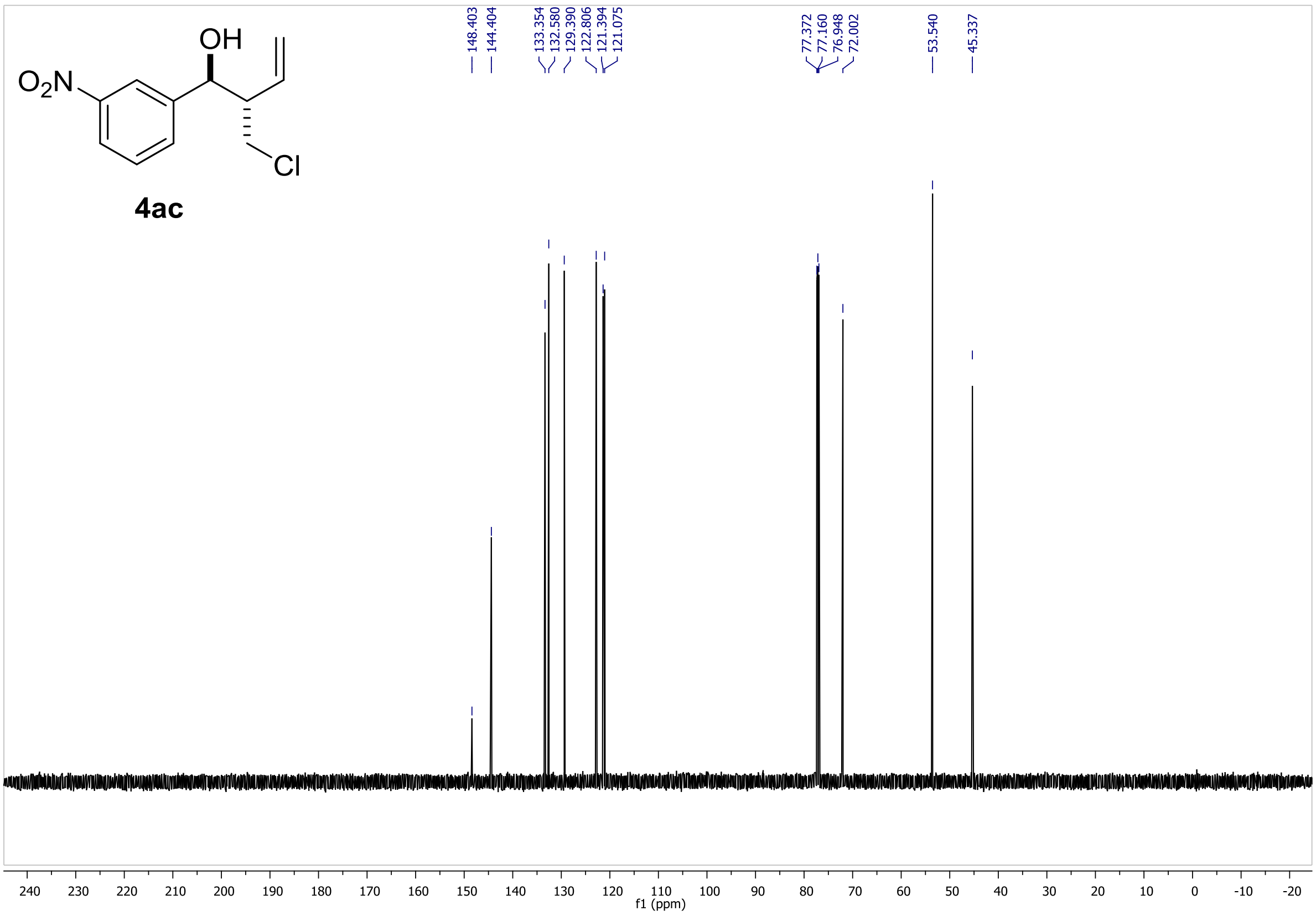

2-benzyl-1-(2-chloro-6-methoxyquinolin-3-yl)but-3-en-1-ol (4ad):

^{1}H NMR, 600 MHz, $CDCl_3$:

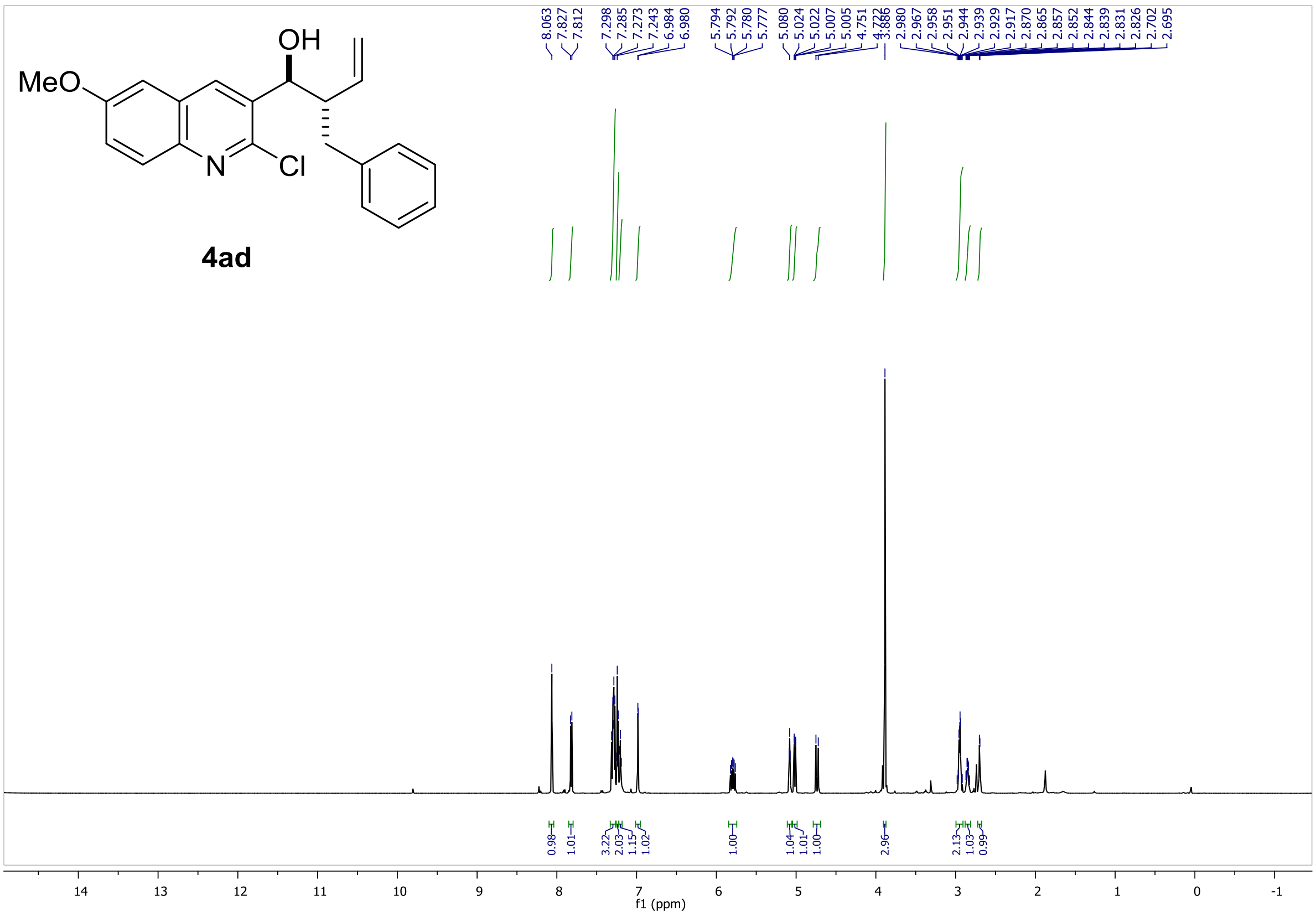

^{13}C NMR, 150 MHz, $CDCl_3$:

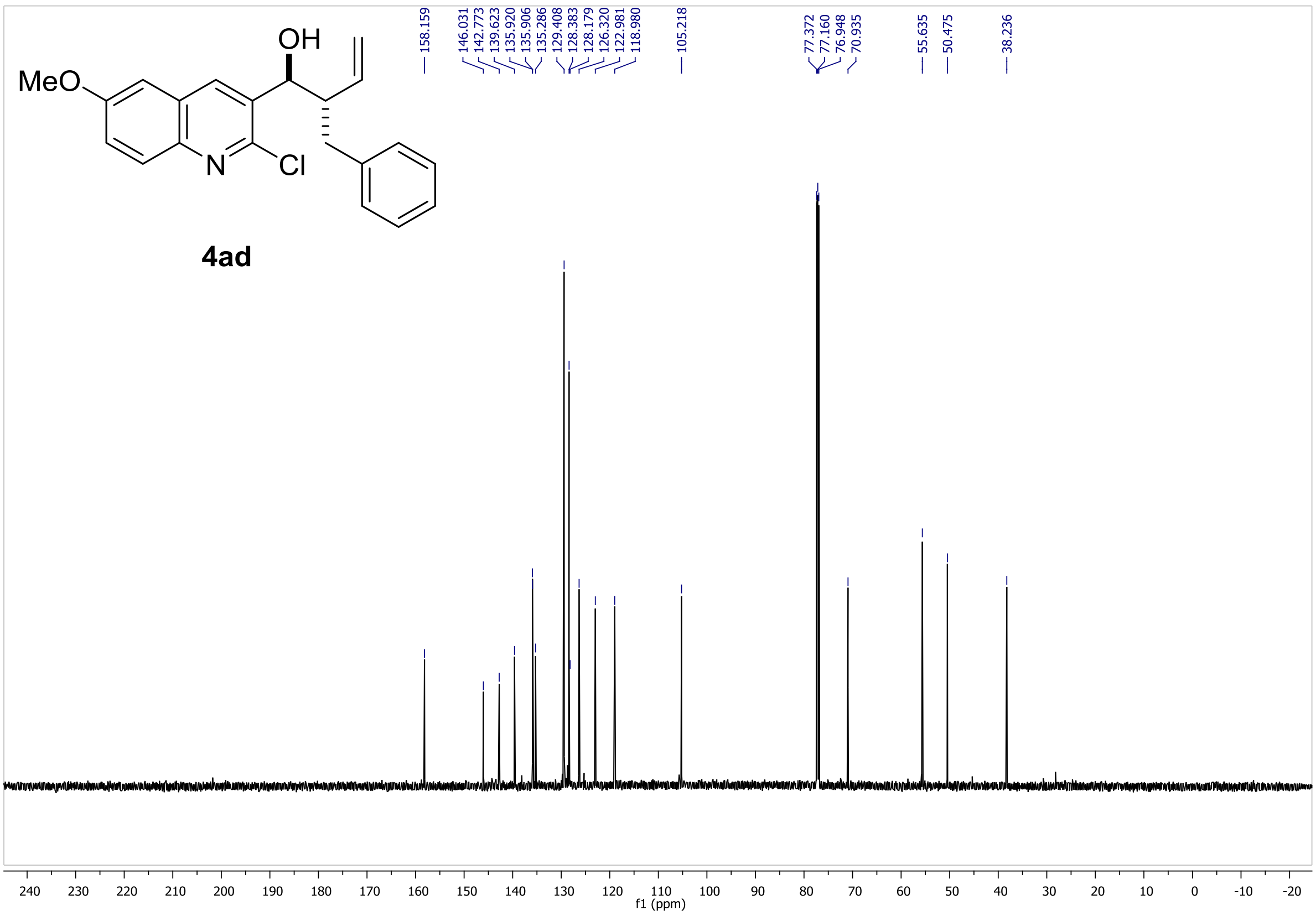

1-(2-chloro-6-methoxyquinolin-3-yl)-2-vinylhexan-1-ol (4ae):

^{1}H NMR, 600 MHz, $CDCl_3$:

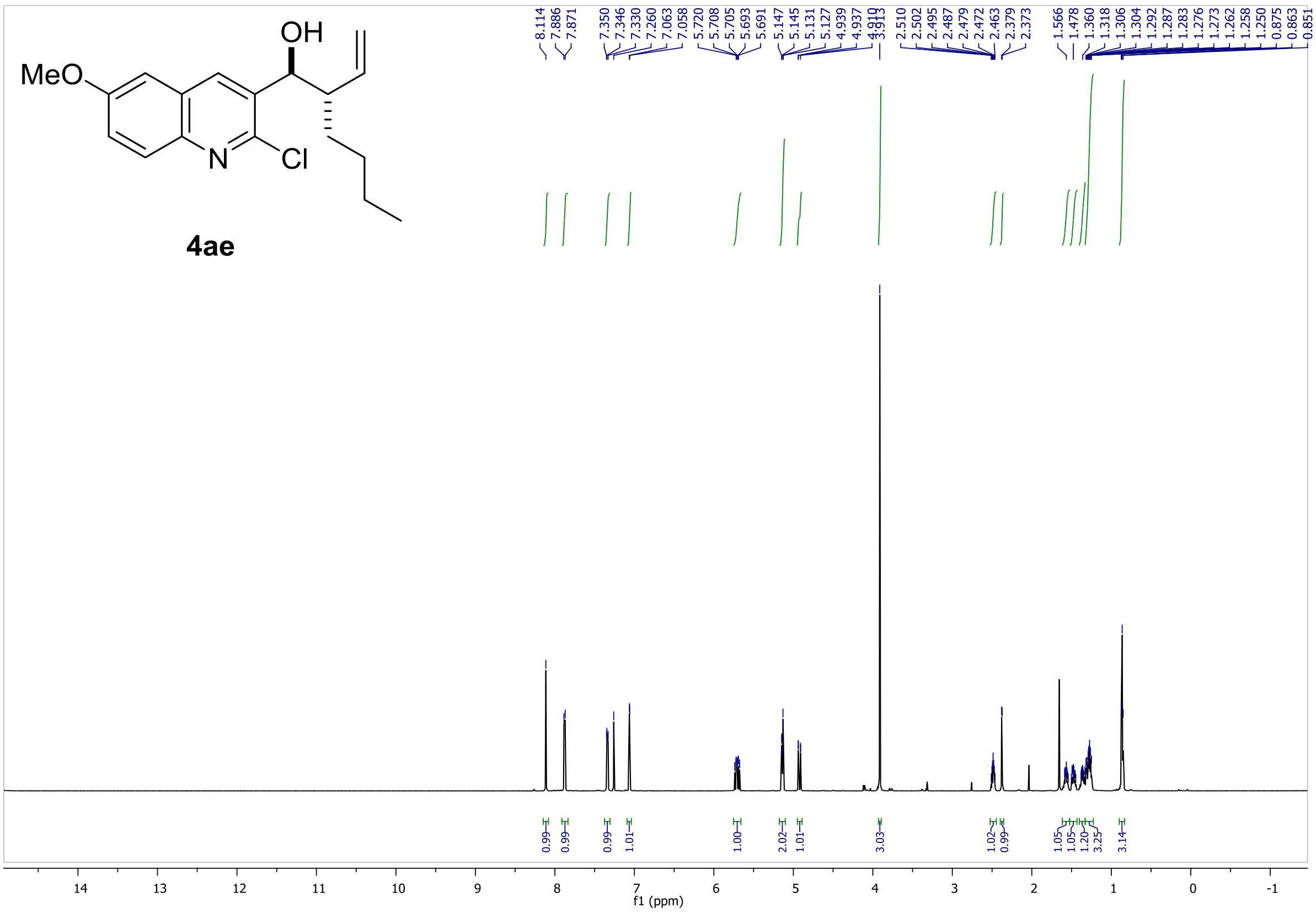

^{13}C NMR, 150 MHz, $CDCl_3$:

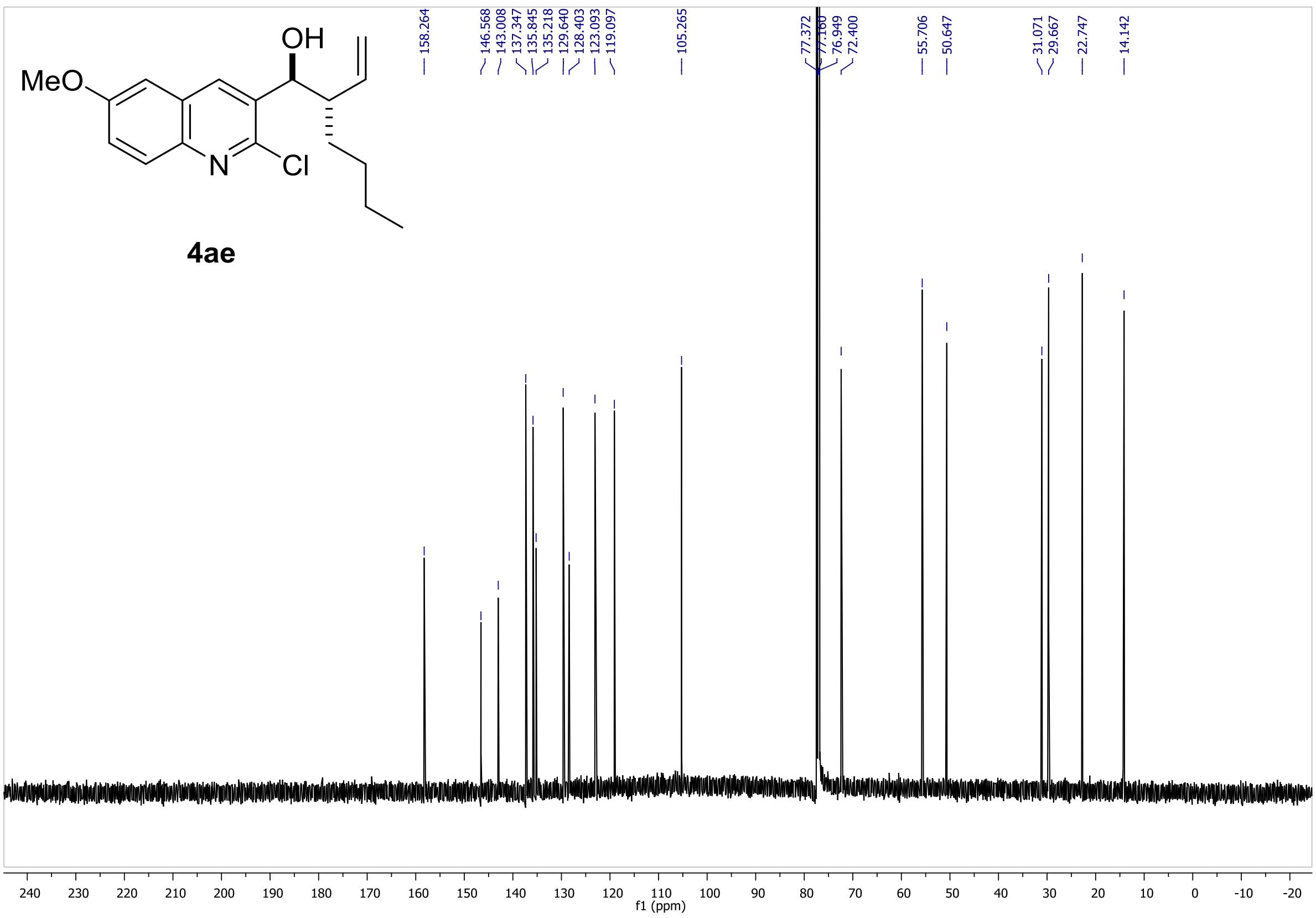

1-(4-bromophenyl)-2-(chloromethyl)but-3-en-1-yl)oxy)triethylsilane (5):

^{1}H NMR, 600 MHz, $CDCl_3$:

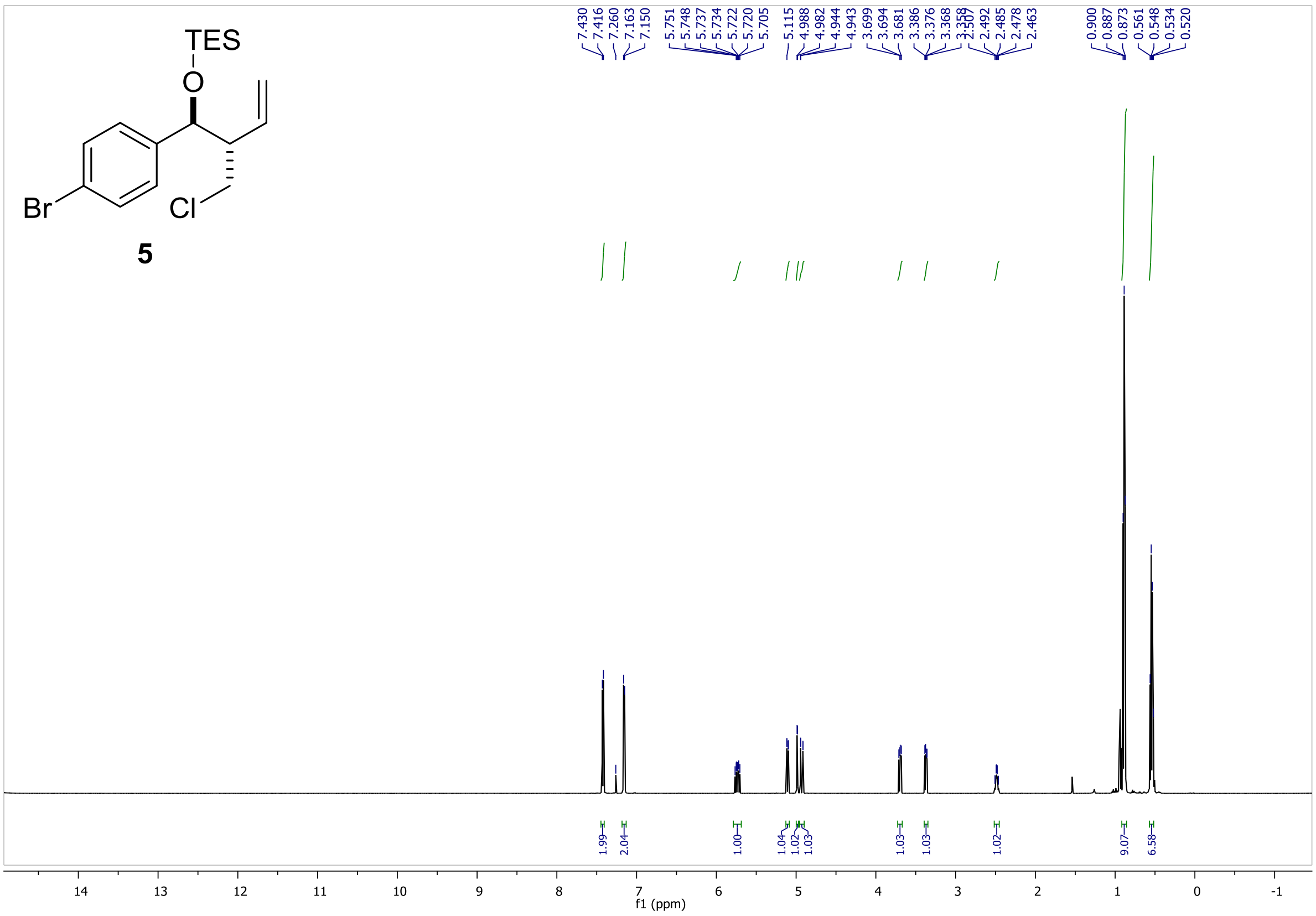

^{13}C NMR, 150 MHz, $CDCl_3$:

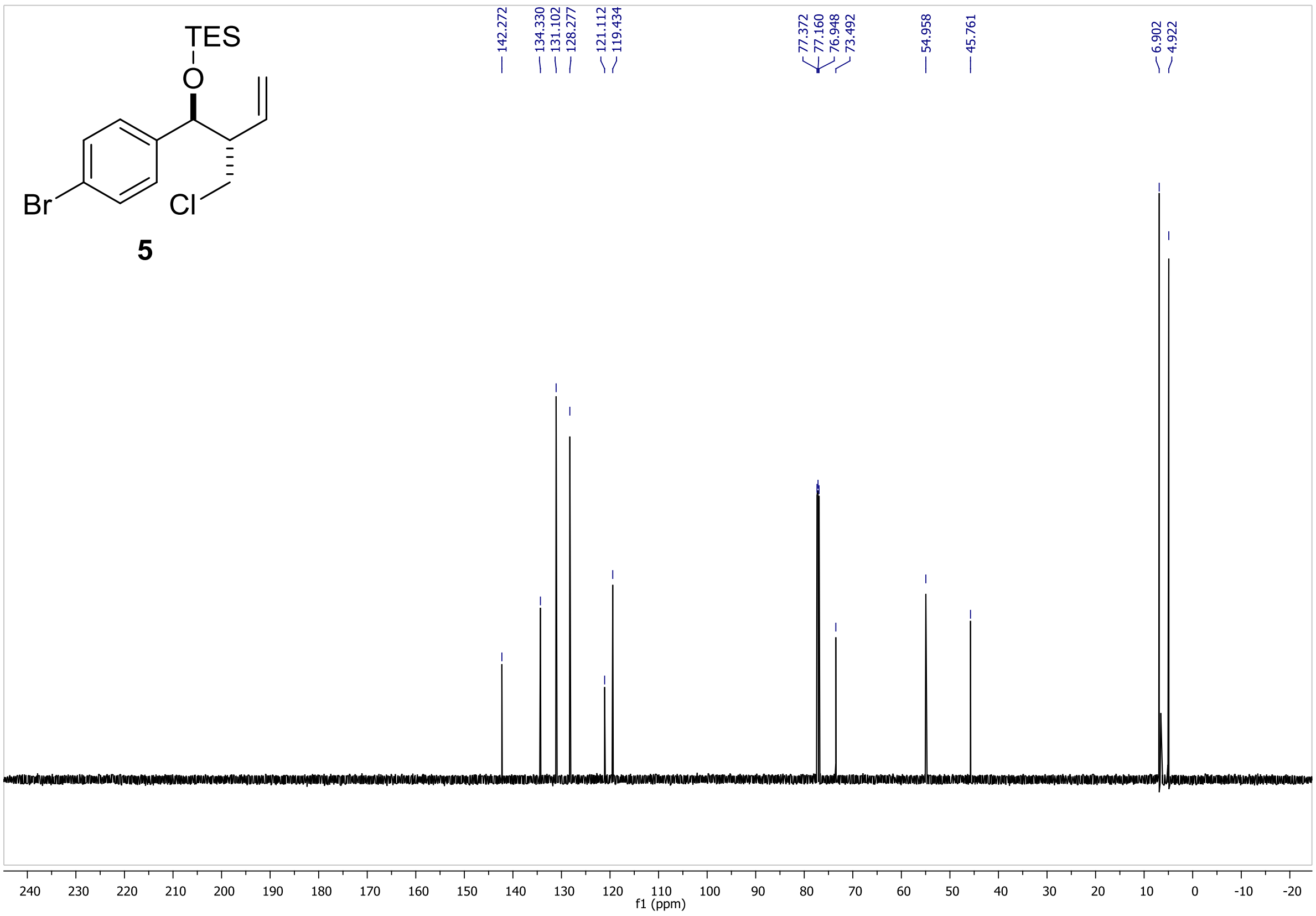

1-(4-bromophenyl)-2-(chloromethyl)-4-(4-fluorophenyl)-1-((triethylsilyl)oxy)hex-5-en-3-ol (5a):

^{1}H NMR, 600 MHz, CDCl$_3$:

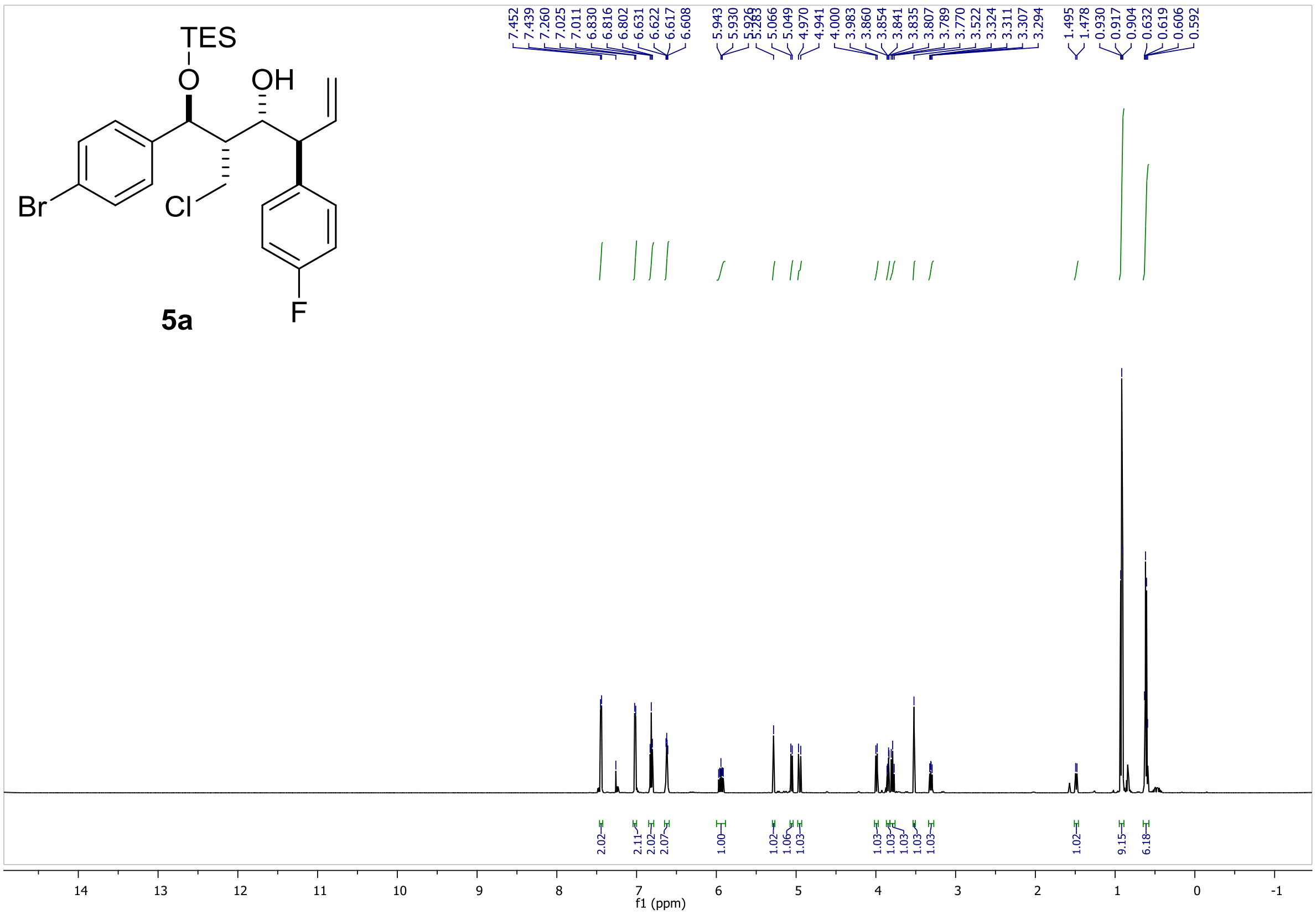

^{13}C NMR, 150 MHz, CDCl$_3$:

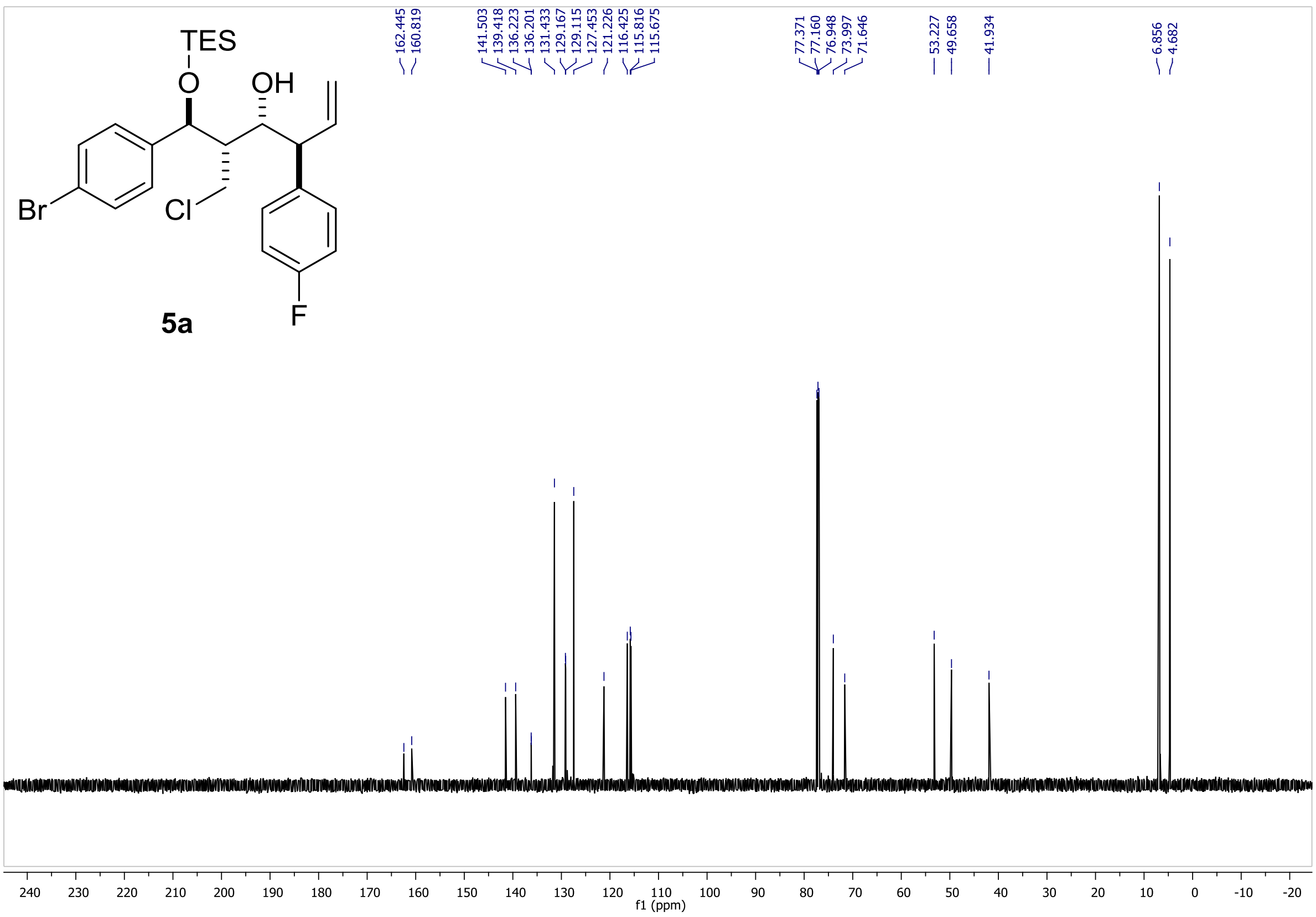

^{19}F NMR, 376 MHz, $CDCl_3$:

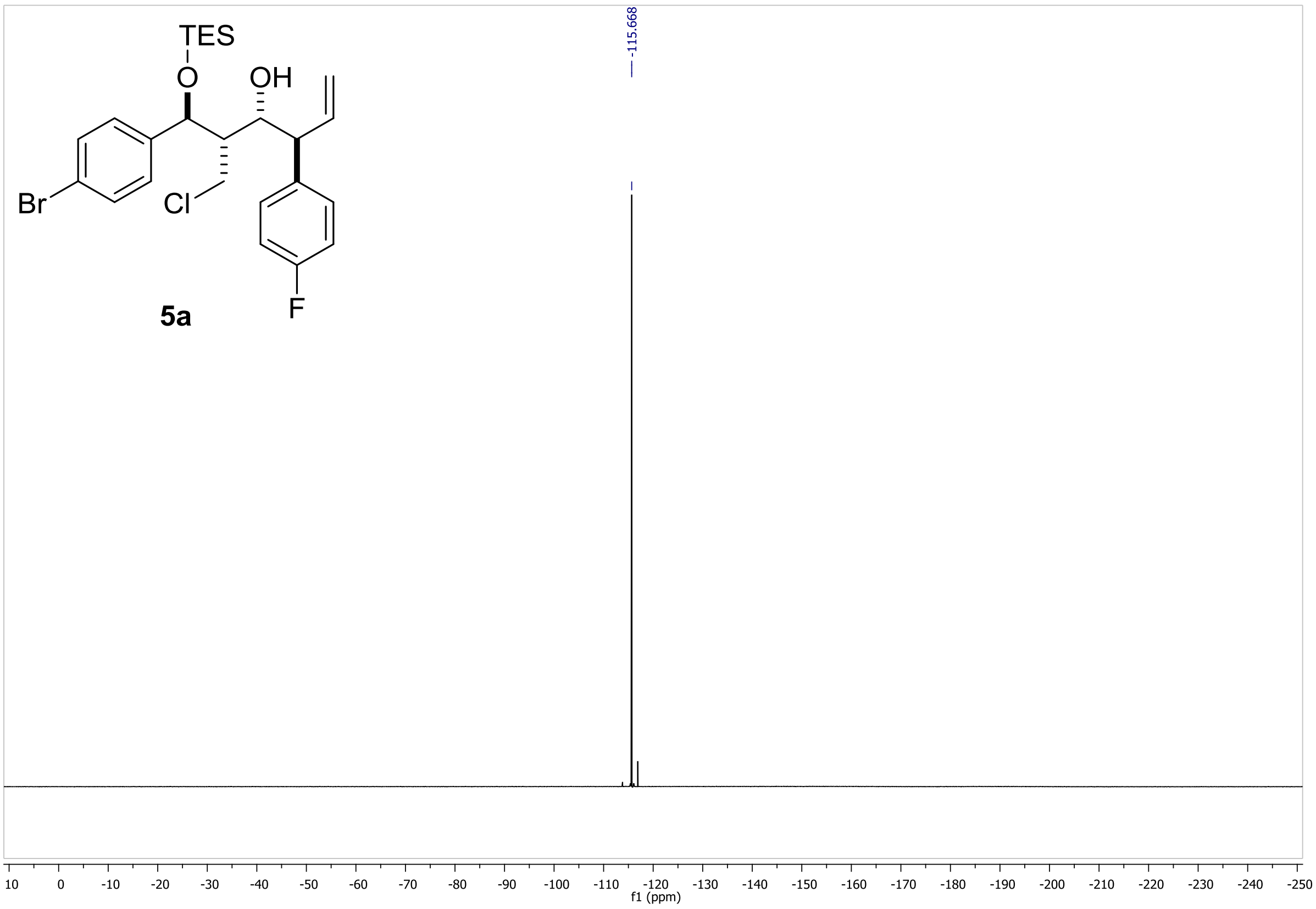

4-(4-bromophenyl)-5-(chloromethyl)-6-(1-(4-fluorophenyl)allyl)-2,2-dimethyl-1,3-dioxane (6):

^{1}H NMR, 600 MHz, $CDCl_3$:

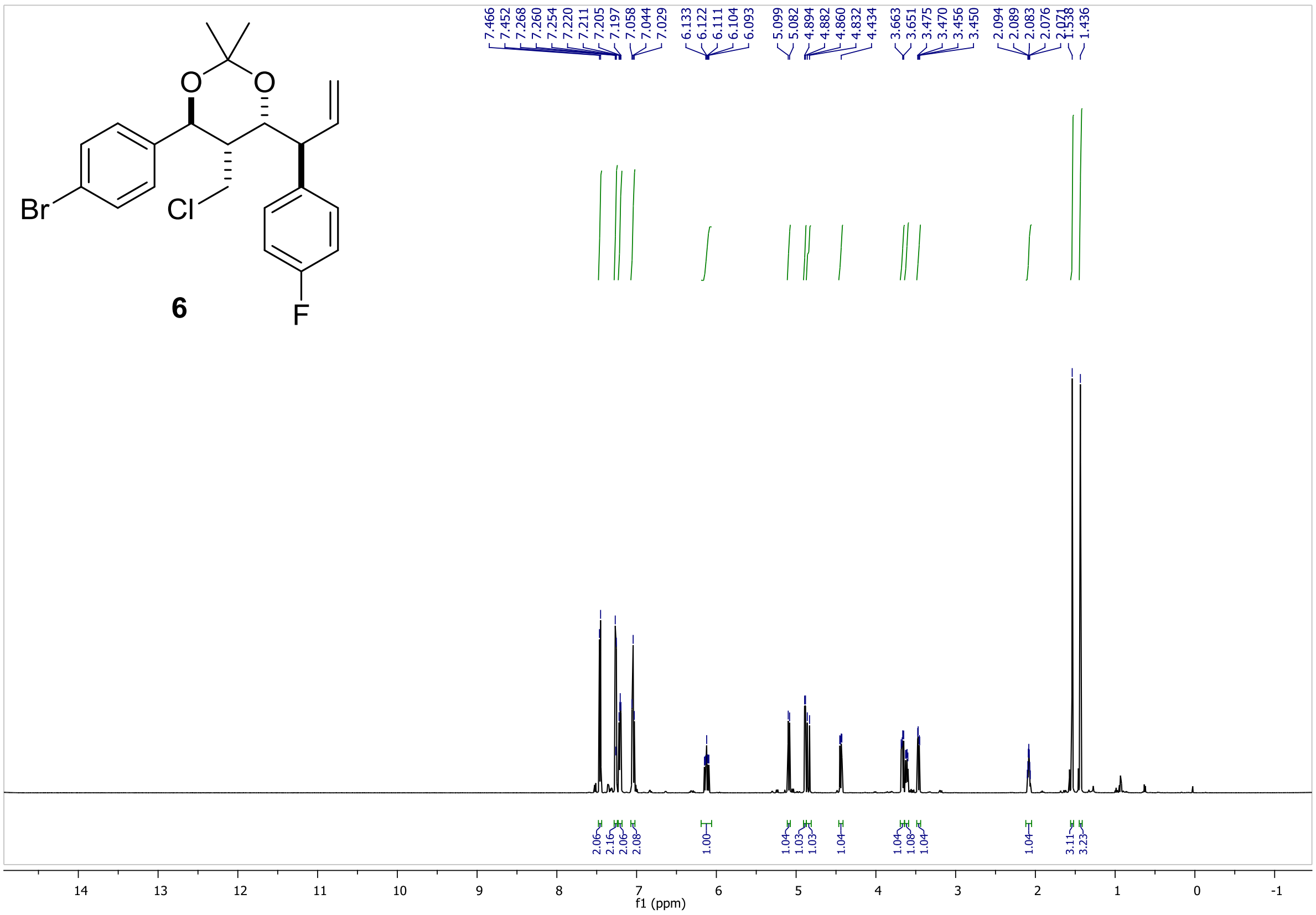

^{13}C NMR, 150 MHz, $CDCl_3$:

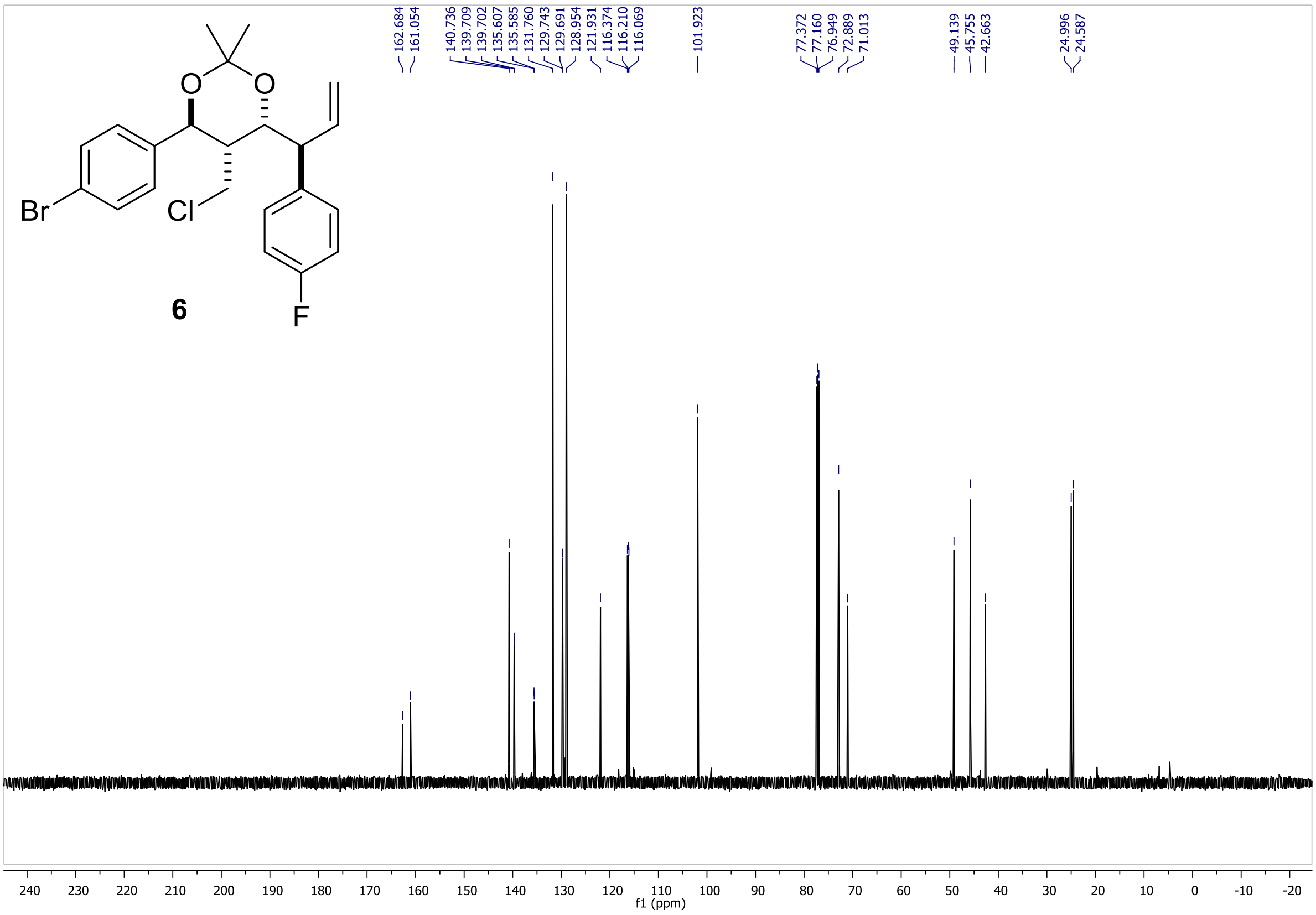

^{19}F NMR, 376 MHz, $CDCl_3$:

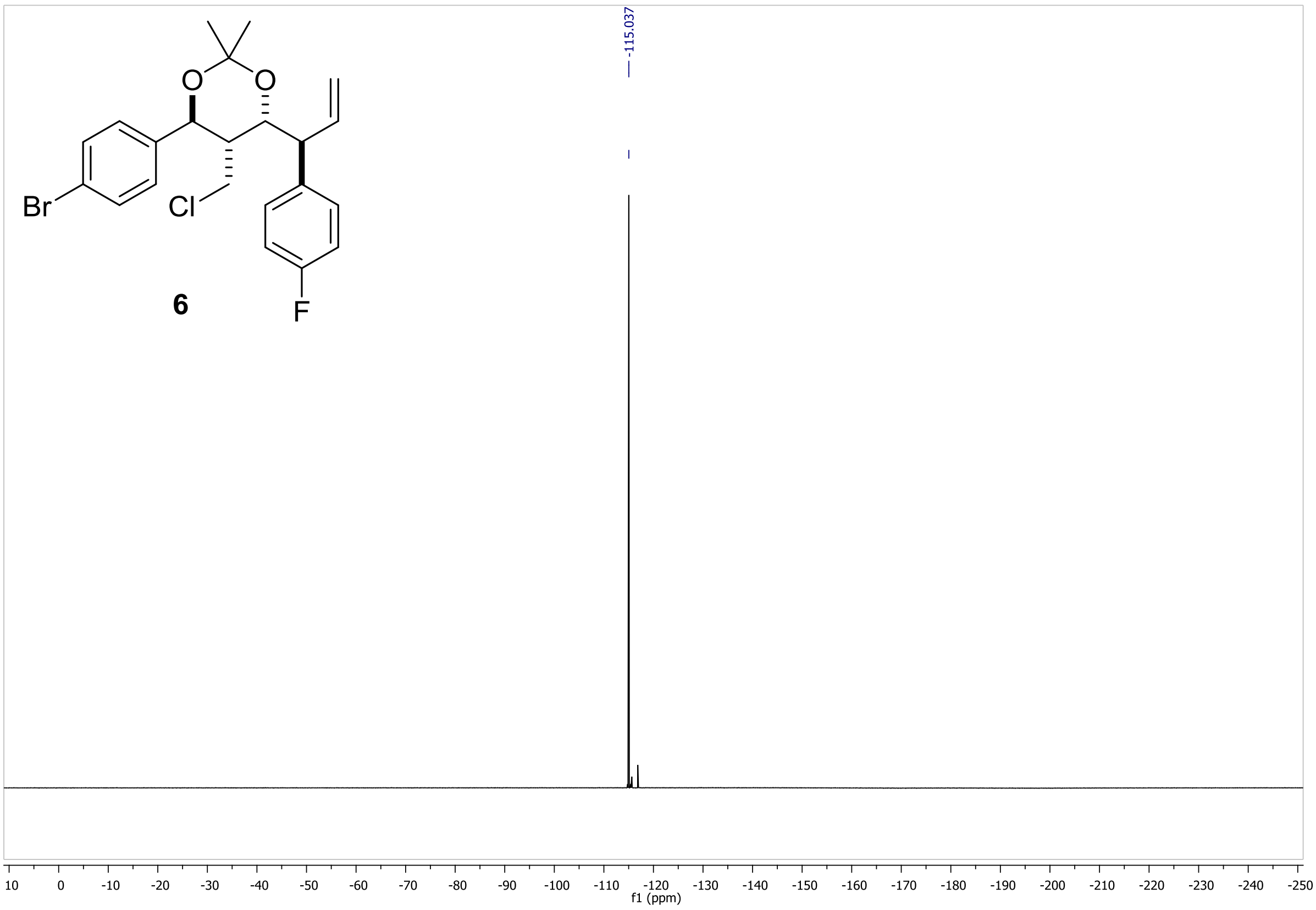

1-(6-(4-bromophenyl)-5-(chloromethyl)-2,2-dimethyl-1,3-dioxan-4-yl)-1-(4-fluoro-phenyl)-3-(*p*-tolyl)pent-4-en-2-ol (6a, *major diastereomer*):

^{1}H NMR, 600 MHz, CDCl$_3$:

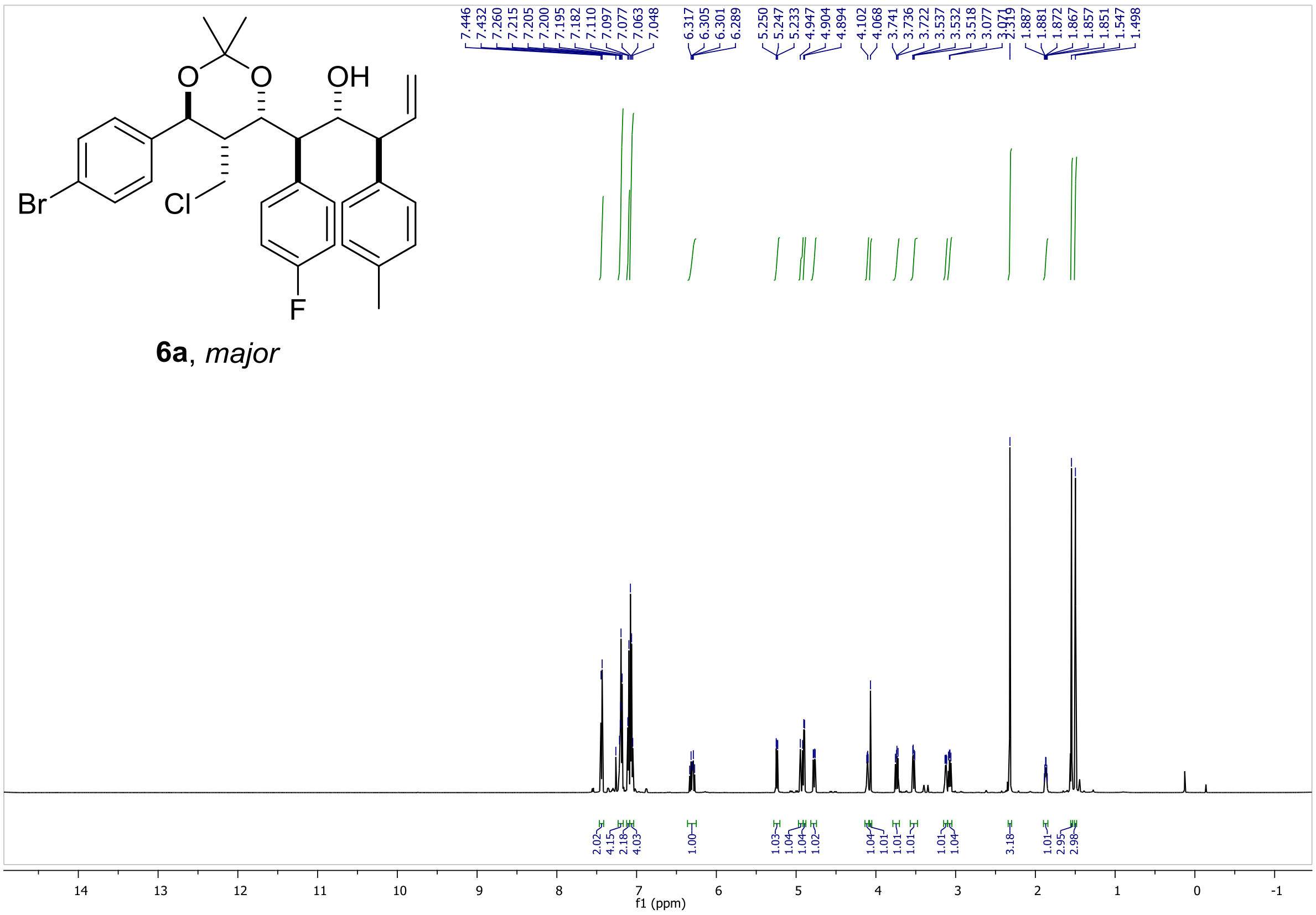

^{13}C NMR, 150 MHz, CDCl$_3$:

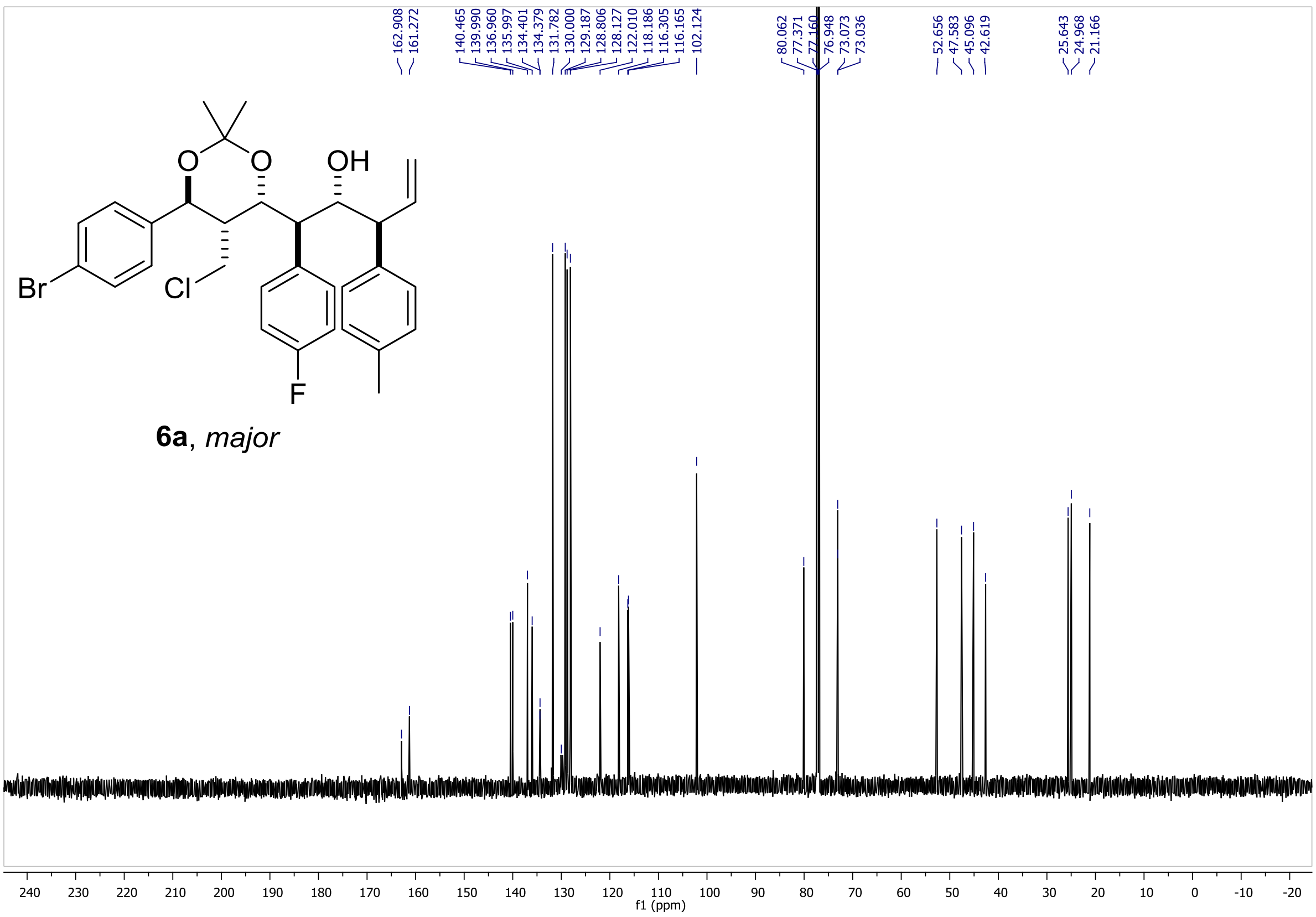

^{19}F NMR, 376 MHz, CDCl$_3$:

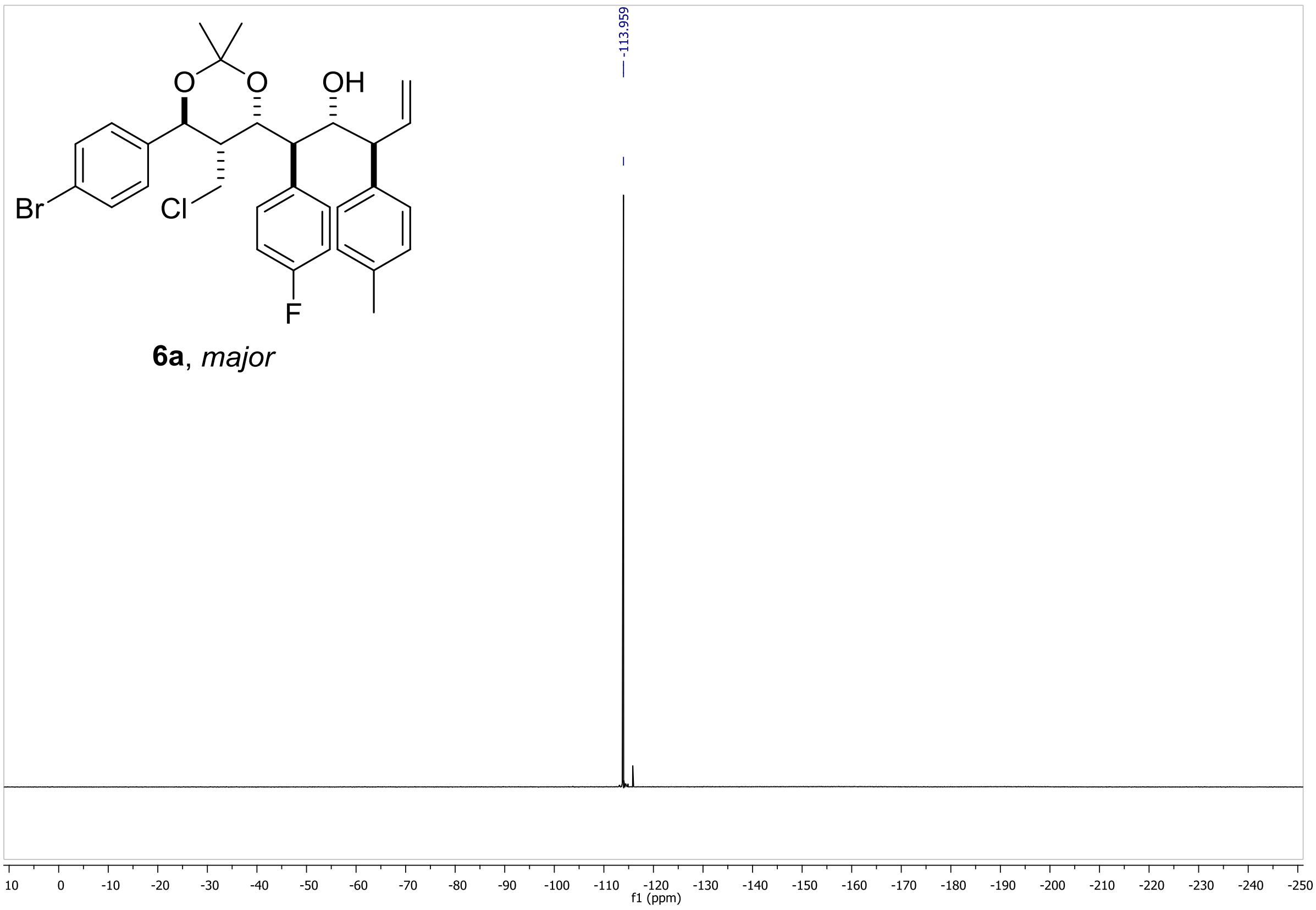

1-(6-(4-bromophenyl)-5-(chloromethyl)-2,2-dimethyl-1,3-dioxan-4-yl)-1-(4-fluoro-phenyl)-3-(*p*-tolyl)pent-4-en-2-ol (6a, *minor diastereomer*):

^{1}H NMR, 600 MHz, $CDCl_3$:

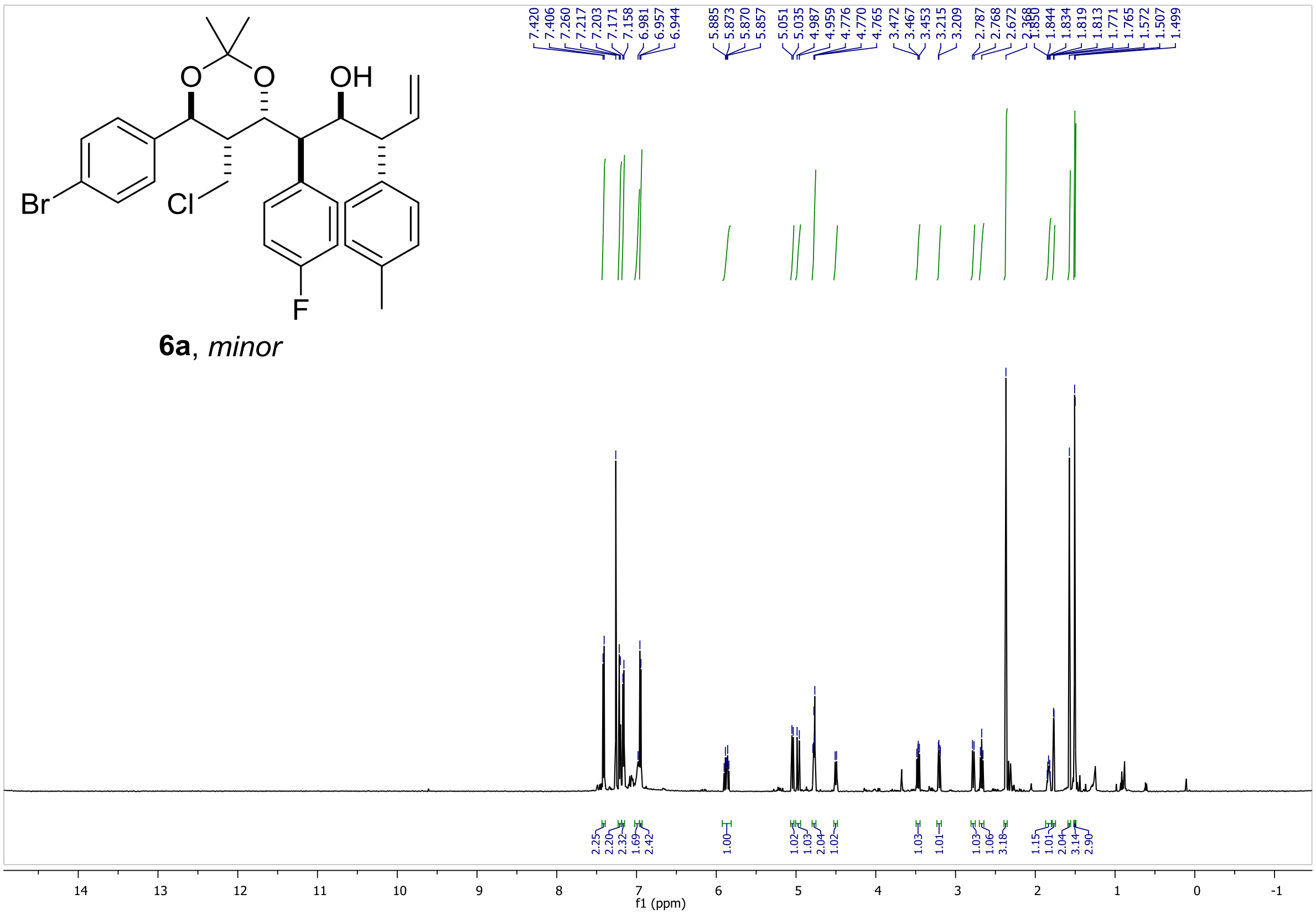

^{13}C NMR, 150 MHz, $CDCl_3$:

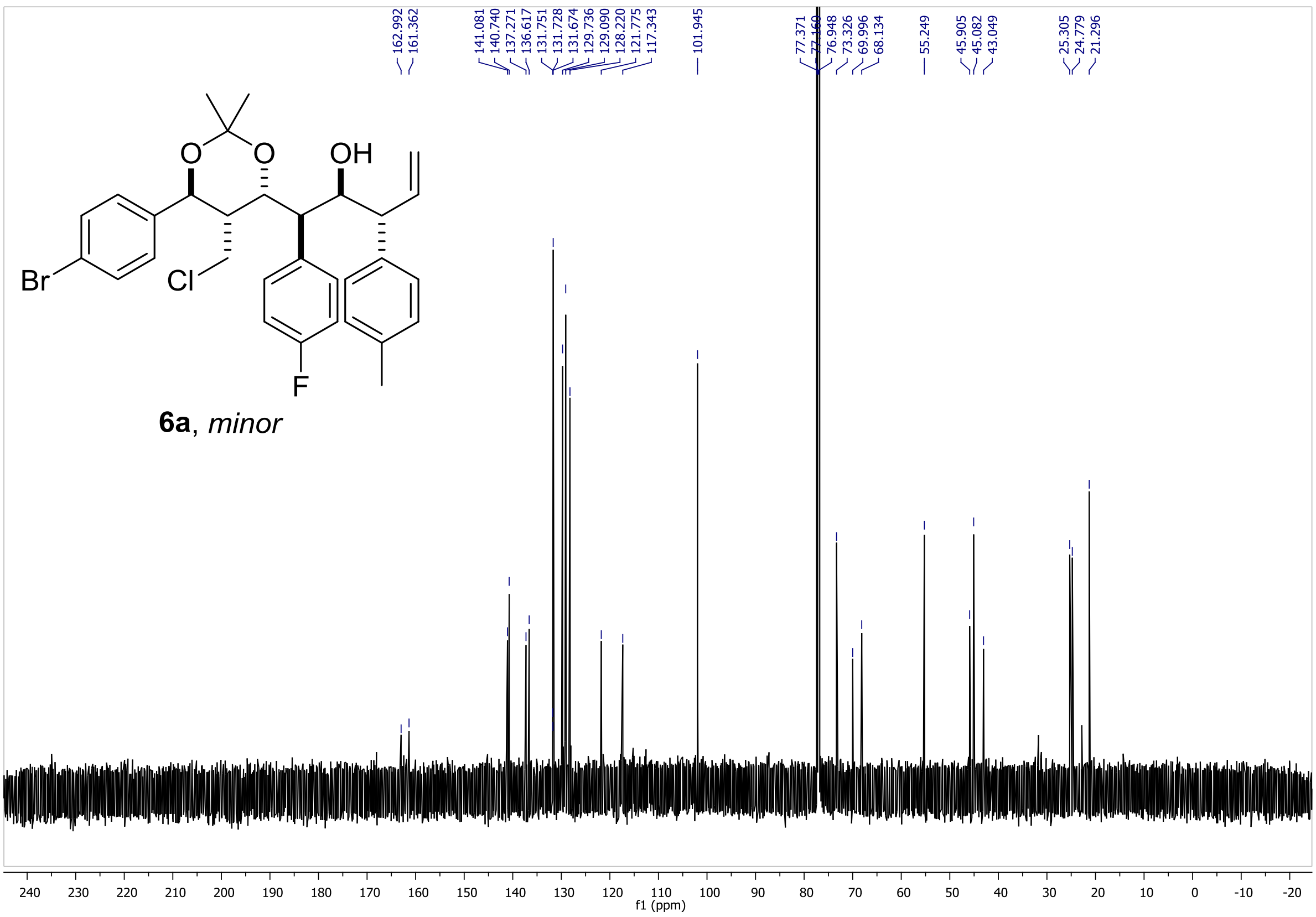

^{19}F NMR, 376 MHz, $CDCl_3$:

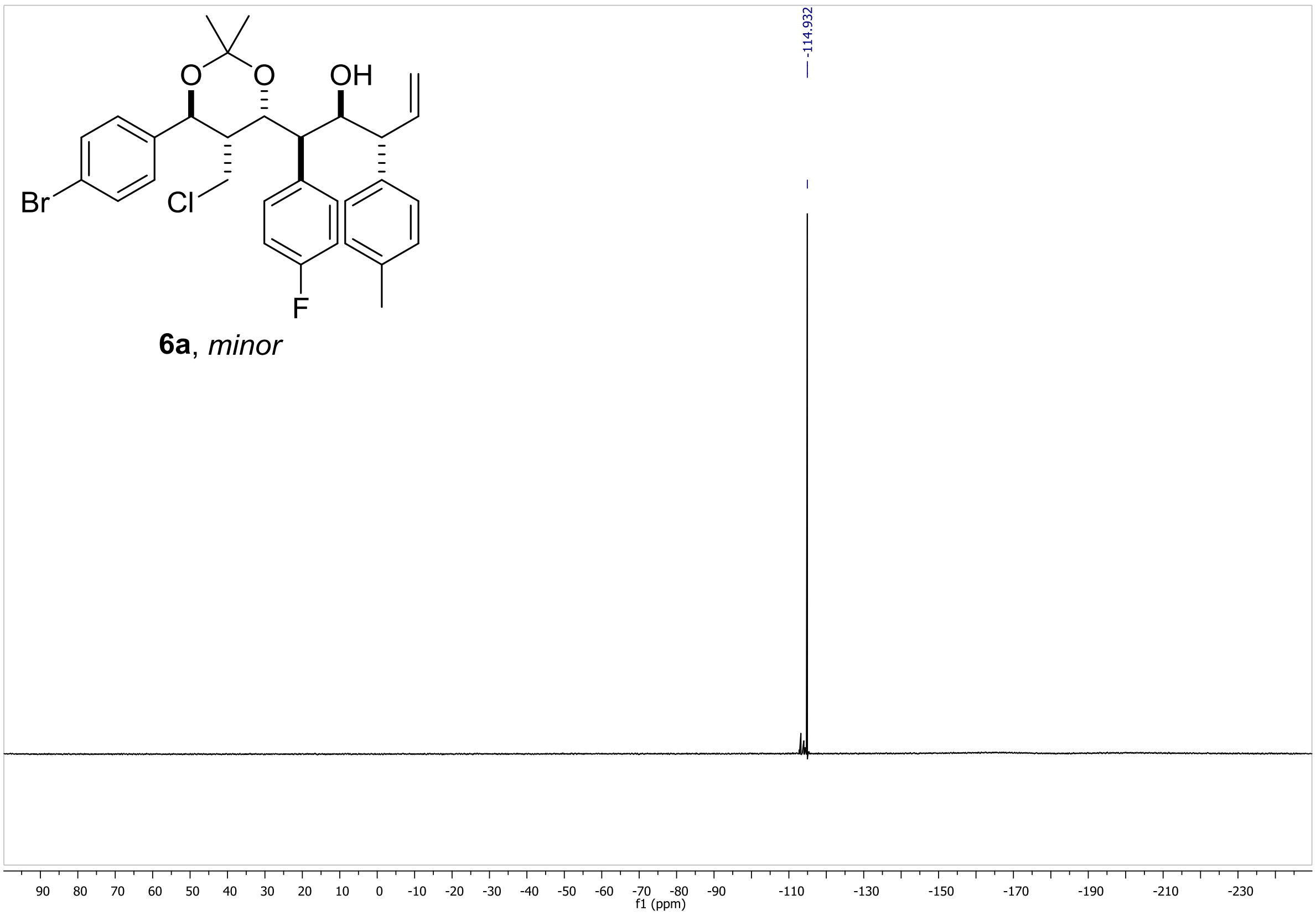

((1-(6-(4-bromophenyl)-5-(chloromethyl)-2,2-dimethyl-1,3-dioxan-4-yl)-1-(4-fluorophenyl)-3-(*p*-tolyl)pent-4-en-2-yl)oxy)triethylsilane (7):

^{1}H NMR, 600 MHz, $CDCl_3$:

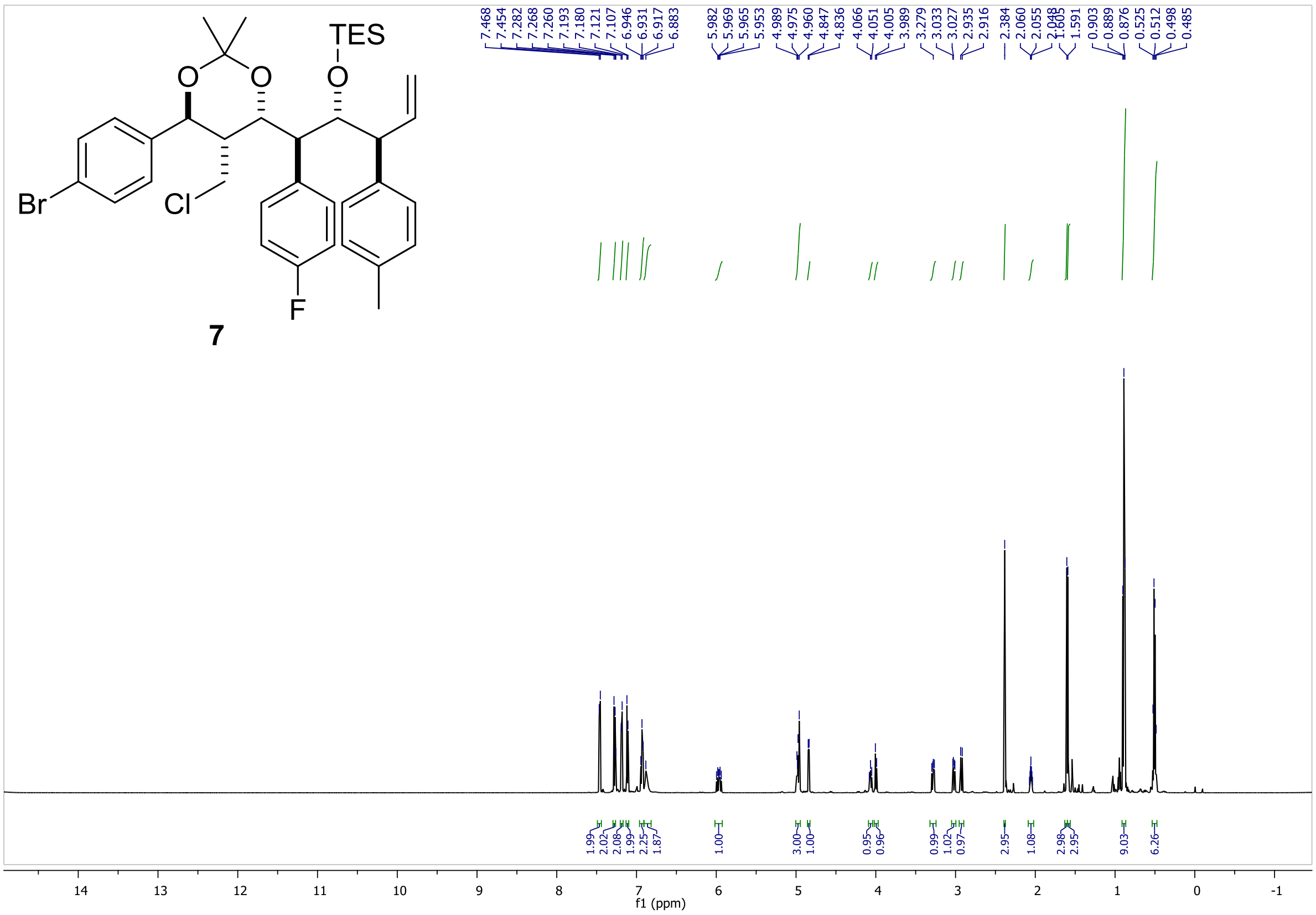

^{13}C NMR, 150 MHz, $CDCl_3$:

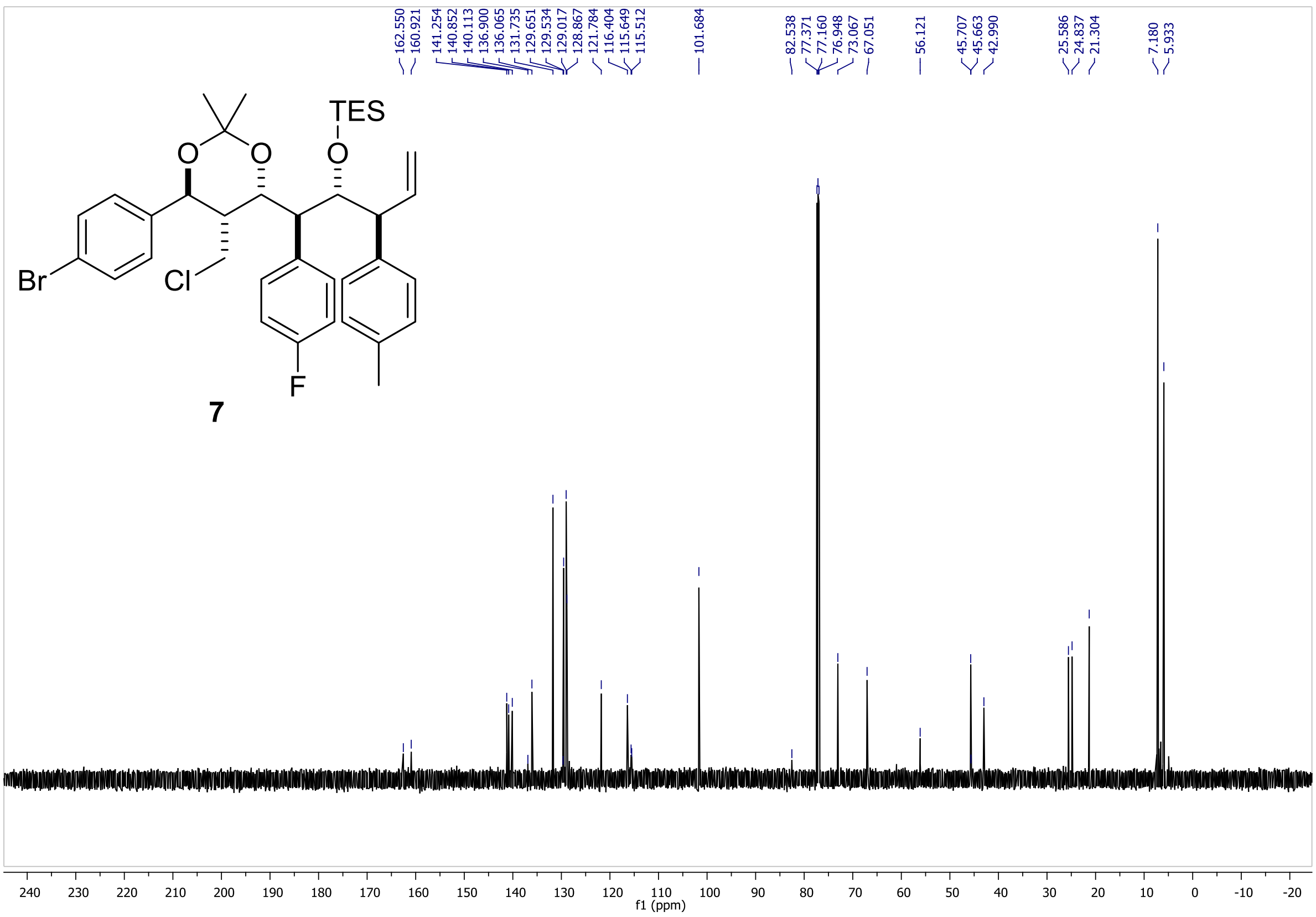

^{19}**F NMR, 376 MHz, $CDCl_3$:**

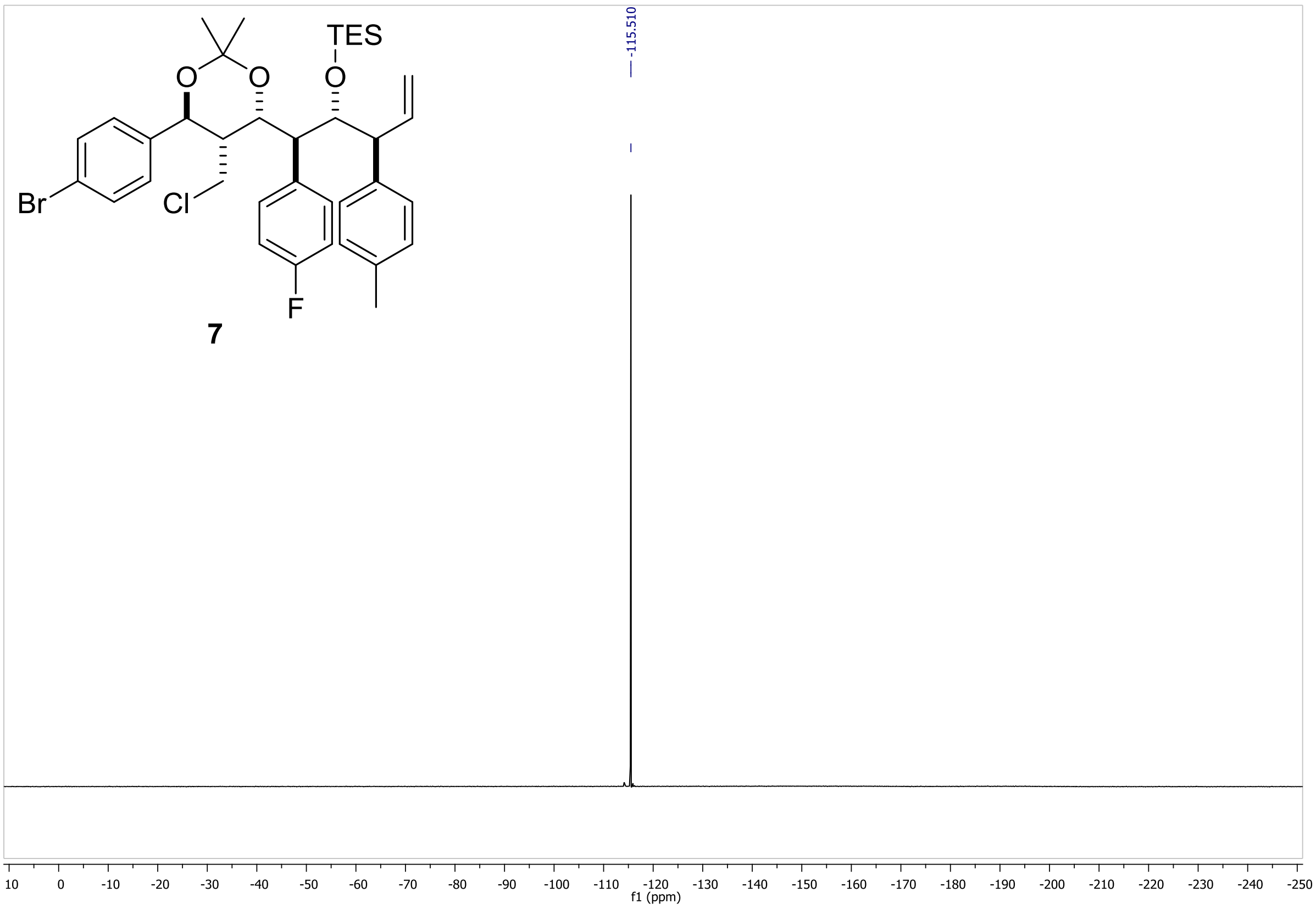

7-(6-(4-bromophenyl)-5-(chloromethyl)-2,2-dimethyl-1,3-dioxan-4-yl)-7-(4-fluorophenyl)-3-phenyl-5-(*p*-tolyl)-6-((triethylsilyl)oxy)hept-1-en-4-ol (8):

^{1}H NMR, 600 MHz, CDCl$_3$:

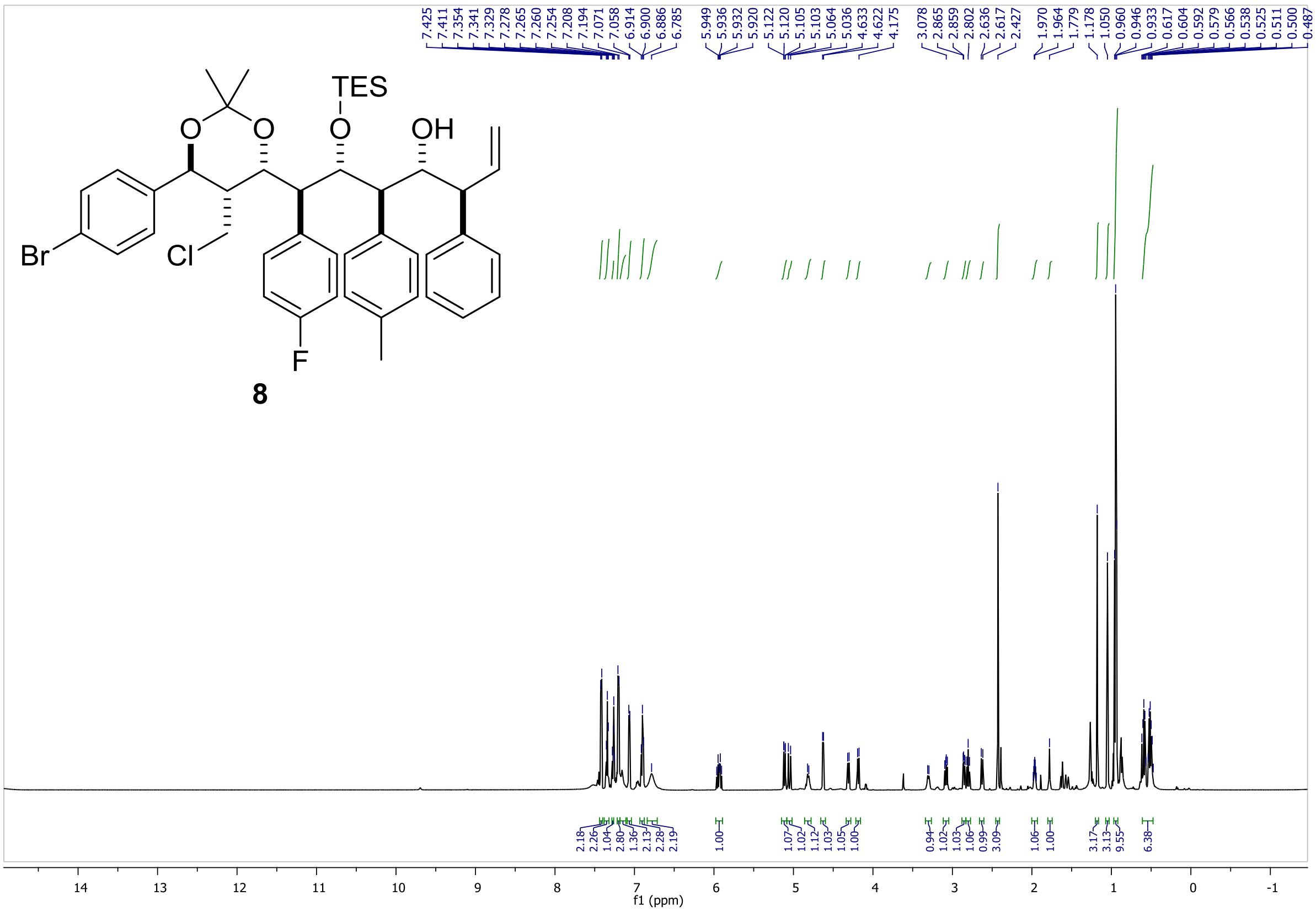

^{13}C NMR, 150 MHz, CDCl$_3$:

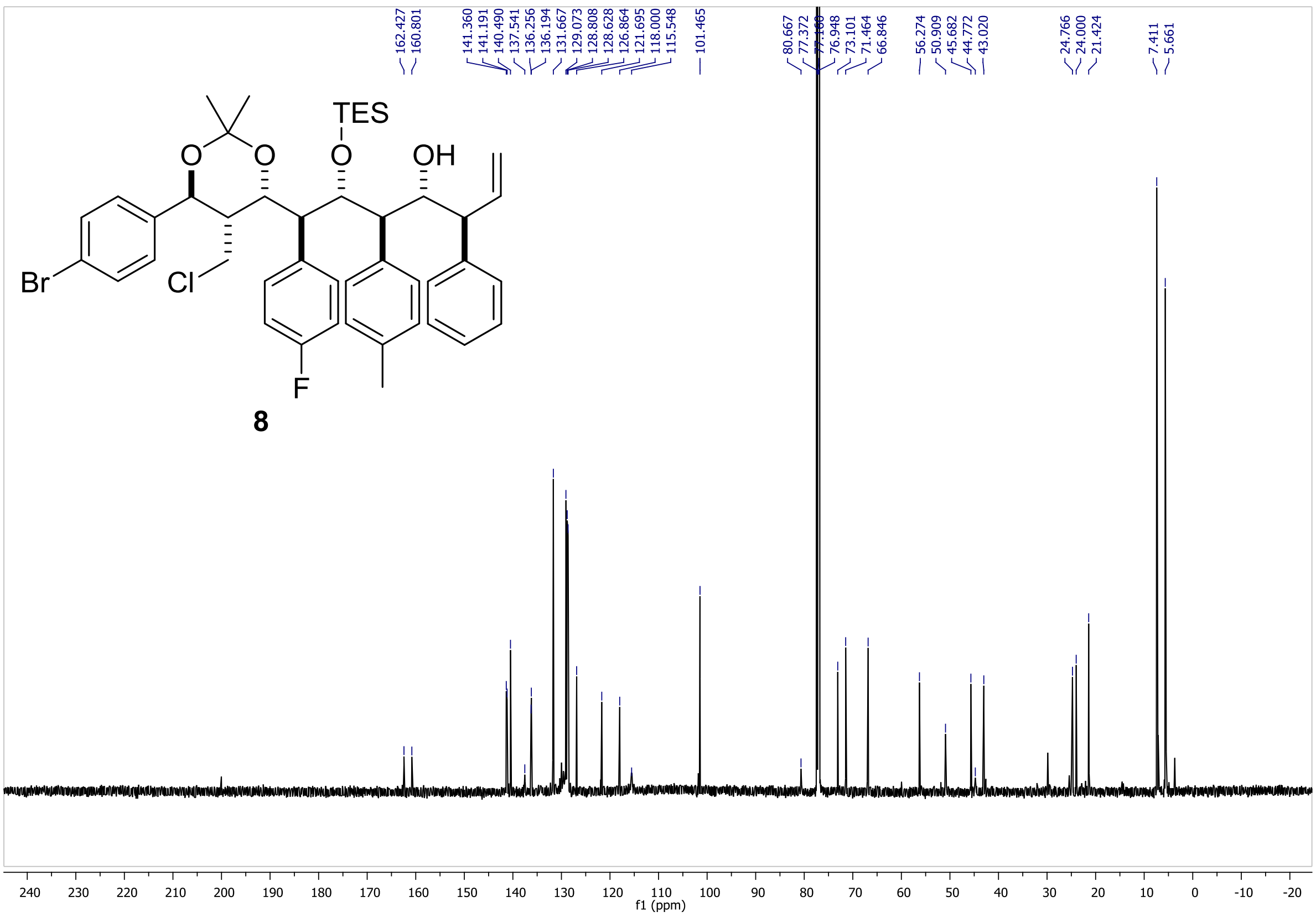

^{19}F NMR, 376 MHz, CDCl$_3$:

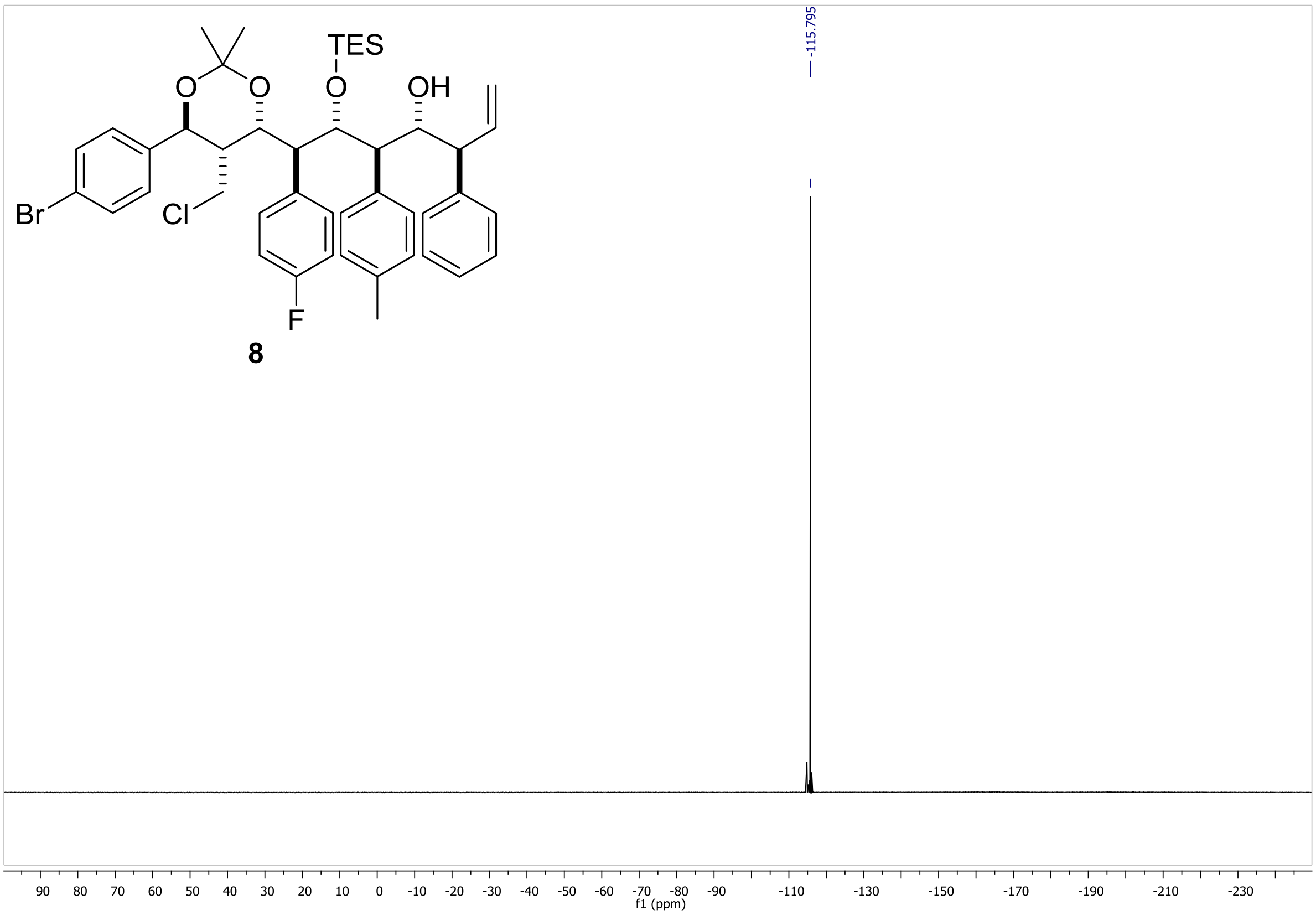

4-(4-bromophenyl)-8-(4-fluorophenyl)-2,2-dimethyl-7-((1-(*p*-tolyl)allyl)tetrahydro-4*H*,5*H*-pyrano[4,3-*d*][1,3]dioxine (9):

^{1}H NMR, 600 MHz, CDCl$_3$:

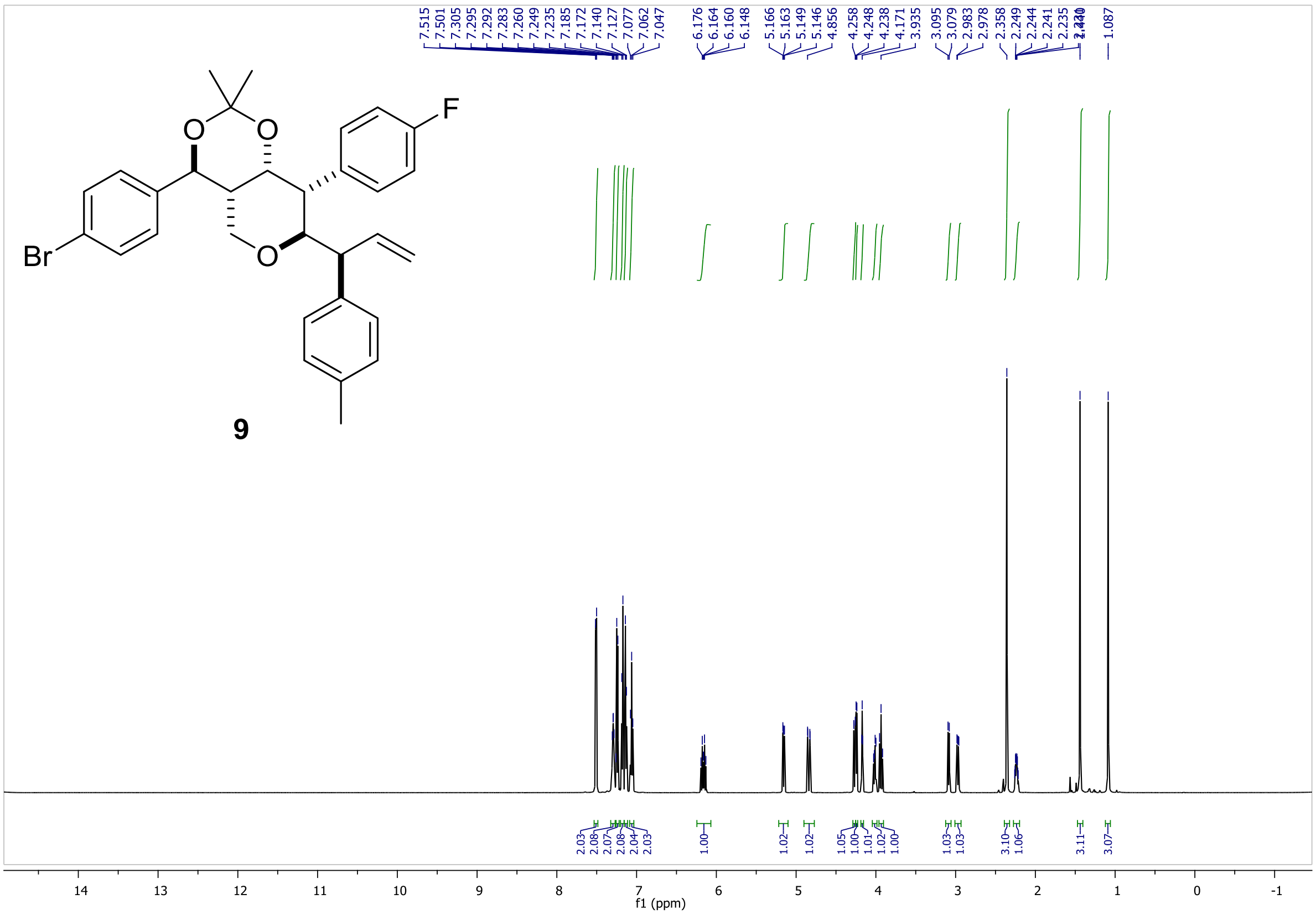

^{13}C NMR, 150 MHz, CDCl$_3$:

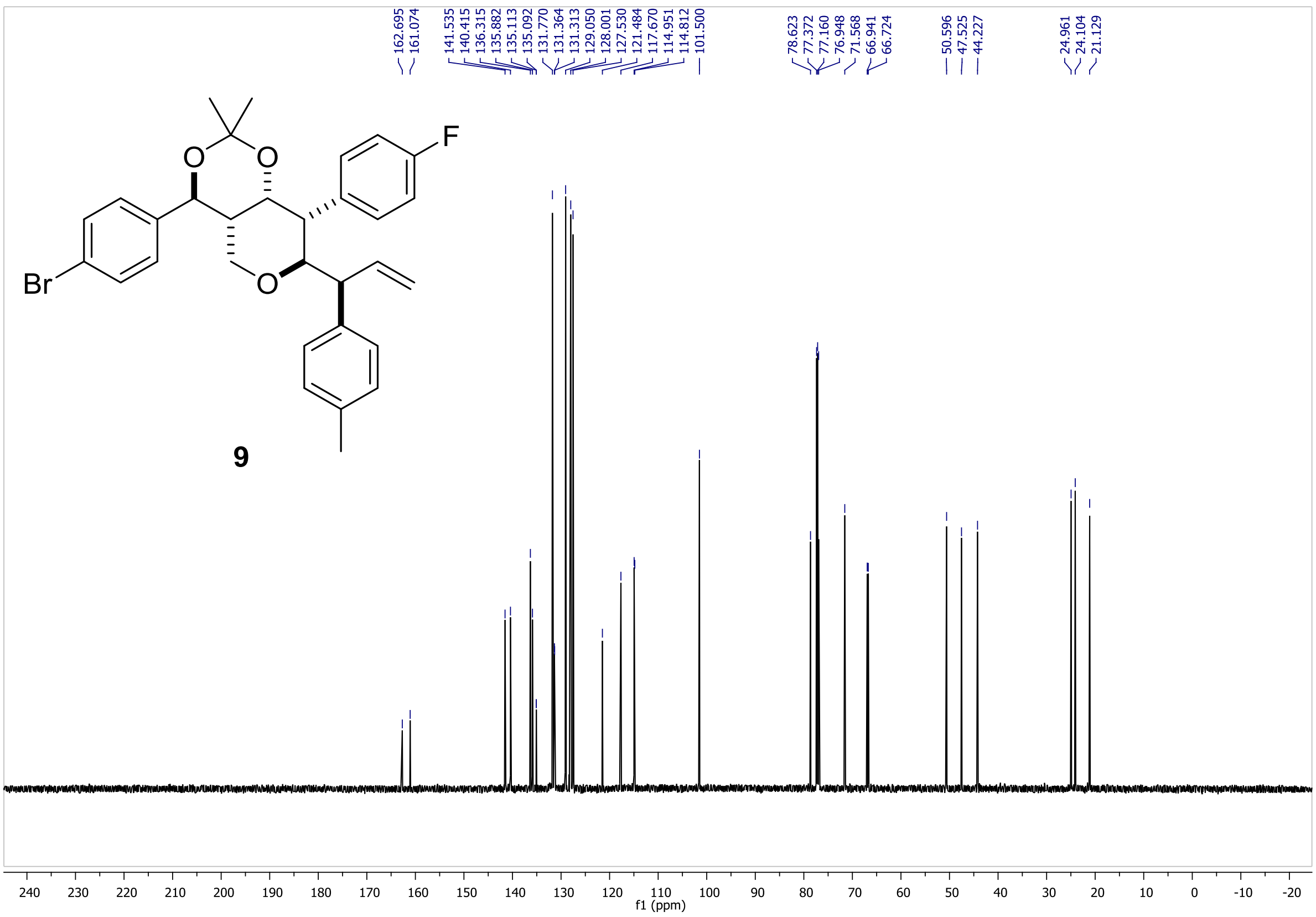

^{19}F NMR, 376 MHz, CDCl$_3$:

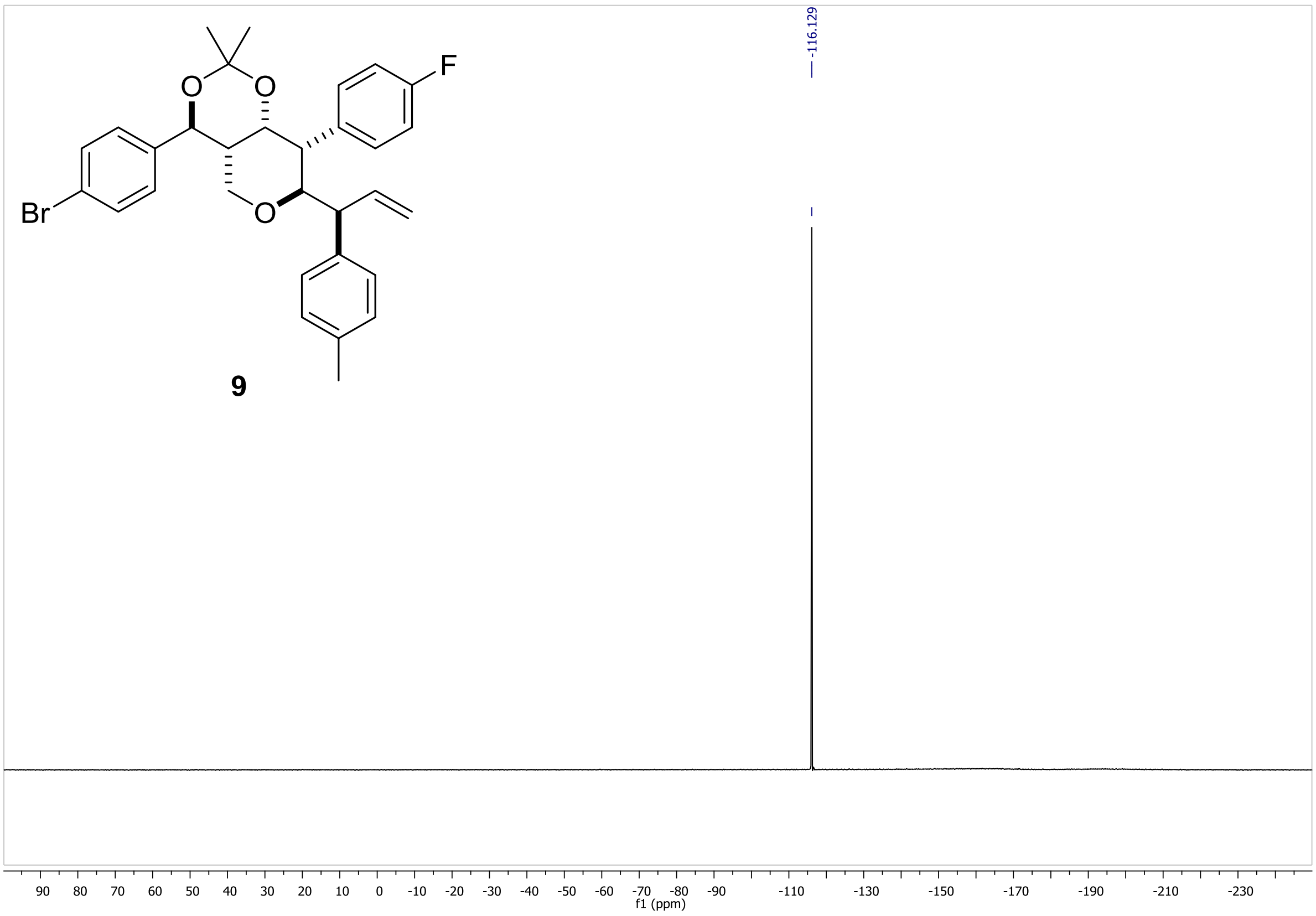

References

[1] http://www.uniqsis.com/
[2] http://www.vapourtec.co.uk/home
